# The mycosis fungoides cutaneous microenvironment shapes dysfunctional cell trafficking, antitumor immunity, matrix interactions, and angiogenesis

**DOI:** 10.1172/jci.insight.170015

**Published:** 2023-10-09

**Authors:** Alyxzandria M. Gaydosik, Connor J. Stonesifer, Tracy Tabib, Robert Lafyatis, Larisa J. Geskin, Patrizia Fuschiotti

**Affiliations:** 1 Department of Medicine, Division of Rheumatology and Clinical Immunology, University of Pittsburgh School of Medicine, Pittsburgh, Pennsylvania, USA.; 2 Columbia University Medical Center, New York, New York, USA.

**Keywords:** Dermatology, Oncology, Skin cancer

## Abstract

Malignant T lymphocyte proliferation in mycosis fungoides (MF) is largely restricted to the skin, implying that malignant cells are dependent on their specific cutaneous tumor microenvironment (TME), including interactions with non-malignant immune and stromal cells, cytokines, and other immunomodulatory factors. To explore these interactions, we performed a comprehensive transcriptome analysis of the TME in advanced-stage MF skin tumors by single-cell RNA sequencing. Our analysis identified cell-type compositions, cellular functions, and cell-to-cell interactions in the MF TME that were distinct from those from healthy skin and benign dermatoses. While patterns of gene expression were common among patient samples, high transcriptional diversity was also observed in immune and stromal cells, with dynamic interactions and crosstalk between these cells and malignant T lymphocytes. This heterogeneity mapped to processes such as cell trafficking, matrix interactions, angiogenesis, immune functions, and metabolism that affect cancer cell growth, migration, and invasion, as well as antitumor immunity. By comprehensively characterizing the transcriptomes of immune and stromal cells within the cutaneous microenvironment of individual MF tumors, we have identified patterns of dysfunction common to all tumors that represent a resource for identifying candidates with therapeutic potential as well as patient-specific heterogeneity that has important implications for personalized disease management.

## Graphical abstract

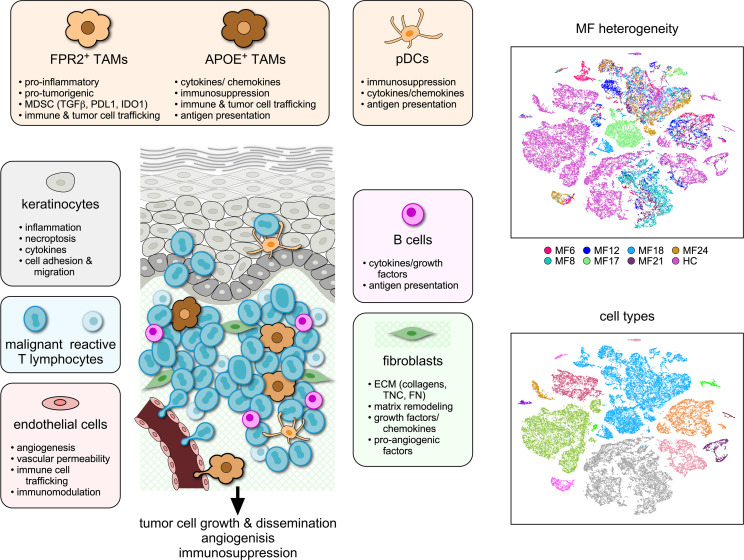

## Introduction

Mycosis fungoides (MF) is the most common subtype of cutaneous T cell lymphoma (CTCL) and is characterized by the clonal expansion of transformed skin-resident memory CD4^+^ T cells (1, 2). Patients diagnosed with early MF develop skin patches and plaques and experience an indolent disease course with a favorable prognosis (2). In the initial phase, most T cells reside in the skin and only a few circulate in peripheral blood and lymph nodes. However, a substantial number of patients progress to an advanced stage with malignant lymphocytes spreading to other sites of the body, which can result in a fatal outcome (2–4).

A striking feature of MF is the restriction of lymphocyte proliferation to the skin, which implies that the malignant cells are dependent on their specific cutaneous tumor microenvironment (TME), including cell-to-cell interactions, spatial distribution, and secreted factors (5, 6). Malignant skin-infiltrating lymphocytes are accompanied by dermal infiltrates of non-malignant T cells as well as other immune and stromal cells (7–9). All these cells produce a variety of cytokines and other immunomodulator factors that affect cutaneous inflammation, and are important constituents of the TME, fostering proliferation, survival, and migration and suppressing tumor cell immunosurveillance.

Advances in single-cell RNA sequencing (scRNA-Seq) (10) allow transcriptional profiling of thousands of individual cells from a large heterogeneous population, such as a patient biopsy. This analysis of cellular heterogeneity offers a unique opportunity to assess the function of individual cells in the context of their microenvironment (11, 12). By scRNA-Seq of MF skin lesions, we (13) and others (14, 15) demonstrated that T lymphocytes in the MF TME display substantial inter- and intra-patient gene expression heterogeneity. However, only recently using new scRNA-Seq methods for high-resolution profiling of the T cell immune repertoire simultaneously with gene expression, we were able to assess the transcriptional profiles of expanded malignant clones and of benign tumor-infiltrating T lymphocytes (TILs) directly in MF samples (16). We observed expansion of several non-overlapping clonotypes within each advanced MF skin sample, including a dominant malignant clone and several less abundant malignant and benign clones. Reactive CD4^+^ and CD8^+^ lineages were characterized by a memory phenotype and the activation of antitumor Th1 and cytotoxic pathways but, conversely, upregulation of checkpoint receptor expression as well as several anti-inflammatory and immunosuppressive mechanisms. We also detected patient-specific immune responses mediated by CXCL13^+^ T follicular helper (TFH) cells, TNFR2 signaling, NRF2-mediated oxidative stress, and ferroptosis. However, these studies did not analyze all immune and stromal cells in the TME of advanced-stage MF patients.

Here we report a comprehensive study including transcriptome analyses of all cell types from skin lesions of patients with advanced-stage MF. Our analysis revealed a distinct MF-specific TME in comparison with skin from healthy controls and benign dermatoses but with extensive interpatient heterogeneity. Major diversity was observed in both the proportion of specific cell types and their gene expression. Focusing on the cell types in the MF TME that exhibit the greatest changes, such as myeloid cells, B cells, keratinocytes, fibroblasts, and endothelial cells, we identified alterations in cellular transcriptomes leading to changes associated with pathways affecting cell trafficking, matrix interactions, angiogenesis, immune functions, and metabolism in patient samples, with dynamic interactions and crosstalk between these cell types and malignant T lymphocytes. We also detected substantial differences in MF TME between patients, indicating that differences in individual MF TMEs may also affect T cell clonal expansions. This work provides a comprehensive view of immune and stromal cell heterogeneity within the skin microenvironment of individual MF tumors and offers important implications for personalized disease management.

## Results

### Single-cell transcriptional profiling of the TME in advanced-stage MF skin tumors.

**Figure 1:**
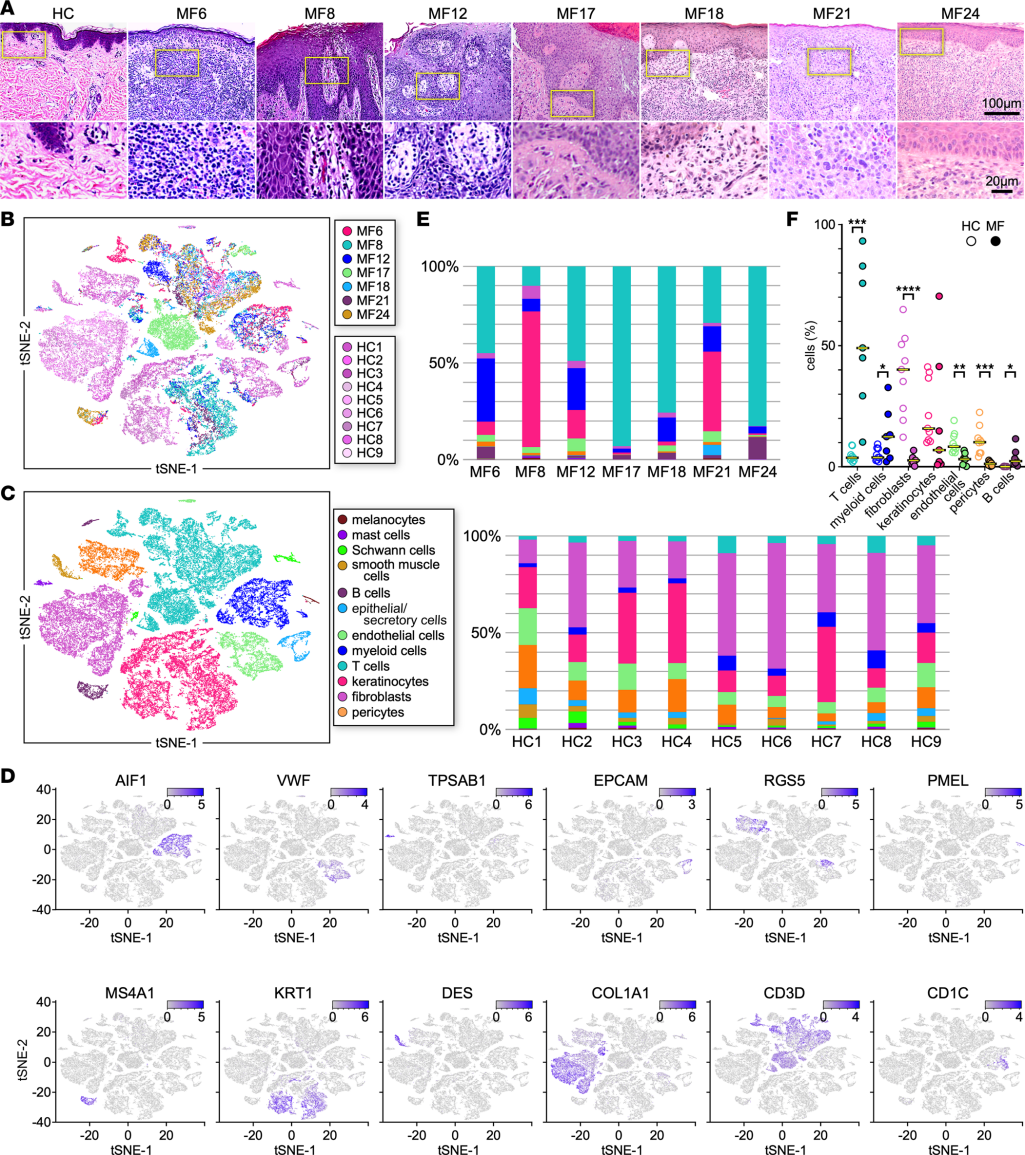
Grouping of MF and HC skin populations. Transcriptomes of 51,468 cells (27,146 from HC [*n* = 9] and 24,322 from MF [*n* = 7] skin samples) were analyzed using Seurat (18, 19). (**A**) H&E staining of skin biopsies from representative healthy control skin (HC) and the 7 MF tumor samples analyzed by scRNA-Seq (top row at ×200, bottom zoomed 3 times). (**B**) Two-dimensional t-distributed stochastic neighbor embedding (t-SNE) shows dimensional reduction of reads from single cells, revealing grouping in each MF sample compared with all HC skin samples. Cells from each subject are indicated by different colors. All samples are combined. (**C**) Distinct gene expression signatures are represented by the clustering of known markers for multiple cell types and visualized using t-SNE (see Methods). Clusters belonging to each cell type are color coded (13). (**D**) Cell types in skin cell suspensions were identified by cell-specific markers as previously described (13); examples are shown. Intensity of purple color indicates the normalized level of gene expression. (**E** and **F**) Proportion of the major cell types identified in **C** and **D** by individual MF or HC samples (**E**) and in all MF or all HC samples combined (**F**). Statistics by unpaired 2-tailed Student’s *t* test (**F**).

By scRNA-Seq we analyzed the cutaneous TME of advanced-stage MF patient samples (Supplemental Table 1; supplemental material available online with this article; https://doi.org/10.1172/jci.insight.170015DS1) that were previously studied to identify T cell heterogeneity and clonality (13, 16). Histopathological evaluation shows a dense mononuclear infiltrate in the epidermis and dermis of all samples tested, including presence of enlarged atypical T lymphocytes and clonal TCRβ population (Figure 1A and Supplemental Table 2).

Experimental procedures and analysis of data followed established procedures as we previously described (13). In total, we analyzed 51,468 cells from enzymatically digested skin of 7 MF (27,146 cells) and 9 healthy control (HC; 24,322 cells) samples. SoupX (17) was used to remove cell-free mRNA contamination from each sample before analysis with Seurat where we used reciprocal principal component analysis to integrate samples for batch correction analysis based on chemistry to group cells according to their expression profiles (18, 19). Groups were visualized by t-distributed stochastic neighbor embedding (t-SNE) dimensional reduction (20). Harmony (21) was used in parallel for comparison, showing that the samples did not suffer from batch effects based on chemistry (Supplemental Figure 1A).

We observed a minimal overlap between the transcriptomes of cells from HC and MF samples, indicating patterns of distinct gene expression in the MF TME (Figure 1B). Moreover, we found that the transcriptional profiles of cells from patient samples coincided only partially with each other, implying intertumor gene expression heterogeneity (Figure 1B). The cell-type composition of the cutaneous microenvironment of MF and HC samples (Figure 1C) was established by the expression of cell-specific marker genes (13, 16) (examples shown in Figure 1D and Supplemental Figure 2). We observed that the greatest gene expression heterogeneity between MF and HC samples, as well as across patient samples, was at the level of T lymphocytes, myeloid cells, keratinocytes, fibroblasts, endothelial cells, and B lymphocytes. We detected heterogeneity not only at the transcriptome level but also in the proportion of the different cell types between patient and HC samples (Figure 1, E and F, and Supplemental Table 8A). Apart from MF8, all other MF samples exhibited higher proportions of T lymphocytes compared with HCs, as expected. We also detected a significant increase in the frequency of myeloid cells in MF samples versus controls. In contrast, the proportions of fibroblasts and endothelial cells/pericytes were significantly decreased in all patients tested compared with HCs. Keratinocytes exhibited a large variability in their proportions among both MF samples, ranging from 0.5% to 32% (Figure 1E), consistent with the pathological features of the tumors, some showing remarkable epidermal hyperplasia (Figure 1A). Finally, although at low frequencies, we detected a significant increase in the number of B cells in the TME of all MF samples compared with HC skin, in which we normally detect few or no B cells. Analysis after batch correction by Harmony (21) showed similar results (Supplemental Figure 1B). Thus, the TME of advanced MF skin tumors presents distinct cell-type compositions and exhibits large intertumor transcriptional heterogeneity within cell types.

### Single-cell RNA-Seq identifies distinct macrophage and dendritic cell subpopulations in the MF TME.

**Figure 2:**
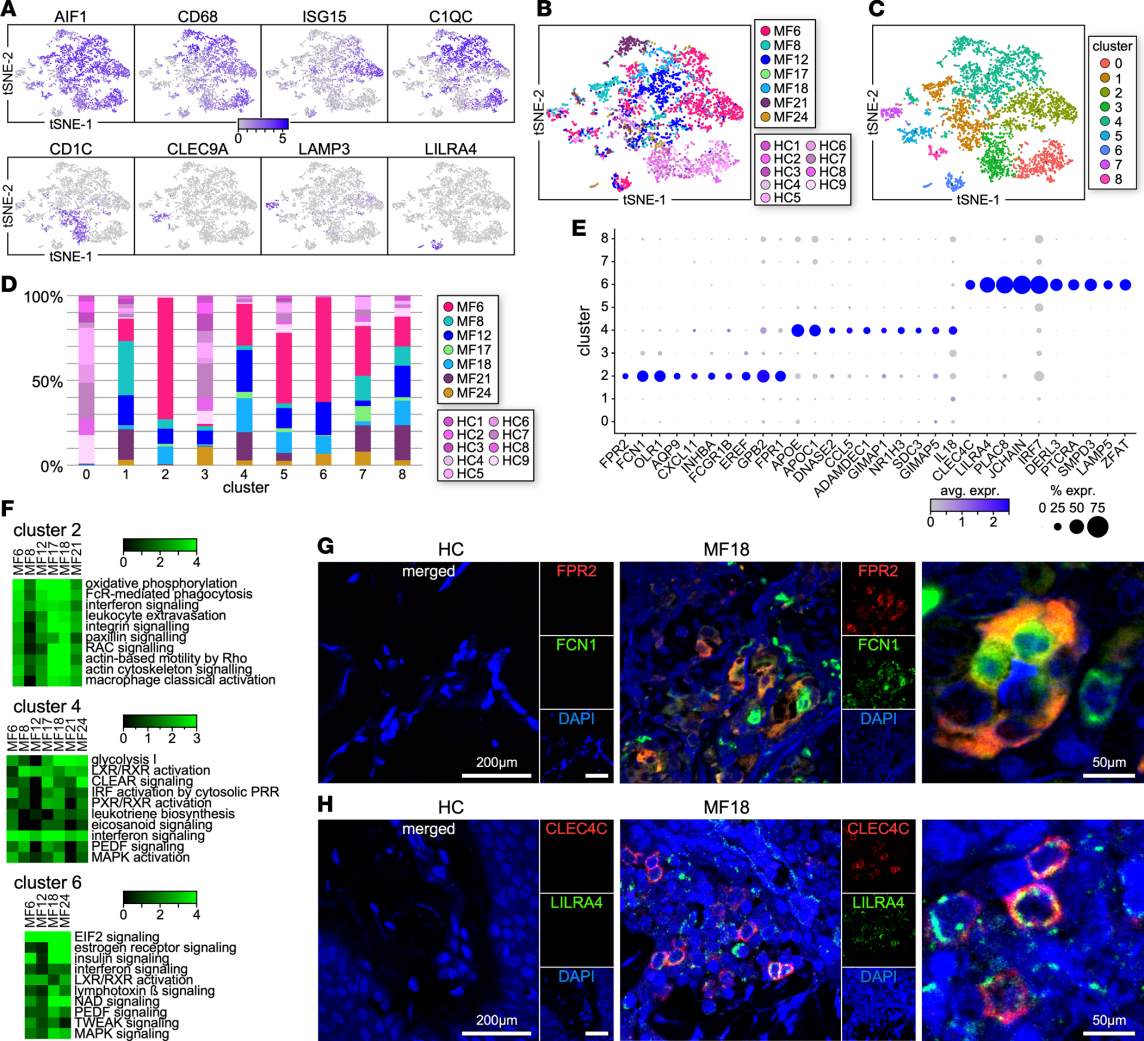
Transcriptional profiles of macrophages and DCs from MF and HC skin samples. (**A**) Expression of macrophage and DC markers by *AIF1*^+^ cells from patient (*n* = 7) and HC (*n* = 9) skin samples. (**B** and **C**) Transcriptomes of 4,425 *AIF1*^+^ cells (1,115 from HC and 3,310 from MF skin samples) (**B**) revealed 9 discrete Louvain clusters (**C**) using Seurat (18, 19). (**D**) Bar plot showing the proportion of cells from each MF or HC sample within individual clusters. (**E**) Dot plot showing the proportion of cells and the scaled average gene expression of signature genes (*n* = 10) from the MF-specific clusters (2, 4, and 6) (**C**). Gene differential tests are described in Methods. (**F**) The differential gene lists were filtered by *P* value < 0.05 for significance and then run in Ingenuity Pathway Analysis (IPA, Qiagen) (78) for significant upregulated pathways. Highly significant examples of distinct pathways activated by the MF-specific clusters are shown. Pathways are represented by enrichment scores (–log *P* values) and selected by absolute *z* scores over 2 (78). (**G** and **H**) Multicolor immunofluorescence microscopy staining for FPR2 and FCN1 (**G**) or CLEC4C and LILRA4 (**H**) in advanced MF (*n* = 7) and HC (*n* = 4) skin samples. Representative examples are shown (×1,000). At right are higher-magnification examples of double-stained cells. DAPI stains nuclei.

In view of their markedly increased numbers in the cutaneous MF TME, we focused on further characterizing the myeloid cells, including macrophages (*AIF1*, *CD68*, *ISG15*, *C1QC*) and dendritic cells (DCs) (*CD1C*, *CLEC9A*, *LAMP3*, *LILRA4*) (Figure 2A). The transcriptional profiles of macrophages and DCs coincided among HC samples as previously described (22); however, the transcriptomes of MF samples generally did not overlap with controls (Figure 2B).

Seurat analysis of combined MF and HC samples identified 9 Louvain clusters (Figure 2C), and we determined the sample composition within each cluster (Figure 2D and Supplemental Table 8B). Most cells from clusters 0 and 3 derived from HC samples, while cells from clusters 2 and 6 were unique to MF tumors. The majority of cells from cluster 4 derived from patient samples (95% of total cells within the cluster), whereas the remaining clusters contained a mix of cells from tumor and healthy skin samples.

Cells from HC clusters 0 (*EMP1*, *CXCL12*, *EGR1*, *CXCL14*) and 3 (*MAP4K4*, *IL1R1*, *IL1R2*, *CCL22*) corresponded to tissue-resident macrophages (22) and conventional DC2 (cDC2) (23), respectively (Supplemental Figure 3). While the IL-6 signaling pathway was prominent in both clusters, cluster 0 also included IL-8, IL-1, and TREM1 signaling, whereas cluster 3 showed activation of TNFR2 and mTOR pathways as well as antigen presentation (Supplemental Figure 3).

The MF-specific macrophage cluster 2 (*FPR2*, *FCN1*, *ORL1*, *AQP9*, *INHBA*, *CXCL11*) contained cells from most MF samples (MF6, MF8, MF12, MF17, MF18, MF21) and upregulated several processes including leukocyte motility and extravasation, IFN signaling, cachexia, and production of reactive oxygen species (Figure 2, E and F, and Supplemental Figure 3). Cells from cluster 2 also expressed markers of myeloid-derived suppressor cells (MDSCs) (24) such as *ORL1*, *TGFB1*, *PTGS2*, *CD274*, *HIF1A*, *IDO1*, and *CD81*. The MF-specific cluster 6 (*CLEC4C*, *LILRA4*, *PLAC8*, *IRF7*, *JCHAIN*, *DERL3*) comprised cells deriving from MF6, MF12, MF18, and MF24 and identified a subset of plasmacytoid dendritic cells (pDCs) (25), upregulating IFN and lymphotoxin β signaling, LRX/RXR activation, NRF2-mediated oxidative stress, and HIF-1α and ferroptosis signaling (Figure 2, E and F, and Supplemental Figure 3). Cells from cluster 4 (*APOE*, *APOC1*, *ADAMDEC1*, *CCL18*, *SDC3*, *C1QB*) identified M2-like macrophages (22) and activated IRF, eicosanoid, and IFN signaling, glycolysis, leukotriene biosynthesis, and complement activation (Figure 2, E and F, and Supplemental Figure 3).

DCs from cluster 1 expressed markers of cDC2 (*CD1C*, *LY6C*, *FCER1A*, *S100A7*), as well as *CD1A*, which characterizes Langerhans cells; however, we found no expression of langerin (CD207) (Supplemental Figure 3), thus identifying a subset of skin migratory cDC2 (26). Pathways such as antigen presentation and glucocorticoid and estrogen receptor signaling were enriched by cells from this cluster (Supplemental Figure 3). Clusters 5 and 7 represent 2 subsets of cDCs: a dermal cDC1 subset (cluster 5, *CLEC9A*, *XCR1*, *BTLA*, *BATF3*) (26) and a LAMP3-DC subset (*LAMP3*, *AOC1*, *IL15*, *CD200*) (26). They activated antigen presentation and phagocytosis (cluster 5) and TNFR2 and PI3K/AKT signaling pathways (Supplemental Figure 3).

Finally, cluster 8 represented a subset of proliferating macrophages and DCs (Supplemental Figure 3). To validate the transcriptional data, we performed immunofluorescence microscopy on multiple advanced-stage MF samples staining for FPR2 and FCN1 to identify MF-specific tumor-associated macrophages (TAMs) and CLEC4C and LILRA4 to identify pDCs. Representative examples depicted in Figure 2, G and H, show high numbers of FPR2^+^FCN1^+^ and CLEC4C^+^LILRA4^+^ cells in MF skin lesions that are absent in HC skin.

Thus, our transcriptome analysis details phenotypic shifts of myeloid cells in the TME of advanced-stage MF, including the prominent expansion of TAMs, M2-like macrophages, and pDC populations.

### B cell transcriptome in the MF TME.

**Figure 3:**
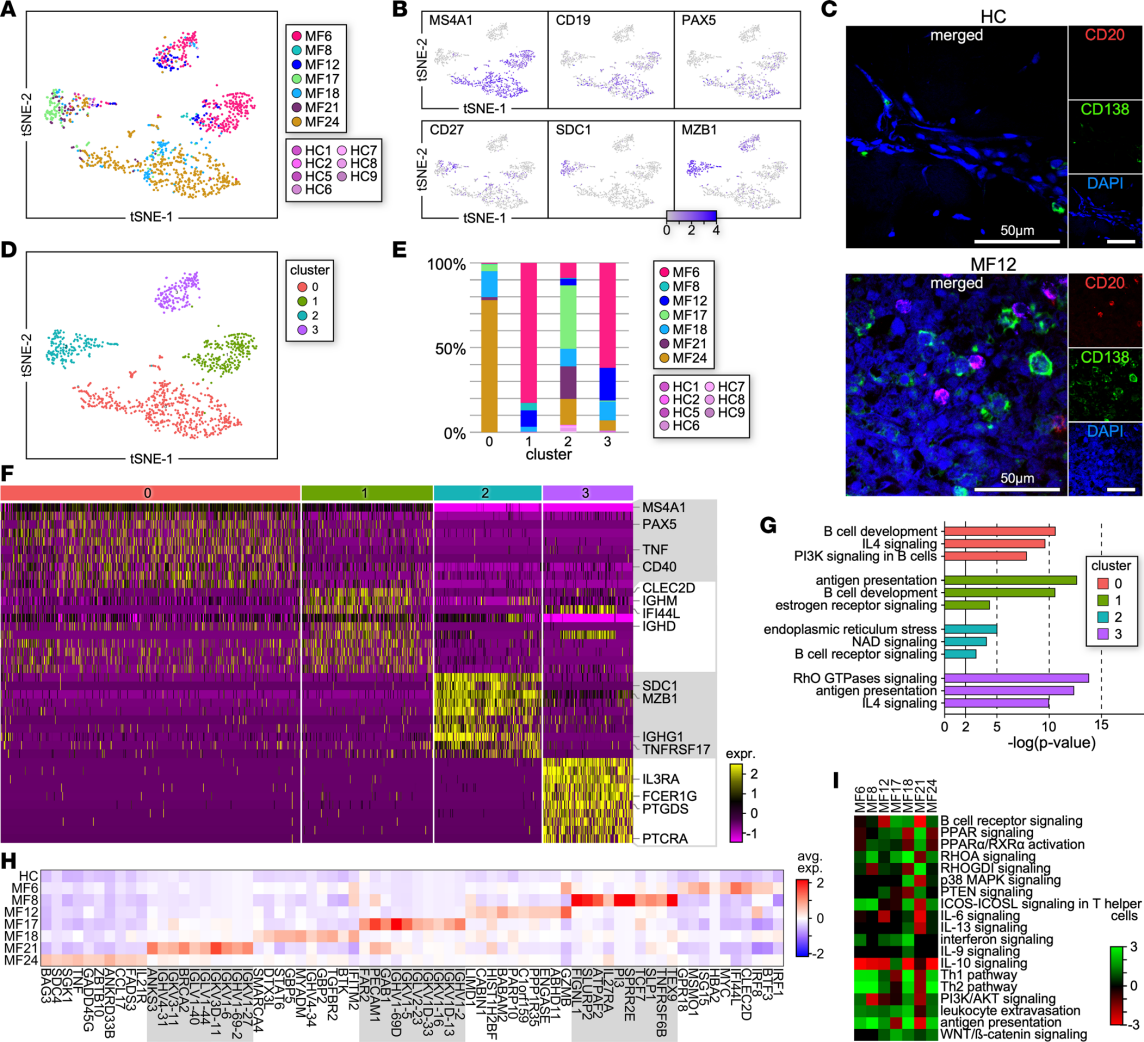
Transcriptional profile of B lymphocytes within individual MF tumors. (**A**) Transcriptomes of 1,018 *MS4A1*^+^ cells (10 from HC and 1,008 from MF samples) revealing grouping in each MF sample (*n* = 7) compared with all HC skin samples (*n* = 9). Cells from each subject are indicated by different colors. All samples are combined. (**B**) Mature memory B cells and plasma cells were identified. (**C**) Multicolor immunofluorescence microscopy staining for CD20 and CD138 in advanced MF (*n* = 7) and HC (*n* = 4) skin samples. Representative examples are shown (×1,000). DAPI stains nuclei. (**D**) Seurat analysis identified 4 discrete Louvain clusters from the B cell data set. (**E**) Bar plot showing the proportion of cells from each MF sample within individual clusters. (**F**) Heatmap showing examples of the most highly significant differentially expressed genes (*n* = 10) for each cluster from **D**. Differential tests were performed as described in Figure 2E and Methods. Cluster numbers are indicated at the top. Each column represents a cell. (**G**) Highly significant examples of upregulated pathways by individual clusters are shown. Pathways are represented by enrichment scores (–log *P* values) and selected by absolute *z* scores over 2. (**H**) Heatmap shows average gene expression of B cell signature genes from individual MF samples versus HCs. Gene differential tests are described in Methods. (**I**) Individual tumors compared with control significant differential expression gene lists (*P* value < 0.05, log fold change 0.1, minimum percentage 10%) were analyzed in IPA and then compared with each other for common pathways. Heatmap shows *z* scores of pathways for up- or downregulation of pathways.

We observed a significant increase in the numbers of B cells in MF skin tumors compared with HC skin, which only rarely contained B cells (Figure 3A and Figure 1, E and F). B cells from MF skin lesions included mature memory cells (*MS4A1*, *CD19*, *PAX5*, *CD27*) (27) as well as long-lived plasma cells (*SDC1*, *MZB1*, *XBP1*, *CD27*) (27) (Figure 3B). Transcriptional data were validated by immunofluorescence microscopy showing large numbers of CD20^+^ (*MS4A1*) B cells and CD138^+^ (*SDC1*) plasma cells in MF skin lesions, whereas no B memory and few plasma cells were detected in HC skin (Figure 3C). Louvain clustering identified specific B cell subpopulations and their sample composition (Figure 3, D and E, and Supplemental Table 8D). We found that the B cell proportions were not comparable among samples but exhibited larger frequencies in some (MF6, MF17, MF24) than in others (MF8, MF12, MF18, MF21). Most cells from cluster 0 (*MS4A1*, *CD40*, *PAX5*, *TNF*) derived from MF24 (78%) and MF18 (15.3%) and upregulated B cell development as well as the IL-4 and PI3K signaling cascades (Figure 3, F and G). Cluster 1 (*IGHD*, *CLEC2D*, *IGHM*, *IFI44L*) comprised cells from MF6 (83%), MF8 (4.4%), MF12 (9.7%), and MF18 (3%) and was enriched in antigen presentation, B cell development, and estrogen receptor signaling pathways (Figure 3, F and G). Plasma cells from cluster 2 (*SDC1*, *CD38*, *TNFRSF17*, *IGHG1*) derived from most MF samples and activated B cell receptor signaling as well as endoplasmic reticulum stress and NAD pathways (Figure 3, F and G). The majority of cells from cluster 3 (*FCER1G*, *PTCRA*, *PTGDS*, *IL3RA*) were from MF6 (62%), MF12 (19.3%), MF18 (11.1%), and MF24 (6%) and upregulated signaling by Rho GTPases and IL-4 as well as antigen presentation (Figure 3, F and G).

However, B cells from individual patient samples also exhibited distinct gene expression (Figure 3H). While B cells from MF18 were enriched in IL-6, IL-13, IL-9, and p38 MAPK signaling, and those from MF17 in IL-10 signaling, we found that B cells from all patient samples variously upregulated several pathways in common (Figure 3I). These included B cell receptor (MF17, MF18, MF24), PI3K/AKT (MF6, MF18, MF24), PPARα/RXRα (MF8, MF12, MF21), and PPAR (MF12, MF17, MF21) signaling. Most patient samples showed activated ICOS/ICOSL, IFN, and WNT/β-catenin signaling as well as leukocyte extravasation, antigen presentation, and Th1/Th2 mechanisms. Thus, accumulation and immunomodulation by memory B and plasma cells may contribute to the tumor permissiveness of MF TME.

### Single-cell transcriptional profile of keratinocytes in the TME of advanced MF.

**Figure 4:**
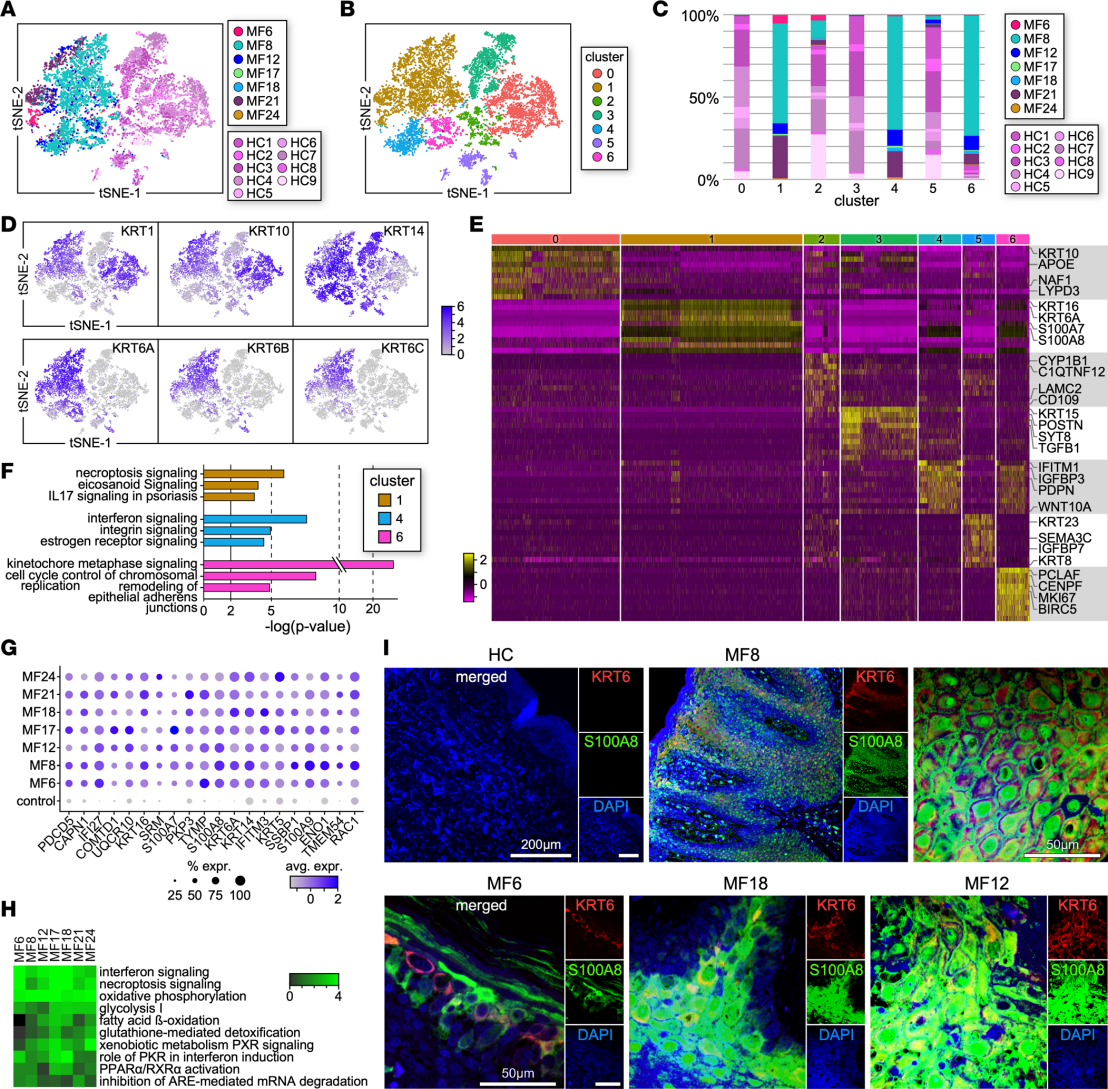
Transcriptional profile of keratinocytes within individual MF tumors. (**A** and **B**) Transcriptomes of 11,773 *KRT1*^+^ cells (5,900 from HC [*n* = 9] and 5,873 from MF [*n* = 7] skin samples) (**A**) revealed 8 discrete Louvain clusters (**B**) using Seurat (18, 19). (**C**) Bar plot showing the proportion of cells from each MF or HC sample within individual clusters. (**D**) t-SNE plots show expression of keratinocyte markers as indicated. (**E**) Heatmap showing examples of the most highly significant differentially expressed genes (*n* = 10) for each cluster from **B**. Cluster numbers are indicated at the top. Each column represents a cell. Differential tests were performed as described in Figure 2E and Methods. (**F**) Highly significant examples of upregulated pathways by individual clusters are shown. Pathways are represented by enrichment scores (–log *P* values) and selected by absolute *z* scores over 2. (**G**) Dot plot showing the proportion of cells and the scaled average gene expression of a panel of genes commonly expressed by KRT1^+^ cells from all MF samples. Gene differential tests are described in Methods. (**H**) The differential gene lists from individual MF samples (*P* value < 0.05, log fold change 0.1, minimum percentage 10%) were run in IPA to compare with each other for common pathways. Heatmap shows *z* scores of pathways for up- or downregulation of pathways. (**I**) Multicolor immunofluorescence microscopy staining for KRT6 and S100A8 in advanced MF (*n* = 7) and HC (*n* = 4) skin samples. Representative examples are shown (×200, ×1,000). DAPI stains nuclei.

Comparison of the keratinocyte transcriptomes between MF and HC samples exhibited a distinct, non-overlapping gene expression (Figure 4A). Louvain clustering identified 7 cell clusters (Figure 4B), of which Figure 4C displays the sample composition. While HC skin samples contained similar proportions of keratinocytes, we observed a large heterogeneity among MF samples (Figure 4C and Supplemental Table 8C). Some MF tumors had low keratinocyte numbers (MF17, MF18, MF24), while others, such as MF8, showed extremely high numbers. Most keratinocyte subpopulations were composed only of cells from HC samples. However, keratinocytes in clusters 1 and 4 were MF specific, and most cells from cluster 6 derived from patient samples (91% of total cells within the cluster). In addition to the expression of the keratinocyte canonical makers such as *KRT1*, *KRT10*, and *KRT14* (28, 29), keratinocytes from all MF samples upregulated expression of *KRT6A*, *KRT6B*, *KRT6C*, *KRT16*, and *KRT17*. In comparison, little or no expression was detected by keratinocytes from HC samples (Figure 4D). Cells from cluster 1 (*KRT6A/B/C*, *S100A7*, *S100A8*, *S100A9*) were specifically enriched in pathways such as necroptosis, eicosanoid signaling, and IL-17 signaling in psoriasis, in line with the MF8 histological pattern of psoriasiform epidermal hyperplasia, whereas cells from cluster 4 (*IFITM1*, *PDPN*, *WNT10A*, *IGFBP3*) upregulated the IFN, estrogen, and integrin signaling cascades (Figure 4, E and F). Finally, cluster 6 (*PCLAF*, *CENPF*, *MKI67*, *BIRC5*) identified proliferating keratinocytes (Figure 4, E and F).

By comparing the differentially expressed genes (DEGs) of keratinocytes among MF samples, we identified 237 genes in common, of which we show a selection of 20 genes that exhibited little or no expression in HC samples (Figure 4G and Supplemental Table 3). These included genes associated with common upregulated pathways such as necroptosis and IFN signaling as well as those associated with several metabolic processes, including glycolysis, fatty acid oxidation, and oxidative phosphorylation (Figure 4H). By immunofluorescence microscopy, we validated the transcriptional phenotype of MF tumor-associated keratinocytes and showed that KRT6A^+^S100A8^+^ are specifically found in the epidermis of advanced MF samples but not in HC skin (Figure 4I). Thus, we observed common upregulation of inflammatory and metabolic processes by tumor-associated keratinocytes as well as keratinocyte heterogeneity in the TME of MF patients.

### Single-cell fibroblast transcriptome in the MF TME.

**Figure 5:**
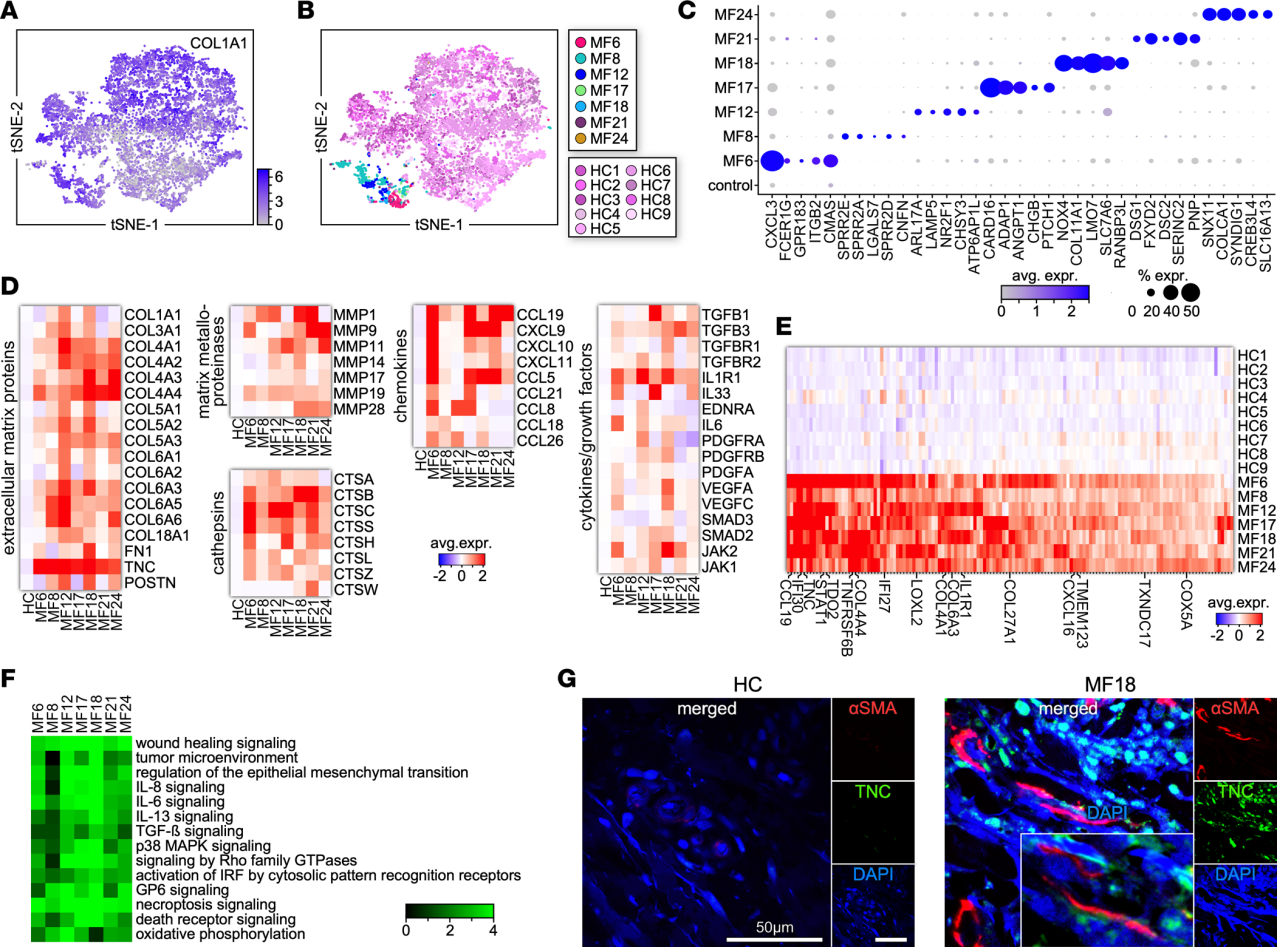
Transcriptional profile of fibroblasts within individual MF tumors. (**A** and **B**) Transcriptomes of 9,533 *COL1A1*^+^ cells (8,748 from HC [*n* = 9] and 785 from MF [*n* = 7] samples) revealing grouping in each MF sample compared with all HC skin samples. Cells from each subject are indicated by different colors. (**C**) Dot plots showing the proportion of cells and the scaled average gene expression of signature genes (*n* = 5) from fibroblasts from individual tumors versus HC cells. (**D**) Average expression levels of marker genes for ECM proteins, matrix metalloproteinases, and cytokines/chemokines by fibroblasts across individual MF samples. (**E**) Heatmap shows average expression of genes (*n* = 165) commonly expressed by all MF samples. Each tumor was compared with the controls for significant differential expression (*P* value < 0.05, log fold change 0.1, minimum percentage 10%) to find common genes between tumors. Examples of commonly expressed genes are shown. (**F**) Heatmap depicts highly significant (*P* < 0.05) examples of upregulated pathways activated by fibroblasts from each MF sample; *z* scores are shown. (**G**) Multicolor immunofluorescence microscopy staining for αSMA and TNC in advanced MF (*n* = 7) and HC (*n* = 4) skin samples. Representative examples are shown (×1,000); inset zoomed ×2. DAPI stains nuclei.

Fibroblasts were identified by the expression of COL1A1 (Figure 5A and Figure 1E). Although fibroblasts from patient samples exhibited a distinct gene expression compared with HCs, their proportions were dramatically decreased (Figure 5B; Figure 1, E and F; Supplemental Figure 4B; and Supplemental Table 8E). To better characterize fibroblast heterogeneity across patient samples, we compared their DEGs across MF samples and HCs. We found that fibroblasts from individual patient samples exhibited distinct gene expression signatures (Figure 5C), including genes associated with inflammation, chemotactic activity, and adhesion. Moreover, MF fibroblasts heterogeneously expressed various genes previously identified in cancer-associated fibroblasts from other human cancers (30), including genes encoding extracellular matrix (ECM) proteins, matrix metalloproteinases, profibrotic factors, chemokines, and cathepsins (Figure 5D). However, fibroblasts from all MF but not from HC samples expressed 165 genes in common (Figure 5E and Supplemental Table 4). This signature included genes associated with IFN signaling (*IFI27*, *IFI30*, *STAT1*, *EPSTI1*), ECM production (*COL4A1*, *COL6A3*, *TNC*, *LOXL2*), metabolism (*TDO2*, *LAP3*, *APOL1*, *PARP14*), and inflammation (*TNFRSF21*, *IL32*, *CTSC*, *PSMB9*). Notably, all samples upregulated several processes in common, including wound healing and IL-6, IL-8, IL-13, TGF-β, VEGFA, PDGF, FGF, IGF-1, and IGF-2 signaling cascades (31, 32). Other relevant pathways included epithelial-mesenchymal transition, necroptosis, and death receptor signaling (Figure 5F). We validated the transcriptional data by immunofluorescence microscopy, staining for α-smooth muscle actin (αSMA) and TNC to identify ECM-producing myofibroblasts, which were absent in HC skin (Figure 5G). Thus, MF fibroblasts secrete multiple pro-tumorigenic factors that facilitate tumor cell spreading, promote angiogenesis, and regulate immune infiltration.

### Single-cell atlas of endothelial cell phenotypes in the MF TME.

**Figure 6:**
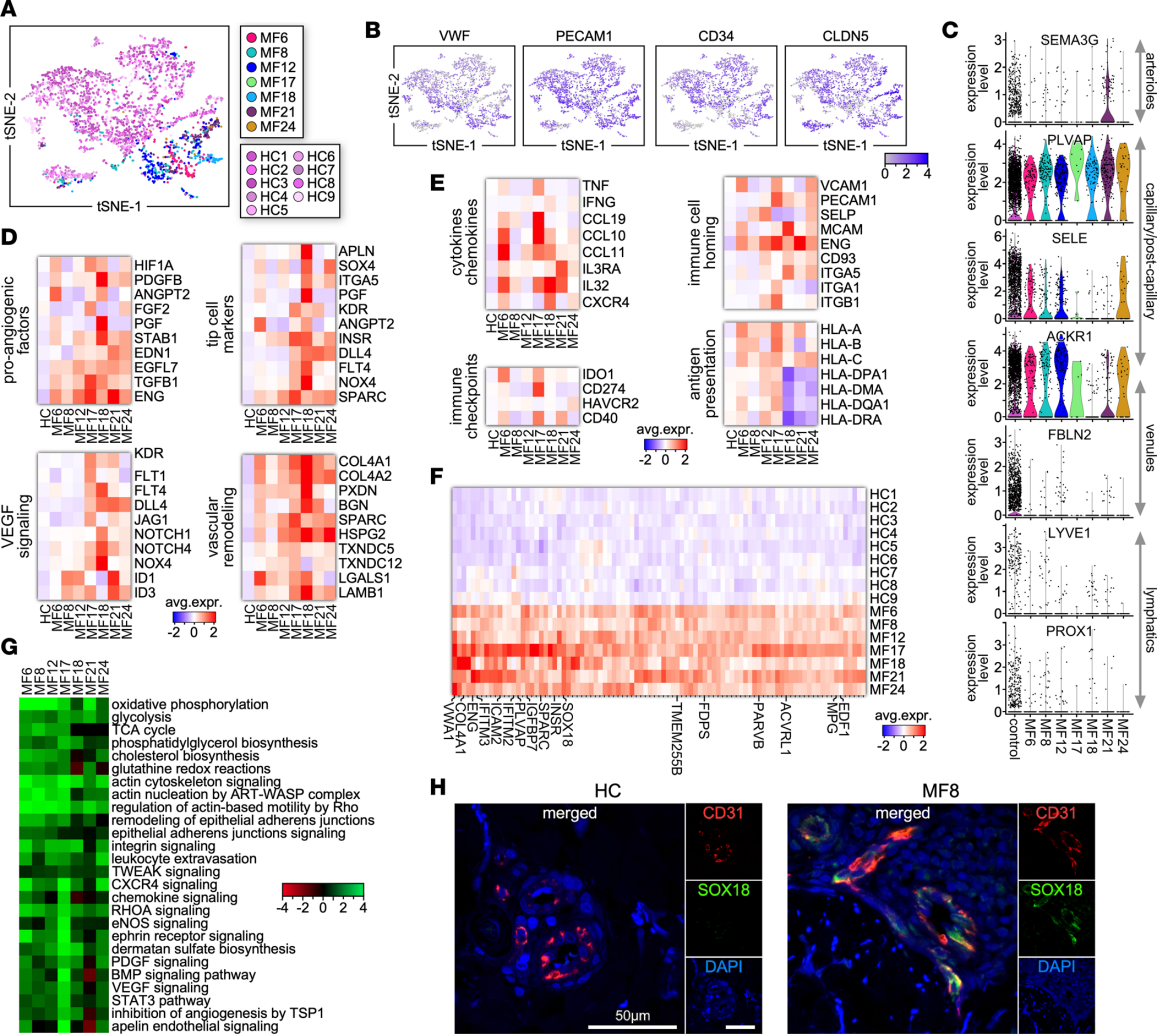
Single-cell EC transcriptome in the MF TME. (**A**) Transcriptomes of 3,319 *VWF*^+^ cells (2,503 from HC [*n* = 9] and 816 from MF [*n* = 7] samples) revealing grouping in each MF sample compared with all HC skin samples. Cells from each subject are indicated by different colors. (**B**) t-SNE plots show expression of indicated EC marker genes. (**C**) Dot plots showing the proportion of cells and the scaled average gene expression of cutaneous EC markers from individual MF and HC samples as indicated. (**D** and **E**) Average expression levels of marker genes for pro-angiogenic (**D**) and immunomodulatory (**E**) factors by ECs from individual MF samples. (**F**) Heatmap shows average expression of genes (*n* = 265) commonly expressed by all MF samples. Each tumor was compared with the controls for significant differential expression (*P* value < 0.05, log fold change 0.1, minimum percentage 10%) to find common genes between tumors. Examples of commonly expressed genes are shown. (**G**) Individual tumors compared with control significant differential expression gene lists (*P* value < 0.05, log fold change 0.1, minimum percentage 10%) were analyzed in IPA and then compared with each other for common pathways. Heatmap shows *z* scores of pathways for up- or downregulation of pathways. (**H**) Multicolor immunofluorescence microscopy staining for CD31 and SOX18 in advanced MF (*n* = 7) and HC (*n* = 4) skin samples. Representative examples are shown (×1,000). DAPI stains nuclei.

Endothelial cells (ECs) were also proportionally decreased in patient samples compared with HCs and exhibited a non-overlapping transcriptional profile (Figure 6A; Figure 1, E and F; Supplemental Figure 5; and Supplemental Table 8F). ECs were selected based on canonical marker expression (*VWF*, *PECAM1*, *CD34*, *CLDN5*) (Figure 6B) and showed enrichment of distinct skin subpopulations (33) (Figure 6C). These included *SEMA3G^+^* arterioles mostly found in MF21, and capillary/postcapillary ECs (*PLVAP*, *ACKR1*, *SELE*) that showed increased PLVAP expression by all MF samples except MF6 compared with individual HC samples. However, most of the latter exhibited increased expression of *SELE*, important for neutrophil rolling in postcapillary venules. Markers for venule (*FBLN2*, *ACKR1*) and lymphatic (*LYVE1*, *PROX1*) ECs were mostly detected in control samples. Notably, ECs from MF samples exhibited increased expression of angiogenic and immunomodulatory gene signatures. The angiogenic phenotype was more prominent in MF17, MF18, and MF21 and included the expression of genes encoding pro-angiogenic factors, endothelial tip cell markers, VEGF and NOTCH signaling molecules, and vascular membrane remodeling factors (Figure 6D). We also detected heterogeneous immune-activated phenotypes including expression of cytokines and chemokines, adhesion molecules, MHC molecules, and immune checkpoints (Figure 6E). ECs from all MF samples expressed 265 genes in common that encoded pro-angiogenic factors (e.g., *ENG*, *VWA1*, *FDPS*, *SOX18*) as well as factors contributing to EC homeostasis (*FAM167B*, *IGFBP7*, *COL4A1*, *MGP*), microvascular permeability, and leukocyte recirculation (*PLVAP*, *ICAM2*, *PARVB*, *ACVRL1*) (Figure 6F and Supplemental Table 5). Notably, ECs for all MF patients upregulated various pathways in common, including glycolysis, mitochondrial respiration, and lipid metabolism as well as cell motility and vascular permeability (Figure 6G). All MF samples apart from MF8 upregulated leukocyte extravasation and integrin signaling as well as multiple pro-angiogenic pathways, including those regulated by ephrin, PDGF, VEGF, BMP, and apelin. By immunofluorescence microscopy, we confirmed the transcriptional data, visualizing pro-angiogenic ECs (CD31^+^SOX18^+^) in microvascular areas of the TME in several MF patient samples, whereas ECs from HC samples were negative for SOX18 expression (Figure 6H). Thus, MF ECs contribute to a tumor-permissive TME by upregulating pro-angiogenic and immunomodulatory functions.

### Comparison of the cutaneous microenvironment between MF and benign dermatoses.

**Figure 7:**
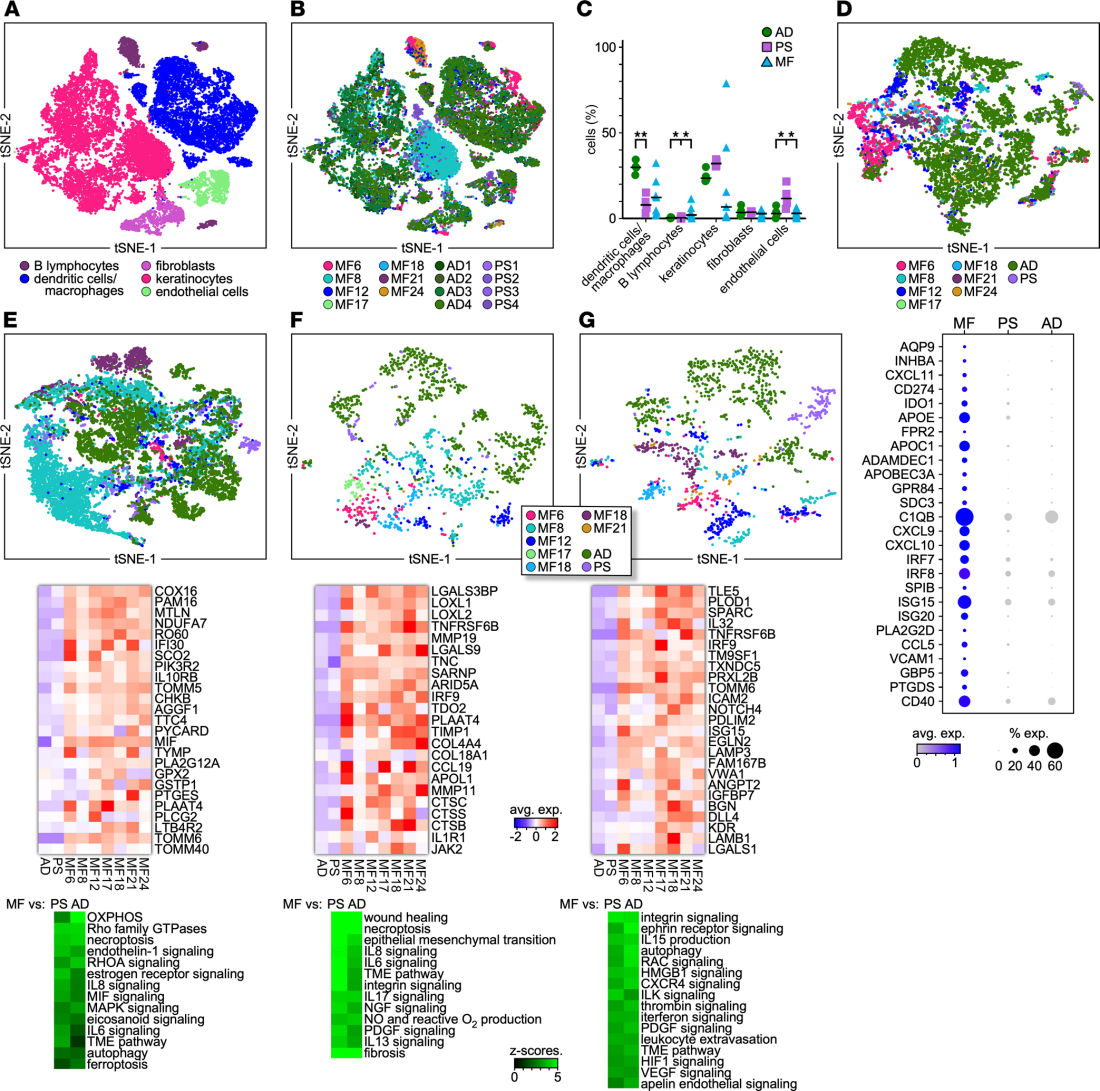
Integration of single-cell data sets from MF and benign dermatoses. (**A**) t-SNE plot showing fibroblast, keratinocyte, B cell, myeloid cell, and EC identifications resulting from reciprocal principal component analysis integration (see Figure 1, C and D, and Methods). Clusters belonging to each cell type are color coded (13). (**B**) t-SNE plot showing grouping in each MF sample (*n* = 7) compared with AD (*n* = 4) and PS (*n* = 4) skin samples for the cell types of interest. Cells from each subject are indicated by different colors. (**C**) Proportion of the cell types identified in **A** by individual MF, AD, or PS samples. Statistics by Student’s *t* test. (**D**) Top panel: Transcriptomes of AIF1^+^ myeloid cells revealing grouping in each MF sample compared with all AD/PS skin samples combined. Cells from each subject are indicated by different colors. Bottom panel: Dot plot shows examples of MF-specific gene expression. (**E**–**G**) t-SNE plots depicting the transcriptional profile of keratinocytes (**E**), fibroblasts (**F**), and ECs (**G**) from MF and AD/PS skin samples (top panels). Corresponding heatmaps (lower panels) depict average expression of select genes commonly expressed by all MF samples and highly significant (*P* < 0.05) examples of upregulated pathways activated by the indicated cell types from MF versus AD/PS samples; *z* scores are shown.

To further investigate the MF cell types analyzed above, we integrated our MF data sets with publicly available scRNA-Seq data from benign inflammatory skin diseases including atopic dermatitis (AD) (34) and psoriasis (PS) (35). Integration of the data sets showed co-clustering of these subpopulations from MF and AD/PS samples as well as partial overlap in their transcriptional profiles (Figure 7, A and B). As we observed in the comparison with HC skin, we detected an increased proportion of B cells in the MF skin lesions compared with AD/PS, which exhibited only rare B cells. The MF TME also exhibited a lower proportion of ECs compared with PS (Figure 7C). Notably, while the proportions and transcriptomes of each of these subpopulations exhibited a large heterogeneity across MF patients, these were very homogeneous in both AD and PS patients (Figure 7, A and C).

We first aimed to profile differences in the phenotype and effector functions of myeloid cells between MF and AD/PS samples. Although we noticed a considerable overlap between the transcriptional profiles of MF and AD myeloid cells, we also found MF-specific gene expression that included signatures also identified in the comparison with HC skin (Figure 2 and Figure 7D). These include gene signatures of FPR2-macrophages and MDSCs as well as of M2-like macrophages and pDCs (Figure 7D, bottom panel). Accordingly, we detected MF-specific activation of classical and alternative macrophage processes, various cytokine signaling (IFN, IL-8, IL-33), and complement activation, as well as production of nitric oxide and reactive oxygen species (Supplemental Figure 6).

As observed in the MF comparison with HC skin samples, integration of MF and AD/PS keratinocyte data sets revealed distinct MF8 and MF21 transcriptional profiles (Figure 4 and Figure 7E). Similarly, we detected activation of multiple proinflammatory and metabolic pathways in MF keratinocytes but not in the AD/PS samples. Other examples of upregulation specific to MF keratinocytes included necroptosis, ferroptosis, and endothelin-1, IFN, and eicosanoid signaling as well as oxidative phosphorylation, glutathione redox, and glycolysis (Figure 7E).

Comparison of the MF and AD/PS fibroblast data sets showed MF differential expression of multiple genes associated with wound healing and fibrosis, including collagens, TNC, LOXL, and MMP enzymes, and signaling through various cytokines and growth factors such as IL-6, IL-8, IL-13, IL-17, IFN, and PDGF (Figure 5 and Figure 7F). In further agreement with the comparison with HC skin, we also detected increased expression of cathepsins, chemokines, adhesion molecules, and galectins.

Lastly, integration of the EC data sets showed an extensive overlap between the MF comparisons with AD/PS and with HC skin (Figure 6 and Figure 7G). In common, multiple processes were specifically upregulated in MF samples that included pro-angiogenic pathways such as those mediated by VEGF, PDGF, apelin, CXCR4, ephrin, and ILK as well as those linked to alterations in permeability, vasomotor tone, and leukocyte trafficking (Figure 7G).

Together, integration of our data sets with those from benign dermatoses validates the results of our comparison with HC skin and further emphasizes the role of the advanced MF TME in promoting dysfunctional cell trafficking, antitumor immunity, matrix interactions, and angiogenesis.

### T lymphocyte–focused intercellular communication in the TME of advanced MF.

**Figure 8:**
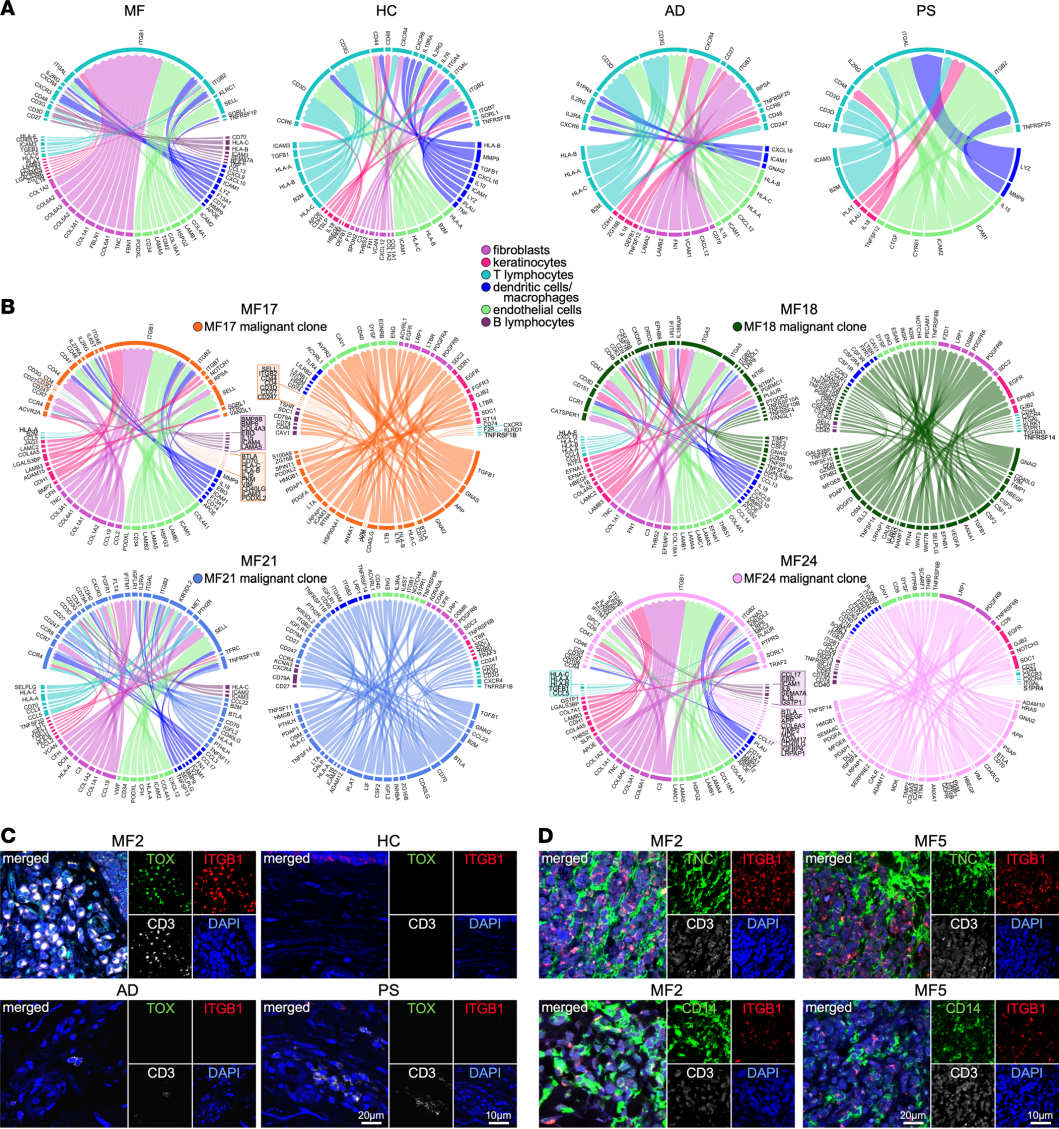
T lymphocyte–focused intercellular communication in the TME of advanced MF. Visualization of the T cell interactions with cell of interest (macrophages/DCs, B cells, keratinocytes, fibroblasts, and ECs) in the cutaneous microenvironment of MF and controls. Circos plots of top 10 interactions between ligand cell type and receptor cell type (edges) are shown. (**A**) Top 10 significant T cell interactions in MF (*n* = 7), HC (*n* = 9), AD (*n* = 4), and PS (*n* = 4) skin samples combined. (**B**) Top 10 significant incoming and outgoing interactions between T cells from the malignant expanded clone (clonotype 1) with the cells of interest and benign T cells are depicted. Four independent tumor samples are shown. In **A** and **B**, edge thickness is proportional to weight scale, which is larger when an edge is more highly associated with a specific cell type–cell type. Edge color labels the source cell type. In all Circos plots, ligands occupy the lower semicircle and corresponding receptors the upper semicircle, and ligands and receptors are colored by the expressing cell type. In all cases, the network shown has been limited to those edges in which the ligand and receptor are both expressed in more than 10% of their respective clusters and have a *P* value less than 0.05. The full results of the connectomic analysis can be found in Supplemental Table 6. (**C** and **D**) Multicolor immunofluorescence microscopy staining for CD3, ITGB1, and TOX in advanced MF (*n* = 5), HC (*n* = 4), AD (*n* = 3), and PS (*n* = 3) skin samples (**C**) and CD3, ITGB1, and TNC (top panels) or CD14 (bottom panels) in advanced MF (*n* = 5) skin samples (**D**). Representative examples are shown (×1,000). DAPI stains nuclei.

We analyzed our scRNA-Seq data with the Connectome package (36) to predict the cell-to-cell interactions in the MF TME. Using a unique database (FANTOM5) (36, 37) of known ligand-receptor interactions, we mapped the interactions between T lymphocytes and the cell types of interest analyzed above. We first compared the cutaneous microenvironments between each of the sample types (MF, HC, AD, PS) by combining specimens and focused on the top 10 interactions (Figure 8A and Supplemental Table 6, A–D). Notably, we showed that none of the interactions predicted in the MF TME are also predicted in the HC, AD, or PS cutaneous microenvironments (Figure 8A). Several HLA-mediated B cell–T cell interactions were predicted in MF samples, likely associated with antigen presentation, while other interactions, such as CD70-CD27, ICAM3-ITGAL, ICAM3-ITGB2, and TNF-TNFRSF1B, could mediate T cell activation, adhesion, and anti-apoptotic signals. Cell adhesion and trafficking were the major predicted processes deriving from the interactions between myeloid cells, ECs, fibroblasts, keratinocytes, and MF T cells, likely promoting cell adhesion and trafficking. These were mostly mediated by chemokines, adhesion molecules, and ECM proteins. Interestingly, various ligands from MF keratinocytes (ADAM15, COL7A1, LAMB3, LGALS38P), fibroblasts (COL1A1/2, COL3A1, COL5A2, COL6A1–3, FBLN1, FBN1, TNC), ECs (LAMA5, COL18A1, COL4A1, HSPG2, LAMB1, TGM2), myeloid cells (CD14, F13A1), and B cells (SEMA7A) were predicted to interact with ITGB1 on T lymphocytes. By focusing on a subset of MF tumor samples (MF17, MF18, MF21, MF24) for which we recently determined the transcriptional profile of the dominant malignant clones (clonotype 1) by scRNA-Seq (16), we predicted that these interactions occur with ITGB1 on malignant T lymphocytes (Figure 8B and Supplemental Table 6, E–H). However, while these interactions are predicted to be significant for MF18 and MF21, they are not within the top 10 and thus do not appear in Figure 8B. Immunofluorescence microscopy was used to validate select predictions. We first showed that ITGB1 expression was detected on malignant T cells that were identified by TOX positivity in advanced MF skin lesions, whereas no CD3^+^TOX^+^ITGB1^+^ cells were detected in HC or in AD/PS skin samples (Figure 8C). Secondly, we validated the interactions of CD3^+^TOX^+^ITGB1^+^ cells with TNC and CD14^+^ cells in the MF TME (Figure 8D).

By further analyzing the cell-to-cell interactions between the malignant T lymphocytes and the cells exhibiting the greatest heterogeneity in the MF TME, as above, we detected heterogeneous cell-to-cell interactions to and from the malignant cells within individual samples. Notably, although several signaling families and processes were commonly activated across samples, these derived from heterogeneous ligand-receptor interactions (Figure 8B and Supplemental Table 6, C–F). Most predicted interactions between B cells and the malignant clones were mediated by adhesion molecules and cytokines (IL-27, IL-6, IL-16, IL-15), while myeloid cells from all tumors were predicted to interact with the malignant clones via various chemokines (CCL3, CCL13, CXCL10, CXCL9, CCL17), matrix metalloproteinases (MMP9, MMP12), and cytokines (IL-18, EBI3). Keratinocytes interacted very heterogeneously with tumor cells from individual samples. While we detected interactions involving lamins and ephrins in most patients, the specific ligand-receptor interactions varied. Fibroblasts from all samples were predicted to interact with malignant cells via collagens, fibronectin, and tenascin, while interactions via chemokines and complement components were specific to some samples. Conversely, malignant cells were predicted to activate fibroblasts by secreting various cytokines and growth factors. In ECs, cell-to-cell adhesion with malignant cells was predicted via lamins, collagens, and matrix glycoproteins in most MF samples, while clonal malignant cells mediated several pro-angiogenic interactions by TGFB1, VEGFA, NAMPT, EFNB2, and other factors according to the patient sample. Chemokine–chemokine receptor interactions were the major estimated mechanism of communication between non-clonal and malignant T cells within each sample.

Collectively, this analysis creates a comprehensive portrait of potential intercellular interactions between malignant T cells and immune and stromal cells in the MF TME that may facilitate tumor cell growth and dissemination, angiogenesis, and immunosuppression.

## Discussion

Lymphocyte proliferation in MF is largely restricted to the skin, implying that malignant cells are dependent on their specific cutaneous microenvironment. Cytokines and other immunomodulatory factors produced by malignant lymphocytes and TILs as well as by other immune and stromal cells affect cutaneous inflammation, and are important constituents of the TME, fostering survival and proliferation of tumor cells and suppression of the tumor-specific immunosurveillance. ScRNA-Seq offers a unique opportunity for dissecting alterations in the microenvironment of MF and predicting how these changes might influence the migration, retention, and growth of the malignant T cells. Here, we present a single-cell atlas of advanced-stage MF patient skin tumors as compared with HC skin and benign dermatoses. In addition to common alterations in the microenvironment between tumors, our analysis revealed transcriptional diversity in immune and stromal cells across patient samples, with dynamic interactions and crosstalk between these cells and malignant T lymphocytes.

Chemokine–chemokine receptor interactions are the major mechanism of communication predicted between TILs and malignant T cells in all MF samples, promoting tumor cell trafficking and homing to the skin. A common predicted interaction involved CCL5 binding to CCR4 that is highly expressed on malignant T cells at all stages of CTCL, thus recruiting them at the tumor site. In addition, CCL5 may also recruit immunosuppressive Tregs and Th2 cells to further promote tumor progression (38). Notably, a fully humanized anti-CCR4 monoclonal antibody, mogamulizumab, is currently used for the treatment of relapsed/refractory MF and Sézary syndrome (SS) (39). Other predicted interactions included those between HLA class I molecules on TILs and KIR3DL1/2 on malignant cells of most patients that may result in an anti-apoptotic effect (40). Conversely, malignant cells established several patient-specific interactions with TILs within the same tumor. Examples of such interactions include TNFSF14-TNFRSF14 (MF18) that may exert an antitumor immune response by promoting the generation of tertiary lymphoid structures (41) or LTα-TNFR2 (*TNFRSF1B*) (MF21, MF17) on highly suppressive TNFR2^+^ Tregs (42). Moreover, ZG16B-CXCR4 (MF21, MF24) and TGFB1-CXCR4 (MF18, MF21) signaling in TILs may induce immunosuppression by cytotoxic T cell exclusion and by recruiting Foxp3^+^ Tregs (43).

We identified two MF-specific macrophage subpopulations in the MF TME. The first subset (FPR2^+^ TAMs) exhibited dichotomous functional phenotypes characterized by the expression of proinflammatory and pro-tumorigenic pathways. Interestingly, cells from this cluster also expressed markers of MDSCs (24), which may primarily exert their immunosuppressive and pro-tumorigenic function by expressing high levels of TGF-β and PDL1 as well as by upregulating IDO1 and HIF-1α pathways associated with metabolic reprogramming (24, 44). Although TAMs and MDSCs are considered separate, they share many characteristics and are developmentally connected (45, 46). Recent reports indicated that accumulation of MDSCs may be crucial in CTCL progression (47), and Geskin et al. observed a reduction in MDSC activity following IFNα2b therapy (48), suggesting a possible correlation with the effective treatment by IFN. The second MF-specific macrophage subset (APOE^+^ TAMs) identified M2-like TAMs (49), upregulating *MRC1*, *CSF1R*, *CD81*, chemokines, matrix remodeling, cathepsins, complement, eicosanoid, and apolipoprotein genes, all contributing to promote immunosuppression, tumor cell extravasation, migration, survival, and proliferation (50–52). Our analysis also identified a subset of tumor-infiltrating pDCs in most of the MF samples that expressed *TGFB1*, *ICOSLG*, and *LILRA4*. These genes were previously associated with pDC dysfunction in other human cancers that promoted an immunosuppressive TME by acting as negative regulators of type I IFN production via TGF-β and ILT7 (*LILRA4*) signaling (53). In addition, the induction of Tregs through the ICOS/ICOSL pathway could potentially create a vicious cycle by producing additional TGF-β (54) that further intensifies the immunosuppressive effect of the tumor (55, 56).

The MF samples presented a statistically significant increase in the frequency of activated memory B cells and terminally differentiated plasma cells compared with HC skin and benign dermatoses, which had few or no B cells. Accordingly, recent studies have shown that B cell infiltration correlates with disease progression in CTCL (57–59). Besides the secretion of antibodies and cytokines and regulation of lymphocyte trafficking, our data indicate that B cells from individual patient samples heterogeneously exerted a multitude of functions in the MF TME, including upregulation of antigen presentation and B cell differentiation and signaling as well as production of immunosuppressive cytokines. Accordingly, B cell–derived TNF is predicted to interact with TNFRSF1B (TNFR2) on MF24 T lymphocytes, consistent with our demonstration of TNF/TNFR2 signaling pathway activation in reactive MF24 CD4^+^FOXP3^+^ Tregs and CD8^+^ T cell effectors (16). Notably, recurrent point mutations and genomic gains of *TNFRSF1B* have been identified in more than a third of patients with MF and SS (60), which have been implicated in T cell survival and proliferation. While we did not detect a substantial activation of the TNFR2 signaling cascade in malignant cells in the samples tested, analysis on a larger cohort of patients by scRNA-Seq coupled with TCR immune profiling will be necessary to ascertain this. Emerging data indicate that B cells may also have an anti-tumorigenic role (61), and Gu-Trantien et al. (62) recently identified a subset of CXCL13^+^ TFH cells that recruited B cells in the TME of breast tumors, which is associated with a better prognosis. Similarly, we have identified a subset of non-malignant CD4^+^CXCL13^+^ T cells in MF18 that recruited B cells in peritumoral areas (16).

Emerging data highlighted the importance of the crosstalk between the malignant and stromal cells in CTCL (7). Notably, our analysis revealed heterogeneous proportions and transcriptomes of 3 stromal cell types: keratinocytes, fibroblasts, and endothelial cells. While we detected higher proportions of keratinocytes in patient samples exhibiting epidermal hyperplasia, keratinocytes from all samples were enriched in several metabolic pathways such as glycolysis, oxidative phosphorylation, and fatty acid oxidation. This increased energy production may be critical for controlling the activation and differentiation of keratinocytes as observed in other chronic inflammatory skin diseases (63) and for providing the bioenergetic support for tumor cells through the generation of metabolic intermediates and oxidative stress components. Keratinocytes from all MF samples upregulated the expression of keratins 6a/b/c, 16, and 17. These keratins are induced in stressed keratinocytes upon skin injury (64). Their upregulation leads to the rapid induction of “alarmins,” such as the S100 family of proteins, as well as proinflammatory cytokines/chemokines, and alters proliferation, cell adhesion, and migration of keratinocytes, contributing to hyperproliferation and innate immune activation (64). Accordingly, MF keratinocytes exhibited activation of necroptosis and upregulation of genes encoding several alarmins (*S100A7A*, *S1008A*, *S1009A*) and signaling pathways fueling necroptosis. In contrast, a previous study showed a decreased expression of alarmins that correlated with predisposition to *Staphylococcus aureus* infection in CTCL skin (65), suggesting that the role of alarmins in MF tumorigenesis needs further investigation. While the predicted keratinocyte–malignant cell interactions were heterogeneous among patient samples, most involved lamins, collagens, matrix glycoproteins, and ephrins, likely promoting cell-to-cell adhesion and migration of tumor cells. Interestingly, several molecules from these families interacted with ITGB1 on MF17 and MF24 malignant cells. Notably, ITGB1 upregulation and signaling were detected in patients with leukemic CTCL (66) and were associated with tumor cell trafficking, survival, proliferation, and resistance to apoptosis (66).

Despite fibroblasts being significantly decreased in the TME of MF patient samples, they exhibited an intense crosstalk with tumor lymphocytes that likely facilitates cancer progression. MF fibroblasts showed a large intertumor heterogeneity, but all samples presented a wound healing–activated myofibroblast phenotype characterized by de novo expression of αSMA. While we observed heterogeneous expression of various ECM proteins across patients, all highly produced tenascin, which is linked to tumor neovascularization and tissue-localized immunosuppressive activities (67). Notably, a histological criterion for CTCL diagnosis is dermal fibrosis, an aberrant deposition of ECM components in the dermis (68). ECM not only promotes tumor cell growth and migration but also regulates cell-to-cell and cell-matrix crosstalk. Accordingly, MF fibroblasts upregulated several remodeling enzymes and cathepsins, likely involved in fine-tuning the cell-matrix interactions. MF fibroblasts also produced various cytokines and growth factors that induce fibroblast activation and wound healing, stimulate ECs to develop tumor angiogenesis, and promote epithelial-mesenchymal transition. Additionally, they could support immune evasion by inducing the secretion of multiple immunosuppressive cytokines and chemokines that recruit malignant and Th2 cells to skin.

Increased microvessel density (MVD) in CTCL lesional skin correlates with aggressive disease subtypes and disease progression (69). Our analysis, however, showed a decreased number of ECs in all samples that was confirmed by the histological examination. While this discrepancy may reflect patient heterogeneity of tumor stroma, quantification of CTCL MVD varies among different studies due to differences in scoring methodology (70). Nonetheless, our analysis agrees with previous studies in showing that ECs from most patients exhibited a pro-angiogenic phenotype. While the VEGF/VEGFR axis is induced via autocrine mechanisms in ECs from only some patient samples (MF17, MF18, MF21), other cells in the tumor stroma secrete multiple factors that activate VEGF, BMP, PDGF, CXCR4, and apelin signaling in ECs of most samples. Moreover, ECs from all patients highly expressed *TGFB1* and its coreceptor *ENG*, which could induce angiogenesis via direct VEGFR2 activation (71, 72). TGF-β is also a major inducer of endothelial-mesenchymal transition, a critical process in stimulating migration and invasion of tumor cells as well as suppressing antitumor immunity (72). Our data indicate that ECs from all MF samples highly express *PLVAP*, a key regulator of vascular permeability (28). Vessel leakiness in the TME may not only contribute to the metastatic process but could also lead to extravasation of fluids and proteins, resulting in hypoxia and the release of pro-angiogenic factors through the direct interaction of HIF-1α with VEGFR2 in ECs (71, 72). While the hypoxic TME enhances glycolysis in MF ECs, they also generate energy through oxidative phosphorylation and lipid metabolism. Additionally to promoting angiogenesis, MF ECs can modulate trafficking of immune cells into the tumor stroma. Although we observed a heterogeneous expression of various selectins, integrin ligands, and adhesion molecules by ECs from individual patient samples, they all appear to favor the recruitment of immunosuppressive rather than immune effector cells, a process referred to as “endothelial anergy” (73). Examples include upregulation of *STAB1* (CLEVER1), which favors the influx of Tregs and TAMs, downregulation of *VCAM1* and *ICAM1*, or secretion of soluble adhesion molecules (*MCAM*, *ENG*) that inhibit the recruitment of effector CD4^+^ and CD8^+^ T cells. Although MF ECs could function in antigen presentation, they lack expression of *CD80* and *CD86* (not shown), thereby promoting CD4^+^ T cell unresponsiveness (71). Furthermore, MF ECs heterogeneously upregulate various inhibitory molecules, including *CD274* (PDL1), *HAVCR2* (TIM3), and IDO1, to directly inhibit T cell activation and suppress antitumor immunity. However, several studies have reported expression of inhibitory checkpoint molecules by malignant MF T lymphocytes (74), and functional studies will be necessary to investigate the immunomodulatory role of ECs in MF pathogenesis.

Mycosis fungoides is a rare cancer, which limits sample availability and homogeneity. Although all samples analyzed were derived from patients with advanced-stage MF and most of them were not receiving any treatment at the time of biopsy, all patients underwent prior and diverse treatments, which may affect the heterogeneity of the TME. Nonetheless, compared with HC skin or benign dermatoses, we robustly detected common patterns of abnormal transcription in MF that may be used as a basis for functional predictions. Thus, our characterization of the TME and its interactions with malignant lymphocytes offers critical insights for developing treatments targeted to TME components that may be further personalized to specific patients.

## Methods

### Patients and skin biopsies.

Skin samples (4 mm punch biopsies) were obtained from 10 patients with a confirmed diagnosis of advanced MF (stage IIB–IVA) at the Comprehensive Skin Cancer Center, Columbia University Medical Center. Patients were well characterized in terms of demographic, disease type, clinical features, and therapy as described in Supplemental Tables 1 and 2. Patients were staged according to the most recent consensus (2). Of these 10 samples, 7 were used for scRNA-Seq experiments and for validation by immunofluorescence microscopy, while 3 were used exclusively for validation. Controls included healthy control skin samples (HC, *n* = 9) obtained from age- and sex-matched donors at the Health Sciences Tissue Bank, University of Pittsburgh.

### Single-cell RNA sequencing and analysis.

Experimental procedures (Supplemental Methods) followed established techniques (13, 16) using the Chromium Single Cell 5′ Library V1 kit and the Chromium Single Cell 3′ Library V2 kit (10x Genomics). RNA-Seq was performed using the Illumina NovaSeq6000 system. Cell-gene unique molecular identifier counting matrices generated were analyzed using Seurat 3.1 to identify distinct cell populations using Louvain clustering (18, 19, 75–77). SoupX (17) was used to remove cell-free mRNA contamination from each sample before analysis with Seurat. The default parameters were used to calculate the contamination fraction per ref. 17. The corrected matrix was loaded into Seurat for further analysis. Reciprocal principal component analysis was used to integrate samples for batch correction analysis based on chemistry. For cell-type identification, we used the FindAllMarkers function in Seurat, which uses Wilcoxon’s rank sum test to show differential genes with a minimum percentage of cells of 25% per cluster, log fold change >0.25, and *P* value less than 0.05 for significance. For gene differential tests, we used the FindMarkers function, which uses Wilcoxon’s rank sum test to show differential genes with a minimum percentage of cells of 10% per identity requested, log fold change >0.1, and *P* value less than 0.05 for significance (75–77). This was used for each differential gene list throughout the analysis (75–77). To identify patterns of DEGs across patient samples, each tumor was compared with the healthy skin samples and the resulting DEG lists were compared with the other tumor samples for common and unique genes. Harmony (21) was used to compare batch analysis tools.

### Data set integration.

Inter–data set integration was performed using reciprocal principal component analysis in Seurat for batch correction analysis based on chemistry. Cell-gene unique molecular identifier counting matrices generated were analyzed using Seurat to identify distinct cell populations using Louvain clustering (18, 19, 75–77).

### Connectome.

Ligand-receptor interactions were analyzed using Connectome v1.0.0. FANTOM5 ligand-receptor data were used for mapping. Ligand-receptor interactions were filtered for minimum percentage 10% within cell types, *P* value less than 0.05, and *z* scores over 0 to remove negative source/sink values for significance. Circos plots were used to show up to 10 top significant interactions between individual cell types (36).

### Pathway analysis.

The differential gene lists were filtered for *P* value less than 0.05 for significance and then run in Ingenuity Pathway Analysis (IPA, Qiagen) for significant upregulated pathways. Pathways were selected by enrichment scores (–log *P* values) and absolute *z* scores over 2 (78).

### Multicolor immunohistochemistry.

Multicolor staining was performed on formalin-fixed, paraffin-embedded skin samples using the tyramide signal amplification kit (Thermo Fisher Scientific) as previously described (13). The antibodies used in these experiments are reported in Supplemental Table 7. Confocal images were captured on an Olympus Fluoview 1000 confocal microscope using an oil immersion ×100 objective.

### Statistics.

For analysis of single-cell data, relevant statistical analyses are indicated in the respective sections of Methods. A *P* value of 0.05 was used throughout to determine significance. Statistical differences of cell-type proportions between MF and HC samples within the skin microenvironment were estimated by unpaired 2-tailed Student’s *t* test.

### Study approval.

Clinical information and biological specimens were deidentified and coded. Research protocols involving humans were approved by the Institutional Review Board of Columbia University. All participants gave written informed consent in accordance with the Declaration of Helsinki.

### Data availability.

All scRNA-Seq data generated in this study were deposited in the Gene Expression Omnibus (GEO) database under accession numbers GSE206123 and GSE182861. The AD and PS data sets analyzed in this study are available in the GEO database under accession numbers GSE153760 and GSE151177. Data used in the Figures and Supplemental Figures are available in the Supporting Data Values file.

## Author contributions

AMG and TT performed experiments and analyzed data. LJG and CJS acquired samples and collected clinical descriptions. RL analyzed data and prepared the manuscript. PF developed the project, analyzed data, and prepared the manuscript.

## Supplementary Material

Supplemental data

Supporting data values

## Version 1 09/05/2023

In-Press Preview

## Version 2 10/09/2023

Electronic publication
