# Supplementary material for: The mycosis fungoides cutaneous microenvironment shapes dysfunctional cell trafficking, antitumor immunity, matrix interactions, and angiogenesis
Source: JCI Insight. 2023 Oct 9;8(19):e170015. doi: 10.1172/jci.insight.170015 (PMC10619438; doi:10.1172/jci.insight.170015)
Supplement: Supplemental data [file jciinsight-8-170015-s119.pdf]

The mycosis fungoides cutaneous microenvironment shapes dysfunctional cell trafficking, anti-tumor immunity, matrix interactions and angiogenesis

Alyxzandria M. Gaydosik, Connor J. Stonesifer, Tracy Tabib, Robert Lafyatis, Larisa J. Geskin and Patrizia Fuschiotti

Supplementary Materials

Supplemental Methods

Supplemental Figures 1-6

Supplemental Tables 1-8

Supplemental Methods

Single cell cDNA and library preparation.

Cell suspensions from skin were obtained as previously described (1). Briefly, skin biopsies were minced and digested enzymatically (Whole Dissociation Skin Kit, Miltenyi Biotec) for 2 hours at 37°C and further dispersed using the Miltenyi gentle MACS Octo Dissociator. A 3mm skin biopsy from each donor yielded 3,016-5,332 cells from tumor samples and 1,138-3,953 cells from HC skin. Experimental procedures followed established techniques (1) using the Chromium Single Cell 5' Library V1 kit (10x Genomics) and the Chromium Single Cell 3' Library V2 kit (10x Genomics). Cell suspensions were separated by the Chromium System (10X Genomics) (1-3) into mini-reaction "partitions" or GEMs formed by oil micro-droplets, each containing a gel bead and a cell. A 1000-fold excess of partitions compared to cells assured that most partitions/GEMs had only one cell per GEM. Gel beads contained an oligonucleotide scaffold composed of an oligo-dT section for priming reverse transcription, and barcodes for each cell (10X Genomics) and each transcript (unique molecular identifier, UMI), as described (4). 7,000 cells were loaded into the instrument to obtain data on ~4,000 cells with a rate of ~3.1% of partitions showing more than one cell/partition. The following steps were all performed using reagents and protocols developed by 10X Genomics. Following GEM formation, the emulsions were transferred from the Chromium chip to a PCR cycler for cDNA synthesis. The emulsion was then broken using a recovery agent, and following Dynabead and SPRI clean up, cDNAs were amplified by PCR (C1000, Bio-Rad). cDNAs were sheared enzymatically into lengths of ~200bp. The libraries were quantified using the KAPA Library Quantification Kit (Illumina), and further characterized for cDNA length by bioanalyzer using a High Sensitivity DNA kit.

RNA sequencing.

RNA-seq was performed on each sample by the UPMC Genome Center (https://upmcgenomecenter.com/research-offerings/) using the NovaSeq 6000 sequencing system (Illumina). Genes detected (6,000 genes/cell) had a plateau at about 200,000 reads/cell (10X Genomics, white paper). We obtained ~200 million reads/sample (NovaSeq, Illumina) through the UPMC Genome Center, Sequencing Facility.

Data Analysis.

Chromium scRNA-seq data produced by the 10X Chromium Platform were processed to generate sample-specific fastq files. Processed reads were then examined by quality metrics, mapped to a reference human genome using RNA-seq aligner STAR and assigned to individual cells of origin according to the cell specific barcodes, using the Cell Ranger pipeline (10X Genomics). To ensure that PCR amplified transcripts were counted only once, only single UMIs were counted for gene expression level (5). In this way, cell x gene UMI counting matrices were generated for downstream analyses. SoupX (6) was used to remove cell free mRNA contamination from each sample before analyzing with Seurat. The default parameters were used to calculate the contamination fraction as per reference. The corrected matrix was loaded into Seurat for further analysis. Seurat, an R package developed for single-cell analysis (7-11), was used to identify distinct cell populations and visualize cell clusters in graphs as in (2). Specifically, the UMI matrix was filtered such that only cells expressing at least 200 genes were utilized in downstream analysis. Samples were filtered for percentage of mitochondrial gene content under 25% and unwanted sources of variation were regressed out of the data by constructing linear models to predict gene expression based on the number of UMIs per cell as well as the percentage of mitochondrial gene content. Reciprocal PCA (RPCA) was utilized to integrate samples for batch correction analysis based on chemistry. Each set of samples was analyzed to find the top 2,000 highly variable genes and run principal component analysis (PCA) per chemistry. Integration anchors were then found for each group to combine the chemistries without batch effects. Cells were clustered using Seurat (Louvain clustering) (7). The resultant clusters were then visualized using a t-distributed stochastic neighbor embedding (t-SNE) projection plot (12). For cell-type identification, we used the “FindAllMarkers” function in Seurat, which uses the Wilcoxon rank sum test to show differential genes with a minimum percentage of cells of 25% per cluster, log-fold change >0.25, and p-value < 0.05 for significance. For gene differential tests throughout the analysis, we used the “FindMarkers” function in Seurat, which uses the Wilcoxon rank sum test to show differential genes with a minimum percentage of cells of 10% per identity requested, log-fold change >0.1, and p-value < 0.05 for significance (9-11). Harmony (13) was used to compare batch analysis tools.

Dataset integration

Inter-dataset integration was performed using Reciprocal PCA (RPCA) in Seurat for batch correction analysis based on chemistry. Cell-gene unique molecular identifier counting matrices generated were analyzed using Seurat to identify distinct cell populations using Louvain clustering (7-11).

Connectome

Ligand-receptor interactions were analyzed using Connectome v1.0.0. FANTOM5 ligand-receptor data was used for mapping. Ligand-receptor interactions were filtered for min percentage 10% within cell types, p-value <0.05, and z-scores over 0 to remove negative source/sink values for significance. CircosPlots were used to show up to 10 top significant interactions between individual cell types (14).

Identification of the malignant tumor clonotype

We identified the malignant expanded clonotypes in MF samples (MF17, MF18, MF21, MF24) in a previous study (Gaydosik, A et al, 2022 Clin Cancer Res.). Briefly, we employed recent advances in scRNAseq methods that allow high resolution profiling of the T-cell immune repertoire simultaneously with gene expression, thus allowing the transcriptional profiles of expanded clonotypes to be assessed. With this approach, we showed that several non-overlapping clonotypes are expanded in the skin, including a more abundant dominant clonotype as well as additional less abundant minor clones. The distinction between malignant and benign clones is based on the gene expression and pathway upregulation by individual clones. The distinct gene expression and enrichment for specific oncogenic pathways previously reported by other human cancer studies clearly indicated the malignant nature of some clones while other clones present a transcriptional profile resembling benign reactive T cells.

Data availability

All scRNA-seq data generated in this study have been deposited in the Gene Expression Omnibus database under accession numbers: GSE206123 and GSE182861. The AD and PS datasets analyzed in this study is available in the GEO database under the accession numbers: GSE153760 and GSE151177.

Pathway analysis

The differential gene lists were filtered for p-value <0.05 for significance and then run in Ingenuity Pathway Analysis (IPA, Qiagen) for significant up-regulated pathways. Pathways were selected by enrichment scores (-log p-values) and absolute z-scores over 2 (15).

Multicolor immunohistochemistry

Multi-color staining was performed on formalin-fixed, paraffin-embedded skin samples using the tyramide signal amplification kit (ThermoFisher) as previously described (1). The antibodies employed in these experiments are reported in Table S7. Confocal images were captured on an Olympus Fluoview 1000 confocal microscope using an oil immersion 100X objective.

References

1. Gaydosik AM, Tabib T, Geskin LJ, Bayan CA, Conway JF, Lafyatis R, et al. Single-Cell Lymphocyte Heterogeneity in Advanced Cutaneous T-cell Lymphoma Skin Tumors. Clin Cancer Res. 2019;25(14):4443-54.
2. Macosko EZ, Basu A, Satija R, Nemesh J, Shekhar K, Goldman M, et al. Highly Parallel Genome-wide Expression Profiling of Individual Cells Using Nanoliter Droplets. Cell. 2015;161(5):1202-14.
3. Gaydosik AM, Stonesifer CJ, Khaleel AE, Geskin LJ, Fuschiotti P. Single-cell RNA sequencing unveils the clonal and transcriptional landscape of cutaneous T-cell lymphomas. Clin Cancer Res. 2022.
4. Zheng GX, Terry JM, Belgrader P, Ryvkin P, Bent ZW, Wilson R, et al. Massively parallel digital transcriptional profiling of single cells. Nature communications. 2017;8:14049.
5. Islam S, Zeisel A, Joost S, La Manno G, Zajac P, Kasper M, et al. Quantitative single-cell RNA-seq with unique molecular identifiers. Nat Methods. 2014;11(2):163-6.
6. Young MD, Behjati S. SoupX removes ambient RNA contamination from droplet-based single-cell RNA sequencing data. Gigascience. 2020;9(12).
7. Satija R, Farrell JA, Gennert D, Schier AF, Regev A. Spatial reconstruction of single-cell gene expression data. Nat Biotechnol. 2015;33(5):495-502.

8. Stuart T, Butler A, Hoffman P, Hafemeister C, Papalexi E, Mauck WM, 3rd, et al. Comprehensive Integration of Single-Cell Data. Cell. 2019;177(7):1888-902 e21.
9. Soneson C, Robinson MD. Bias, robustness and scalability in single-cell differential expression analysis. Nat Methods. 2018;15(4):255-61.
10. Butler A, Hoffman P, Smibert P, Papalexi E, Satija R. Integrating single-cell transcriptomic data across different conditions, technologies, and species. Nat Biotechnol. 2018;36(5):411-20.
11. Hao Y, Hao S, Andersen-Nissen E, Mauck WM, 3rd, Zheng S, Butler A, et al. Integrated analysis of multimodal single-cell data. Cell. 2021;184(13):3573-87 e29.
12. van der Maarten L, Hinton G. Visualizing Data using t-SNE. Journal of Machine Learning Research. 2008;9:2579-29605.
13. Korsunsky I, Millard N, Fan J, Slowikowski K, Zhang F, Wei K, et al. Fast, sensitive and accurate integration of single-cell data with Harmony. Nat Methods. 2019;16(12):1289-96.
14. Raredon MSB, Yang J, Garritano J, Wang M, Kushnir D, Schupp JC, et al. Computation and visualization of cell-cell signaling topologies in single-cell systems data using Connectome. Sci Rep. 2022;12(1):4187.
15. Kramer A, Green J, Pollard J, Jr., Tugendreich S. Causal analysis approaches in Ingenuity Pathway Analysis. Bioinformatics. 2014;30(4):523-30.
16. Kim D, Kobayashi T, Voisin B, Jo JH, Sakamoto K, Jin SP, et al. Targeted therapy guided by single-cell transcriptomic analysis in drug-induced hypersensitivity syndrome: a case report. Nat Med. 2020;26(2):236-43.
17. Sole-Boldo L, Raddatz G, Schutz S, Mallm JP, Rippe K, Lonsdorf AS, et al. Single-cell transcriptomes of the human skin reveal age-related loss of fibroblast priming. Commun Biol. 2020;3(1):188.
18. Tabib T, Huang M, Morse N, Papazoglou A, Behera R, Jia M, et al. Myofibroblast transcriptome indicates SFRP2(hi) fibroblast progenitors in systemic sclerosis skin. Nature communications. 2021;12(1):4384.

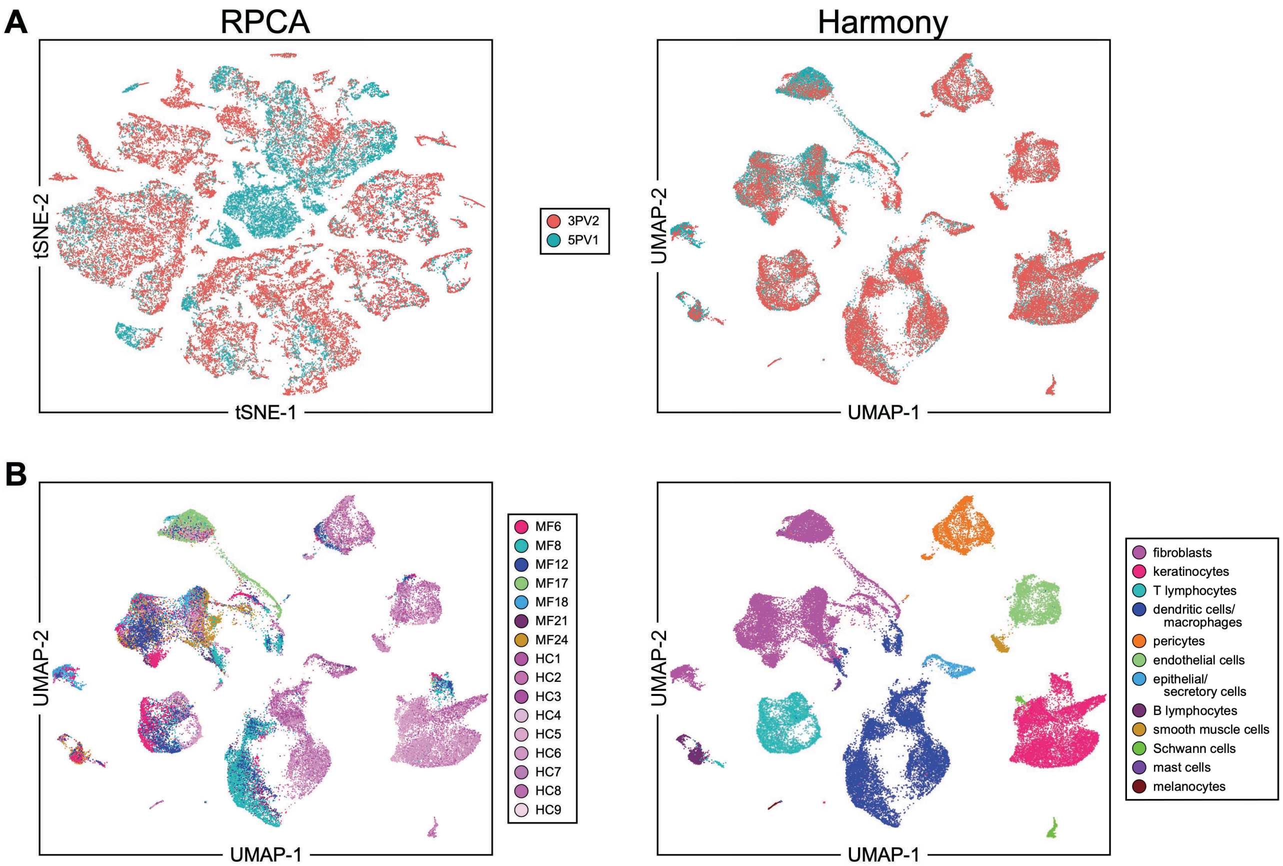

<u>Figure S1.</u> Batch correlation analysis comparison by Harmony (13). **(A)** Transcriptomes of 51,468 cells including all cell-types from nine healthy controls (HC) (24,322 cells) and seven advanced MF (27,146 cells) skin biopsies clustered using Reciprocal PCA (RPCA) and Harmony, respectively. Chemistry differences were corrected for in two-dimensional t-SNE and UMAP, respectively. **(B)** UMAP of all samples IDs to verify clustering exhibits no batch effects as no individual samples create own clusters (left panel)**.** UMAP of all cell types using Harmony to verify there are no batch effects as all samples overlap per cell type in this UMAP as we have shown in Seurat's clustering (left panel). UMAP shows similar clustering as Seurat in separating out unique cell types (right panel).

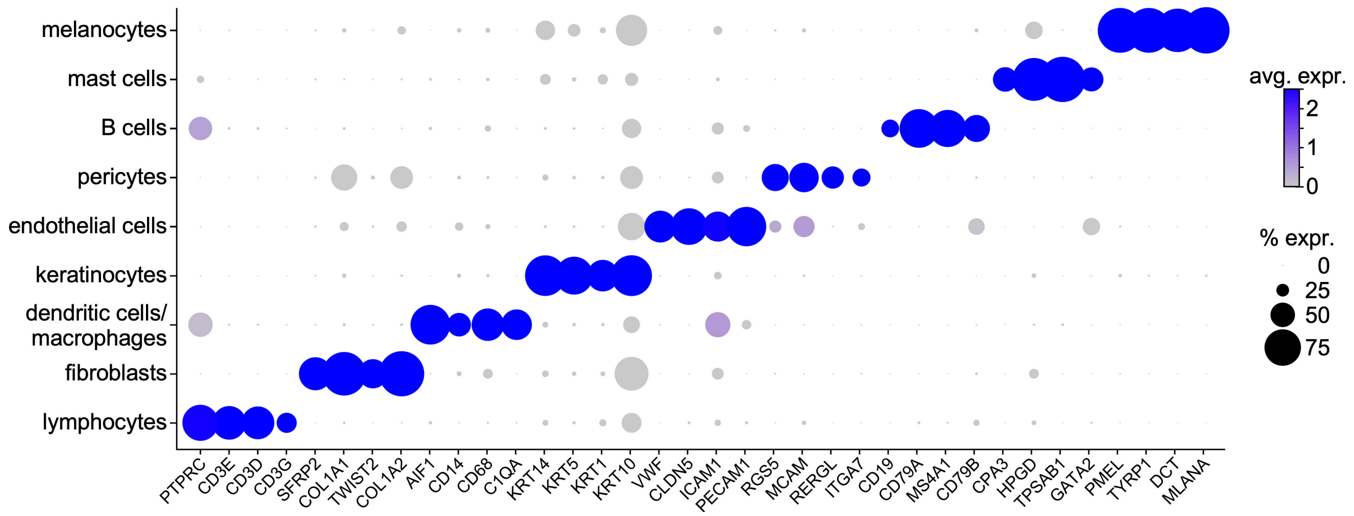

Figure S2. Dot-plot showing the proportion of cells and the scaled average gene expression of a panel of known markers for multiple cell types (1, 16-18) from all MF and HC sample combined (Figure 1, main text). Gene differential tests are described in the Methods. Briefly, we used the “FindAllMarkers” function of Seurat, which uses the Wilcoxon rank sum test to show differential genes with a minimum percentage of cells of 25% per cluster, log-fold change >0.25, and p-value < 0.05 for significance (9, 10).

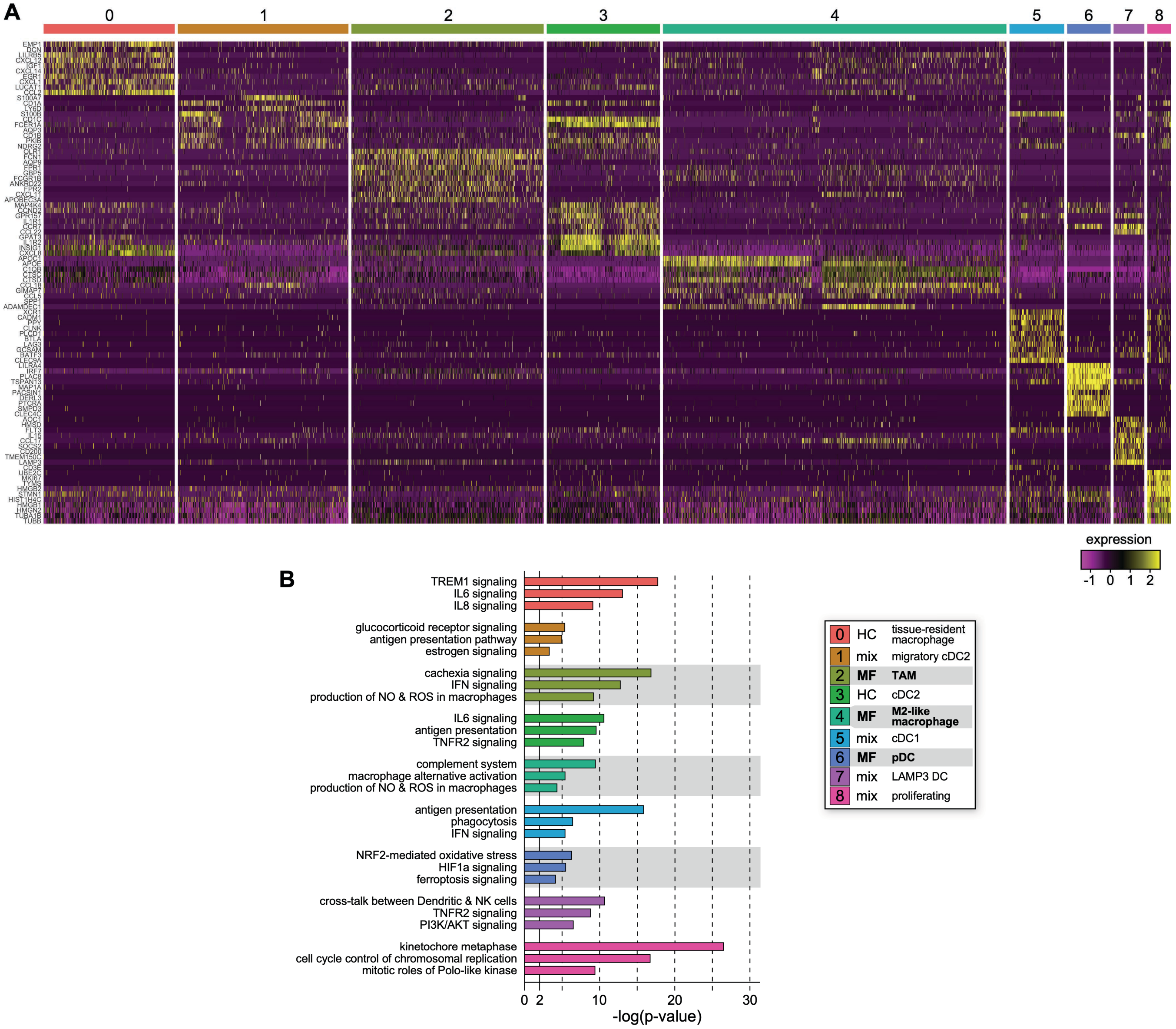

Figure S3. Myeloid-cell transcriptome in the cutaneous microenvironment of HC and MF samples. **(A)** Transcriptomes of 4425 AIF1^{+} cells (1,730 from HC and 3,295 from MF skin samples) revealed 9 discrete Louvain clusters using Seurat (7, 8) (see main text Figure 2). Heatmap showing a selection of the most highly significant DE genes in each of these clusters (n=10). Cluster numbers are indicated at the top. Each column represents a cell. **(B)** The differential gene lists were filtered by p-value <0.05 for significance and then run in Ingenuity Pathway Analysis (IPA, Qiagen) (15) for significant up-regulated pathways. Highly significant examples of distinct pathways activated by individual clusters are shown. Pathways are represented by enrichment scores (-log p-values) and selected by absolute z-scores over 2 (15).

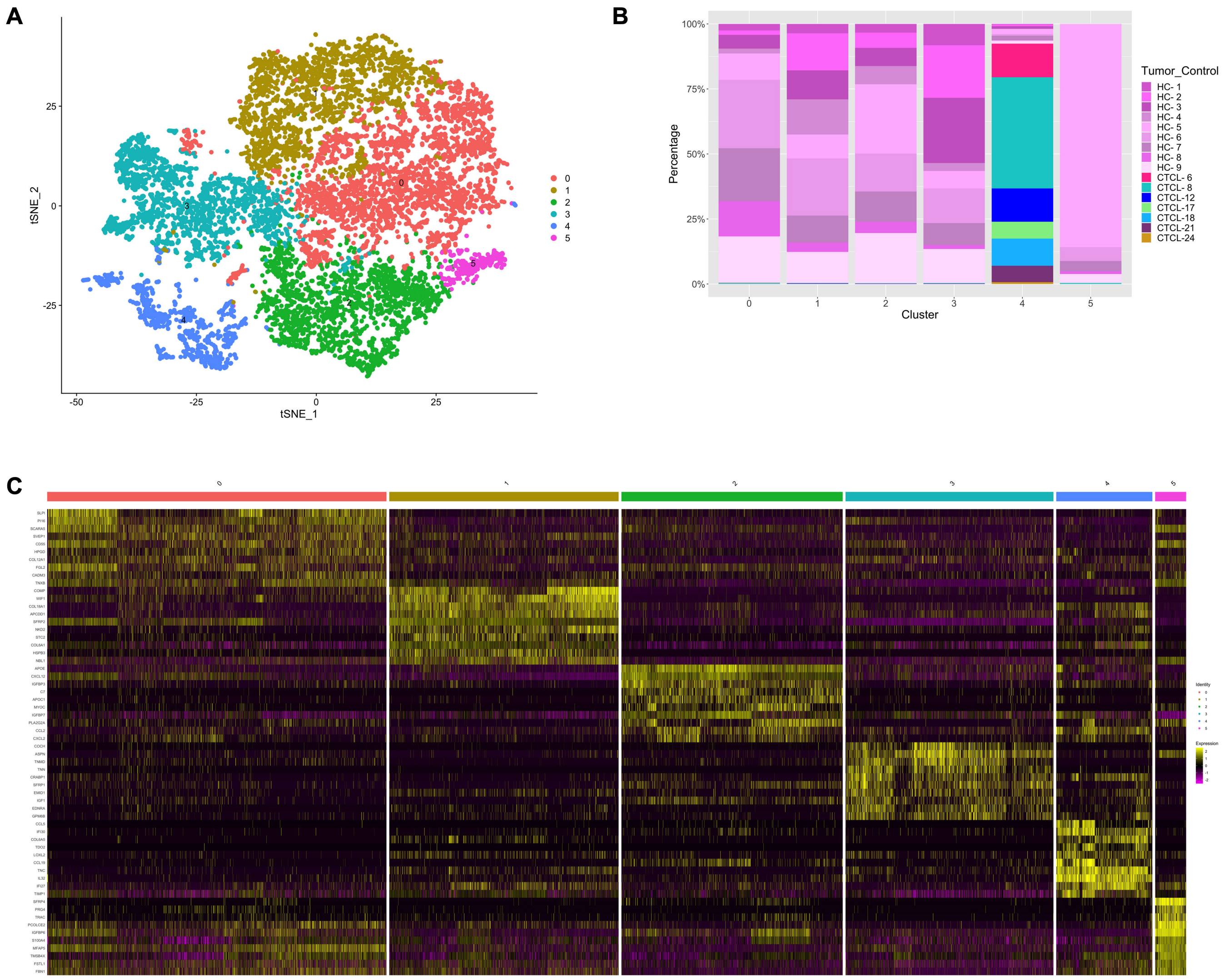

<u>Figure S4.</u> (A) Transcriptomes of 9,533 COL1A1^{+} cells (8,748 from HC and 785 from MF samples) revealed 6 discrete Louvain clusters using Seurat (18, 19). **(B)** Bar plot showing the proportion of cells from each MF or HC sample within individual clusters. **(C)** Heatmap showing examples of the most highly significant differentially expressed genes (n=10) for each cluster from (A). Cluster numbers are indicated at the top. Each column represents a cell. Differential tests were performed as described in Material and Methods.

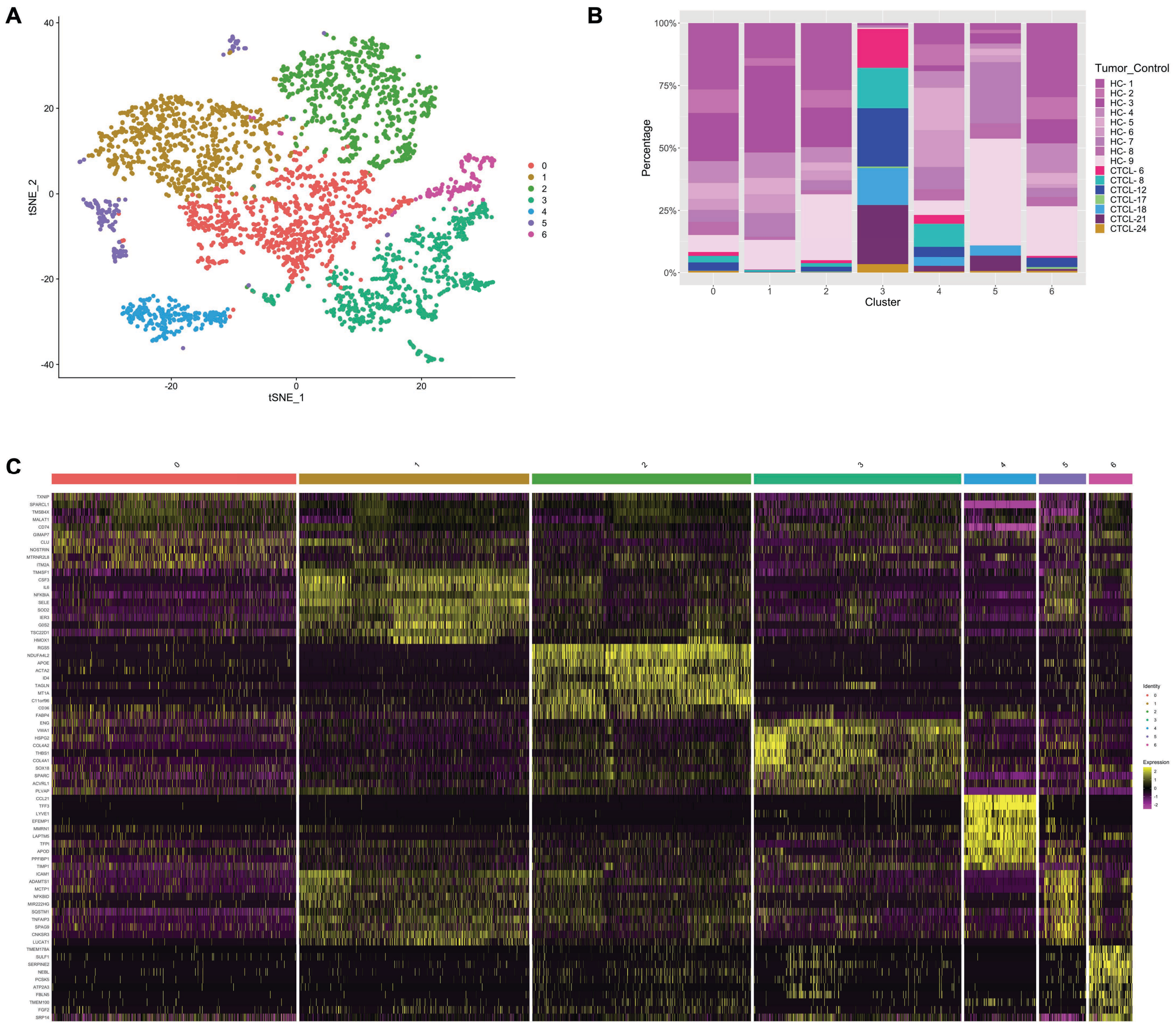

Figure S5. **(A)** Transcriptomes of 3,319 VWF^{+} cells (2,503 from HC and 816 from MF samples) revealed 7 discrete Louvain clusters using Seurat (18, 19). **(B)** Bar plot showing the proportion of cells from each MF or HC sample within individual clusters. **(C)** Heatmap showing examples of the most highly significant differentially expressed genes (n=10) for each cluster from (A). Cluster numbers are indicated at the top. Each column represents a cell. Differential tests were performed as described in Material and Methods.

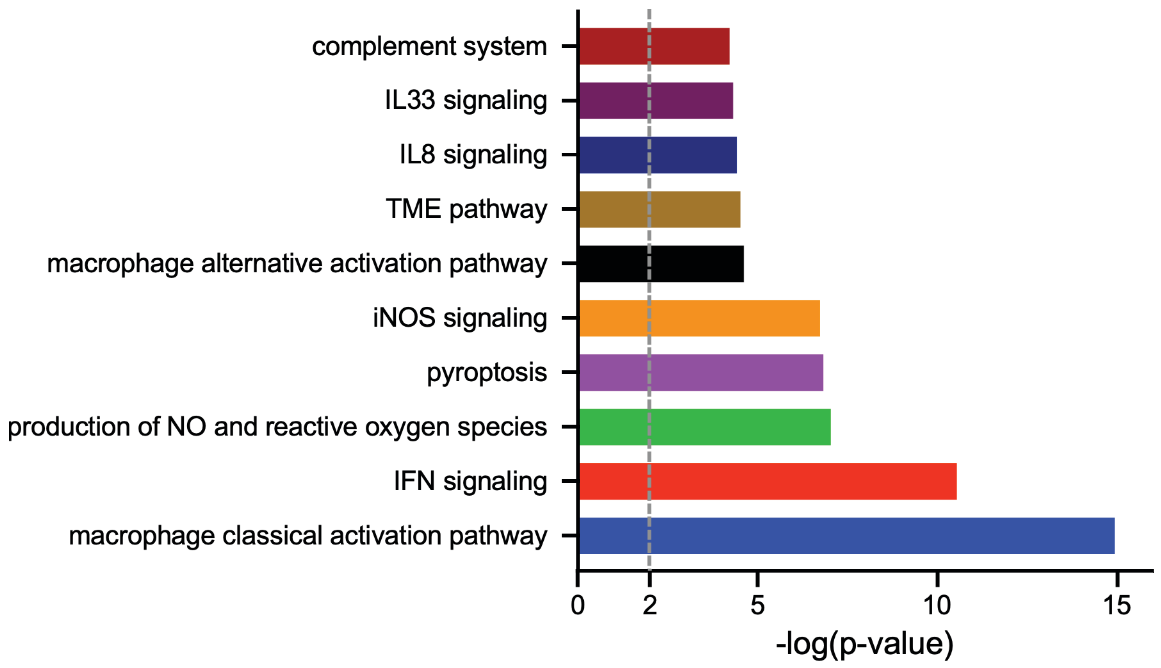

<u>Figure S6.</u> Highly significant examples of upregulated pathways by MF versus AD and PS myeloid cells are shown (Ingenuity Pathway Analysis, Qiagen) (15). Pathways are represented by enrichment scores (-log p-values) and selected by absolute z-scores over 2.

Table S1. Demographic and clinical features of patients.

Patient ID Age/Sex	Presence of Nodal Disease (Y/N with full description)	Stage*	TBSA (%patch, %plaque, %tumor)*	Year of Diagnosis/ Study Biopsy Date	Treatment prior to biopsy	Treatment at time of biopsy
**MF 17 76/F**	PET-CTs in 3/2019 and 4/2019 showed multiple new areas of FDG avid lymphadenopathy and FDG avid skin thickening relative to PET-CT from 2016.	MF IIB T3NxM0B0 *Positive FDG avid nodes with no biopsy suspicious for lymph nodal involvement	TBSA 11.6% (6.35% tumor, 3.75% plaque, 1.5% patch), mSWAT 34.4	Diag: 2016 Biopsy:12/2018	• Targretin: 2/2017 - 11/2018<br>• Radiation: 7/2017 - 8/2017 involving the lower arms and legs, face and posterior scalp.<br>• Valchlor: 7/2018 - 12/2018	None
**MF18 87/M**	PET-CT 2/27/2019: "No regional lymphadenopathy involvement that is concerning for metastatic disease."	MF IIB T3N0M0B0 (CD30- large cell transformation	13.45% TBSA (11.1% patch, 1.75% plaque, 0.6% tumor), mSWAT 17	Diag: 2019 Biopsy: 2/2019	None	None
**MF21 61/M**	PET-CT 5/22/2020: "Stable small bilateral axillary nodes and stable small bilateral inguinal nodes, one of which demonstrates slightly increased uptake"	MF IIB T3NxM0B0 *Positive FDG avid nodes with no biopsy suspicious for lymph nodal involvement	15% TBSA (2% plaque, 13% tumors), 64.0 mSWAT	Diag: 2017 Biopsy:11/2020	• PUVA: 8/2017 - 5/2018<br>• Bexarotene: 8/2017 - 5/2018<br>• CD30/CD16A bispecific antibody on trial (AAAP4461), from 6/2018 - 8/2018<br>• Brentuximab: 8/2018 - 12/2018;<br>• TTI62101 (antiphagocytic CD47 binding protein): 12/2018 - 4/2019;<br>• Pembrolizumab/pralatrexate/decitabine: 4/2019 - 6/2020<br>• Mogalizumab: 7/2020 - 11/2020<br>• Radiation: 10/2020 - 11/2020	Mogalizumab
**MF24 42/M**	No evidence of lymph nodes on clinical exam. PET/CT not performed.	MF IIB T3NxM0B0 (CD30- large cell transformation)	2.8% (1.3% tumor, 1.5% plaque)	Diag: 2001 Biopsy: 3/2021	• PUVA (2001-2003)<br>• Bexarotene (2004-2006)<br>• Gemcitabine (2006-2008),<br>• Vorinostat (2008/2009-2011)<br>• Double cord stem cell transplant (5/17/12) after non-MAC with Cy/Flu/TBI200<br>• Radiation (November 2012).<br>• Radiation (2017)<br>• Bexarotene (10/2018 – time of biopsy)	Bexarotone
**MF8 46/F**	PET/CT 11/2016 showed no evidence of FDG avid lymph nodes.<br>No evidence of lymphadenopathy or visceral disease.	MF IIB T3N0M0B0	30% (29% patch/plaque, 1% tumor)	Diag: 2005 Biopsy:11/2017	2006-2016:<br>• NBUVB<br>• PUVA<br>• Vorinostat<br>• methotrexate<br>• IFN alpha<br>• Nitrogen mustard gel<br>• Bexarotene<br>• Pralatrexate	None
**MF12 77/M**	Low grade FDG uptake in the skin. Bilateral FDG avid cervical lymph nodes	MF, IIIA T4NxM0B0	100% (94.5% patch, 4.5% plaque, 1% tumor)	Diag: 2016 Biopsy: 4/2018	• Valchlor: 4/2016 - 12/2017<br>• Romidepsin: 5/2018<br>• Bexarotene: 6/2016 - 12/2017	None

**MF6 83/F**	No evidence of lymph nodes on clinical exam. PET/CT not performed.	MF IVB T3NxM0B2	37.5% (2% patch, 35% plaque 0. 5% tumor)	Diag: 2016 Biopsy:11/2017	• Nitrogen mustard<br>• Bexarotene<br>• Pralatrexate<br>• LEB	Bexarotene
**MF2 71/M**	PET/CT 12/2018 showed no pathologically enlarged lymph nodes	MF IIB with PC-ALCL	4.25% TBSA (.25% papules, 4.5% plaques)	Diag: 2018 Biopsy: 8/2018	• None	None
**MF5 63/F**	No evidence of lymph nodes on clinical exam. PET/CT not performed.	MF IIB T3NxM0B0	2% (1.5% patch, 0.25% tumor)	Diag: 2016 Biopsy: 3/2018	• Mechlorethamine gel<br>• ILTAC 10	Mechlorethamine gel
**MF4 72/F**	No evidence of lymph nodes on clinical exam. PET/CT not performed.	MF, IVA T3N1xB0M0	11.6% (1.5% patch, 3.75% plaque, 6.35% tumor)	Diag: 2016 Biopsy: 1/2018	• Topical steroids<br>• brentuximab	None

Abbreviations:

* Reported data is from the closest time point to the study biopsy date.

TBSA (total body surface area) at last follow-up visit with % patch, % plaque, and % tumor.

NBUVB: Narrowband UVB phototherapy

PUVA: psoralen long-wave ultraviolet radiation

IFN: Interferon

TTI-621 (Trillium) clinical trial: NCT02663518 Phase I trial of TTI-621 for patients with hematologic malignancies and selected solid tumors; NCT02890368: Phase I trial of intratumoral injections of TTI-621 in subjects with relapsed and refractory solid tumors and mycosis fungoides

Table S2. Histology features of samples used in scRNA-seq and imaged in Figure 1A.

Patient No.	Histology and Immunohistochemistry
**MF17**	The patient presented with a prior history of tumor stage MF. A prior peripheral blood flow analysis (December/2016) showed marked eosinophilia and a clonal TCR population. The patient has completed several courses of antibiotics. Several anatomical punch biopsies were taken for pathological and TCR rearrangement analyses, including soft tissue, right arm, right buttock, core (January-April 2019). The skin architecture presents parakeratosis, spongiosis as well as foci with lymphocytes in the epidermis and in the dermis admixed with eosinophils. Lymphocytes show loss of CD5 and CD7, skewed CD4 to CD8 ratio (greater than 5:1 approaching 10:1 range). CD30 expression in large malignant cells. The actual percent of CD30 positive staining is not more than 20% but overall a significant extent of the large cell infiltrate does express CD30. The lymphocytic infiltrate is positive for clonal TCR-beta gene rearrangement, consistent with a clonal lymphoid population in the sample. Pas stain fails to reveal fungal hyphae or basement membrane thickening. Pathological examination was consistent with tumor stage granulomatous MF with large cell transformation and CD30 positivity.
**MF18**	The patient presented with an extensive patch and plaque-like eruption with a large tumor that has biopsied. This punch biopsy shows an extensive tumefactive lymphomatous process involving the entire sampled thickness of the dermis. In addition, there is focal infiltration of the epidermis by atypical lymphocytes, whereby the epidermal response is a varied one, ranging from attenuation to hyperplasia. The dermal infiltrate is highly atypical. There is infiltration of the adventitial dermis of the eccrine coil by malignant lymphocytes. While there are some smaller lymphocytes in the background, the prevailing lymphoid populace is intermediate to larger sized lymphocytes many of which have a large, transformed morphology, more open chromatin, and conspicuous basophilic nucleoli. A positive TCR was detected on flow cytometry. Multiple atypical lymphocytes stained positively for Beta F1. We detected an extensive immunoreactivity of the infiltrate for CD2, CD3, and CD7, but loss of CD5. CD4 and CD8 demonstrate reactive CD8 T cells in the background with a number of the neoplastic cellular elements expressing CD4 as well as lymphocytes with a double negative CD4 and CD8 phenotype. There is focal staining for granzyme. Multiple neoplastic cells show granzyme positivity noted amidst the neoplastic cells in the superficial dermis. A similar pattern is observed for TIA. The malignant lymphocytes also show immunoreactivity for CD25; the staining is cytoplasmic and relatively deep but a number of the cells show some staining likely in the realm of 50%. A significant number of lymphocytes are negative for Foxp3, CD56, CD57, or PD-1, EBER, CD30. Pathology was consistent with advanced tumor stage mycosis fungoides with large cell transformation.
**MF21**	The patient presented with a history of mycosis fungoides with reported CD30+ large cell transformation and lymph node involvement. There is an extensive pan-dermal highly atypical lymphocytic infiltrate accompanied by many histiocytes including multinucleated giant cells. The dominant localization of the infiltrate is dermal, closely apposed to the epidermis with focal areas of epidermotropism. In these focal areas, the lymphocytes are a mixture of small, intermediate, and very atypical larger lymphoid forms. The infiltrate within the dermis is characterized by highly atypical large lymphocytes (15-20 micron) manifesting complex nuclear contours with conspicuous eosinophilic nucleolation. This highly atypical lymphoid population exhibits a coalescing multinodular and sheet-like growth throughout the dermis. There is a palisade of histiocytes around areas of fibrinoid necrobiotic change whereby the epicenter is a damaged vessel showing luminal

	and mural fibrin deposition with attendant red cell extravasation. Phenotypic analysis of the lymphocytes indicate that they are alpha-beta subset as revealed by the degree of immunoreactivity for beta F1. The T-cell nature of this malignant process is highlighted by the positive CD2, CD3, and CD5 stains. The CD7 stain is minimally positive; similarly, the TIA stain is negative except for a few reactive CD8-positive cells that show TIA positivity. Granzyme is positive in small reactive CD8-positive T cells. There are many malignant cells that show a very strong nuclear signal for TOX (~ 20%). There is an extensive degree of immunoreactivity for ICOS corresponding with the large cell activated morphology that prevails in this tumor. In contradistinction, the PD-1 stain is minimally positive. The Ki-67 proliferation index in focal areas is in the 30-40% realm with an overall percentage in the 20-30% realm. Many of the malignant cells are highlighted by CD4, while the CD8 preparation shows only a few reactive CD8-positive T cells. The CD4-CD8 ratio is in excess of 10:1. There are a number of malignant lymphocytes, however, that are double-negative. Most of the tumor is negative for CD30 expression. The infiltrate is largely localized within the dermis with minimal epidermotropism. The malignant cells have a highly atypical morphology with 20-30 micron size range. The findings are consistent with tumor stage mycosis fungoides with extensive pan-dermal large cell malignant lymphoid infiltrate.
**MF24**	This patient presented with a history of tumor stage mycosis fungoides with concern for large cell transformation. The biopsy is significant for a nodular infiltrate of atypical lymphocytes that have open chromatin and frequent single-cell necrosis. Mitoses are also seen. The cells are large in size. Large cells occupy more than 80% of the atypical lymphocytic population. The lymphocytes are composed of CD3 positive T cells. Interestingly, the T cells actually appear to have partial loss of CD3 expression. The lymphocytes are diffusely immunopositive for CD5, and when CD3 is compared to CD5, there is at least 50% loss. The lymphocytes also show similar loss of CD2. The atypical lymphocytes show diffuse PD-1 expression, and they also show nuclear positivity for TOX. Ki-67 shows proliferation in the range of over 90%. A CD7 immunostain shows reduction in the range of 60%. The T cells are dominated by CD4 positive cells with an over 6:1 CD4-CD8 ratio. The T cells are also immunopositive for beta F1. A CD30 is only positive in less than 5% of the cells. The findings are consistent with tumor stage mycosis fungoides with CD30 negative large cell transformation.
**MF6**	An atypical lymphoid infiltrate with CD2, CD3, CD4 and CD5 immunostains mark the majority of the cells within the infiltrate. CD7 immunostain appears slightly down regulated. CD8 immunostain marks one third of cells within the infiltrate. CD79a and CD20 immunostain mark a very small minority of the cells in the infiltrate. There is a significant eccrinotropic and vasocentric component with evidence of vascular injury. The neoplastic lymphocytes are noncerebriform and are predominated by small and intermediate sized lymphocytes with a minor large cell component (<30%) without evidence of CD30 and ALK1 positivity. CD56 immunostain is mostly negative. TCR gamma immunostain is negative. EBER is negative. T-cell receptor-beta gene rearrangement is positive for clonal TCR-beta gene rearrangement, consistent with a clonal lymphoid population in the sample. This represents a T-cell lymphoproliferative disorder/lymphoma.
**MF8**	The patient had tumor stage mycosis fungoides with concern for large cell transformation. The biopsy shows a very striking and exaggerated pattern of psoriasiform epidermal hyperplasia. The findings are diagnostic of a fully evolved plaque stage lesion of mycosis fungoides. There is a striking pattern of follicular involvement captured on A1 whereby there is infiltration of the hair follicle and adventitial dermis by neoplastic lymphocytes. There are a number of neoplastic larger lymphoid elements within the epidermis and around blood vessels although insufficient to warrant categorization as large cell transformation since these larger cellular elements are intimately admixed with smaller lymphocytes (<25%). Nevertheless, they are discernible and in certain perivascular aggregates they are more numerous corresponding to

	focal areas where the perivascular Ki-67 proliferation index is quite high (> 50%). Overall, the findings are compatible with plaque stage mycosis fungoides with follicular involvement and a subtle interstitial granulomatous component with supervening pustular and eczematous features. Phenotypic analysis indicates that the lymphocytes are primarily alpha-beta (beta-F1^{+}). They are also extensively highlighted by CD3 and CD5 positivity, but with a significant decrease for CD2 and CD7 of approximately 80%. There is a strong nuclear signal for TOX amidst the neoplastic lymphocytes. The NFAT preparation shows a cytoplasmic staining pattern without significant nuclear staining. The proliferation index is variable. In some of the nodular lymphocytic foci (~50%). The CD30 stain highlights a few of the lymphocytes, mostly in the epidermis (<10%). In examining the CD4 and CD8 preparations it is apparent that the neoplastic cells are clearly of the CD4 subset, with the CD4 to CD8 ratio is in excess of 5:1. A diagnosis was rendered of mycosis fungoides with follicular involvement and early granulomatous features.
**MF12**	The patient presented with concern for tumor stage mycosis fungoides. The findings are those of an atypical epidermotropic lymphocytic infiltrate accompanied by a very extensive deeper seated tumefactive atypical lymphocytic infiltrate with a striking degree of granulomatous inflammation. Many of the lymphocytes have a cerebriform appearance and phenotypically the infiltrate is very abnormal showing a loss of CD2, CD5, beta-F1 and no staining for CD4 or CD8. Overall, the findings are compatible with an advanced tumor stage lesion of granulomatous mycosis fungoides. A significant part of the infiltrate in fact is histiocytic and is composed by smaller to intermediate size neoplastic T cells. There are scattered larger atypical cells of which a subset exhibit positivity for CD30 with an increased proliferation index in the 20-30% realm suggesting impending large cell transformation although not fulfilling the unequivocal criteria for large cell transformation. A diagnosis was rendered of tumor stage granulomatous mycosis fungoides with impending large cell transformation with focal CD30 positivity.

Table S3. Differentially expressed genes in keratinocytes that are common among the MF patient samples.

	MF6					MF8					MF12					MF17				
	p val	avg logFC	pct.1	pct.2	p val adj	p val	avg logFC	pct.1	pct.2	p val adj	p_val	avg logFC	pct.1	pct.2	p val adj	p_val	avg logFC	pct.1	pct.2	p val adj
ACP5	1.05E-51	0.278	0.293	0.056	3.38E-47	3.67E-292	0.470	0.339	0.056	1.18E-287	5.64E-62	0.238	0.264	0.056	1.82E-57	3.11E-23	0.247	0.419	0.056	1.00E-18
AGTRAP	3.38E-83	0.455	0.506	0.106	1.09E-78	0.00E+00	0.546	0.520	0.106	0.00E+00	7.84E-94	0.430	0.441	0.106	2.52E-89	8.48E-37	0.679	0.698	0.106	2.73E-32
AKR1B10	6.65E-46	0.423	0.239	0.043	2.14E-41	0.00E+00	1.919	0.688	0.043	0.00E+00	2.87E-298	1.318	0.507	0.043	9.24E-294	5.23E-64	1.648	0.581	0.043	1.69E-59
ALDH16A1	6.71E-18	0.109	0.297	0.111	2.16E-13	2.16E-188	0.271	0.367	0.111	6.96E-184	5.80E-52	0.330	0.360	0.111	1.87E-47	3.63E-16	0.159	0.535	0.111	1.17E-11
ALDH3B2	2.38E-16	0.161	0.328	0.139	7.68E-12	1.99E-226	0.536	0.430	0.139	6.41E-222	4.86E-79	0.652	0.468	0.139	1.57E-74	6.82E-09	1.146	0.442	0.139	2.20E-04
ALG3	4.35E-22	0.103	0.367	0.134	1.40E-17	2.82E-229	0.340	0.432	0.134	9.09E-225	4.06E-36	0.208	0.358	0.134	1.31E-31	2.91E-16	0.542	0.581	0.134	9.37E-12
ANGPTL4	4.22E-77	0.719	0.548	0.128	1.36E-72	0.00E+00	0.860	0.590	0.128	0.00E+00	7.60E-134	0.959	0.561	0.128	2.45E-129	9.56E-15	0.718	0.535	0.128	3.08E-10
ANKRD29	2.46E-38	0.162	0.178	0.028	7.94E-34	3.00E-117	0.157	0.158	0.028	9.65E-113	3.87E-86	0.279	0.221	0.028	1.25E-81	4.82E-79	0.463	0.535	0.028	1.55E-74
AP1S1	6.54E-13	0.142	0.452	0.236	2.11E-08	2.09E-78	0.163	0.434	0.236	6.74E-74	2.02E-51	0.389	0.550	0.236	6.51E-47	1.32E-12	0.683	0.721	0.236	4.25E-08
AP2S1	8.56E-24	0.532	0.730	0.475	2.76E-19	0.00E+00	0.754	0.778	0.475	0.00E+00	1.26E-70	0.794	0.782	0.475	4.06E-66	3.08E-09	0.749	0.860	0.475	9.92E-05
APEX2	5.04E-26	0.134	0.232	0.060	1.62E-21	6.10E-165	0.195	0.259	0.060	1.96E-160	4.10E-29	0.129	0.203	0.060	1.32E-24	1.49E-07	0.120	0.256	0.060	4.79E-03
ARHGDIA	2.48E-21	0.488	0.618	0.365	7.99E-17	4.15E-179	0.404	0.651	0.365	1.34E-174	1.90E-34	0.349	0.637	0.365	6.12E-30	3.67E-10	0.550	0.814	0.365	1.18E-05
ARHGDIB	5.14E-49	0.429	0.521	0.159	1.66E-44	4.43E-272	0.468	0.497	0.159	1.43E-267	8.92E-49	0.321	0.441	0.159	2.87E-44	8.59E-11	0.434	0.558	0.159	2.77E-06
ARL2	3.15E-22	0.186	0.510	0.227	1.02E-17	6.83E-256	0.472	0.567	0.227	2.20E-251	3.61E-41	0.338	0.502	0.227	1.16E-36	7.22E-10	0.285	0.651	0.227	2.33E-05
ARPC1B	1.10E-110	1.198	0.649	0.147	3.55E-106	0.00E+00	0.861	0.558	0.147	0.00E+00	3.18E-85	0.763	0.500	0.147	1.02E-80	1.00E-36	1.350	0.791	0.147	3.24E-32
ARPC3	2.13E-28	0.501	0.811	0.579	6.85E-24	0.00E+00	0.858	0.837	0.579	0.00E+00	1.92E-38	0.479	0.815	0.579	6.18E-34	6.43E-08	0.705	0.884	0.579	2.07E-03
ARPC4	3.12E-38	0.445	0.575	0.239	1.01E-33	0.00E+00	0.784	0.660	0.239	0.00E+00	3.11E-59	0.440	0.574	0.239	1.00E-54	3.94E-14	0.536	0.744	0.239	1.27E-09
ATOX1	1.52E-72	1.111	0.730	0.296	4.90E-68	2.16E-262	0.528	0.651	0.296	6.95E-258	2.42E-120	1.018	0.748	0.296	7.79E-116	2.12E-09	0.416	0.744	0.296	6.82E-05
ATP5F1D	3.28E-11	0.315	0.780	0.637	1.06E-06	0.00E+00	0.735	0.859	0.637	0.00E+00	4.49E-78	0.890	0.881	0.637	1.45E-73	4.58E-05	0.414	0.837	0.637	1.00E+00
ATP5PD	7.28E-19	0.437	0.776	0.577	2.35E-14	5.79E-292	0.707	0.820	0.577	1.86E-287	4.60E-47	0.544	0.822	0.577	1.48E-42	6.32E-05	0.348	0.814	0.577	1.00E+00
BANF1	1.48E-12	0.203	0.633	0.399	4.77E-08	2.38E-78	0.181	0.621	0.399	7.66E-74	3.74E-33	0.406	0.678	0.399	1.20E-28	2.19E-05	0.351	0.744	0.399	7.06E-01
BSG	3.33E-15	0.339	0.707	0.492	1.07E-10	3.33E-172	0.494	0.745	0.492	1.07E-167	3.68E-31	0.414	0.730	0.492	1.19E-26	2.67E-08	0.776	0.814	0.492	8.58E-04
C10orf99	7.33E-65	0.938	0.255	0.035	2.36E-60	0.00E+00	2.043	0.525	0.035	0.00E+00	1.19E-190	1.351	0.369	0.035	3.83E-186	1.06E-23	0.997	0.326	0.035	3.42E-19
C1R	1.63E-249	1.553	0.656	0.063	5.26E-245	0.00E+00	1.232	0.607	0.063	0.00E+00	2.55E-206	1.142	0.493	0.063	8.21E-202	2.57E-70	0.923	0.744	0.063	8.29E-66
CALML3	2.42E-20	0.717	0.579	0.328	7.79E-16	1.18E-203	0.920	0.637	0.328	3.80E-199	6.00E-114	1.657	0.777	0.328	1.93E-109	9.45E-14	2.035	0.767	0.328	3.04E-09
CAPG	1.33E-12	0.257	0.683	0.453	4.28E-08	3.13E-143	0.390	0.724	0.453	1.01E-138	4.46E-48	0.589	0.750	0.453	1.44E-43	2.08E-09	0.720	0.837	0.453	6.71E-05
CAPN1	1.79E-32	0.553	0.687	0.386	5.78E-28	2.18E-278	0.625	0.722	0.386	7.01E-274	4.88E-37	0.398	0.667	0.386	1.57E-32	5.31E-07	0.429	0.767	0.386	1.71E-02
CAPNS1	1.19E-79	0.449	0.459	0.091	3.82E-75	1.65E-251	0.310	0.381	0.091	5.32E-247	1.90E-80	0.428	0.378	0.091	6.10E-76	4.89E-27	0.378	0.581	0.091	1.57E-22
CARHSP1	9.20E-92	0.874	0.687	0.200	2.96E-87	0.00E+00	1.250	0.710	0.200	0.00E+00	8.61E-114	0.941	0.624	0.200	2.77E-109	3.42E-19	0.662	0.744	0.200	1.10E-14
CBLC	2.68E-10	0.145	0.332	0.168	8.63E-06	6.30E-122	0.201	0.396	0.168	2.03E-117	1.12E-37	0.247	0.417	0.168	3.61E-33	1.43E-11	0.231	0.581	0.168	4.61E-07
CCDC124	5.69E-19	0.280	0.521	0.263	1.83E-14	1.70E-180	0.337	0.559	0.263	5.48E-176	7.59E-45	0.390	0.565	0.263	2.44E-40	2.13E-12	0.527	0.767	0.263	6.88E-08
CD47	1.31E-62	0.986	0.695	0.296	4.22E-58	0.00E+00	0.909	0.722	0.296	0.00E+00	1.50E-31	0.376	0.554	0.296	4.84E-27	1.55E-14	0.948	0.767	0.296	4.98E-10
CD74	0.00E+00	2.799	0.834	0.087	0.00E+00	0.00E+00	1.131	0.612	0.087	0.00E+00	0.00E+00	2.898	0.836	0.087	0.00E+00	4.57E-94	4.830	0.930	0.087	1.47E-89
CD82	4.37E-14	0.130	0.598	0.354	1.41E-09	3.67E-168	0.350	0.652	0.354	1.18E-163	2.47E-15	0.100	0.547	0.354	7.96E-11	1.06E-09	0.606	0.791	0.354	3.41E-05
CDC42EP1	9.32E-39	0.589	0.560	0.233	3.00E-34	1.10E-280	0.599	0.585	0.233	3.53E-276	4.40E-47	0.418	0.538	0.233	1.42E-42	9.60E-29	1.669	0.837	0.233	3.09E-24
CDH3	5.90E-76	0.647	0.587	0.149	1.90E-71	2.08E-288	0.562	0.498	0.149	6.69E-284	1.27E-53	0.411	0.435	0.149	4.08E-49	4.03E-30	1.258	0.744	0.149	1.30E-25
CIB1	8.56E-34	0.311	0.541	0.208	2.76E-29	5.91E-205	0.340	0.519	0.208	1.90E-200	1.09E-47	0.307	0.509	0.208	3.50E-43	2.94E-22	0.702	0.814	0.208	9.47E-18
CISD2	4.98E-27	0.246	0.498	0.206	1.60E-22	5.77E-116	0.209	0.441	0.206	1.86E-111	7.31E-13	0.195	0.358	0.206	2.36E-08	3.61E-08	0.109	0.605	0.206	1.16E-03
CISD3	8.20E-21	0.191	0.336	0.127	2.64E-16	6.96E-235	0.318	0.423	0.127	2.24E-230	1.63E-56	0.296	0.403	0.127	5.25E-52	1.61E-18	0.618	0.605	0.127	5.17E-14
CLCA2	8.41E-18	0.210	0.568	0.297	2.71E-13	0.00E+00	0.662	0.700	0.297	0.00E+00	7.87E-96	0.983	0.712	0.297	2.54E-91	1.29E-19	1.390	0.837	0.297	4.16E-15
CLEC7A	3.98E-271	0.454	0.390	0.013	1.28E-266	0.00E+00	0.316	0.283	0.013	0.00E+00	2.29E-227	0.391	0.297	0.013	7.39E-223	1.79E-106	0.356	0.442	0.013	5.75E-102
CMAS	9.56E-24	0.147	0.409	0.157	3.08E-19	2.11E-118	0.179	0.374	0.157	6.80E-114	5.81E-21	0.110	0.338	0.157	1.87E-16	2.40E-10	0.158	0.558	0.157	7.72E-06
CNDP2	1.62E-30	0.185	0.344	0.103	5.20E-26	5.19E-169	0.206	0.340	0.103	1.67E-164	3.35E-44	0.207	0.331	0.103	1.08E-39	1.70E-20	0.292	0.558	0.103	5.47E-16
CNIH4	9.32E-15	0.243	0.556	0.324	3.00E-10	4.62E-135	0.301	0.590	0.324	1.49E-130	1.11E-15	0.170	0.525	0.324	3.58E-11	4.36E-06	0.224	0.698	0.324	1.40E-01
CNN2	1.02E-18	0.293	0.560	0.303	3.30E-14	2.95E-74	0.159	0.522	0.303	9.49E-70	8.83E-17	0.141	0.502	0.303	2.84E-12	2.59E-13	0.905	0.791	0.303	8.33E-09
CNP	1.26E-63	0.405	0.506	0.129	4.06E-59	4.67E-173	0.270	0.380	0.129	1.50E-168	1.54E-52	0.322	0.392	0.129	4.97E-48	8.65E-06	0.128	0.372	0.129	2.79E-01

COA3	2.86E-17	0.165	0.456	0.214	9.21E-13	7.00E-250	0.428	0.553	0.214	2.25E-245	1.52E-46	0.373	0.505	0.214	4.90E-42	3.68E-08	0.228	0.581	0.214	1.19E-03
COMTD1	3.76E-33	0.444	0.552	0.229	1.21E-28	5.93E-288	0.502	0.589	0.229	1.91E-283	9.42E-120	0.851	0.673	0.229	3.03E-115	9.61E-24	1.141	0.814	0.229	3.09E-19
COPE	3.89E-19	0.432	0.703	0.464	1.25E-14	1.08E-271	0.612	0.754	0.464	3.48E-267	1.09E-47	0.559	0.745	0.464	3.52E-43	8.48E-06	0.352	0.814	0.464	2.73E-01
COQ4	4.02E-13	0.156	0.494	0.265	1.30E-08	5.85E-124	0.215	0.519	0.265	1.89E-119	4.55E-31	0.256	0.523	0.265	1.47E-26	2.42E-13	0.995	0.767	0.265	7.79E-09
CORO1B	2.72E-30	0.290	0.479	0.183	8.74E-26	3.79E-116	0.206	0.408	0.183	1.22E-111	1.37E-37	0.292	0.435	0.183	4.43E-33	7.96E-16	0.519	0.674	0.183	2.56E-11
COX16	1.74E-12	0.207	0.606	0.361	5.60E-08	4.38E-125	0.263	0.622	0.361	1.41E-120	4.85E-46	0.401	0.676	0.361	1.56E-41	4.22E-06	0.180	0.744	0.361	1.36E-01
CST3	4.34E-32	0.677	0.815	0.491	1.40E-27	0.00E+00	2.694	0.914	0.491	0.00E+00	8.11E-120	1.690	0.869	0.491	2.61E-115	7.52E-08	0.734	0.837	0.491	2.42E-03
CTSA	5.18E-17	0.127	0.405	0.183	1.67E-12	5.19E-215	0.352	0.491	0.183	1.67E-210	3.13E-27	0.163	0.399	0.183	1.01E-22	2.21E-08	0.264	0.535	0.183	7.13E-04
CTSB	8.57E-41	0.427	0.637	0.259	2.76E-36	0.00E+00	0.675	0.682	0.259	0.00E+00	8.52E-65	0.622	0.610	0.259	2.75E-60	2.17E-22	1.627	0.791	0.259	6.99E-18
CTSC	6.21E-35	0.281	0.548	0.204	2.00E-30	0.00E+00	1.533	0.770	0.204	0.00E+00	5.36E-107	0.884	0.628	0.204	1.73E-102	5.49E-21	1.072	0.767	0.204	1.77E-16
CTSD	2.20E-22	0.473	0.695	0.424	7.08E-18	0.00E+00	0.906	0.792	0.424	0.00E+00	3.62E-80	0.998	0.773	0.424	1.16E-75	2.01E-15	1.498	0.837	0.424	6.48E-11
CYBC1	7.80E-46	0.166	0.286	0.057	2.51E-41	3.48E-244	0.283	0.313	0.057	1.12E-239	6.31E-92	0.331	0.322	0.057	2.03E-87	3.12E-38	0.336	0.535	0.057	1.01E-33
DBNL	1.42E-48	0.370	0.490	0.146	4.57E-44	0.00E+00	0.671	0.582	0.146	0.00E+00	2.24E-69	0.397	0.462	0.146	7.22E-65	6.19E-16	0.350	0.605	0.146	1.99E-11
DCTD	7.74E-34	0.260	0.405	0.129	2.49E-29	1.13E-164	0.238	0.376	0.129	3.64E-160	1.44E-21	0.153	0.295	0.129	4.65E-17	9.72E-13	0.271	0.512	0.129	3.13E-08
DNAJC15	1.04E-41	0.392	0.583	0.221	3.35E-37	6.57E-103	0.157	0.444	0.221	2.12E-98	2.20E-55	0.377	0.550	0.221	7.09E-51	2.33E-21	0.610	0.814	0.221	7.51E-17
EIF4EBP1	5.06E-26	0.208	0.344	0.118	1.63E-21	0.00E+00	0.684	0.540	0.118	0.00E+00	4.28E-63	0.502	0.394	0.118	1.38E-58	1.84E-15	0.540	0.512	0.118	5.93E-11
ELOVL1	3.88E-17	0.197	0.436	0.198	1.25E-12	4.12E-170	0.334	0.477	0.198	1.33E-165	1.71E-18	0.111	0.387	0.198	5.50E-14	3.10E-12	0.297	0.651	0.198	9.99E-08
ENO1	1.42E-33	0.916	0.876	0.689	4.57E-29	0.00E+00	1.388	0.909	0.689	0.00E+00	4.00E-42	0.732	0.865	0.689	1.29E-37	1.71E-11	1.085	0.907	0.689	5.49E-07
EPHX3	7.59E-29	0.329	0.394	0.142	2.45E-24	6.16E-259	0.607	0.455	0.142	1.98E-254	5.27E-20	0.218	0.302	0.142	1.70E-15	4.65E-06	0.472	0.395	0.142	1.50E-01
ETHE1	1.70E-109	0.881	0.591	0.122	5.47E-105	0.00E+00	0.862	0.618	0.122	0.00E+00	2.71E-85	0.456	0.462	0.122	8.74E-81	6.13E-37	0.704	0.767	0.122	1.98E-32
ETV7	1.01E-250	0.545	0.475	0.027	3.24E-246	7.67E-172	0.174	0.198	0.027	2.47E-167	3.65E-121	0.333	0.261	0.027	1.18E-116	4.57E-63	0.883	0.465	0.027	1.47E-58
FABP5	1.90E-15	1.822	0.676	0.449	6.13E-11	0.00E+00	2.361	0.786	0.449	0.00E+00	6.43E-98	2.272	0.815	0.449	2.07E-93	1.86E-19	3.834	0.930	0.449	6.00E-15
FADD	3.96E-17	0.105	0.166	0.046	1.27E-12	0.00E+00	0.326	0.343	0.046	0.00E+00	1.30E-59	0.183	0.239	0.046	4.20E-55	3.34E-19	0.172	0.349	0.046	1.08E-14
FAM207A	2.81E-25	0.168	0.313	0.100	9.05E-21	1.83E-259	0.276	0.403	0.100	5.90E-255	4.09E-53	0.244	0.345	0.100	1.32E-48	6.84E-21	0.247	0.558	0.100	2.20E-16
FAM83H	1.12E-09	0.100	0.266	0.129	3.61E-05	2.49E-136	0.357	0.345	0.129	8.01E-132	5.25E-33	0.208	0.342	0.129	1.69E-28	1.42E-08	0.135	0.442	0.129	4.56E-04
FARSA	9.84E-27	0.193	0.421	0.157	3.17E-22	2.06E-110	0.200	0.363	0.157	6.63E-106	9.68E-22	0.141	0.338	0.157	3.12E-17	5.21E-14	0.300	0.605	0.157	1.68E-09
FASTK	2.05E-19	0.135	0.413	0.174	6.61E-15	7.05E-236	0.337	0.497	0.174	2.27E-231	1.95E-23	0.143	0.372	0.174	6.27E-19	1.17E-15	0.324	0.674	0.174	3.77E-11
FKBP1A	1.88E-18	0.493	0.691	0.497	6.07E-14	5.37E-212	0.552	0.756	0.497	1.73E-207	7.90E-39	0.547	0.741	0.497	2.54E-34	4.36E-09	0.777	0.814	0.497	1.40E-04
FKBP8	1.31E-09	0.169	0.575	0.356	4.23E-05	7.13E-154	0.350	0.643	0.356	2.30E-149	2.93E-67	0.657	0.707	0.356	9.42E-63	5.71E-07	0.547	0.744	0.356	1.84E-02
GADD45GIP1	3.54E-25	0.494	0.691	0.421	1.14E-20	0.00E+00	0.758	0.744	0.421	0.00E+00	4.68E-74	0.828	0.755	0.421	1.51E-69	1.77E-06	0.330	0.791	0.421	5.70E-02
GAPDH	4.91E-13	0.533	0.950	0.872	1.58E-08	0.00E+00	0.803	0.971	0.872	0.00E+00	2.93E-21	0.425	0.968	0.872	9.42E-17	6.35E-18	1.418	1.000	0.872	2.04E-13
GAS2L1	3.06E-10	0.160	0.390	0.207	9.86E-06	7.86E-118	0.267	0.439	0.207	2.53E-113	3.08E-29	0.273	0.446	0.207	9.91E-25	7.30E-09	0.530	0.605	0.207	2.35E-04
GATB	3.66E-35	0.237	0.293	0.074	1.18E-30	0.00E+00	0.522	0.454	0.074	0.00E+00	5.51E-126	0.594	0.414	0.074	1.77E-121	8.02E-28	0.251	0.535	0.074	2.58E-23
GET3	3.35E-15	0.214	0.544	0.302	1.08E-10	1.22E-118	0.239	0.552	0.302	3.92E-114	2.13E-21	0.187	0.527	0.302	6.85E-17	3.85E-05	0.138	0.628	0.302	1.00E+00
GGCT	1.67E-25	0.515	0.606	0.320	5.36E-21	0.00E+00	0.730	0.697	0.320	0.00E+00	5.64E-48	0.523	0.637	0.320	1.82E-43	2.76E-08	0.425	0.744	0.320	8.89E-04
GJB2	2.63E-42	1.221	0.556	0.205	8.48E-38	0.00E+00	2.652	0.879	0.205	0.00E+00	3.79E-95	0.908	0.640	0.205	1.22E-90	6.90E-28	1.951	0.837	0.205	2.22E-23
GM2A	1.42E-26	0.377	0.463	0.190	4.59E-22	5.08E-139	0.325	0.439	0.190	1.64E-134	4.44E-27	0.222	0.410	0.190	1.43E-22	3.37E-21	0.731	0.744	0.190	1.08E-16
GNA15	3.06E-45	0.501	0.606	0.246	9.85E-41	0.00E+00	0.670	0.640	0.246	0.00E+00	1.52E-31	0.305	0.498	0.246	4.90E-27	6.24E-12	0.593	0.744	0.246	2.01E-07
GRN	1.70E-13	0.335	0.676	0.462	5.47E-09	2.89E-209	0.615	0.753	0.462	9.31E-205	4.89E-59	0.734	0.775	0.462	1.57E-54	1.07E-05	0.729	0.744	0.462	3.46E-01
GSTO1	1.47E-21	0.308	0.552	0.282	4.73E-17	3.24E-266	0.634	0.619	0.282	1.04E-261	3.05E-33	0.374	0.536	0.282	9.81E-29	8.11E-10	0.457	0.721	0.282	2.61E-05
GSTP1	1.94E-19	0.660	0.865	0.771	6.24E-15	0.00E+00	1.011	0.932	0.771	0.00E+00	2.18E-84	1.190	0.932	0.771	7.01E-80	1.18E-20	1.784	1.000	0.771	3.79E-16
GTF3C6	4.61E-35	0.405	0.595	0.247	1.48E-30	0.00E+00	0.925	0.716	0.247	0.00E+00	2.20E-68	0.583	0.597	0.247	7.09E-64	1.67E-09	0.526	0.674	0.247	5.37E-05
H2AFY	2.37E-17	0.289	0.676	0.400	7.64E-13	6.53E-308	0.649	0.738	0.400	2.10E-303	2.05E-47	0.481	0.705	0.400	6.61E-43	7.18E-08	0.840	0.767	0.400	2.31E-03
HCP5	9.63E-40	0.371	0.448	0.140	3.10E-35	8.83E-81	0.118	0.312	0.140	2.85E-76	1.21E-17	0.111	0.300	0.140	3.89E-13	3.71E-11	0.467	0.512	0.140	1.19E-06
HM13	2.26E-24	0.224	0.440	0.178	7.28E-20	2.74E-222	0.332	0.493	0.178	8.84E-218	1.28E-38	0.289	0.437	0.178	4.12E-34	4.03E-26	0.947	0.767	0.178	1.30E-21
HMGN1	9.58E-12	0.310	0.799	0.617	3.08E-07	6.28E-133	0.426	0.813	0.617	2.02E-128	1.81E-39	0.507	0.858	0.617	5.84E-35	1.62E-04	0.430	0.860	0.617	1.00E+00
HSBP1	5.03E-28	0.588	0.741	0.478	1.62E-23	1.05E-186	0.503	0.737	0.478	3.39E-182	4.23E-29	0.414	0.714	0.478	1.36E-24	5.09E-07	0.866	0.814	0.478	1.64E-02
IDH2	2.09E-32	0.432	0.598	0.279	6.73E-28	0.00E+00	0.619	0.664	0.279	0.00E+00	3.30E-59	0.515	0.608	0.279	1.06E-54	2.23E-13	0.691	0.767	0.279	7.20E-09
IFI27	0.00E+00	4.314	0.919	0.130	0.00E+00	0.00E+00	3.993	0.958	0.130	0.00E+00	0.00E+00	4.048	0.948	0.130	0.00E+00	4.39E-67	3.703	0.930	0.130	1.42E-62
IFI27L2	5.71E-99	1.091	0.749	0.240	1.84E-94	4.69E-271	0.510	0.603	0.240	1.51E-266	1.07E-133	1.013	0.721	0.240	3.45E-129	6.02E-17	0.745	0.767	0.240	1.94E-12
IFI6	0.00E+00	2.592	0.803	0.026	0.00E+00	1.20E-305	0.432	0.287	0.026	3.87E-301	0.00E+00	1.472	0.568	0.026	0.00E+00	2.96E-86	1.161	0.535	0.026	9.54E-82

IFITM1	6.64E-152	0.859	0.560	0.075	2.14E-147	1.72E-137	0.213	0.263	0.075	5.54E-133	5.18E-88	0.849	0.354	0.075	1.67E-83	2.05E-68	2.669	0.767	0.075	6.59E-64
IFITM3	2.57E-129	2.587	0.919	0.410	8.29E-125	0.00E+00	2.115	0.836	0.410	0.00E+00	3.23E-129	2.460	0.851	0.410	1.04E-124	6.87E-26	2.874	0.907	0.410	2.21E-21
IL4R	3.90E-101	0.546	0.444	0.068	1.26E-96	3.29E-239	0.375	0.330	0.068	1.06E-234	9.01E-69	0.293	0.311	0.068	2.90E-64	4.31E-43	0.368	0.628	0.068	1.39E-38
IMP3	3.48E-22	0.340	0.645	0.358	1.12E-17	3.04E-205	0.446	0.673	0.358	9.80E-201	1.74E-26	0.295	0.617	0.358	5.60E-22	1.60E-08	0.452	0.791	0.358	5.16E-04
IMPA2	6.92E-10	0.199	0.610	0.407	2.23E-05	2.34E-261	0.671	0.731	0.407	7.54E-257	4.17E-51	0.711	0.696	0.407	1.34E-46	3.52E-06	0.409	0.744	0.407	1.13E-01
ISOC2	1.77E-21	0.348	0.548	0.274	5.69E-17	0.00E+00	0.737	0.652	0.274	0.00E+00	4.27E-58	0.575	0.601	0.274	1.38E-53	2.45E-10	0.485	0.721	0.274	7.90E-06
ITPA	1.00E-17	0.205	0.514	0.258	3.23E-13	7.30E-229	0.415	0.584	0.258	2.35E-224	1.62E-26	0.259	0.500	0.258	5.21E-22	2.45E-07	0.156	0.698	0.258	7.88E-03
JOSD2	4.50E-35	0.360	0.494	0.183	1.45E-30	0.00E+00	0.496	0.554	0.183	0.00E+00	2.34E-86	0.588	0.559	0.183	7.55E-82	1.67E-14	0.206	0.698	0.183	5.36E-10
KCTD1	3.97E-14	0.152	0.494	0.259	1.28E-09	1.25E-128	0.284	0.514	0.259	4.02E-124	6.70E-22	0.210	0.477	0.259	2.16E-17	5.14E-09	0.301	0.698	0.259	1.66E-04
KRT14	1.10E-38	1.135	0.954	0.769	3.55E-34	0.00E+00	1.482	0.978	0.769	0.00E+00	4.22E-38	1.141	0.872	0.769	1.36E-33	1.77E-05	0.853	0.884	0.769	5.68E-01
KRT16	5.17E-102	3.787	0.815	0.305	1.66E-97	0.00E+00	4.884	0.840	0.305	0.00E+00	1.16E-128	3.452	0.764	0.305	3.74E-124	6.61E-11	2.839	0.674	0.305	2.13E-06
KRT5	5.98E-19	0.674	0.919	0.704	1.93E-14	5.78E-256	0.952	0.910	0.704	1.86E-251	3.87E-22	0.484	0.872	0.704	1.25E-17	3.49E-11	1.439	0.977	0.704	1.13E-06
KRT6A	8.77E-262	4.435	0.907	0.148	2.83E-257	0.00E+00	4.936	0.904	0.148	0.00E+00	0.00E+00	3.603	0.903	0.148	0.00E+00	2.78E-57	4.473	0.930	0.148	8.94E-53
KRT6B	2.29E-152	1.759	0.660	0.101	7.38E-148	0.00E+00	2.713	0.738	0.101	0.00E+00	8.92E-131	0.881	0.518	0.101	2.87E-126	8.44E-22	1.845	0.558	0.101	2.72E-17
KRT6C	0.00E+00	3.613	0.618	0.016	0.00E+00	0.00E+00	4.145	0.666	0.016	0.00E+00	0.00E+00	2.265	0.410	0.016	0.00E+00	2.84E-91	3.085	0.442	0.016	9.13E-87
LAD1	8.61E-24	0.429	0.660	0.372	2.77E-19	0.00E+00	1.056	0.781	0.372	0.00E+00	2.00E-56	0.698	0.687	0.372	6.43E-52	2.19E-10	0.815	0.791	0.372	7.06E-06
LAMTOR1	1.86E-17	0.243	0.583	0.308	5.99E-13	0.00E+00	0.692	0.686	0.308	0.00E+00	8.13E-50	0.488	0.622	0.308	2.62E-45	2.53E-08	0.338	0.721	0.308	8.15E-04
LGALS3BP	6.43E-184	1.412	0.699	0.110	2.07E-179	0.00E+00	0.717	0.535	0.110	0.00E+00	1.06E-269	1.398	0.689	0.110	3.41E-265	3.58E-46	1.421	0.767	0.110	1.15E-41
LMAN2	3.28E-16	0.289	0.583	0.336	1.06E-11	1.02E-203	0.478	0.642	0.336	3.29E-199	5.12E-33	0.338	0.604	0.336	1.65E-28	6.71E-08	0.374	0.721	0.336	2.16E-03
LSM7	1.91E-14	0.377	0.695	0.471	6.16E-10	2.85E-137	0.464	0.709	0.471	9.17E-133	3.67E-50	0.691	0.752	0.471	1.18E-45	5.69E-07	0.654	0.837	0.471	1.83E-02
LTB4R2	3.96E-24	0.239	0.405	0.156	1.27E-19	6.60E-272	0.490	0.492	0.156	2.13E-267	6.21E-46	0.305	0.423	0.156	2.00E-41	1.02E-24	0.746	0.721	0.156	3.28E-20
LYPLA1	1.61E-14	0.144	0.463	0.235	5.20E-10	5.15E-195	0.314	0.541	0.235	1.66E-190	7.60E-30	0.291	0.466	0.235	2.45E-25	2.40E-08	0.141	0.651	0.235	7.73E-04
MALL	4.63E-27	0.229	0.266	0.076	1.49E-22	0.00E+00	0.602	0.405	0.076	0.00E+00	2.61E-46	0.196	0.282	0.076	8.41E-42	1.48E-10	0.855	0.349	0.076	4.77E-06
MAPKAP1	4.06E-13	0.120	0.270	0.115	1.31E-08	1.79E-90	0.138	0.286	0.115	5.77E-86	2.33E-15	0.102	0.250	0.115	7.51E-11	1.26E-08	0.199	0.419	0.115	4.06E-04
MDFI	5.72E-60	0.356	0.367	0.073	1.84E-55	0.00E+00	0.395	0.403	0.073	0.00E+00	4.31E-58	0.284	0.300	0.073	1.39E-53	1.89E-14	0.180	0.395	0.073	6.08E-10
MIB2	2.90E-12	0.226	0.614	0.385	9.33E-08	2.17E-104	0.252	0.632	0.385	6.98E-100	4.51E-27	0.332	0.644	0.385	1.45E-22	1.23E-04	0.221	0.698	0.385	1.00E+00
MICALL1	8.53E-14	0.224	0.205	0.075	2.75E-09	3.28E-120	0.213	0.248	0.075	1.06E-115	4.62E-59	0.267	0.306	0.075	1.49E-54	4.64E-20	0.225	0.465	0.075	1.50E-15
MICOS13	1.29E-13	0.263	0.633	0.394	4.15E-09	8.48E-182	0.423	0.688	0.394	2.73E-177	9.29E-58	0.570	0.734	0.394	2.99E-53	1.16E-09	0.926	0.837	0.394	3.75E-05
MIF	2.58E-26	0.807	0.931	0.803	8.32E-22	8.01E-231	0.716	0.924	0.803	2.58E-226	2.57E-104	1.286	0.962	0.803	8.27E-100	8.73E-10	1.060	0.953	0.803	2.81E-05
MPHOSPH6	1.63E-50	0.421	0.533	0.172	5.23E-46	0.00E+00	0.671	0.598	0.172	0.00E+00	3.88E-76	0.434	0.527	0.172	1.25E-71	6.34E-09	0.255	0.535	0.172	2.04E-04
MRPL12	1.86E-57	0.663	0.591	0.197	5.98E-53	0.00E+00	1.057	0.699	0.197	0.00E+00	9.50E-120	0.789	0.637	0.197	3.06E-115	9.02E-13	0.618	0.605	0.197	2.90E-08
MRPL20	1.62E-21	0.337	0.676	0.380	5.21E-17	0.00E+00	0.801	0.767	0.380	0.00E+00	1.07E-41	0.422	0.669	0.380	3.45E-37	3.77E-06	0.283	0.744	0.380	1.21E-01
MRPL27	1.67E-28	0.365	0.610	0.296	5.39E-24	0.00E+00	0.644	0.679	0.296	0.00E+00	3.17E-74	0.589	0.673	0.296	1.02E-69	3.61E-05	0.118	0.628	0.296	1.00E+00
MRPL52	1.18E-15	0.219	0.687	0.414	3.80E-11	4.22E-95	0.291	0.646	0.414	1.36E-90	5.21E-61	0.635	0.764	0.414	1.68E-56	4.77E-06	0.298	0.767	0.414	1.53E-01
MRPL54	6.13E-14	0.221	0.541	0.304	1.97E-09	2.88E-210	0.452	0.616	0.304	9.29E-206	1.45E-38	0.356	0.599	0.304	4.68E-34	7.02E-06	0.177	0.674	0.304	2.26E-01
MRPL57	2.60E-25	0.325	0.687	0.371	8.36E-21	4.33E-176	0.408	0.662	0.371	1.39E-171	2.71E-57	0.553	0.716	0.371	8.72E-53	2.96E-08	0.644	0.791	0.371	9.53E-04
MRPS34	1.27E-13	0.224	0.637	0.382	4.09E-09	6.80E-214	0.554	0.679	0.382	2.19E-209	1.69E-44	0.492	0.678	0.382	5.45E-40	4.74E-07	0.423	0.767	0.382	1.53E-02
MRPS7	3.78E-16	0.205	0.498	0.247	1.22E-11	1.54E-269	0.470	0.600	0.247	4.97E-265	2.12E-48	0.354	0.565	0.247	6.84E-44	1.61E-06	0.129	0.605	0.247	5.18E-02
MTG1	4.94E-20	0.135	0.363	0.141	1.59E-15	1.40E-118	0.109	0.356	0.141	4.49E-114	3.25E-45	0.216	0.403	0.141	1.05E-40	2.18E-16	0.284	0.605	0.141	7.01E-12
MVP	7.82E-75	0.720	0.602	0.171	2.52E-70	0.00E+00	0.590	0.549	0.171	0.00E+00	1.51E-95	0.704	0.556	0.171	4.85E-91	9.70E-22	0.570	0.721	0.171	3.12E-17
MX1	0.00E+00	1.235	0.699	0.025	0.00E+00	8.76E-199	0.267	0.212	0.025	2.82E-194	3.32E-302	0.714	0.435	0.025	1.07E-297	1.70E-104	0.820	0.581	0.025	5.47E-100
NAPRT	1.15E-28	0.430	0.502	0.210	3.71E-24	0.00E+00	1.306	0.760	0.210	0.00E+00	1.19E-134	0.930	0.682	0.210	3.84E-130	1.96E-05	0.121	0.512	0.210	6.33E-01
NCLN	7.88E-24	0.219	0.255	0.075	2.54E-19	4.66E-147	0.215	0.271	0.075	1.50E-142	2.41E-36	0.219	0.250	0.075	7.76E-32	2.69E-25	0.490	0.512	0.075	8.67E-21
NDUFS8	2.37E-18	0.312	0.633	0.377	7.62E-14	1.40E-213	0.449	0.689	0.377	4.51E-209	8.78E-49	0.495	0.689	0.377	2.83E-44	9.81E-08	0.397	0.767	0.377	3.16E-03
NME2	1.31E-08	0.321	0.876	0.773	4.23E-04	1.41E-261	0.745	0.910	0.773	4.54E-257	1.27E-30	0.516	0.899	0.773	4.08E-26	5.65E-05	0.558	0.930	0.773	1.00E+00
NMI	2.32E-122	0.656	0.579	0.103	7.47E-118	0.00E+00	0.503	0.501	0.103	0.00E+00	7.17E-105	0.447	0.455	0.103	2.31E-100	1.16E-23	0.570	0.581	0.103	3.74E-19
NOP10	1.24E-16	0.364	0.668	0.452	3.99E-12	2.04E-250	0.636	0.745	0.452	6.58E-246	3.40E-47	0.509	0.745	0.452	1.09E-42	1.91E-04	0.395	0.767	0.452	1.00E+00
NT5C	2.23E-13	0.136	0.475	0.252	7.20E-09	4.13E-245	0.418	0.597	0.252	1.33E-240	1.37E-35	0.306	0.527	0.252	4.42E-31	4.34E-11	0.272	0.721	0.252	1.40E-06
NUCB1	2.32E-32	0.439	0.548	0.236	7.48E-28	2.77E-100	0.181	0.465	0.236	8.92E-96	1.74E-38	0.353	0.514	0.236	5.60E-34	1.93E-16	0.672	0.767	0.236	6.22E-12
NUDT22	6.22E-34	0.230	0.405	0.130	2.00E-29	4.17E-255	0.319	0.447	0.130	1.34E-250	1.27E-57	0.307	0.412	0.130	4.09E-53	7.99E-15	0.598	0.558	0.130	2.57E-10
NUTF2	3.64E-14	0.102	0.444	0.220	1.17E-09	1.85E-175	0.328	0.502	0.220	5.94E-171	1.75E-23	0.205	0.435	0.220	5.63E-19	4.87E-11	0.323	0.651	0.220	1.57E-06

OAS1	2.55E-261	0.781	0.544	0.037	8.20E-257	3.93E-287	0.302	0.300	0.037	1.27E-282	7.09E-109	0.314	0.284	0.037	2.28E-104	1.69E-65	0.423	0.558	0.037	5.44E-61
OAS3	4.29E-237	0.657	0.486	0.031	1.38E-232	1.02E-139	0.196	0.183	0.031	3.29E-135	3.88E-81	0.301	0.225	0.031	1.25E-76	3.62E-39	0.386	0.395	0.031	1.17E-34
ODF3B	1.58E-22	0.194	0.290	0.096	5.08E-18	0.00E+00	0.452	0.453	0.096	0.00E+00	1.44E-121	0.493	0.468	0.096	4.63E-117	3.24E-49	1.028	0.744	0.096	1.04E-44
ORMDL2	9.68E-44	0.397	0.510	0.174	3.12E-39	0.00E+00	0.575	0.577	0.174	0.00E+00	5.03E-45	0.303	0.450	0.174	1.62E-40	7.33E-14	0.225	0.651	0.174	2.36E-09
P4HB	2.10E-13	0.333	0.761	0.585	6.75E-09	0.00E+00	0.804	0.856	0.585	0.00E+00	1.15E-35	0.466	0.795	0.585	3.69E-31	4.57E-09	0.834	0.907	0.585	1.47E-04
PARP14	1.16E-233	1.133	0.591	0.051	3.72E-229	4.28E-128	0.196	0.214	0.051	1.38E-123	6.26E-143	0.623	0.378	0.051	2.02E-138	1.26E-28	0.451	0.442	0.051	4.06E-24
PARP9	7.35E-198	0.601	0.490	0.041	2.37E-193	0.00E+00	0.405	0.344	0.041	0.00E+00	7.03E-196	0.512	0.405	0.041	2.26E-191	6.52E-82	0.596	0.651	0.041	2.10E-77
PCYT2	5.31E-26	0.137	0.189	0.043	1.71E-21	8.66E-117	0.110	0.189	0.043	2.79E-112	1.35E-41	0.138	0.194	0.043	4.36E-37	3.64E-27	0.160	0.395	0.043	1.17E-22
PDAP1	1.07E-19	0.233	0.641	0.354	3.45E-15	3.27E-281	0.595	0.690	0.354	1.05E-276	2.39E-26	0.259	0.604	0.354	7.69E-22	6.74E-11	0.556	0.791	0.354	2.17E-06
PDCD5	1.58E-19	0.534	0.718	0.463	5.09E-15	1.52E-183	0.545	0.736	0.463	4.88E-179	1.01E-15	0.275	0.664	0.463	3.25E-11	6.18E-05	0.620	0.767	0.463	1.00E+00
PDLIM1	3.63E-09	0.421	0.641	0.469	1.17E-04	4.89E-81	0.380	0.684	0.469	1.57E-76	3.51E-07	0.203	0.606	0.469	1.13E-02	2.52E-10	1.459	0.814	0.469	8.12E-06
PDPN	1.10E-104	0.640	0.313	0.032	3.54E-100	5.76E-165	0.259	0.203	0.032	1.86E-160	5.64E-27	0.167	0.135	0.032	1.82E-22	1.21E-29	0.402	0.349	0.032	3.90E-25
PDZK1IP1	1.19E-91	1.172	0.614	0.162	3.84E-87	0.00E+00	2.213	0.838	0.162	0.00E+00	3.74E-194	1.531	0.678	0.162	1.21E-189	1.18E-33	1.314	0.814	0.162	3.81E-29
PGAM1	5.85E-17	0.449	0.710	0.511	1.88E-12	2.41E-194	0.585	0.757	0.511	7.75E-190	5.01E-11	0.185	0.651	0.511	1.61E-06	1.42E-19	1.538	0.977	0.511	4.56E-15
PGM2	9.08E-20	0.243	0.405	0.178	2.93E-15	1.37E-140	0.265	0.422	0.178	4.41E-136	2.90E-13	0.124	0.324	0.178	9.35E-09	1.50E-10	0.298	0.558	0.178	4.83E-06
PKM	4.57E-26	0.824	0.865	0.699	1.47E-21	4.20E-211	0.670	0.883	0.699	1.35E-206	1.34E-15	0.420	0.858	0.699	4.31E-11	2.52E-11	1.181	0.860	0.699	8.11E-07
PKP3	2.54E-12	0.307	0.764	0.543	8.19E-08	5.76E-134	0.473	0.787	0.543	1.86E-129	2.13E-27	0.380	0.773	0.543	6.87E-23	1.21E-03	0.517	0.814	0.543	1.00E+00
PLAAT4	9.10E-182	0.652	0.471	0.041	2.93E-177	5.18E-105	0.192	0.173	0.041	1.67E-100	2.11E-161	0.600	0.365	0.041	6.78E-157	1.14E-76	0.888	0.628	0.041	3.67E-72
PLBD1	1.03E-37	0.209	0.359	0.099	3.33E-33	9.46E-304	0.496	0.421	0.099	3.05E-299	6.58E-33	0.145	0.288	0.099	2.12E-28	5.76E-14	0.783	0.442	0.099	1.86E-09
PLEKHJ1	1.36E-18	0.118	0.402	0.169	4.38E-14	3.82E-291	0.410	0.527	0.169	1.23E-286	3.16E-58	0.344	0.480	0.169	1.02E-53	4.74E-12	0.443	0.581	0.169	1.53E-07
PLSCR1	9.94E-170	0.921	0.622	0.087	3.20E-165	6.17E-232	0.300	0.362	0.087	1.99E-227	2.35E-69	0.397	0.354	0.087	7.56E-65	4.20E-39	0.682	0.674	0.087	1.35E-34
PPIB	8.18E-26	0.611	0.772	0.563	2.64E-21	0.00E+00	0.803	0.824	0.563	0.00E+00	3.73E-27	0.432	0.768	0.563	1.20E-22	1.23E-12	1.351	0.860	0.563	3.98E-08
PPP1CA	4.93E-08	0.133	0.591	0.416	1.59E-03	5.65E-254	0.609	0.724	0.416	1.82E-249	1.36E-38	0.452	0.689	0.416	4.38E-34	5.51E-09	0.747	0.791	0.416	1.78E-04
PRR13	7.01E-18	0.139	0.490	0.231	2.26E-13	1.57E-186	0.305	0.534	0.231	5.05E-182	2.30E-47	0.305	0.543	0.231	7.39E-43	2.25E-12	0.641	0.698	0.231	7.25E-08
PSMA7	3.14E-20	0.497	0.819	0.624	1.01E-15	5.89E-289	0.806	0.847	0.624	1.90E-284	4.25E-32	0.556	0.777	0.624	1.37E-27	8.39E-04	0.272	0.837	0.624	1.00E+00
PSMB10	1.33E-139	1.057	0.656	0.124	4.30E-135	0.00E+00	0.747	0.522	0.124	0.00E+00	4.06E-181	1.067	0.610	0.124	1.31E-176	1.89E-41	1.337	0.767	0.124	6.08E-37
PSMB8	9.39E-55	0.930	0.722	0.344	3.02E-50	8.30E-238	0.640	0.659	0.344	2.67E-233	3.35E-53	0.672	0.651	0.344	1.08E-48	4.60E-19	1.349	0.837	0.344	1.48E-14
PSMD13	2.20E-15	0.192	0.494	0.257	7.08E-11	6.86E-127	0.233	0.507	0.257	2.21E-122	1.13E-15	0.136	0.441	0.257	3.63E-11	1.95E-09	0.293	0.698	0.257	6.27E-05
PSME1	9.53E-54	1.108	0.834	0.520	3.07E-49	1.94E-199	0.672	0.772	0.520	6.25E-195	8.30E-59	0.776	0.813	0.520	2.67E-54	1.12E-15	1.326	0.884	0.520	3.62E-11
PSME2	3.20E-87	1.643	0.846	0.457	1.03E-82	0.00E+00	0.958	0.785	0.457	0.00E+00	5.05E-107	1.310	0.840	0.457	1.63E-102	4.06E-17	1.663	0.860	0.457	1.31E-12
PTPMT1	3.07E-14	0.126	0.297	0.125	9.90E-10	2.07E-92	0.135	0.301	0.125	6.68E-88	1.39E-20	0.153	0.288	0.125	4.47E-16	4.68E-13	0.285	0.512	0.125	1.51E-08
PYCARD	7.76E-68	1.297	0.811	0.426	2.50E-63	0.00E+00	1.297	0.837	0.426	0.00E+00	1.27E-129	1.364	0.833	0.426	4.10E-125	4.05E-13	1.194	0.860	0.426	1.30E-08
PYM1	3.70E-19	0.113	0.375	0.155	1.19E-14	6.07E-253	0.361	0.481	0.155	1.96E-248	1.75E-46	0.265	0.426	0.155	5.64E-42	1.82E-09	0.174	0.512	0.155	5.86E-05
R3HDM4	3.20E-16	0.112	0.386	0.171	1.03E-11	1.05E-111	0.212	0.389	0.171	3.40E-107	6.15E-13	0.173	0.311	0.171	1.98E-08	2.84E-11	0.316	0.581	0.171	9.14E-07
RAB25	7.42E-13	0.322	0.641	0.424	2.39E-08	1.95E-280	0.859	0.741	0.424	6.29E-276	7.08E-26	0.449	0.653	0.424	2.28E-21	1.27E-03	0.192	0.744	0.424	1.00E+00
RAB38	2.44E-23	0.319	0.544	0.271	7.86E-19	9.48E-228	0.457	0.599	0.271	3.05E-223	3.38E-17	0.207	0.462	0.271	1.09E-12	1.17E-14	0.659	0.767	0.271	3.77E-10
RAN	6.68E-04	0.155	0.799	0.688	1.00E+00	1.10E-175	0.673	0.855	0.688	3.53E-171	5.29E-06	0.160	0.824	0.688	1.70E-01	5.49E-07	0.939	0.930	0.688	1.77E-02
RBCK1	5.51E-28	0.328	0.541	0.236	1.77E-23	2.02E-92	0.204	0.453	0.236	6.49E-88	4.06E-34	0.314	0.500	0.236	1.31E-29	5.00E-12	0.719	0.674	0.236	1.61E-07
RNASEK	2.36E-09	0.212	0.792	0.643	7.60E-05	7.30E-45	0.144	0.799	0.643	2.35E-40	2.23E-08	0.118	0.795	0.643	7.17E-04	4.59E-05	0.589	0.860	0.643	1.00E+00
RPL22L1	1.90E-25	0.743	0.819	0.578	6.11E-21	3.68E-137	0.531	0.783	0.578	1.19E-132	7.79E-03	0.176	0.714	0.578	1.00E+00	3.78E-06	0.718	0.837	0.578	1.22E-01
RPL28	1.47E-04	0.190	0.965	0.926	1.00E+00	1.15E-66	0.234	0.985	0.926	3.71E-62	2.23E-55	0.552	0.993	0.926	7.19E-51	3.02E-08	0.554	1.000	0.926	9.74E-04
S100A10	1.70E-25	0.993	0.911	0.763	5.48E-21	0.00E+00	1.096	0.927	0.763	0.00E+00	6.73E-15	0.369	0.917	0.763	2.17E-10	8.47E-05	0.765	0.837	0.763	1.00E+00
S100A11	8.18E-65	1.380	0.969	0.858	2.63E-60	0.00E+00	1.344	0.978	0.858	0.00E+00	4.47E-79	1.041	0.982	0.858	1.44E-74	7.98E-16	1.531	1.000	0.858	2.57E-11
S100A16	4.68E-41	1.040	0.873	0.672	1.51E-36	0.00E+00	1.294	0.914	0.672	0.00E+00	2.67E-71	0.961	0.883	0.672	8.59E-67	7.26E-15	1.302	0.953	0.672	2.34E-10
S100A7	1.52E-241	4.789	0.575	0.048	4.89E-237	0.00E+00	5.245	0.868	0.048	0.00E+00	0.00E+00	5.865	0.782	0.048	0.00E+00	8.19E-140	6.765	0.884	0.048	2.64E-135
S100A8	0.00E+00	6.663	0.923	0.093	0.00E+00	0.00E+00	6.980	0.991	0.093	0.00E+00	0.00E+00	6.788	0.962	0.093	0.00E+00	3.65E-85	6.468	0.907	0.093	1.18E-80
S100A9	0.00E+00	6.633	0.911	0.094	0.00E+00	0.00E+00	7.396	0.994	0.094	0.00E+00	0.00E+00	6.574	0.944	0.094	0.00E+00	1.86E-84	6.614	0.907	0.094	6.00E-80
SELENOW	3.23E-11	0.284	0.795	0.613	1.04E-06	6.49E-140	0.370	0.830	0.613	2.09E-135	7.73E-12	0.147	0.788	0.613	2.49E-07	3.56E-04	0.339	0.837	0.613	1.00E+00
SERF2	4.76E-18	0.537	0.927	0.797	1.53E-13	0.00E+00	0.859	0.932	0.797	0.00E+00	2.97E-125	1.081	0.966	0.797	9.56E-121	1.65E-07	0.692	0.907	0.797	5.32E-03
SERINC2	1.97E-19	0.240	0.320	0.126	6.36E-15	9.89E-251	0.373	0.431	0.126	3.19E-246	2.87E-38	0.258	0.349	0.126	9.25E-34	4.34E-23	0.787	0.628	0.126	1.40E-18
SERPINB1	1.36E-94	0.894	0.606	0.143	4.39E-90	0.00E+00	0.876	0.606	0.143	0.00E+00	2.64E-56	0.318	0.423	0.143	8.50E-52	8.74E-35	1.013	0.791	0.143	2.82E-30

SERPINB3	0.00E+00	1.621	0.394	0.008	0.00E+00	0.00E+00	3.416	0.814	0.008	0.00E+00	0.00E+00	1.946	0.588	0.008	0.00E+00	1.73E-220	1.849	0.535	0.008	5.58E-216
SERPINB4	0.00E+00	2.225	0.490	0.007	0.00E+00	0.00E+00	4.680	0.848	0.007	0.00E+00	0.00E+00	2.841	0.682	0.007	0.00E+00	8.54E-239	2.696	0.535	0.007	2.75E-234
SERPINB6	1.50E-24	0.102	0.239	0.066	4.84E-20	1.17E-298	0.333	0.367	0.066	3.77E-294	5.38E-162	0.527	0.448	0.066	1.73E-157	2.77E-21	0.178	0.442	0.066	8.93E-17
SH3BGRL3	7.92E-38	1.060	0.869	0.675	2.55E-33	0.00E+00	0.999	0.892	0.675	0.00E+00	1.72E-42	0.678	0.883	0.675	5.55E-38	2.67E-10	1.022	0.930	0.675	8.61E-06
SHISA5	1.39E-31	0.318	0.421	0.147	4.48E-27	0.00E+00	0.494	0.507	0.147	0.00E+00	8.58E-72	0.481	0.473	0.147	2.76E-67	9.10E-29	0.722	0.744	0.147	2.93E-24
SLC27A4	5.08E-33	0.165	0.239	0.054	1.64E-28	8.23E-208	0.296	0.280	0.054	2.65E-203	1.88E-36	0.150	0.207	0.054	6.05E-32	2.45E-16	0.201	0.349	0.054	7.90E-12
SLC50A1	5.29E-37	0.240	0.386	0.114	1.70E-32	9.26E-217	0.272	0.394	0.114	2.98E-212	4.02E-34	0.165	0.318	0.114	1.29E-29	5.24E-10	0.135	0.442	0.114	1.69E-05
SP110	1.05E-80	0.373	0.394	0.066	3.37E-76	1.47E-173	0.224	0.278	0.066	4.74E-169	2.09E-79	0.311	0.324	0.066	6.73E-75	3.04E-29	0.352	0.512	0.066	9.78E-25
SRD5A1	1.77E-39	0.272	0.537	0.191	5.71E-35	1.04E-116	0.118	0.427	0.191	3.35E-112	5.29E-38	0.293	0.450	0.191	1.70E-33	6.24E-23	0.949	0.767	0.191	2.01E-18
SRM	2.65E-23	0.329	0.475	0.216	8.54E-19	0.00E+00	0.633	0.584	0.216	0.00E+00	7.54E-111	0.842	0.646	0.216	2.43E-106	1.44E-10	0.338	0.651	0.216	4.63E-06
SSBP1	7.32E-15	0.321	0.741	0.500	2.36E-10	8.68E-207	0.576	0.765	0.500	2.80E-202	1.02E-28	0.357	0.748	0.500	3.29E-24	1.62E-03	0.400	0.814	0.500	1.00E+00
SSNA1	5.45E-21	0.315	0.595	0.307	1.76E-16	0.00E+00	0.650	0.686	0.307	0.00E+00	5.21E-63	0.601	0.662	0.307	1.68E-58	2.45E-09	0.426	0.721	0.307	7.90E-05
STMP1	2.76E-13	0.179	0.571	0.339	8.89E-09	8.41E-188	0.395	0.643	0.339	2.71E-183	1.11E-40	0.428	0.637	0.339	3.59E-36	2.46E-09	0.700	0.791	0.339	7.93E-05
SURF2	4.32E-19	0.172	0.517	0.245	1.39E-14	6.66E-109	0.233	0.480	0.245	2.14E-104	9.05E-19	0.175	0.450	0.245	2.91E-14	4.00E-07	0.180	0.628	0.245	1.29E-02
SYNGR2	4.60E-33	0.634	0.734	0.412	1.48E-28	1.11E-70	0.130	0.636	0.412	3.57E-66	9.42E-17	0.151	0.637	0.412	3.03E-12	3.01E-11	1.049	0.837	0.412	9.70E-07
TALDO1	4.49E-11	0.185	0.614	0.410	1.45E-06	4.96E-283	0.631	0.730	0.410	1.60E-278	1.12E-33	0.432	0.658	0.410	3.59E-29	3.72E-09	0.570	0.791	0.410	1.20E-04
TAPBP	3.09E-104	1.334	0.830	0.305	9.96E-100	7.95E-182	0.426	0.614	0.305	2.56E-177	1.36E-39	0.431	0.604	0.305	4.37E-35	1.36E-14	0.844	0.791	0.305	4.39E-10
TBCB	1.17E-38	0.493	0.587	0.244	3.77E-34	8.49E-191	0.324	0.549	0.244	2.73E-186	4.79E-36	0.290	0.518	0.244	1.54E-31	6.17E-13	0.312	0.744	0.244	1.99E-08
TIMM8B	5.88E-27	0.509	0.718	0.410	1.89E-22	7.95E-198	0.507	0.700	0.410	2.56E-193	4.94E-97	0.945	0.797	0.410	1.59E-92	1.31E-04	0.161	0.744	0.410	1.00E+00
TMED10	1.82E-09	0.124	0.633	0.425	5.87E-05	9.27E-154	0.383	0.693	0.425	2.98E-149	1.14E-19	0.216	0.644	0.425	3.66E-15	8.47E-06	0.344	0.767	0.425	2.73E-01
TMEM54	3.59E-56	0.590	0.517	0.156	1.16E-51	0.00E+00	1.088	0.622	0.156	0.00E+00	1.47E-133	0.830	0.597	0.156	4.72E-129	6.49E-17	0.301	0.651	0.156	2.09E-12
TMSB10	2.64E-100	2.006	0.988	0.775	8.49E-96	1.67E-238	0.781	0.939	0.775	5.38E-234	4.03E-102	1.754	0.973	0.775	1.30E-97	5.09E-18	1.917	0.953	0.775	1.64E-13
TNFSF10	9.34E-127	1.216	0.699	0.157	3.01E-122	0.00E+00	1.262	0.729	0.157	0.00E+00	1.07E-220	1.281	0.739	0.157	3.45E-216	2.88E-30	1.501	0.744	0.157	9.29E-26
TOLLIP	8.84E-14	0.165	0.494	0.275	2.85E-09	1.01E-277	0.578	0.623	0.275	3.27E-273	7.06E-28	0.256	0.514	0.275	2.27E-23	3.32E-06	0.839	0.628	0.275	1.07E-01
TRAPPC1	2.04E-28	0.392	0.622	0.295	6.56E-24	5.63E-218	0.419	0.623	0.295	1.81E-213	4.54E-72	0.619	0.676	0.295	1.46E-67	5.95E-06	0.259	0.628	0.295	1.92E-01
TRAPPC5	9.20E-19	0.378	0.691	0.436	2.96E-14	0.00E+00	0.739	0.757	0.436	0.00E+00	1.58E-77	0.787	0.777	0.436	5.09E-73	6.37E-07	0.580	0.814	0.436	2.05E-02
TRIM47	5.86E-59	0.598	0.479	0.129	1.89E-54	4.60E-228	0.439	0.419	0.129	1.48E-223	1.13E-31	0.254	0.333	0.129	3.63E-27	3.40E-17	0.344	0.581	0.129	1.09E-12
TUBB6	1.02E-75	0.959	0.691	0.240	3.28E-71	0.00E+00	1.142	0.723	0.240	0.00E+00	6.64E-53	0.496	0.547	0.240	2.14E-48	9.80E-12	0.395	0.721	0.240	3.16E-07
TXNDC17	1.70E-34	0.894	0.757	0.451	5.49E-30	0.00E+00	1.192	0.788	0.451	0.00E+00	3.31E-79	1.104	0.793	0.451	1.07E-74	6.19E-11	1.180	0.767	0.451	1.99E-06
TYMP	2.05E-227	3.544	0.961	0.248	6.61E-223	0.00E+00	2.158	0.894	0.248	0.00E+00	0.00E+00	2.815	0.937	0.248	0.00E+00	1.37E-34	2.293	0.907	0.248	4.40E-30
UBE2F	1.35E-31	0.280	0.405	0.137	4.36E-27	0.00E+00	0.486	0.496	0.137	0.00E+00	5.97E-52	0.297	0.405	0.137	1.92E-47	2.82E-15	0.286	0.581	0.137	9.08E-11
UBE2L6	1.81E-180	1.317	0.680	0.110	5.83E-176	0.00E+00	0.588	0.502	0.110	0.00E+00	1.86E-180	0.912	0.577	0.110	5.99E-176	2.16E-45	1.375	0.744	0.110	6.97E-41
UQCR10	1.59E-22	0.590	0.772	0.535	5.13E-18	5.78E-191	0.520	0.781	0.535	1.86E-186	5.20E-83	0.882	0.860	0.535	1.68E-78	7.06E-09	1.047	0.860	0.535	2.27E-04
UQCR11	4.33E-24	0.462	0.807	0.547	1.39E-19	1.50E-194	0.405	0.799	0.547	4.85E-190	1.20E-107	0.990	0.885	0.547	3.87E-103	2.22E-07	0.877	0.884	0.547	7.14E-03
VPS25	3.18E-31	0.202	0.413	0.142	1.02E-26	5.23E-302	0.412	0.493	0.142	1.68E-297	5.27E-57	0.305	0.428	0.142	1.70E-52	2.24E-16	0.266	0.605	0.142	7.21E-12
VSNL1	5.87E-57	0.503	0.537	0.160	1.89E-52	1.29E-304	0.396	0.530	0.160	4.15E-300	1.22E-56	0.440	0.455	0.160	3.92E-52	4.55E-24	0.846	0.721	0.160	1.46E-19
WDR18	1.74E-32	0.200	0.417	0.139	5.62E-28	1.10E-183	0.273	0.403	0.139	3.55E-179	1.49E-36	0.209	0.369	0.139	4.81E-32	4.47E-19	0.375	0.628	0.139	1.44E-14
ZNRD2	3.68E-20	0.157	0.405	0.167	1.19E-15	9.29E-237	0.390	0.480	0.167	2.99E-232	1.49E-44	0.343	0.435	0.167	4.80E-40	5.82E-15	0.626	0.651	0.167	1.87E-10

	MF18					MF21					MF24				
	p val	avg logFC	pct.1	pct.2	p val adj	p val	avg logFC	pct.1	pct.2	p val adj	p_val	avg logFC	pct.1	pct.2	p val adj
ACP5	8.98E-39	0.664	0.417	0.056	2.89E-34	0.00E+00	0.651	0.498	0.056	0.00E+00	2.94E-09	0.110	0.303	0.056	9.48E-05
AGTRAP	1.99E-23	0.433	0.472	0.106	6.41E-19	0.00E+00	0.814	0.648	0.106	0.00E+00	5.73E-12	0.334	0.485	0.106	1.85E-07
AKR1B10	4.59E-35	0.439	0.347	0.043	1.48E-30	0.00E+00	1.151	0.629	0.043	0.00E+00	3.42E-35	0.688	0.485	0.043	1.10E-30
ALDH16A1	2.11E-23	0.403	0.486	0.111	6.81E-19	2.65E-119	0.241	0.382	0.111	8.55E-115	1.04E-06	0.172	0.394	0.111	3.36E-02
ALDH3B2	3.96E-05	0.520	0.292	0.139	1.00E+00	1.21E-268	0.597	0.588	0.139	3.88E-264	2.49E-02	0.113	0.273	0.139	1.00E+00
ALG3	9.72E-07	0.101	0.347	0.134	3.13E-02	3.09E-161	0.292	0.476	0.134	9.94E-157	2.53E-07	0.208	0.455	0.134	8.15E-03
ANGPTL4	1.77E-50	1.609	0.708	0.128	5.69E-46	0.00E+00	0.908	0.619	0.128	0.00E+00	9.55E-04	0.391	0.333	0.128	1.00E+00

ANKRD29	6.38E-45	0.368	0.319	0.028	2.05E-40	7.38E-264	0.342	0.319	0.028	2.38E-259	5.32E-25	0.339	0.333	0.028	1.71E-20
AP1S1	5.96E-05	0.190	0.444	0.236	1.00E+00	6.53E-181	0.462	0.639	0.236	2.10E-176	3.83E-05	0.249	0.545	0.236	1.00E+00
AP2S1	1.33E-02	0.135	0.583	0.475	1.00E+00	1.51E-186	0.699	0.818	0.475	4.87E-182	3.81E-06	0.915	0.818	0.475	1.23E-01
APEX2	6.71E-11	0.167	0.250	0.060	2.16E-06	5.79E-159	0.218	0.319	0.060	1.86E-154	1.57E-10	0.173	0.333	0.060	5.05E-06
ARHGDIA	3.70E-09	0.438	0.667	0.365	1.19E-04	4.12E-103	0.334	0.707	0.365	1.33E-98	3.14E-03	0.124	0.636	0.365	1.00E+00
ARHGDIB	6.22E-10	0.271	0.444	0.159	2.00E-05	1.08E-139	0.404	0.481	0.159	3.48E-135	1.62E-08	0.287	0.545	0.159	5.23E-04
ARL2	2.58E-05	0.418	0.431	0.227	8.31E-01	1.24E-141	0.364	0.596	0.227	3.98E-137	2.23E-09	0.511	0.667	0.227	7.17E-05
ARPC1B	1.06E-16	0.708	0.486	0.147	3.41E-12	5.64E-287	1.060	0.589	0.147	1.82E-282	7.20E-04	0.163	0.364	0.147	1.00E+00
ARPC3	2.40E-05	0.376	0.778	0.579	7.73E-01	1.34E-178	0.721	0.860	0.579	4.32E-174	1.31E-03	0.615	0.788	0.579	1.00E+00
ARPC4	1.21E-07	0.251	0.514	0.239	3.91E-03	5.45E-216	0.547	0.672	0.239	1.75E-211	1.34E-06	0.643	0.606	0.239	4.30E-02
ATOX1	2.40E-07	0.287	0.597	0.296	7.73E-03	0.00E+00	1.084	0.799	0.296	0.00E+00	9.93E-05	0.841	0.576	0.296	1.00E+00
ATP5F1D	1.29E-03	0.269	0.750	0.637	1.00E+00	1.85E-293	1.062	0.921	0.637	5.95E-289	3.10E-08	0.910	0.879	0.637	9.97E-04
ATP5PD	2.22E-04	0.279	0.736	0.577	1.00E+00	1.12E-189	0.744	0.878	0.577	3.59E-185	1.65E-03	0.313	0.758	0.577	1.00E+00
BANF1	1.53E-03	0.109	0.597	0.399	1.00E+00	8.32E-62	0.226	0.681	0.399	2.68E-57	3.89E-03	0.220	0.636	0.399	1.00E+00
BSG	1.65E-04	0.325	0.681	0.492	1.00E+00	1.46E-57	0.240	0.763	0.492	4.69E-53	7.16E-03	0.311	0.667	0.492	1.00E+00
C10orf99	9.88E-55	1.517	0.389	0.035	3.18E-50	0.00E+00	2.586	0.630	0.035	0.00E+00	9.97E-44	2.021	0.485	0.035	3.21E-39
C1R	1.73E-64	0.929	0.569	0.063	5.57E-60	1.18E-131	0.340	0.297	0.063	3.82E-127	4.79E-05	0.301	0.242	0.063	1.00E+00
CALML3	1.56E-07	0.699	0.597	0.328	5.02E-03	2.52E-299	2.221	0.806	0.328	8.12E-295	1.51E-11	1.911	0.727	0.328	4.85E-07
CAPG	1.66E-06	0.633	0.653	0.453	5.34E-02	1.28E-228	0.957	0.821	0.453	4.13E-224	8.19E-06	0.650	0.788	0.453	2.64E-01
CAPN1	3.76E-13	0.619	0.750	0.386	1.21E-08	7.15E-186	0.596	0.796	0.386	2.30E-181	8.94E-04	0.212	0.667	0.386	1.00E+00
CAPNS1	6.42E-12	0.184	0.333	0.091	2.07E-07	1.72E-192	0.355	0.421	0.091	5.54E-188	3.67E-15	0.769	0.485	0.091	1.18E-10
CARHSP1	3.42E-19	0.861	0.583	0.200	1.10E-14	1.45E-275	0.822	0.667	0.200	4.67E-271	1.15E-09	0.968	0.636	0.200	3.69E-05
CBLC	7.19E-11	0.411	0.458	0.168	2.31E-06	3.10E-293	0.590	0.649	0.168	9.98E-289	1.03E-06	0.294	0.515	0.168	3.31E-02
CCDC124	1.43E-06	0.270	0.528	0.263	4.61E-02	0.00E+00	0.844	0.762	0.263	0.00E+00	5.57E-08	0.534	0.667	0.263	1.79E-03
CD47	4.64E-16	0.700	0.681	0.296	1.49E-11	6.72E-306	0.968	0.778	0.296	2.16E-301	2.81E-04	0.315	0.576	0.296	1.00E+00
CD74	4.99E-41	2.515	0.528	0.087	1.61E-36	0.00E+00	2.643	0.726	0.087	0.00E+00	9.81E-06	1.975	0.303	0.087	3.16E-01
CD82	1.13E-03	0.180	0.528	0.354	1.00E+00	2.63E-206	0.727	0.761	0.354	8.46E-202	1.37E-03	0.201	0.576	0.354	1.00E+00
CDC42EP1	1.29E-09	0.504	0.514	0.233	4.15E-05	9.26E-140	0.495	0.585	0.233	2.98E-135	1.76E-06	0.671	0.576	0.233	5.68E-02
CDH3	6.45E-46	1.247	0.722	0.149	2.08E-41	0.00E+00	1.018	0.683	0.149	0.00E+00	1.94E-05	0.245	0.424	0.149	6.26E-01
CIB1	1.41E-07	0.261	0.458	0.208	4.54E-03	3.97E-97	0.256	0.508	0.208	1.28E-92	5.43E-06	0.208	0.545	0.208	1.75E-01
CISD2	1.79E-07	0.233	0.472	0.206	5.76E-03	7.77E-64	0.155	0.452	0.206	2.50E-59	2.25E-04	0.464	0.515	0.206	1.00E+00
CISD3	1.50E-08	0.173	0.361	0.127	4.83E-04	2.45E-116	0.224	0.408	0.127	7.91E-112	7.40E-12	0.412	0.515	0.127	2.38E-07
CLCA2	2.31E-14	1.174	0.611	0.297	7.44E-10	8.25E-157	0.530	0.704	0.297	2.66E-152	9.10E-08	0.521	0.697	0.297	2.93E-03
CLEC7A	9.82E-35	0.227	0.194	0.013	3.16E-30	0.00E+00	0.440	0.400	0.013	0.00E+00	5.32E-51	0.379	0.333	0.013	1.71E-46
CMAS	5.41E-05	0.135	0.347	0.157	1.00E+00	2.88E-135	0.293	0.478	0.157	9.27E-131	8.72E-05	0.103	0.424	0.157	1.00E+00
CNDP2	8.59E-16	0.344	0.403	0.103	2.77E-11	1.52E-107	0.213	0.353	0.103	4.90E-103	2.36E-12	0.368	0.485	0.103	7.61E-08
CNIH4	7.84E-05	0.296	0.528	0.324	1.00E+00	1.46E-56	0.141	0.599	0.324	4.69E-52	2.91E-04	0.241	0.636	0.324	1.00E+00
CNN2	6.72E-11	0.596	0.611	0.303	2.17E-06	2.71E-195	0.621	0.724	0.303	8.74E-191	1.70E-02	0.357	0.545	0.303	1.00E+00
CNP	3.03E-03	0.167	0.264	0.129	1.00E+00	5.79E-141	0.260	0.441	0.129	1.87E-136	1.48E-03	0.139	0.333	0.129	1.00E+00
COA3	5.02E-09	0.312	0.514	0.214	1.62E-04	6.05E-256	0.603	0.676	0.214	1.95E-251	5.91E-10	0.719	0.667	0.214	1.90E-05
COMTD1	2.25E-14	0.465	0.597	0.229	7.26E-10	0.00E+00	0.954	0.763	0.229	0.00E+00	1.46E-09	0.584	0.667	0.229	4.71E-05
COPE	2.12E-02	0.355	0.611	0.464	1.00E+00	1.31E-135	0.519	0.788	0.464	4.22E-131	1.88E-05	0.381	0.788	0.464	6.06E-01
COQ4	6.82E-09	0.408	0.569	0.265	2.20E-04	7.93E-193	0.512	0.679	0.265	2.55E-188	2.95E-07	0.417	0.667	0.265	9.49E-03
CORO1B	1.09E-08	0.245	0.458	0.183	3.50E-04	2.59E-168	0.406	0.556	0.183	8.33E-164	5.50E-04	0.129	0.424	0.183	1.00E+00
COX16	6.67E-05	0.269	0.597	0.361	1.00E+00	2.11E-57	0.162	0.641	0.361	6.81E-53	7.39E-05	0.629	0.667	0.361	1.00E+00
CST3	3.12E-15	1.482	0.792	0.491	1.01E-10	2.17E-232	1.316	0.848	0.491	6.97E-228	3.25E-17	1.992	0.970	0.491	1.05E-12
CTSA	9.08E-10	0.191	0.472	0.183	2.92E-05	3.22E-260	0.638	0.642	0.183	1.04E-255	3.13E-05	0.145	0.485	0.183	1.00E+00
CTSB	1.62E-26	1.747	0.736	0.259	5.21E-22	1.66E-298	0.893	0.755	0.259	5.35E-294	7.51E-10	0.465	0.727	0.259	2.42E-05
CTSC	6.61E-11	0.421	0.514	0.204	2.13E-06	1.65E-231	0.688	0.643	0.204	5.32E-227	8.76E-10	0.848	0.606	0.204	2.82E-05
CTSD	1.71E-10	0.799	0.708	0.424	5.51E-06	0.00E+00	1.329	0.882	0.424	0.00E+00	5.87E-07	0.832	0.758	0.424	1.89E-02
CYBC1	6.46E-25	0.469	0.347	0.057	2.08E-20	4.02E-243	0.329	0.390	0.057	1.29E-238	2.67E-07	0.108	0.273	0.057	8.60E-03

DBNL	1.44E-18	0.659	0.514	0.146	4.64E-14	0.00E+00	0.770	0.708	0.146	0.00E+00	2.66E-07	0.166	0.485	0.146	8.56E-03
DCTD	2.99E-09	0.160	0.375	0.129	9.64E-05	3.23E-256	0.500	0.549	0.129	1.04E-251	2.65E-06	0.124	0.424	0.129	8.54E-02
DNAJC15	1.10E-07	0.252	0.500	0.221	3.54E-03	6.21E-247	0.609	0.683	0.221	2.00E-242	6.21E-14	0.535	0.758	0.221	2.00E-09
EIF4EBP1	3.59E-10	0.408	0.361	0.118	1.16E-05	9.79E-286	0.564	0.553	0.118	3.15E-281	5.27E-29	1.090	0.727	0.118	1.70E-24
ELOVL1	2.38E-12	0.382	0.528	0.198	7.67E-08	0.00E+00	0.842	0.708	0.198	0.00E+00	1.11E-05	0.152	0.515	0.198	3.58E-01
ENO1	1.03E-09	0.909	0.833	0.689	3.32E-05	1.14E-162	0.744	0.919	0.689	3.67E-158	9.93E-07	0.988	0.879	0.689	3.20E-02
EPHX3	2.45E-02	0.144	0.236	0.142	1.00E+00	0.00E+00	1.411	0.724	0.142	0.00E+00	9.00E-03	0.172	0.303	0.142	1.00E+00
ETHE1	3.77E-24	0.600	0.514	0.122	1.21E-19	0.00E+00	0.982	0.576	0.122	0.00E+00	1.37E-09	0.312	0.485	0.122	4.40E-05
ETV7	1.77E-19	0.142	0.208	0.027	5.70E-15	0.00E+00	0.545	0.466	0.027	0.00E+00	1.73E-05	0.104	0.152	0.027	5.57E-01
FABP5	7.39E-08	2.339	0.653	0.449	2.38E-03	0.00E+00	3.741	0.889	0.449	0.00E+00	1.26E-08	3.716	0.788	0.449	4.05E-04
FADD	9.48E-09	0.140	0.194	0.046	3.05E-04	2.29E-238	0.276	0.359	0.046	7.39E-234	1.05E-19	0.223	0.394	0.046	3.39E-15
FAM207A	3.25E-13	0.273	0.375	0.100	1.05E-08	1.40E-116	0.197	0.359	0.100	4.52E-112	3.32E-10	0.528	0.455	0.100	1.07E-05
FAM83H	1.22E-09	0.272	0.375	0.129	3.94E-05	2.75E-52	0.163	0.310	0.129	8.84E-48	1.18E-06	0.245	0.424	0.129	3.79E-02
FARSA	2.00E-07	0.243	0.389	0.157	6.43E-03	1.67E-143	0.278	0.491	0.157	5.39E-139	6.69E-06	0.316	0.455	0.157	2.15E-01
FASTK	4.57E-05	0.325	0.361	0.174	1.00E+00	2.61E-97	0.178	0.463	0.174	8.40E-93	1.10E-08	0.227	0.576	0.174	3.53E-04
FKBP1A	1.98E-07	0.631	0.722	0.497	6.38E-03	2.09E-173	0.748	0.812	0.497	6.72E-169	7.70E-03	0.355	0.727	0.497	1.00E+00
FKBP8	9.69E-10	0.620	0.639	0.356	3.12E-05	3.11E-124	0.438	0.736	0.356	1.00E-119	1.32E-03	0.214	0.606	0.356	1.00E+00
GADD45GIP1	1.65E-02	0.207	0.542	0.421	1.00E+00	1.14E-146	0.568	0.774	0.421	3.67E-142	5.48E-05	0.460	0.727	0.421	1.00E+00
GAPDH	1.02E-14	1.383	0.944	0.872	3.30E-10	1.62E-242	1.035	0.984	0.872	5.23E-238	9.54E-11	1.453	0.970	0.872	3.07E-06
GAS2L1	4.10E-07	0.216	0.458	0.207	1.32E-02	1.13E-71	0.209	0.463	0.207	3.64E-67	2.53E-03	0.130	0.455	0.207	1.00E+00
GATB	2.23E-20	0.199	0.375	0.074	7.18E-16	0.00E+00	0.624	0.556	0.074	0.00E+00	1.08E-11	0.204	0.394	0.074	3.49E-07
GET3	1.46E-02	0.149	0.444	0.302	1.00E+00	2.67E-152	0.474	0.678	0.302	8.60E-148	7.56E-06	0.762	0.697	0.302	2.43E-01
GGCT	5.37E-03	0.284	0.486	0.320	1.00E+00	0.00E+00	1.128	0.812	0.320	0.00E+00	2.19E-04	0.438	0.667	0.320	1.00E+00
GJB2	3.93E-52	3.039	0.833	0.205	1.26E-47	5.57E-188	1.333	0.603	0.205	1.79E-183	1.61E-15	1.080	0.758	0.205	5.17E-11
GM2A	1.07E-10	0.490	0.500	0.190	3.44E-06	0.00E+00	0.740	0.709	0.190	0.00E+00	1.47E-08	0.307	0.606	0.190	4.74E-04
GNA15	4.64E-08	0.279	0.514	0.246	1.49E-03	3.24E-151	0.377	0.621	0.246	1.05E-146	3.68E-03	0.129	0.485	0.246	1.00E+00
GRN	2.06E-11	1.075	0.736	0.462	6.64E-07	1.96E-270	1.002	0.861	0.462	6.32E-266	8.87E-06	0.615	0.788	0.462	2.86E-01
GSTO1	6.34E-04	0.199	0.472	0.282	1.00E+00	7.22E-61	0.252	0.540	0.282	2.33E-56	1.65E-03	0.243	0.545	0.282	1.00E+00
GSTP1	1.20E-04	0.511	0.847	0.771	1.00E+00	0.00E+00	1.555	0.960	0.771	0.00E+00	2.03E-14	2.263	0.939	0.771	6.55E-10
GTF3C6	9.61E-10	0.364	0.556	0.247	3.10E-05	1.03E-141	0.378	0.624	0.247	3.31E-137	1.07E-06	0.701	0.636	0.247	3.46E-02
H2AFY	8.81E-07	0.441	0.639	0.400	2.84E-02	2.57E-220	0.720	0.812	0.400	8.28E-216	4.26E-04	0.345	0.667	0.400	1.00E+00
HCP5	3.98E-04	0.168	0.292	0.140	1.00E+00	1.42E-137	0.280	0.459	0.140	4.59E-133	9.95E-03	0.280	0.303	0.140	1.00E+00
HM13	5.04E-19	0.644	0.583	0.178	1.62E-14	0.00E+00	0.742	0.697	0.178	0.00E+00	2.05E-05	0.873	0.455	0.178	6.61E-01
HMGN1	1.39E-02	0.258	0.736	0.617	1.00E+00	1.95E-25	0.108	0.805	0.617	6.28E-21	1.35E-02	0.406	0.848	0.617	1.00E+00
HSBP1	1.14E-03	0.288	0.639	0.478	1.00E+00	4.24E-48	0.209	0.745	0.478	1.37E-43	6.36E-04	0.522	0.788	0.478	1.00E+00
IDH2	1.89E-10	0.424	0.597	0.279	6.07E-06	0.00E+00	1.041	0.805	0.279	0.00E+00	4.20E-05	0.160	0.606	0.279	1.00E+00
IFI27	2.52E-74	3.549	0.792	0.130	8.13E-70	0.00E+00	3.787	0.939	0.130	0.00E+00	9.65E-52	3.484	0.939	0.130	3.11E-47
IFI27L2	2.79E-07	0.305	0.500	0.240	8.98E-03	8.28E-304	0.844	0.743	0.240	2.67E-299	4.32E-06	0.324	0.576	0.240	1.39E-01
IFI6	6.00E-173	1.649	0.611	0.026	1.93E-168	1.04E-272	0.731	0.316	0.026	3.35E-268	5.83E-04	0.259	0.121	0.026	1.00E+00
IFITM1	6.15E-120	3.679	0.792	0.075	1.98E-115	3.76E-162	1.116	0.343	0.075	1.21E-157	7.91E-34	1.712	0.636	0.075	2.55E-29
IFITM3	1.41E-31	3.265	0.833	0.410	4.55E-27	1.37E-219	2.036	0.771	0.410	4.43E-215	5.61E-12	1.714	0.879	0.410	1.81E-07
IL4R	3.32E-48	0.740	0.514	0.068	1.07E-43	8.20E-148	0.277	0.326	0.068	2.64E-143	3.27E-07	0.137	0.303	0.068	1.05E-02
IMP3	1.15E-03	0.227	0.556	0.358	1.00E+00	4.11E-249	0.813	0.799	0.358	1.32E-244	3.22E-07	0.547	0.788	0.358	1.04E-02
IMPA2	4.19E-08	0.650	0.653	0.407	1.35E-03	2.01E-100	0.422	0.743	0.407	6.48E-96	5.12E-18	1.643	0.909	0.407	1.65E-13
ISOC2	3.29E-02	0.130	0.389	0.274	1.00E+00	9.70E-97	0.340	0.578	0.274	3.12E-92	1.64E-04	0.232	0.576	0.274	1.00E+00
ITPA	3.99E-04	0.321	0.458	0.258	1.00E+00	1.92E-53	0.116	0.502	0.258	6.18E-49	5.16E-07	0.271	0.667	0.258	1.66E-02
JOSD2	6.39E-08	0.169	0.444	0.183	2.06E-03	1.72E-208	0.483	0.592	0.183	5.55E-204	4.68E-12	0.788	0.636	0.183	1.51E-07
KCTD1	5.80E-05	0.108	0.500	0.259	1.00E+00	2.97E-84	0.264	0.563	0.259	9.57E-80	2.92E-03	0.413	0.515	0.259	1.00E+00
KRT14	3.17E-16	1.406	0.931	0.769	1.02E-11	2.41E-39	0.842	0.796	0.769	7.76E-35	3.09E-09	1.320	1.000	0.769	9.95E-05
KRT16	1.05E-25	4.780	0.750	0.305	3.38E-21	0.00E+00	4.905	0.917	0.305	0.00E+00	8.35E-19	4.108	0.848	0.305	2.69E-14
KRT5	6.59E-09	1.217	0.847	0.704	2.12E-04	4.14E-35	0.706	0.765	0.704	1.33E-30	2.72E-11	1.740	0.970	0.704	8.75E-07

KRT6A	2.60E-89	5.141	0.917	0.148	8.39E-85	0.00E+00	4.411	0.970	0.148	0.00E+00	9.15E-49	4.611	0.970	0.148	2.95E-44
KRT6B	1.07E-34	3.531	0.528	0.101	3.43E-30	0.00E+00	2.570	0.756	0.101	0.00E+00	3.13E-33	3.131	0.727	0.101	1.01E-28
KRT6C	1.96E-217	5.047	0.569	0.016	6.32E-213	0.00E+00	3.744	0.749	0.016	0.00E+00	2.05E-158	4.551	0.667	0.016	6.61E-154
LAD1	2.72E-08	0.731	0.625	0.372	8.78E-04	1.77E-300	1.062	0.844	0.372	5.69E-296	2.10E-08	0.855	0.758	0.372	6.77E-04
LAMTOR1	1.38E-03	0.132	0.500	0.308	1.00E+00	5.97E-215	0.654	0.736	0.308	1.92E-210	4.55E-06	0.360	0.667	0.308	1.47E-01
LGALS3BP	8.41E-31	0.933	0.528	0.110	2.71E-26	0.00E+00	0.702	0.563	0.110	0.00E+00	1.27E-15	0.584	0.545	0.110	4.08E-11
LMAN2	4.46E-07	0.474	0.597	0.336	1.44E-02	2.19E-92	0.317	0.656	0.336	7.07E-88	1.28E-03	0.154	0.636	0.336	1.00E+00
LSM7	1.13E-03	0.261	0.639	0.471	1.00E+00	1.65E-38	0.185	0.717	0.471	5.32E-34	1.53E-03	0.304	0.697	0.471	1.00E+00
LTB4R2	1.27E-21	0.672	0.556	0.156	4.10E-17	0.00E+00	0.841	0.694	0.156	0.00E+00	6.21E-09	0.245	0.545	0.156	2.00E-04
LYPLA1	3.83E-05	0.181	0.458	0.235	1.00E+00	5.66E-108	0.273	0.560	0.235	1.82E-103	7.79E-04	0.513	0.515	0.235	1.00E+00
MALL	4.69E-43	0.793	0.514	0.076	1.51E-38	2.37E-161	0.383	0.357	0.076	7.64E-157	1.52E-06	0.553	0.303	0.076	4.89E-02
MAPKAP1	5.99E-12	0.442	0.389	0.115	1.93E-07	6.75E-91	0.186	0.354	0.115	2.17E-86	3.99E-06	0.154	0.394	0.115	1.28E-01
MDFI	2.19E-31	0.463	0.444	0.073	7.05E-27	7.01E-170	0.304	0.361	0.073	2.26E-165	2.06E-16	0.741	0.455	0.073	6.62E-12
MIB2	1.27E-06	0.431	0.667	0.385	4.10E-02	5.77E-116	0.437	0.724	0.385	1.86E-111	4.25E-04	0.184	0.697	0.385	1.00E+00
MICALL1	1.49E-10	0.224	0.278	0.075	4.80E-06	6.72E-108	0.263	0.294	0.075	2.16E-103	8.32E-33	0.475	0.636	0.075	2.68E-28
MICOS13	6.35E-03	0.201	0.556	0.394	1.00E+00	5.22E-81	0.353	0.707	0.394	1.68E-76	3.29E-05	0.472	0.697	0.394	1.00E+00
MIF	4.91E-09	1.008	0.903	0.803	1.58E-04	3.32E-90	0.543	0.953	0.803	1.07E-85	9.33E-06	0.970	0.939	0.803	3.00E-01
MPHOSPH6	7.82E-11	0.299	0.472	0.172	2.52E-06	7.09E-194	0.462	0.570	0.172	2.28E-189	7.63E-06	0.190	0.485	0.172	2.46E-01
MRPL12	7.94E-14	0.501	0.542	0.197	2.56E-09	0.00E+00	0.800	0.696	0.197	0.00E+00	2.64E-10	0.389	0.636	0.197	8.51E-06
MRPL20	4.43E-06	0.218	0.667	0.380	1.43E-01	5.05E-134	0.432	0.737	0.380	1.63E-129	3.41E-06	0.596	0.758	0.380	1.10E-01
MRPL27	2.91E-04	0.183	0.500	0.296	1.00E+00	1.03E-216	0.600	0.733	0.296	3.31E-212	1.14E-04	0.440	0.606	0.296	1.00E+00
MRPL52	3.21E-03	0.159	0.597	0.414	1.00E+00	8.25E-40	0.134	0.663	0.414	2.66E-35	4.62E-05	0.450	0.697	0.414	1.00E+00
MRPL54	1.53E-04	0.272	0.500	0.304	1.00E+00	2.17E-108	0.369	0.640	0.304	7.00E-104	4.62E-04	0.545	0.606	0.304	1.00E+00
MRPL57	6.41E-05	0.285	0.611	0.371	1.00E+00	8.17E-125	0.412	0.738	0.371	2.63E-120	4.90E-05	0.677	0.697	0.371	1.00E+00
MRPS34	2.68E-07	0.424	0.639	0.382	8.63E-03	3.65E-192	0.688	0.779	0.382	1.18E-187	9.29E-04	0.364	0.606	0.382	1.00E+00
MRPS7	2.03E-06	0.239	0.514	0.247	6.55E-02	2.01E-147	0.402	0.624	0.247	6.48E-143	7.29E-06	0.207	0.606	0.247	2.35E-01
MTG1	3.97E-15	0.332	0.486	0.141	1.28E-10	2.82E-161	0.268	0.491	0.141	9.07E-157	8.68E-07	0.234	0.455	0.141	2.80E-02
MVP	5.27E-21	0.563	0.583	0.171	1.70E-16	1.47E-203	0.473	0.573	0.171	4.75E-199	1.52E-09	0.365	0.576	0.171	4.90E-05
MX1	3.43E-141	1.056	0.542	0.025	1.11E-136	0.00E+00	0.569	0.350	0.025	0.00E+00	1.92E-11	0.188	0.212	0.025	6.17E-07
NAPRT	3.09E-05	0.158	0.431	0.210	9.96E-01	8.05E-134	0.317	0.560	0.210	2.59E-129	1.03E-06	0.315	0.576	0.210	3.32E-02
NCLN	3.83E-14	0.188	0.319	0.075	1.23E-09	2.37E-100	0.167	0.288	0.075	7.64E-96	9.54E-07	0.166	0.303	0.075	3.07E-02
NDUFS8	5.66E-04	0.119	0.597	0.377	1.00E+00	3.52E-206	0.677	0.783	0.377	1.13E-201	5.71E-05	0.317	0.727	0.377	1.00E+00
NME2	2.40E-02	0.245	0.833	0.773	1.00E+00	1.06E-177	0.813	0.939	0.773	3.41E-173	4.29E-08	1.042	0.909	0.773	1.38E-03
NMI	5.33E-18	0.391	0.417	0.103	1.72E-13	3.39E-235	0.387	0.488	0.103	1.09E-230	9.63E-08	0.585	0.394	0.103	3.10E-03
NOP10	7.74E-07	0.413	0.708	0.452	2.49E-02	2.96E-46	0.216	0.712	0.452	9.55E-42	1.80E-02	0.108	0.667	0.452	1.00E+00
NT5C	7.06E-08	0.240	0.542	0.252	2.27E-03	1.35E-141	0.331	0.627	0.252	4.36E-137	2.70E-08	0.357	0.667	0.252	8.71E-04
NUCB1	2.88E-13	0.511	0.583	0.236	9.26E-09	2.32E-128	0.347	0.587	0.236	7.47E-124	5.16E-06	0.261	0.606	0.236	1.66E-01
NUDT22	2.86E-07	0.122	0.347	0.130	9.22E-03	4.72E-232	0.402	0.540	0.130	1.52E-227	4.00E-11	0.472	0.545	0.130	1.29E-06
NUTF2	2.24E-04	0.351	0.417	0.220	1.00E+00	1.63E-187	0.476	0.629	0.220	5.26E-183	2.21E-04	0.144	0.515	0.220	1.00E+00
OAS1	1.70E-90	0.864	0.514	0.037	5.46E-86	0.00E+00	0.475	0.390	0.037	0.00E+00	1.85E-12	0.297	0.273	0.037	5.96E-08
OAS3	9.26E-47	0.253	0.347	0.031	2.98E-42	3.01E-160	0.220	0.244	0.031	9.71E-156	1.13E-06	0.299	0.182	0.031	3.63E-02
ODF3B	6.17E-63	1.070	0.667	0.096	1.99E-58	1.98E-245	0.445	0.482	0.096	6.38E-241	2.51E-11	0.616	0.455	0.096	8.08E-07
ORMDL2	7.88E-04	0.116	0.347	0.174	1.00E+00	1.41E-231	0.475	0.611	0.174	4.56E-227	1.53E-04	0.207	0.424	0.174	1.00E+00
P4HB	3.66E-10	0.731	0.792	0.585	1.18E-05	1.13E-273	1.041	0.908	0.585	3.65E-269	3.40E-06	0.706	0.879	0.585	1.10E-01
PARP14	8.37E-12	0.165	0.236	0.051	2.70E-07	3.00E-86	0.205	0.219	0.051	9.68E-82	7.41E-04	0.227	0.182	0.051	1.00E+00
PARP9	2.28E-68	0.680	0.472	0.041	7.33E-64	8.26E-302	0.410	0.391	0.041	2.66E-297	8.85E-07	0.580	0.212	0.041	2.85E-02
PCYT2	7.44E-04	0.103	0.125	0.043	1.00E+00	5.32E-71	0.111	0.183	0.043	1.71E-66	3.61E-10	0.106	0.273	0.043	1.16E-05
PDAP1	1.51E-04	0.348	0.583	0.354	1.00E+00	2.73E-158	0.555	0.740	0.354	8.78E-154	1.73E-08	0.544	0.788	0.354	5.56E-04
PDCD5	1.08E-04	0.399	0.667	0.463	1.00E+00	5.44E-90	0.432	0.777	0.463	1.75E-85	7.80E-05	0.366	0.758	0.463	1.00E+00
PDLIM1	1.45E-04	0.561	0.653	0.469	1.00E+00	5.37E-37	0.196	0.726	0.469	1.73E-32	1.22E-07	0.863	0.879	0.469	3.94E-03
PDPN	4.09E-16	0.255	0.208	0.032	1.32E-11	6.12E-69	0.268	0.154	0.032	1.97E-64	1.94E-06	0.148	0.182	0.032	6.25E-02

PDZK1IP1	4.63E-18	0.681	0.514	0.162	1.49E-13	0.00E+00	2.406	0.832	0.162	0.00E+00	2.06E-09	0.709	0.545	0.162	6.62E-05
PGAM1	5.48E-09	0.907	0.722	0.511	1.77E-04	6.71E-269	1.107	0.851	0.511	2.16E-264	3.72E-06	0.921	0.758	0.511	1.20E-01
PGM2	5.33E-06	0.297	0.389	0.178	1.72E-01	3.70E-250	0.585	0.624	0.178	1.19E-245	4.70E-04	0.868	0.424	0.178	1.00E+00
PKM	7.59E-19	1.344	0.958	0.699	2.45E-14	7.58E-224	1.013	0.942	0.699	2.44E-219	6.61E-07	0.921	0.909	0.699	2.13E-02
PKP3	1.39E-07	0.616	0.764	0.543	4.48E-03	1.71E-192	0.785	0.874	0.543	5.51E-188	8.02E-03	0.195	0.727	0.543	1.00E+00
PLAAT4	6.12E-12	0.256	0.208	0.041	1.97E-07	1.98E-133	0.269	0.245	0.041	6.38E-129	2.48E-02	0.109	0.121	0.041	1.00E+00
PLBD1	1.60E-11	0.465	0.333	0.099	5.16E-07	0.00E+00	0.644	0.559	0.099	0.00E+00	8.68E-10	0.258	0.424	0.099	2.79E-05
PLEKHJ1	1.75E-07	0.258	0.417	0.169	5.65E-03	1.42E-241	0.492	0.616	0.169	4.58E-237	3.69E-07	0.249	0.515	0.169	1.19E-02
PLSCR1	2.55E-27	0.405	0.458	0.087	8.20E-23	7.18E-131	0.292	0.347	0.087	2.31E-126	4.69E-09	0.182	0.394	0.087	1.51E-04
PPIB	1.06E-07	0.575	0.778	0.563	3.41E-03	1.90E-283	1.070	0.904	0.563	6.13E-279	2.73E-08	0.845	0.879	0.563	8.80E-04
PPP1CA	4.47E-07	0.418	0.694	0.416	1.44E-02	7.53E-151	0.569	0.767	0.416	2.43E-146	1.28E-05	0.646	0.758	0.416	4.11E-01
PRR13	2.67E-05	0.138	0.458	0.231	8.61E-01	1.01E-232	0.614	0.683	0.231	3.27E-228	2.78E-07	0.221	0.636	0.231	8.97E-03
PSMA7	2.42E-02	0.126	0.764	0.624	1.00E+00	6.72E-95	0.478	0.860	0.624	2.16E-90	3.91E-03	0.365	0.758	0.624	1.00E+00
PSMB10	7.71E-21	0.503	0.486	0.124	2.48E-16	1.19E-222	0.571	0.509	0.124	3.83E-218	2.05E-15	1.024	0.576	0.124	6.60E-11
PSMB8	1.60E-07	0.515	0.583	0.344	5.15E-03	4.33E-214	0.827	0.763	0.344	1.40E-209	5.08E-03	0.392	0.636	0.344	1.00E+00
PSMD13	1.83E-06	0.226	0.514	0.257	5.89E-02	3.02E-73	0.200	0.538	0.257	9.72E-69	2.08E-05	0.228	0.606	0.257	6.69E-01
PSME1	2.41E-05	0.713	0.653	0.520	7.77E-01	1.04E-251	1.104	0.864	0.520	3.35E-247	1.70E-02	0.390	0.727	0.520	1.00E+00
PSME2	2.71E-08	0.766	0.681	0.457	8.74E-04	0.00E+00	1.351	0.864	0.457	0.00E+00	3.67E-05	0.389	0.788	0.457	1.00E+00
PTPMT1	5.01E-15	0.395	0.444	0.125	1.61E-10	4.05E-198	0.364	0.496	0.125	1.30E-193	2.00E-09	0.640	0.485	0.125	6.44E-05
PYCARD	1.53E-04	0.278	0.639	0.426	1.00E+00	0.00E+00	1.838	0.911	0.426	0.00E+00	6.70E-06	1.221	0.697	0.426	2.16E-01
PYM1	1.34E-07	0.209	0.403	0.155	4.33E-03	4.00E-250	0.501	0.597	0.155	1.29E-245	1.15E-07	0.170	0.515	0.155	3.69E-03
R3HDM4	2.20E-20	0.822	0.569	0.171	7.09E-16	2.11E-161	0.362	0.536	0.171	6.78E-157	1.83E-12	0.429	0.636	0.171	5.90E-08
RAB25	1.16E-02	0.215	0.611	0.424	1.00E+00	6.56E-273	1.197	0.823	0.424	2.11E-268	7.19E-06	0.903	0.758	0.424	2.32E-01
RAB38	4.03E-12	0.624	0.611	0.271	1.30E-07	1.16E-235	0.680	0.722	0.271	3.75E-231	4.63E-05	0.675	0.545	0.271	1.00E+00
RAN	1.62E-02	0.306	0.778	0.688	1.00E+00	7.71E-78	0.459	0.893	0.688	2.48E-73	3.62E-03	0.486	0.848	0.688	1.00E+00
RBCK1	1.97E-10	0.662	0.556	0.236	6.33E-06	1.12E-128	0.328	0.592	0.236	3.61E-124	8.12E-05	0.231	0.545	0.236	1.00E+00
RNASEK	9.55E-03	0.226	0.806	0.643	1.00E+00	6.92E-202	0.756	0.902	0.643	2.23E-197	7.22E-03	0.224	0.818	0.643	1.00E+00
RPL22L1	6.58E-08	0.744	0.792	0.578	2.12E-03	0.00E+00	1.368	0.898	0.578	0.00E+00	8.79E-06	0.763	0.848	0.578	2.83E-01
RPL28	1.02E-04	0.375	0.986	0.926	1.00E+00	0.00E+00	1.085	0.993	0.926	0.00E+00	2.57E-06	0.613	1.000	0.926	8.27E-02
S100A10	1.30E-08	1.215	0.833	0.763	4.20E-04	1.83E-30	0.658	0.882	0.763	5.88E-26	6.05E-12	1.410	0.970	0.763	1.95E-07
S100A11	3.46E-09	0.753	0.903	0.858	1.11E-04	0.00E+00	1.257	0.985	0.858	0.00E+00	1.60E-04	0.679	0.970	0.858	1.00E+00
S100A16	4.00E-03	0.334	0.778	0.672	1.00E+00	0.00E+00	1.471	0.940	0.672	0.00E+00	1.96E-08	1.216	0.879	0.672	6.30E-04
S100A7	6.71E-88	4.385	0.569	0.048	2.16E-83	0.00E+00	3.958	0.535	0.048	0.00E+00	1.54E-35	3.387	0.515	0.048	4.96E-31
S100A8	1.06E-120	6.082	0.861	0.093	3.42E-116	0.00E+00	5.925	0.959	0.093	0.00E+00	1.48E-59	5.365	0.879	0.093	4.76E-55
S100A9	4.60E-95	5.032	0.778	0.094	1.48E-90	0.00E+00	5.917	0.944	0.094	0.00E+00	1.61E-39	4.142	0.727	0.094	5.19E-35
SELENOW	2.96E-04	0.339	0.778	0.613	1.00E+00	2.16E-163	0.745	0.892	0.613	6.96E-159	5.04E-03	0.416	0.788	0.613	1.00E+00
SERF2	1.03E-08	0.725	0.903	0.797	3.33E-04	8.96E-31	0.214	0.935	0.797	2.89E-26	1.13E-03	0.516	0.879	0.797	1.00E+00
SERINC2	8.16E-14	0.448	0.431	0.126	2.63E-09	1.09E-59	0.166	0.319	0.126	3.51E-55	3.07E-11	0.361	0.515	0.126	9.90E-07
SERPINB1	5.65E-22	0.633	0.528	0.143	1.82E-17	1.45E-238	0.792	0.549	0.143	4.67E-234	6.22E-14	0.360	0.606	0.143	2.00E-09
SERPINB3	2.72E-260	1.359	0.486	0.008	8.77E-256	0.00E+00	2.158	0.689	0.008	0.00E+00	6.41E-34	0.138	0.212	0.008	2.07E-29
SERPINB4	1.55E-194	1.425	0.389	0.007	5.01E-190	0.00E+00	2.858	0.806	0.007	0.00E+00	2.13E-28	0.415	0.182	0.007	6.87E-24
SERPINB6	2.62E-19	0.549	0.333	0.066	8.45E-15	0.00E+00	0.509	0.526	0.066	0.00E+00	8.45E-08	0.147	0.303	0.066	2.72E-03
SH3BGRL3	3.74E-12	1.004	0.847	0.675	1.20E-07	0.00E+00	1.389	0.947	0.675	0.00E+00	4.45E-05	0.720	0.758	0.675	1.00E+00
SHISA5	6.23E-21	0.754	0.528	0.147	2.01E-16	7.21E-169	0.396	0.500	0.147	2.32E-164	1.88E-04	0.111	0.394	0.147	1.00E+00
SLC27A4	5.56E-13	0.275	0.250	0.054	1.79E-08	5.42E-229	0.366	0.367	0.054	1.75E-224	9.08E-15	0.273	0.364	0.054	2.92E-10
SLC50A1	2.98E-10	0.185	0.361	0.114	9.58E-06	6.74E-211	0.360	0.486	0.114	2.17E-206	1.11E-07	0.297	0.424	0.114	3.58E-03
SP110	7.07E-19	0.484	0.333	0.066	2.28E-14	2.49E-126	0.249	0.299	0.066	8.03E-122	1.10E-07	0.214	0.303	0.066	3.55E-03
SRD5A1	5.29E-09	0.188	0.472	0.191	1.70E-04	9.48E-268	0.602	0.674	0.191	3.05E-263	1.25E-03	0.324	0.455	0.191	1.00E+00
SRM	5.69E-07	0.275	0.472	0.216	1.83E-02	4.53E-248	0.640	0.668	0.216	1.46E-243	4.78E-10	0.967	0.606	0.216	1.54E-05
SSBP1	4.13E-05	0.402	0.736	0.500	1.00E+00	6.39E-49	0.227	0.751	0.500	2.06E-44	2.16E-03	0.327	0.758	0.500	1.00E+00
SSNA1	2.51E-08	0.449	0.597	0.307	8.09E-04	5.84E-198	0.594	0.732	0.307	1.88E-193	8.20E-04	0.274	0.576	0.307	1.00E+00

STMP1	2.59E-07	0.440	0.639	0.339	8.33E-03	1.60E-173	0.552	0.742	0.339	5.14E-169	7.35E-08	0.924	0.697	0.339	2.37E-03
SURF2	3.01E-02	0.139	0.389	0.245	1.00E+00	4.38E-138	0.411	0.609	0.245	1.41E-133	2.57E-04	0.242	0.545	0.245	1.00E+00
SYNGR2	7.52E-10	0.768	0.694	0.412	2.42E-05	0.00E+00	1.980	0.900	0.412	0.00E+00	3.30E-04	0.399	0.697	0.412	1.00E+00
TALDO1	3.14E-05	0.535	0.625	0.410	1.00E+00	7.48E-213	0.707	0.801	0.410	2.41E-208	7.33E-04	0.251	0.667	0.410	1.00E+00
TAPBP	1.71E-16	0.813	0.722	0.305	5.49E-12	1.41E-172	0.577	0.720	0.305	4.56E-168	3.52E-05	0.282	0.697	0.305	1.00E+00
TBCB	3.96E-06	0.153	0.500	0.244	1.28E-01	9.46E-223	0.589	0.676	0.244	3.05E-218	4.44E-05	0.229	0.576	0.244	1.00E+00
TIMM8B	3.66E-04	0.197	0.611	0.410	1.00E+00	7.77E-66	0.251	0.705	0.410	2.50E-61	1.30E-07	0.877	0.788	0.410	4.17E-03
TMED10	3.65E-04	0.253	0.611	0.425	1.00E+00	1.07E-106	0.430	0.755	0.425	3.44E-102	4.51E-03	0.185	0.697	0.425	1.00E+00
TMEM54	1.27E-21	0.846	0.556	0.156	4.08E-17	0.00E+00	0.961	0.637	0.156	0.00E+00	1.73E-11	0.464	0.576	0.156	5.56E-07
TMSB10	5.40E-23	1.793	0.931	0.775	1.74E-18	6.32E-29	0.393	0.930	0.775	2.04E-24	1.71E-09	1.095	0.939	0.775	5.50E-05
TNFSF10	7.57E-38	1.265	0.681	0.157	2.44E-33	0.00E+00	1.824	0.866	0.157	0.00E+00	7.20E-06	0.222	0.455	0.157	2.32E-01
TOLLIP	3.95E-05	0.568	0.500	0.275	1.00E+00	1.79E-198	0.577	0.685	0.275	5.78E-194	8.68E-05	0.196	0.606	0.275	1.00E+00
TRAPPC1	3.58E-08	0.318	0.625	0.295	1.15E-03	9.68E-259	0.725	0.770	0.295	3.12E-254	3.57E-04	0.146	0.636	0.295	1.00E+00
TRAPPC5	2.80E-05	0.424	0.639	0.436	9.00E-01	1.59E-220	0.797	0.828	0.436	5.13E-216	6.45E-06	0.699	0.727	0.436	2.08E-01
TRIM47	5.91E-21	0.753	0.500	0.129	1.90E-16	1.04E-141	0.354	0.440	0.129	3.36E-137	4.04E-06	0.743	0.394	0.129	1.30E-01
TUBB6	1.01E-18	0.977	0.625	0.240	3.26E-14	0.00E+00	0.929	0.747	0.240	0.00E+00	7.91E-04	0.536	0.485	0.240	1.00E+00
TXNDC17	2.43E-11	1.049	0.694	0.451	7.83E-07	3.60E-52	0.281	0.718	0.451	1.16E-47	8.60E-09	0.886	0.818	0.451	2.77E-04
TYMP	9.69E-45	1.929	0.847	0.248	3.12E-40	0.00E+00	3.120	0.955	0.248	0.00E+00	3.69E-19	1.688	0.818	0.248	1.19E-14
UBE2F	3.82E-12	0.207	0.444	0.137	1.23E-07	2.52E-239	0.453	0.556	0.137	8.11E-235	1.75E-08	0.272	0.485	0.137	5.65E-04
UBE2L6	2.19E-22	0.700	0.458	0.110	7.05E-18	0.00E+00	1.026	0.666	0.110	0.00E+00	7.49E-06	0.348	0.364	0.110	2.41E-01
UQCR10	1.81E-03	0.229	0.694	0.535	1.00E+00	1.67E-95	0.391	0.824	0.535	5.38E-91	2.71E-04	0.711	0.727	0.535	1.00E+00
UQCR11	1.56E-03	0.111	0.736	0.547	1.00E+00	6.47E-61	0.155	0.810	0.547	2.08E-56	7.03E-07	0.693	0.879	0.547	2.26E-02
VPS25	6.00E-08	0.126	0.375	0.142	1.93E-03	1.16E-244	0.434	0.570	0.142	3.75E-240	2.88E-13	0.430	0.606	0.142	9.29E-09
VSNL1	2.03E-26	0.702	0.611	0.160	6.52E-22	2.02E-160	0.374	0.519	0.160	6.51E-156	5.00E-05	0.214	0.424	0.160	1.00E+00
WDR18	3.92E-13	0.265	0.444	0.139	1.26E-08	2.36E-148	0.273	0.466	0.139	7.61E-144	1.62E-07	0.374	0.455	0.139	5.23E-03
ZNRD2	2.37E-07	0.182	0.417	0.167	7.65E-03	2.94E-100	0.213	0.451	0.167	9.47E-96	5.19E-05	0.115	0.455	0.167	1.00E+00

Table S4. Differentially expressed genes in fibroblasts that are common among the MF patient samples.

	MF6					MF8					MF12					MF17				
	p val	avg logFC	pct.1	pct.2	p val adj	p val	avg logFC	pct.1	pct.2	p val adj	p_val	avg logFC	pct.1	pct.2	p val adj	p_val	avg logFC	pct.1	pct.2	p val adj
ACOT7	2.29E-74	0.293	0.358	0.030	7.39E-70	2.34E-16	0.243	0.109	0.030	7.53E-12	2.90E-10	0.178	0.135	0.030	9.35E-06	3.22E-98	0.835	0.536	0.030	1.04E-93
ADAMDEC1	3.08E-258	0.758	0.302	0.004	9.91E-254	1.21E-111	0.594	0.117	0.004	3.89E-107	2.18E-87	0.522	0.153	0.004	7.03E-83	0.00E+00	3.234	0.857	0.004	0.00E+00
ADIRF	3.89E-47	-3.759	0.208	0.849	1.25E-42	1.37E-56	-1.413	0.634	0.849	4.41E-52	5.93E-35	-2.446	0.514	0.849	1.91E-30	1.80E-23	-3.258	0.393	0.849	5.81E-19
APOL1	4.04E-259	1.628	0.726	0.037	1.30E-254	9.27E-100	0.816	0.277	0.037	2.99E-95	6.37E-180	1.280	0.595	0.037	2.05E-175	4.74E-41	0.457	0.393	0.037	1.53E-36
APOL6	3.39E-108	1.010	0.679	0.076	1.09E-103	4.83E-15	0.373	0.190	0.076	1.56E-10	1.18E-69	0.786	0.541	0.076	3.81E-65	7.89E-42	0.842	0.571	0.076	2.54E-37
ARID5A	1.38E-74	0.689	0.547	0.068	4.45E-70	1.44E-11	0.217	0.162	0.068	4.64E-07	1.37E-19	0.274	0.297	0.068	4.40E-15	7.22E-24	0.326	0.429	0.068	2.32E-19
ARPC5L	1.95E-34	0.389	0.547	0.121	6.28E-30	6.19E-14	0.377	0.257	0.121	1.99E-09	2.00E-13	0.196	0.369	0.121	6.46E-09	1.84E-13	0.486	0.464	0.121	5.94E-09
ARSA	6.59E-31	0.323	0.387	0.073	2.12E-26	9.26E-17	0.251	0.193	0.073	2.98E-12	4.42E-11	0.166	0.243	0.073	1.42E-06	1.77E-18	0.358	0.393	0.073	5.70E-14
ATOX1	1.70E-11	0.444	0.717	0.390	5.47E-07	7.32E-06	0.360	0.500	0.390	2.36E-01	3.30E-14	0.595	0.739	0.390	1.06E-09	1.52E-09	0.463	0.857	0.390	4.89E-05
B2M	1.41E-58	2.019	1.000	0.987	4.55E-54	7.26E-153	1.633	1.000	0.987	2.34E-148	5.09E-60	2.062	1.000	0.987	1.64E-55	1.97E-27	1.437	1.000	0.987	6.36E-23
BAX	2.04E-12	0.259	0.566	0.222	6.57E-08	6.13E-08	0.286	0.344	0.222	1.97E-03	1.16E-13	0.557	0.532	0.222	3.75E-09	1.65E-12	0.722	0.661	0.222	5.32E-08
BCAR1	7.30E-49	0.506	0.434	0.063	2.35E-44	1.30E-07	0.144	0.134	0.063	4.20E-03	3.79E-25	0.282	0.315	0.063	1.22E-20	1.34E-15	0.166	0.339	0.063	4.32E-11
BIRC3	5.78E-185	2.315	0.868	0.080	1.86E-180	1.05E-15	0.565	0.198	0.080	3.38E-11	5.49E-15	0.500	0.288	0.080	1.77E-10	1.05E-32	0.786	0.536	0.080	3.37E-28
BMP1	1.46E-33	0.426	0.528	0.116	4.72E-29	7.28E-22	0.492	0.285	0.116	2.35E-17	2.24E-70	1.088	0.667	0.116	7.22E-66	8.85E-16	0.253	0.500	0.116	2.85E-11
BST2	4.60E-123	2.081	0.726	0.084	1.48E-118	6.45E-184	1.870	0.536	0.084	2.08E-179	4.42E-112	2.364	0.676	0.084	1.42E-107	3.27E-89	2.091	0.821	0.084	1.05E-84
BTN3A2	8.92E-35	0.339	0.415	0.075	2.87E-30	1.79E-32	0.369	0.251	0.075	5.76E-28	7.04E-28	0.502	0.360	0.075	2.27E-23	1.22E-44	0.671	0.589	0.075	3.94E-40
C1QTNF6	3.48E-45	0.349	0.415	0.061	1.12E-40	7.09E-16	0.176	0.170	0.061	2.28E-11	5.81E-58	0.747	0.441	0.061	1.87E-53	4.83E-13	0.294	0.304	0.061	1.56E-08
CCL19	8.49E-269	5.525	0.934	0.066	2.74E-264	3.00E-63	2.709	0.299	0.066	9.68E-59	2.37E-14	0.772	0.252	0.066	7.62E-10	1.34E-126	4.735	0.857	0.066	4.32E-122
CCM2	1.15E-33	0.364	0.491	0.103	3.70E-29	4.83E-05	0.132	0.173	0.103	1.00E+00	3.12E-07	0.151	0.261	0.103	1.00E-02	3.64E-13	0.294	0.429	0.103	1.17E-08
CD40	2.95E-133	2.037	0.783	0.095	9.51E-129	9.50E-10	0.191	0.196	0.095	3.06E-05	5.95E-10	0.225	0.279	0.095	1.92E-05	5.24E-31	0.680	0.571	0.095	1.69E-26
CD74	1.94E-143	4.022	0.925	0.132	6.26E-139	1.35E-109	2.030	0.536	0.132	4.34E-105	1.53E-52	1.937	0.622	0.132	4.92E-48	2.73E-98	5.310	0.982	0.132	8.81E-94
CD82	5.32E-165	2.285	0.802	0.081	1.71E-160	3.34E-61	0.750	0.332	0.081	1.08E-56	2.83E-30	0.558	0.387	0.081	9.11E-26	4.22E-49	0.813	0.643	0.081	1.36E-44
CFD	3.73E-55	-4.939	0.406	0.934	1.20E-50	1.09E-90	-2.098	0.656	0.934	3.50E-86	1.23E-45	-3.360	0.604	0.934	3.96E-41	1.51E-18	-2.607	0.875	0.934	4.85E-14
CFH	1.39E-10	-1.375	0.792	0.769	4.47E-06	6.81E-34	-1.279	0.598	0.769	2.19E-29	1.41E-06	-1.070	0.838	0.769	4.54E-02	3.68E-08	-1.632	0.768	0.769	1.19E-03
CHN1	1.59E-69	0.929	0.453	0.051	5.11E-65	4.53E-175	1.083	0.427	0.051	1.46E-170	2.55E-195	1.614	0.721	0.051	8.20E-191	1.24E-90	1.009	0.679	0.051	3.99E-86
CHST11	3.98E-89	0.412	0.368	0.026	1.28E-84	1.17E-32	0.322	0.137	0.026	3.76E-28	4.74E-35	0.351	0.225	0.026	1.53E-30	5.53E-31	0.285	0.286	0.026	1.78E-26
CHST15	3.47E-128	0.435	0.340	0.015	1.12E-123	9.27E-32	0.245	0.101	0.015	2.99E-27	3.07E-76	0.352	0.252	0.015	9.89E-72	8.23E-92	0.404	0.375	0.015	2.65E-87
CLSTN3	6.69E-115	0.950	0.689	0.071	2.16E-110	7.10E-17	0.291	0.193	0.071	2.29E-12	1.91E-11	0.306	0.243	0.071	6.15E-07	1.68E-94	1.340	0.804	0.071	5.40E-90
COL18A1	1.60E-04	-0.553	0.604	0.305	1.00E+00	1.20E-20	0.422	0.547	0.305	3.85E-16	4.50E-32	1.081	0.811	0.305	1.45E-27	5.90E-19	0.809	0.893	0.305	1.90E-14
COL27A1	1.57E-123	1.172	0.557	0.045	5.07E-119	1.35E-14	0.267	0.134	0.045	4.34E-10	6.86E-34	0.367	0.297	0.045	2.21E-29	3.39E-27	0.471	0.357	0.045	1.09E-22
COL4A1	1.14E-23	0.490	0.547	0.156	3.68E-19	1.30E-34	0.679	0.399	0.156	4.17E-30	3.25E-82	2.395	0.775	0.156	1.05E-77	4.76E-31	1.234	0.732	0.156	1.53E-26
COL4A2	6.54E-06	0.270	0.434	0.211	2.11E-01	1.66E-18	0.460	0.402	0.211	5.34E-14	3.47E-62	1.619	0.802	0.211	1.12E-57	2.21E-22	1.199	0.750	0.211	7.12E-18
COL4A4	6.01E-262	0.645	0.443	0.011	1.94E-257	1.58E-84	0.433	0.145	0.011	5.09E-80	1.39E-93	0.604	0.243	0.011	4.47E-89	3.23E-110	0.730	0.357	0.011	1.04E-105
COL6A3	1.46E-08	0.805	0.896	0.634	4.70E-04	2.71E-63	1.899	0.791	0.634	8.72E-59	6.44E-61	2.673	0.991	0.634	2.07E-56	3.99E-11	1.504	0.875	0.634	1.28E-06
COL6A5	8.31E-62	0.309	0.330	0.030	2.68E-57	0.00E+00	1.564	0.500	0.030	0.00E+00	5.50E-297	1.948	0.712	0.030	1.77E-292	3.80E-38	0.749	0.339	0.030	1.22E-33
COL6A6	6.97E-13	0.156	0.208	0.049	2.24E-08	4.60E-217	1.573	0.458	0.049	1.48E-212	1.42E-150	1.641	0.613	0.049	4.56E-146	4.00E-22	0.511	0.339	0.049	1.29E-17
COX5A	2.99E-15	0.426	0.745	0.325	9.62E-11	2.21E-14	0.567	0.494	0.325	7.13E-10	4.63E-07	0.348	0.586	0.325	1.49E-02	1.16E-05	0.300	0.661	0.325	3.72E-01
CPE	2.57E-19	-2.035	0.151	0.562	8.27E-15	5.16E-50	-1.504	0.162	0.562	1.66E-45	2.89E-04	-0.640	0.432	0.562	1.00E+00	2.65E-08	-1.729	0.268	0.562	8.54E-04
CSF1	7.59E-41	0.562	0.642	0.145	2.44E-36	8.91E-03	0.139	0.196	0.145	1.00E+00	9.87E-09	0.139	0.360	0.145	3.18E-04	1.45E-32	1.030	0.714	0.145	4.67E-28
CSRP2	2.60E-25	0.349	0.425	0.097	8.38E-21	1.52E-38	0.622	0.310	0.097	4.90E-34	2.22E-08	0.257	0.261	0.097	7.16E-04	3.12E-13	0.224	0.411	0.097	1.00E-08
CTSB	8.05E-37	1.930	0.849	0.426	2.59E-32	4.66E-28	0.880	0.640	0.426	1.50E-23	1.12E-17	1.002	0.748	0.426	3.61E-13	8.91E-16	1.186	0.911	0.426	2.87E-11
CTSC	3.07E-92	2.326	0.811	0.146	9.88E-88	1.64E-79	1.214	0.506	0.146	5.27E-75	4.87E-99	2.437	0.820	0.146	1.57E-94	1.31E-50	2.266	0.821	0.146	4.20E-46
CXCL16	3.01E-35	0.246	0.377	0.062	9.69E-31	1.71E-12	0.180	0.159	0.062	5.52E-08	4.14E-22	0.376	0.297	0.062	1.33E-17	1.13E-50	0.572	0.571	0.062	3.64E-46
CYP7B1	1.68E-85	1.163	0.736	0.116	5.43E-81	4.79E-105	1.209	0.489	0.116	1.54E-100	6.25E-89	1.340	0.730	0.116	2.01E-84	3.63E-48	1.350	0.732	0.116	1.17E-43
DAAM1	1.29E-23	0.323	0.368	0.081	4.16E-19	4.84E-37	0.488	0.277	0.081	1.56E-32	1.22E-07	0.160	0.225	0.081	3.93E-03	3.60E-21	0.328	0.446	0.081	1.16E-16

DCN	2.53E-35	-1.857	0.906	0.979	8.15E-31	1.44E-18	-0.615	0.958	0.979	4.64E-14	3.88E-09	-0.733	0.991	0.979	1.25E-04	2.44E-22	-2.143	0.911	0.979	7.86E-18
DYNLT1	3.39E-42	1.230	0.792	0.261	1.09E-37	1.71E-27	0.742	0.497	0.261	5.51E-23	7.30E-25	0.878	0.676	0.261	2.35E-20	2.23E-16	0.739	0.750	0.261	7.18E-12
EDF1	5.15E-06	0.382	0.915	0.762	1.66E-01	1.62E-13	0.453	0.802	0.762	5.21E-09	2.81E-06	0.341	0.910	0.762	9.06E-02	4.97E-04	0.371	0.929	0.762	1.00E+00
EFEMP2	3.77E-08	0.439	0.670	0.398	1.21E-03	9.59E-10	0.518	0.511	0.398	3.09E-05	8.65E-14	0.603	0.730	0.398	2.79E-09	8.05E-07	0.474	0.732	0.398	2.59E-02
ELK3	1.52E-32	0.417	0.453	0.091	4.89E-28	5.75E-05	0.137	0.156	0.091	1.00E+00	3.37E-09	0.237	0.261	0.091	1.08E-04	2.24E-06	0.167	0.286	0.091	7.20E-02
ELN	6.09E-09	-1.627	0.274	0.471	1.96E-04	9.51E-25	-1.228	0.207	0.471	3.06E-20	3.34E-06	-1.083	0.297	0.471	1.08E-01	3.08E-06	-1.715	0.232	0.471	9.91E-02
EPAS1	8.24E-86	0.773	0.557	0.063	2.66E-81	1.74E-63	0.662	0.299	0.063	5.60E-59	1.13E-79	0.867	0.523	0.063	3.65E-75	8.03E-18	0.337	0.357	0.063	2.59E-13
EPSTI1	1.30E-251	1.692	0.811	0.049	4.20E-247	2.15E-25	0.377	0.176	0.049	6.94E-21	3.11E-77	0.786	0.450	0.049	1.00E-72	1.57E-42	0.607	0.464	0.049	5.05E-38
ETHE1	1.19E-31	0.725	0.604	0.173	3.84E-27	7.91E-17	0.626	0.338	0.173	2.55E-12	7.54E-11	0.310	0.432	0.173	2.43E-06	4.51E-20	0.681	0.679	0.173	1.45E-15
FAM207A	2.69E-21	0.110	0.358	0.080	8.67E-17	7.29E-04	0.215	0.131	0.080	1.00E+00	7.25E-10	0.117	0.252	0.080	2.33E-05	2.23E-06	0.389	0.268	0.080	7.18E-02
FAM20A	1.02E-68	0.803	0.623	0.097	3.27E-64	5.62E-43	0.674	0.321	0.097	1.81E-38	2.59E-59	0.946	0.568	0.097	8.33E-55	3.14E-23	0.447	0.518	0.097	1.01E-18
FBLN1	2.25E-24	-2.163	0.708	0.850	7.24E-20	1.70E-33	-1.247	0.693	0.850	5.48E-29	1.07E-10	-1.246	0.811	0.850	3.44E-06	6.31E-17	-2.537	0.643	0.850	2.03E-12
FRMD6	6.80E-11	0.121	0.500	0.196	2.19E-06	1.71E-07	0.337	0.304	0.196	5.49E-03	1.84E-29	0.837	0.622	0.196	5.93E-25	4.55E-19	1.126	0.679	0.196	1.46E-14
G0S2	1.96E-168	3.076	0.726	0.061	6.31E-164	1.32E-09	0.488	0.140	0.061	4.24E-05	3.20E-09	0.661	0.198	0.061	1.03E-04	6.23E-10	0.733	0.268	0.061	2.01E-05
GAPDH	8.71E-07	0.498	0.991	0.878	2.81E-02	1.47E-26	0.621	0.936	0.878	4.73E-22	1.26E-13	0.642	0.964	0.878	4.06E-09	4.38E-08	0.842	1.000	0.878	1.41E-03
GBP2	1.97E-55	0.960	0.689	0.144	6.34E-51	3.36E-06	0.331	0.235	0.144	1.08E-01	7.31E-15	0.596	0.405	0.144	2.35E-10	1.77E-26	0.784	0.661	0.144	5.69E-22
GGCT	2.58E-27	0.312	0.547	0.139	8.32E-23	5.63E-12	0.240	0.277	0.139	1.81E-07	2.11E-19	0.473	0.450	0.139	6.80E-15	1.51E-12	0.183	0.518	0.139	4.86E-08
GPR137	4.07E-51	0.493	0.547	0.093	1.31E-46	8.25E-11	0.232	0.198	0.093	2.66E-06	1.47E-10	0.283	0.279	0.093	4.74E-06	3.86E-26	0.381	0.536	0.093	1.24E-21
GSN	3.97E-53	-3.898	0.736	0.944	1.28E-48	7.91E-86	-2.011	0.785	0.944	2.55E-81	4.36E-49	-3.101	0.784	0.944	1.41E-44	6.20E-26	-3.269	0.821	0.944	2.00E-21
GUCY1A1	4.74E-170	0.499	0.415	0.017	1.53E-165	2.24E-270	1.042	0.335	0.017	7.22E-266	2.89E-209	1.157	0.450	0.017	9.30E-205	4.26E-286	1.552	0.714	0.017	1.37E-281
GUCY1B1	1.39E-169	0.450	0.406	0.016	4.46E-165	2.26E-33	0.197	0.106	0.016	7.27E-29	2.38E-41	0.280	0.189	0.016	7.66E-37	5.59E-184	0.837	0.554	0.016	1.80E-179
H2AFY2	4.20E-144	0.327	0.302	0.010	1.35E-139	1.75E-49	0.217	0.103	0.010	5.65E-45	1.49E-47	0.161	0.162	0.010	4.79E-43	1.65E-98	0.300	0.321	0.010	5.32E-94
HDDC2	1.46E-12	0.131	0.509	0.184	4.72E-08	1.82E-04	0.222	0.268	0.184	1.00E+00	4.21E-08	0.242	0.405	0.184	1.36E-03	1.55E-10	0.300	0.571	0.184	4.98E-06
HEG1	3.67E-109	0.707	0.538	0.047	1.18E-104	1.24E-27	0.464	0.176	0.047	3.99E-23	8.77E-12	0.200	0.189	0.047	2.82E-07	3.04E-65	0.605	0.554	0.047	9.80E-61
HES4	4.53E-35	0.344	0.349	0.054	1.46E-30	9.18E-29	0.390	0.198	0.054	2.96E-24	6.20E-36	0.427	0.342	0.054	2.00E-31	6.32E-97	1.273	0.714	0.054	2.04E-92
HIF1A	1.35E-77	1.024	0.717	0.116	4.35E-73	7.81E-82	1.233	0.441	0.116	2.52E-77	2.20E-66	1.091	0.649	0.116	7.09E-62	3.98E-21	0.615	0.536	0.116	1.28E-16
HLA-A	1.04E-50	2.110	0.991	0.785	3.35E-46	8.80E-65	1.432	0.852	0.785	2.83E-60	1.86E-52	2.199	0.973	0.785	5.99E-48	1.21E-25	2.105	0.964	0.785	3.89E-21
HLA-B	1.57E-63	2.696	1.000	0.676	5.05E-59	3.25E-144	2.606	0.919	0.676	1.05E-139	1.17E-59	2.268	0.991	0.676	3.77E-55	1.24E-35	2.951	0.982	0.676	3.99E-31
HLA-C	1.30E-22	1.267	0.943	0.782	4.18E-18	1.91E-69	1.288	0.891	0.782	6.15E-65	3.04E-32	1.564	0.973	0.782	9.78E-28	8.31E-14	0.929	0.982	0.782	2.68E-09
HLA-E	3.10E-13	0.699	0.840	0.569	9.98E-09	3.20E-10	0.518	0.642	0.569	1.03E-05	4.78E-23	0.985	0.919	0.569	1.54E-18	6.74E-24	1.800	0.911	0.569	2.17E-19
HLA-F	1.54E-130	1.376	0.745	0.080	4.97E-126	1.07E-87	0.908	0.385	0.080	3.45E-83	7.44E-92	1.066	0.631	0.080	2.40E-87	8.34E-87	1.416	0.821	0.080	2.69E-82
IDH2	1.91E-20	0.394	0.406	0.105	6.17E-16	1.14E-13	0.318	0.229	0.105	3.67E-09	1.44E-21	0.401	0.396	0.105	4.64E-17	2.86E-09	0.109	0.375	0.105	9.20E-05
IFI27	2.87E-60	2.324	0.811	0.213	9.24E-56	2.43E-213	2.833	0.832	0.213	7.83E-209	2.97E-37	1.746	0.703	0.213	9.57E-33	4.59E-14	1.066	0.607	0.213	1.48E-09
IFI30	0.00E+00	2.188	0.821	0.034	0.00E+00	7.40E-158	0.911	0.335	0.034	2.38E-153	1.89E-172	1.396	0.559	0.034	6.08E-168	6.01E-117	1.272	0.625	0.034	1.94E-112
IFITM1	1.88E-04	-0.137	0.774	0.471	1.00E+00	1.88E-14	-1.169	0.344	0.471	6.05E-10	1.11E-07	0.168	0.775	0.471	3.57E-03	2.64E-25	1.671	0.982	0.471	8.51E-21
IFITM3	6.48E-35	1.275	0.962	0.881	2.09E-30	1.98E-42	0.769	0.913	0.881	6.39E-38	1.12E-42	1.314	0.982	0.881	3.60E-38	5.69E-19	1.238	0.982	0.881	1.83E-14
IKBIP	1.09E-27	0.519	0.594	0.164	3.50E-23	6.95E-30	0.648	0.385	0.164	2.24E-25	3.18E-10	0.169	0.414	0.164	1.03E-05	2.16E-07	0.205	0.464	0.164	6.94E-03
IL1R1	5.55E-52	0.878	0.642	0.128	1.79E-47	9.20E-22	0.495	0.302	0.128	2.96E-17	2.18E-82	1.352	0.730	0.128	7.02E-78	5.14E-16	0.427	0.518	0.128	1.66E-11
IL32	9.04E-224	3.391	0.943	0.086	2.91E-219	0.00E+00	2.590	0.701	0.086	0.00E+00	1.69E-128	2.018	0.739	0.086	5.45E-124	4.56E-120	2.916	0.929	0.086	1.47E-115
IL4I1	0.00E+00	1.418	0.585	0.005	0.00E+00	6.20E-236	0.668	0.204	0.005	2.00E-231	4.97E-42	0.154	0.108	0.005	1.60E-37	6.28E-73	0.188	0.196	0.005	2.02E-68
IRF8	0.00E+00	1.116	0.547	0.009	0.00E+00	4.67E-52	0.417	0.101	0.009	1.50E-47	2.06E-42	0.176	0.144	0.009	6.65E-38	0.00E+00	1.163	0.643	0.009	0.00E+00
IRF9	6.08E-33	0.490	0.698	0.187	1.96E-28	7.80E-15	0.475	0.352	0.187	2.51E-10	2.03E-19	0.456	0.550	0.187	6.55E-15	7.27E-15	0.479	0.625	0.187	2.34E-10
KLF4	5.34E-04	-0.987	0.519	0.541	1.00E+00	1.37E-41	-1.415	0.201	0.541	4.42E-37	3.11E-12	-1.414	0.261	0.541	1.00E-07	2.32E-04	-1.243	0.429	0.541	1.00E+00
KPNA2	3.36E-81	0.635	0.632	0.082	1.08E-76	2.15E-14	0.209	0.201	0.082	6.93E-10	8.16E-12	0.275	0.270	0.082	2.63E-07	3.40E-17	0.352	0.411	0.082	1.09E-12
LAP3	3.87E-128	2.619	0.906	0.151	1.25E-123	1.10E-42	0.893	0.402	0.151	3.55E-38	7.23E-88	1.707	0.793	0.151	2.33E-83	2.14E-53	1.808	0.839	0.151	6.88E-49
LGALS3BP	2.87E-35	1.290	0.802	0.293	9.23E-31	4.70E-16	0.720	0.458	0.293	1.51E-11	3.00E-47	1.791	0.802	0.293	9.66E-43	4.20E-15	1.074	0.750	0.293	1.35E-10
LGALS9	3.60E-74	0.533	0.443	0.046	1.16E-69	1.18E-09	0.192	0.117	0.046	3.79E-05	7.70E-12	0.178	0.189	0.046	2.48E-07	1.19E-67	0.787	0.554	0.046	3.84E-63
LMO4	3.19E-57	1.514	0.840	0.239	1.03E-52	4.91E-02	-0.272	0.207	0.239	1.00E+00	9.75E-06	0.168	0.450	0.239	3.14E-01	1.13E-22	1.000	0.821	0.239	3.64E-18
LOXL2	2.23E-42	0.353	0.453	0.074	7.18E-38	1.33E-63	0.654	0.324	0.074	4.27E-59	1.37E-64	1.034	0.514	0.074	4.41E-60	2.68E-39	0.820	0.554	0.074	8.64E-35
LPCAT3	3.96E-27	0.153	0.302	0.051	1.27E-22	2.58E-09	0.113	0.126	0.051	8.32E-05	4.67E-09	0.160	0.180	0.051	1.50E-04	2.97E-12	0.201	0.268	0.051	9.55E-08
MDK	1.20E-18	0.680	0.642	0.256	3.85E-14	1.06E-04	-0.436	0.176	0.256	1.00E+00	5.06E-15	0.469	0.604	0.256	1.63E-10	4.91E-30	1.525	0.839	0.256	1.58E-25

MFAP4	1.03E-23	-2.004	0.679	0.818	3.33E-19	3.84E-39	-1.201	0.587	0.818	1.24E-34	4.67E-16	-1.420	0.712	0.818	1.50E-11	3.20E-05	-0.892	0.732	0.818	1.00E+00
MGP	1.10E-30	-2.981	0.538	0.815	3.54E-26	4.45E-111	-3.370	0.299	0.815	1.43E-106	2.37E-37	-3.142	0.252	0.815	7.63E-33	5.02E-14	-2.842	0.696	0.815	1.62E-09
MIF	2.57E-14	0.671	0.934	0.763	8.26E-10	6.39E-40	0.956	0.883	0.763	2.06E-35	3.01E-31	1.114	0.991	0.763	9.71E-27	3.97E-08	0.741	0.964	0.763	1.28E-03
MIR4435-2HG	4.86E-21	0.602	0.377	0.095	1.56E-16	6.11E-16	0.349	0.226	0.095	1.97E-11	2.72E-54	1.066	0.532	0.095	8.77E-50	1.41E-39	0.850	0.625	0.095	4.56E-35
MRPL36	3.85E-15	0.201	0.557	0.195	1.24E-10	2.61E-14	0.379	0.363	0.195	8.40E-10	2.97E-07	0.148	0.423	0.195	9.56E-03	9.27E-09	0.112	0.571	0.195	2.99E-04
MSC	9.81E-95	0.856	0.604	0.068	3.16E-90	2.25E-24	0.383	0.212	0.068	7.26E-20	6.25E-22	0.497	0.306	0.068	2.01E-17	3.43E-85	1.124	0.750	0.068	1.11E-80
MT-ATP8	9.54E-07	-1.778	0.132	0.330	3.07E-02	1.03E-30	-1.884	0.039	0.330	3.32E-26	2.16E-12	-1.997	0.009	0.330	6.95E-08	1.03E-27	1.671	0.911	0.330	3.30E-23
MT-ND6	8.15E-03	-1.113	0.160	0.242	1.00E+00	4.66E-17	-1.137	0.053	0.242	1.50E-12	2.43E-02	-0.931	0.171	0.242	1.00E+00	3.99E-37	1.751	0.929	0.242	1.29E-32
MYH9	1.58E-34	0.486	0.726	0.197	5.09E-30	4.15E-06	0.169	0.302	0.197	1.34E-01	1.04E-25	0.640	0.613	0.197	3.34E-21	8.49E-37	1.297	0.857	0.197	2.74E-32
NDFIP1	1.75E-10	-1.129	0.660	0.659	5.64E-06	1.23E-28	-0.853	0.458	0.659	3.97E-24	1.50E-07	-0.817	0.640	0.659	4.82E-03	1.36E-04	-0.942	0.750	0.659	1.00E+00
NFKB2	1.89E-74	0.739	0.660	0.098	6.09E-70	4.54E-05	0.207	0.165	0.098	1.00E+00	2.34E-10	0.248	0.288	0.098	7.52E-06	1.10E-50	0.891	0.714	0.098	3.55E-46
NNMT	1.65E-29	1.496	0.915	0.587	5.32E-25	1.77E-31	1.005	0.729	0.587	5.70E-27	1.09E-27	1.369	0.892	0.587	3.52E-23	3.41E-02	0.256	0.857	0.587	1.00E+00
NR4A1	5.72E-03	-1.314	0.406	0.445	1.00E+00	1.20E-22	-1.515	0.204	0.445	3.88E-18	1.56E-10	-1.951	0.180	0.445	5.03E-06	4.70E-02	-0.725	0.357	0.445	1.00E+00
NUB1	7.19E-53	0.966	0.679	0.143	2.32E-48	1.39E-08	0.349	0.251	0.143	4.48E-04	5.91E-09	0.123	0.360	0.143	1.90E-04	2.35E-17	0.446	0.571	0.143	7.58E-13
OSBPL8	1.12E-15	0.216	0.594	0.213	3.62E-11	1.36E-16	0.488	0.388	0.213	4.37E-12	2.70E-06	0.192	0.423	0.213	8.69E-02	8.69E-11	0.299	0.625	0.213	2.80E-06
OSMR	3.44E-71	0.856	0.642	0.096	1.11E-66	2.19E-12	0.314	0.212	0.096	7.07E-08	4.00E-08	0.150	0.261	0.096	1.29E-03	1.99E-15	0.411	0.429	0.096	6.42E-11
OSTC	2.85E-08	0.382	0.783	0.463	9.19E-04	1.75E-19	0.751	0.620	0.463	5.64E-15	4.51E-24	0.975	0.847	0.463	1.45E-19	4.79E-04	0.122	0.821	0.463	1.00E+00
P4HB	1.56E-14	0.749	0.858	0.548	5.01E-10	6.32E-18	0.705	0.679	0.548	2.04E-13	6.09E-15	0.830	0.802	0.548	1.96E-10	1.98E-03	0.258	0.821	0.548	1.00E+00
PAPSS2	2.32E-63	0.336	0.340	0.031	7.49E-59	3.26E-27	0.293	0.140	0.031	1.05E-22	3.57E-31	0.361	0.234	0.031	1.15E-26	1.09E-57	0.374	0.429	0.031	3.51E-53
PARP14	2.53E-151	2.121	0.887	0.101	8.14E-147	1.02E-47	0.686	0.346	0.101	3.27E-43	2.74E-71	1.317	0.622	0.101	8.83E-67	5.31E-41	0.922	0.661	0.101	1.71E-36
PDGFRL	1.21E-14	-1.590	0.075	0.441	3.90E-10	1.14E-12	-0.715	0.296	0.441	3.66E-08	4.64E-09	-1.213	0.207	0.441	1.50E-04	2.69E-08	-1.619	0.089	0.441	8.66E-04
PFN1	4.86E-14	0.866	0.896	0.754	1.57E-09	3.10E-08	0.422	0.737	0.754	9.99E-04	7.01E-13	0.621	0.928	0.754	2.26E-08	8.78E-13	0.839	0.964	0.754	2.83E-08
PLAAT4	1.89E-39	1.268	0.717	0.230	6.09E-35	1.53E-13	0.591	0.385	0.230	4.92E-09	1.11E-15	0.683	0.541	0.230	3.58E-11	3.59E-20	0.942	0.750	0.230	1.16E-15
PLAC9	2.65E-23	-1.523	0.830	0.855	8.53E-19	5.05E-77	-1.576	0.592	0.855	1.63E-72	8.28E-26	-1.622	0.730	0.855	2.67E-21	8.60E-16	-1.831	0.768	0.855	2.77E-11
PLEKHO1	2.92E-93	0.746	0.557	0.058	9.41E-89	1.13E-06	0.214	0.120	0.058	3.64E-02	1.45E-11	0.144	0.216	0.058	4.67E-07	1.20E-19	0.291	0.357	0.058	3.87E-15
PLSCR1	2.93E-51	1.211	0.774	0.207	9.44E-47	3.40E-11	0.393	0.352	0.207	1.10E-06	6.66E-32	0.830	0.658	0.207	2.15E-27	2.83E-09	0.382	0.571	0.207	9.11E-05
PLXND1	6.00E-21	0.339	0.415	0.106	1.93E-16	2.45E-02	-0.162	0.070	0.106	1.00E+00	4.65E-10	0.280	0.297	0.106	1.50E-05	4.70E-17	0.303	0.482	0.106	1.51E-12
PPIB	2.24E-11	0.632	0.877	0.730	7.21E-07	7.18E-25	0.641	0.810	0.730	2.31E-20	5.57E-14	0.680	0.928	0.730	1.79E-09	3.45E-03	0.283	0.893	0.730	1.00E+00
PRSS23	4.19E-62	1.710	0.821	0.188	1.35E-57	8.03E-21	0.768	0.380	0.188	2.59E-16	5.08E-35	1.188	0.649	0.188	1.64E-30	1.42E-16	1.373	0.607	0.188	4.56E-12
PSMB5	1.51E-08	0.356	0.651	0.368	4.86E-04	1.89E-12	0.497	0.508	0.368	6.10E-08	1.30E-12	0.487	0.703	0.368	4.19E-08	3.68E-03	0.101	0.625	0.368	1.00E+00
PSMB9	2.81E-68	1.496	0.802	0.185	9.06E-64	2.17E-60	1.121	0.506	0.185	7.00E-56	8.11E-74	1.684	0.802	0.185	2.61E-69	4.99E-49	1.547	0.893	0.185	1.61E-44
RAB13	4.70E-50	1.709	0.915	0.414	1.51E-45	2.53E-42	1.244	0.654	0.414	8.16E-38	9.04E-23	0.966	0.811	0.414	2.91E-18	4.75E-15	1.157	0.893	0.414	1.53E-10
RARRES2	6.48E-05	0.203	0.585	0.344	1.00E+00	2.90E-44	1.192	0.637	0.344	9.33E-40	1.22E-33	1.931	0.793	0.344	3.93E-29	7.92E-15	1.043	0.839	0.344	2.55E-10
RBP5	2.58E-178	1.242	0.623	0.039	8.30E-174	2.56E-68	0.690	0.237	0.039	8.25E-64	8.18E-11	0.268	0.162	0.039	2.64E-06	1.81E-204	2.040	0.875	0.039	5.84E-200
RPL18	3.06E-07	-0.575	0.915	0.905	9.84E-03	2.45E-03	-0.133	0.908	0.905	1.00E+00	2.79E-05	-0.448	0.964	0.905	8.98E-01	3.36E-10	0.612	1.000	0.905	1.08E-05
RPL22L1	2.40E-43	1.445	0.858	0.312	7.72E-39	4.04E-24	0.835	0.556	0.312	1.30E-19	4.99E-14	0.873	0.640	0.312	1.61E-09	6.00E-14	0.755	0.839	0.312	1.93E-09
RPL41	2.11E-11	-0.547	0.991	0.985	6.80E-07	2.48E-49	-0.660	0.983	0.985	7.97E-45	2.12E-04	-0.270	0.991	0.985	1.00E+00	5.39E-27	-1.831	0.982	0.985	1.74E-22
RPS4Y1	3.62E-09	-1.363	0.000	0.253	1.16E-04	3.29E-27	-1.363	0.000	0.253	1.06E-22	1.29E-34	0.847	0.829	0.253	4.16E-30	1.77E-05	-1.363	0.000	0.253	5.69E-01
S100A4	1.98E-12	-1.052	0.915	0.931	6.37E-08	8.43E-07	0.179	0.933	0.931	2.71E-02	2.06E-04	-0.605	0.937	0.931	1.00E+00	6.80E-17	-1.889	0.857	0.931	2.19E-12
SELENOP	2.53E-31	-2.106	0.509	0.822	8.14E-27	3.06E-86	-1.784	0.425	0.822	9.86E-82	6.36E-21	-1.447	0.577	0.822	2.05E-16	1.60E-11	-1.501	0.732	0.822	5.14E-07
SEMA5A	2.59E-46	0.446	0.387	0.053	8.35E-42	2.11E-39	0.477	0.221	0.053	6.78E-35	3.76E-73	0.780	0.459	0.053	1.21E-68	1.79E-77	0.756	0.643	0.053	5.76E-73
SLFN5	1.35E-29	0.312	0.491	0.111	4.35E-25	7.07E-26	0.557	0.288	0.111	2.28E-21	1.07E-11	0.256	0.324	0.111	3.43E-07	4.72E-14	0.396	0.446	0.111	1.52E-09
SLIT3	1.84E-22	-1.984	0.236	0.641	5.92E-18	7.77E-29	-0.910	0.391	0.641	2.50E-24	3.33E-12	-1.297	0.468	0.641	1.07E-07	7.46E-05	-1.056	0.589	0.641	1.00E+00
SNHG5	3.85E-11	0.546	0.934	0.737	1.24E-06	7.10E-03	-0.229	0.721	0.737	1.00E+00	5.37E-15	-1.469	0.640	0.737	1.73E-10	2.64E-06	-1.300	0.786	0.737	8.51E-02
SOD3	5.80E-16	-1.047	0.811	0.898	1.87E-11	1.35E-97	-1.752	0.589	0.898	4.33E-93	5.69E-05	-0.392	0.919	0.898	1.00E+00	3.09E-03	-0.508	0.929	0.898	1.00E+00
SPCS3	3.79E-21	0.130	0.472	0.125	1.22E-16	5.77E-16	0.303	0.274	0.125	1.86E-11	1.63E-16	0.262	0.405	0.125	5.24E-12	2.20E-11	0.283	0.446	0.125	7.09E-07
SPHK1	6.70E-290	0.901	0.594	0.020	2.16E-285	3.51E-47	0.301	0.140	0.020	1.13E-42	6.51E-138	0.811	0.387	0.020	2.10E-133	4.88E-47	0.300	0.304	0.020	1.57E-42
SQLE	5.78E-79	0.548	0.462	0.047	1.86E-74	8.82E-14	0.170	0.137	0.047	2.84E-09	6.49E-12	0.387	0.189	0.047	2.09E-07	8.49E-06	0.170	0.179	0.047	2.73E-01
STAT1	3.28E-121	1.767	0.830	0.116	1.06E-116	4.60E-39	0.662	0.341	0.116	1.48E-34	5.89E-143	2.358	0.856	0.116	1.90E-138	2.55E-77	2.383	0.857	0.116	8.23E-73
STK17A	1.91E-37	0.186	0.368	0.056	6.16E-33	2.45E-08	0.127	0.128	0.056	7.88E-04	4.56E-12	0.198	0.216	0.056	1.47E-07	7.88E-41	0.753	0.482	0.056	2.54E-36
SULF1	3.02E-157	0.985	0.500	0.028	9.74E-153	5.63E-15	0.214	0.101	0.028	1.81E-10	1.31E-33	0.736	0.225	0.028	4.23E-29	4.80E-09	0.161	0.161	0.028	1.54E-04

SVEP1	5.76E-06	-1.190	0.226	0.386	1.86E-01	2.64E-19	-0.980	0.165	0.386	8.50E-15	1.96E-03	-0.875	0.288	0.386	1.00E+00	4.71E-03	-1.117	0.268	0.386	1.00E+00
TAF9	1.77E-16	0.136	0.519	0.163	5.70E-12	6.59E-04	0.155	0.240	0.163	1.00E+00	3.89E-06	0.194	0.342	0.163	1.25E-01	1.55E-08	0.477	0.482	0.163	4.99E-04
TAP1	6.71E-118	1.318	0.708	0.081	2.16E-113	9.81E-47	0.489	0.302	0.081	3.16E-42	7.27E-60	0.698	0.523	0.081	2.34E-55	1.46E-62	1.165	0.696	0.081	4.70E-58
TDO2	0.00E+00	1.833	0.642	0.001	0.00E+00	4.43E-212	0.535	0.137	0.001	1.43E-207	0.00E+00	1.901	0.423	0.001	0.00E+00	2.78E-72	0.193	0.107	0.001	8.96E-68
TGFB3	2.61E-24	0.156	0.415	0.094	8.40E-20	2.66E-06	0.205	0.170	0.094	8.56E-02	1.21E-04	0.179	0.207	0.094	1.00E+00	5.33E-07	0.233	0.304	0.094	1.72E-02
THY1	1.14E-46	2.011	0.943	0.485	3.68E-42	1.58E-107	2.162	0.830	0.485	5.10E-103	1.01E-47	1.861	0.946	0.485	3.24E-43	1.95E-21	1.710	0.946	0.485	6.30E-17
TIMP1	9.42E-48	3.138	0.962	0.855	3.03E-43	3.14E-32	0.757	0.883	0.855	1.01E-27	1.07E-26	2.474	0.946	0.855	3.44E-22	1.46E-05	0.821	0.964	0.855	4.70E-01
TMEM123	1.30E-20	0.376	0.660	0.224	4.20E-16	4.66E-35	0.803	0.489	0.224	1.50E-30	7.13E-22	0.594	0.622	0.224	2.30E-17	2.03E-14	0.501	0.679	0.224	6.54E-10
TMEM173	1.23E-48	1.238	0.755	0.210	3.97E-44	1.85E-08	0.383	0.330	0.210	5.97E-04	1.52E-09	0.244	0.468	0.210	4.91E-05	4.50E-11	0.408	0.607	0.210	1.45E-06
TMEM176A	7.36E-40	1.412	0.774	0.267	2.37E-35	1.07E-36	0.955	0.545	0.267	3.44E-32	2.64E-38	1.191	0.793	0.267	8.52E-34	2.01E-31	1.609	0.875	0.267	6.46E-27
TMEM176B	2.24E-40	1.720	0.830	0.339	7.22E-36	1.08E-48	1.230	0.631	0.339	3.47E-44	1.14E-43	1.688	0.838	0.339	3.69E-39	2.32E-26	1.707	0.893	0.339	7.46E-22
TNC	8.24E-194	3.205	0.906	0.084	2.65E-189	0.00E+00	3.263	0.704	0.084	0.00E+00	9.05E-198	3.257	0.892	0.084	2.92E-193	4.91E-99	2.519	0.875	0.084	1.58E-94
TNFRSF21	1.49E-23	0.194	0.160	0.019	4.80E-19	2.72E-164	0.729	0.265	0.019	8.77E-160	4.73E-224	1.349	0.495	0.019	1.52E-219	1.85E-20	0.199	0.196	0.019	5.96E-16
TNFRSF6B	7.64E-228	1.766	0.717	0.041	2.46E-223	3.76E-174	1.338	0.383	0.041	1.21E-169	2.79E-67	0.930	0.387	0.041	8.98E-63	9.34E-66	1.077	0.518	0.041	3.01E-61
TNIP1	1.22E-22	0.541	0.585	0.189	3.91E-18	9.53E-09	0.405	0.304	0.189	3.07E-04	4.09E-07	0.236	0.387	0.189	1.32E-02	1.20E-18	0.671	0.661	0.189	3.86E-14
TYMP	1.08E-58	2.155	0.925	0.406	3.48E-54	1.46E-39	1.144	0.654	0.406	4.71E-35	6.40E-34	1.377	0.838	0.406	2.06E-29	5.41E-08	0.529	0.804	0.406	1.74E-03
UQCR10	1.33E-06	0.310	0.802	0.484	4.29E-02	3.37E-03	0.290	0.584	0.484	1.00E+00	7.68E-16	0.661	0.838	0.484	2.47E-11	2.20E-06	0.553	0.875	0.484	7.08E-02
XAF1	2.64E-103	0.988	0.689	0.079	8.49E-99	2.80E-22	0.328	0.229	0.079	9.01E-18	2.42E-33	0.522	0.405	0.079	7.78E-29	6.26E-28	0.423	0.500	0.079	2.02E-23

	MF18					MF21					MF24				
	p val	avg logFC	pct.1	pct.2	p val adj	p val	avg logFC	pct.1	pct.2	p val adj	p_val	avg logFC	pct.1	pct.2	p val adj
ACOT7	6.12E-29	0.183	0.242	0.030	1.97E-24	8.98E-19	0.393	0.241	0.030	2.89E-14	1.19E-07	1.518	0.333	0.030	3.82E-03
ADAMDEC1	7.01E-178	0.727	0.253	0.004	2.26E-173	0.00E+00	1.434	0.444	0.004	0.00E+00	1.39E-47	1.806	0.333	0.004	4.49E-43
ADIRF	6.71E-38	-3.369	0.341	0.849	2.16E-33	8.28E-25	-3.683	0.204	0.849	2.67E-20	3.99E-05	-3.496	0.333	0.849	1.00E+00
APOL1	7.97E-123	0.993	0.538	0.037	2.57E-118	1.73E-39	0.604	0.389	0.037	5.56E-35	1.80E-06	0.827	0.333	0.037	5.80E-02
APOL6	1.59E-27	0.427	0.396	0.076	5.12E-23	5.36E-04	0.242	0.204	0.076	1.00E+00	5.70E-03	0.170	0.333	0.076	1.00E+00
ARID5A	3.11E-47	0.759	0.473	0.068	1.00E-42	2.46E-27	0.870	0.444	0.068	7.92E-23	2.35E-03	0.263	0.333	0.068	1.00E+00
ARPC5L	8.54E-11	0.222	0.363	0.121	2.75E-06	7.85E-09	0.467	0.389	0.121	2.53E-04	6.56E-03	0.195	0.444	0.121	1.00E+00
ARSA	2.87E-23	0.381	0.363	0.073	9.24E-19	1.64E-08	0.564	0.278	0.073	5.29E-04	4.40E-03	0.139	0.333	0.073	1.00E+00
ATOX1	1.49E-04	0.327	0.626	0.390	1.00E+00	7.17E-05	0.666	0.611	0.390	1.00E+00	1.08E-02	0.837	0.778	0.390	1.00E+00
B2M	4.70E-16	1.033	0.956	0.987	1.51E-11	1.13E-29	1.481	1.000	0.987	3.64E-25	1.94E-02	0.434	1.000	0.987	1.00E+00
BAX	1.80E-04	0.200	0.429	0.222	1.00E+00	5.80E-10	0.827	0.556	0.222	1.87E-05	2.97E-03	0.655	0.667	0.222	1.00E+00
BCAR1	4.78E-45	0.611	0.440	0.063	1.54E-40	2.23E-03	0.129	0.167	0.063	1.00E+00	1.24E-03	0.239	0.333	0.063	1.00E+00
BIRC3	1.30E-21	0.918	0.363	0.080	4.18E-17	1.98E-24	1.104	0.463	0.080	6.38E-20	4.65E-10	0.639	0.667	0.080	1.50E-05
BMP1	5.17E-62	1.385	0.681	0.116	1.67E-57	1.80E-12	0.798	0.426	0.116	5.79E-08	1.37E-03	0.701	0.444	0.116	1.00E+00
BST2	6.87E-68	1.441	0.604	0.084	2.21E-63	1.81E-38	2.132	0.556	0.084	5.83E-34	2.83E-10	1.660	0.667	0.084	9.12E-06
BTN3A2	7.08E-30	0.562	0.407	0.075	2.28E-25	1.64E-05	0.210	0.241	0.075	5.27E-01	4.82E-03	0.240	0.333	0.075	1.00E+00
C1QTNF6	1.24E-77	1.028	0.549	0.061	4.00E-73	1.88E-03	0.240	0.167	0.061	1.00E+00	8.29E-04	0.341	0.333	0.061	1.00E+00
CCL19	9.53E-25	1.942	0.341	0.066	3.07E-20	2.23E-116	5.414	0.833	0.066	7.18E-112	7.81E-14	3.238	0.667	0.066	2.52E-09
CCM2	5.31E-13	0.284	0.352	0.103	1.71E-08	3.84E-04	0.196	0.259	0.103	1.00E+00	3.31E-02	0.182	0.333	0.103	1.00E+00
CD40	1.98E-51	0.931	0.571	0.095	6.38E-47	2.71E-31	1.477	0.537	0.095	8.72E-27	1.29E-02	0.465	0.333	0.095	1.00E+00
CD74	2.39E-65	3.405	0.714	0.132	7.69E-61	1.10E-88	4.205	0.963	0.132	3.55E-84	9.52E-05	1.935	0.556	0.132	1.00E+00
CD82	2.82E-78	1.141	0.626	0.081	9.09E-74	8.15E-78	2.839	0.741	0.081	2.62E-73	2.68E-08	2.007	0.556	0.081	8.64E-04
CFD	2.91E-52	-6.205	0.110	0.934	9.38E-48	1.61E-31	-5.923	0.111	0.934	5.19E-27	6.70E-06	-4.384	0.222	0.934	2.16E-01
CFH	1.10E-06	-1.005	0.747	0.769	3.53E-02	6.69E-13	-2.107	0.463	0.769	2.16E-08	3.32E-03	-2.158	0.444	0.769	1.00E+00
CHN1	3.44E-37	0.604	0.363	0.051	1.11E-32	9.75E-15	0.210	0.296	0.051	3.14E-10	1.40E-11	1.006	0.556	0.051	4.50E-07
CHST11	8.21E-109	0.747	0.429	0.026	2.64E-104	7.09E-08	0.102	0.148	0.026	2.28E-03	2.69E-04	0.297	0.222	0.026	1.00E+00

CHST15	1.48E-114	0.550	0.341	0.015	4.77E-110	1.56E-22	0.230	0.185	0.015	5.02E-18	2.25E-14	0.263	0.333	0.015	7.24E-10
CLSTN3	3.53E-84	1.103	0.626	0.071	1.14E-79	1.12E-15	0.923	0.352	0.071	3.62E-11	3.16E-03	0.333	0.333	0.071	1.00E+00
COL18A1	1.26E-09	0.221	0.659	0.305	4.07E-05	1.00E-02	-0.208	0.537	0.305	1.00E+00	2.34E-02	0.420	0.667	0.305	1.00E+00
COL27A1	8.41E-85	1.367	0.495	0.045	2.71E-80	2.74E-35	0.650	0.407	0.045	8.84E-31	1.60E-08	0.484	0.444	0.045	5.17E-04
COL4A1	1.64E-39	1.314	0.659	0.156	5.29E-35	8.14E-09	0.733	0.444	0.156	2.62E-04	1.18E-05	1.421	0.667	0.156	3.79E-01
COL4A2	4.89E-41	1.473	0.758	0.211	1.57E-36	3.16E-14	1.264	0.593	0.211	1.02E-09	5.40E-06	1.324	0.778	0.211	1.74E-01
COL4A4	0.00E+00	1.607	0.549	0.011	0.00E+00	3.68E-115	1.074	0.370	0.011	1.19E-110	3.68E-73	1.436	0.667	0.011	1.19E-68
COL6A3	6.77E-46	2.830	0.967	0.634	2.18E-41	3.49E-05	0.990	0.704	0.634	1.00E+00	1.28E-03	1.307	0.889	0.634	1.00E+00
COL6A5	2.09E-51	0.668	0.319	0.030	6.74E-47	1.33E-35	0.804	0.333	0.030	4.27E-31	1.47E-12	0.664	0.444	0.030	4.73E-08
COL6A6	2.00E-37	0.805	0.352	0.049	6.43E-33	8.19E-04	0.181	0.148	0.049	1.00E+00	1.46E-08	1.437	0.444	0.049	4.70E-04
COX5A	8.96E-08	0.310	0.626	0.325	2.89E-03	8.23E-08	0.957	0.593	0.325	2.65E-03	1.86E-03	0.831	0.778	0.325	1.00E+00
CPE	1.05E-03	-0.821	0.516	0.562	1.00E+00	2.03E-09	-1.752	0.167	0.562	6.53E-05	5.43E-03	-2.153	0.111	0.562	1.00E+00
CSF1	7.11E-32	0.878	0.593	0.145	2.29E-27	2.11E-06	0.477	0.370	0.145	6.79E-02	1.07E-02	0.528	0.444	0.145	1.00E+00
CSRP2	4.40E-08	0.104	0.286	0.097	1.42E-03	6.92E-10	0.578	0.352	0.097	2.23E-05	5.90E-04	0.559	0.444	0.097	1.00E+00
CTSB	8.72E-40	3.176	0.846	0.426	2.81E-35	2.31E-26	2.823	0.852	0.426	7.45E-22	1.95E-03	0.921	0.889	0.426	1.00E+00
CTSC	1.71E-39	1.194	0.637	0.146	5.50E-35	8.49E-38	1.974	0.722	0.146	2.73E-33	8.68E-04	0.521	0.556	0.146	1.00E+00
CXCL16	6.92E-18	0.163	0.297	0.062	2.23E-13	3.17E-04	0.197	0.185	0.062	1.00E+00	7.63E-04	0.758	0.333	0.062	1.00E+00
CYP7B1	1.96E-52	1.155	0.648	0.116	6.31E-48	2.33E-18	0.983	0.500	0.116	7.49E-14	2.59E-05	1.002	0.556	0.116	8.33E-01
DAAM1	1.53E-09	0.241	0.264	0.081	4.93E-05	4.86E-14	0.471	0.370	0.081	1.56E-09	8.00E-03	0.209	0.333	0.081	1.00E+00
DCN	6.23E-20	-1.352	0.934	0.979	2.01E-15	7.26E-10	-1.075	0.944	0.979	2.34E-05	1.75E-04	-2.017	0.778	0.979	1.00E+00
DYNLT1	1.56E-15	0.755	0.604	0.261	5.01E-11	3.10E-12	1.056	0.630	0.261	1.00E-07	7.13E-03	0.637	0.667	0.261	1.00E+00
EDF1	5.10E-03	-0.366	0.725	0.762	1.00E+00	3.63E-04	0.523	0.815	0.762	1.00E+00	6.01E-03	0.864	1.000	0.762	1.00E+00
EFEMP2	1.28E-05	0.481	0.615	0.398	4.12E-01	1.27E-03	0.471	0.556	0.398	1.00E+00	2.12E-02	1.060	0.778	0.398	1.00E+00
ELK3	1.25E-16	0.226	0.363	0.091	4.02E-12	1.66E-02	0.320	0.185	0.091	1.00E+00	5.53E-04	0.266	0.444	0.091	1.00E+00
ELN	7.23E-16	-1.938	0.022	0.471	2.33E-11	1.75E-09	-1.801	0.037	0.471	5.65E-05	2.71E-02	-1.766	0.111	0.471	1.00E+00
EPAS1	2.07E-108	1.285	0.648	0.063	6.67E-104	3.02E-15	0.644	0.333	0.063	9.74E-11	8.85E-04	0.713	0.333	0.063	1.00E+00
EPSTI1	1.85E-107	1.161	0.571	0.049	5.97E-103	2.52E-29	0.648	0.389	0.049	8.12E-25	1.77E-02	0.291	0.222	0.049	1.00E+00
ETHE1	8.31E-10	0.428	0.418	0.173	2.68E-05	6.39E-27	1.831	0.648	0.173	2.06E-22	2.16E-02	0.728	0.444	0.173	1.00E+00
FAM207A	1.37E-14	0.176	0.319	0.080	4.40E-10	2.83E-04	0.241	0.222	0.080	1.00E+00	6.81E-03	0.364	0.333	0.080	1.00E+00
FAM20A	1.19E-64	1.043	0.637	0.097	3.84E-60	2.96E-18	1.013	0.444	0.097	9.53E-14	3.48E-06	0.805	0.556	0.097	1.12E-01
FBLN1	3.32E-16	-1.798	0.725	0.850	1.07E-11	3.64E-15	-2.415	0.611	0.850	1.17E-10	6.45E-03	-1.615	0.667	0.850	1.00E+00
FRMD6	1.37E-44	1.529	0.747	0.196	4.42E-40	1.80E-02	0.561	0.315	0.196	1.00E+00	8.73E-03	0.402	0.556	0.196	1.00E+00
G0S2	4.24E-32	1.658	0.363	0.061	1.37E-27	1.18E-47	1.808	0.537	0.061	3.81E-43	8.78E-04	0.471	0.333	0.061	1.00E+00
GAPDH	5.85E-16	1.324	0.978	0.878	1.89E-11	8.95E-24	1.916	0.981	0.878	2.88E-19	1.46E-02	0.666	1.000	0.878	1.00E+00
GBP2	1.12E-25	0.850	0.538	0.144	3.60E-21	6.39E-04	0.395	0.315	0.144	1.00E+00	1.29E-02	1.261	0.444	0.144	1.00E+00
GGCT	4.54E-30	0.535	0.582	0.139	1.46E-25	2.94E-07	0.478	0.389	0.139	9.46E-03	1.09E-02	0.395	0.444	0.139	1.00E+00
GPR137	4.16E-22	0.332	0.407	0.093	1.34E-17	3.23E-13	0.542	0.389	0.093	1.04E-08	2.08E-02	0.153	0.333	0.093	1.00E+00
GSN	1.38E-47	-4.119	0.604	0.944	4.44E-43	2.28E-29	-4.220	0.481	0.944	7.34E-25	3.89E-04	-2.285	0.667	0.944	1.00E+00
GUCY1A1	5.42E-119	0.686	0.363	0.017	1.74E-114	4.14E-58	0.835	0.315	0.017	1.33E-53	4.02E-50	1.341	0.667	0.017	1.30E-45
GUCY1B1	2.74E-123	0.604	0.363	0.016	8.82E-119	5.33E-83	0.500	0.370	0.016	1.72E-78	1.01E-06	0.399	0.222	0.016	3.24E-02
H2AFY2	6.78E-93	0.292	0.253	0.010	2.18E-88	8.58E-48	0.273	0.222	0.010	2.76E-43	2.51E-03	0.273	0.111	0.010	1.00E+00
HDDC2	4.28E-07	0.126	0.429	0.184	1.38E-02	6.89E-05	0.454	0.407	0.184	1.00E+00	5.28E-03	1.395	0.556	0.184	1.00E+00
HEG1	1.12E-50	0.695	0.396	0.047	3.60E-46	1.94E-09	0.390	0.222	0.047	6.26E-05	1.33E-02	0.272	0.222	0.047	1.00E+00
HES4	4.91E-69	1.006	0.495	0.054	1.58E-64	1.35E-25	0.641	0.389	0.054	4.33E-21	1.11E-10	0.707	0.556	0.054	3.58E-06
HIF1A	7.01E-55	1.075	0.648	0.116	2.26E-50	1.48E-03	0.170	0.259	0.116	1.00E+00	4.60E-02	0.543	0.333	0.116	1.00E+00
HLA-A	7.65E-15	1.066	0.901	0.785	2.46E-10	8.34E-33	2.372	1.000	0.785	2.68E-28	3.61E-03	1.511	0.889	0.785	1.00E+00
HLA-B	5.67E-42	2.521	0.934	0.676	1.83E-37	4.10E-33	2.338	1.000	0.676	1.32E-28	2.61E-06	2.169	1.000	0.676	8.42E-02
HLA-C	1.35E-05	0.455	0.890	0.782	4.35E-01	3.75E-26	1.852	0.963	0.782	1.21E-21	3.52E-03	1.053	1.000	0.782	1.00E+00
HLA-E	2.31E-08	0.633	0.780	0.569	7.44E-04	3.60E-06	0.787	0.722	0.569	1.16E-01	4.54E-02	0.816	0.778	0.569	1.00E+00
HLA-F	7.36E-84	1.244	0.648	0.080	2.37E-79	6.68E-53	1.462	0.648	0.080	2.15E-48	3.32E-07	0.703	0.556	0.080	1.07E-02
IDH2	1.02E-12	0.295	0.352	0.105	3.28E-08	1.30E-06	0.464	0.315	0.105	4.18E-02	8.80E-04	0.756	0.444	0.105	1.00E+00

IFI27	3.49E-29	1.157	0.692	0.213	1.13E-24	8.04E-26	1.914	0.741	0.213	2.59E-21	2.66E-05	2.047	0.778	0.213	8.58E-01
IFI30	2.76E-195	1.602	0.648	0.034	8.89E-191	1.98E-193	2.709	0.796	0.034	6.37E-189	2.82E-11	0.642	0.444	0.034	9.07E-07
IFITM1	2.38E-36	2.557	0.846	0.471	7.67E-32	1.83E-39	3.009	1.000	0.471	5.90E-35	4.51E-02	0.608	0.778	0.471	1.00E+00
IFITM3	2.37E-28	1.787	0.934	0.881	7.63E-24	1.04E-30	2.044	1.000	0.881	3.36E-26	1.10E-02	0.597	1.000	0.881	1.00E+00
IKBIP	5.63E-17	0.458	0.505	0.164	1.81E-12	3.51E-07	0.435	0.426	0.164	1.13E-02	1.82E-02	0.570	0.444	0.164	1.00E+00
IL1R1	1.32E-48	1.152	0.648	0.128	4.24E-44	1.90E-07	0.662	0.370	0.128	6.12E-03	3.74E-04	0.407	0.556	0.128	1.00E+00
IL32	5.31E-121	2.735	0.758	0.086	1.71E-116	7.85E-108	3.822	0.889	0.086	2.53E-103	8.30E-03	0.569	0.333	0.086	1.00E+00
IL4I1	9.22E-198	0.466	0.286	0.005	2.97E-193	3.17E-276	1.050	0.426	0.005	1.02E-271	2.32E-41	0.710	0.333	0.005	7.48E-37
IRF8	2.49E-137	0.376	0.297	0.009	8.03E-133	3.45E-19	0.296	0.130	0.009	1.11E-14	1.05E-23	0.413	0.333	0.009	3.37E-19
IRF9	2.92E-32	0.860	0.692	0.187	9.41E-28	2.64E-04	0.331	0.389	0.187	1.00E+00	4.03E-05	0.506	0.778	0.187	1.00E+00
KLF4	9.89E-06	-1.217	0.429	0.541	3.19E-01	3.41E-04	-1.143	0.370	0.541	1.00E+00	3.20E-02	-1.576	0.222	0.541	1.00E+00
KPNA2	2.28E-12	0.446	0.297	0.082	7.34E-08	6.76E-08	0.375	0.296	0.082	2.18E-03	1.14E-04	0.586	0.444	0.082	1.00E+00
LAP3	7.77E-54	1.629	0.692	0.151	2.50E-49	8.61E-23	1.393	0.593	0.151	2.77E-18	2.79E-04	0.780	0.556	0.151	1.00E+00
LGALS3BP	3.61E-16	1.105	0.648	0.293	1.16E-11	7.01E-14	1.222	0.685	0.293	2.26E-09	2.65E-02	1.085	0.556	0.293	1.00E+00
LGALS9	3.00E-39	0.386	0.352	0.046	9.67E-35	1.85E-11	0.526	0.241	0.046	5.95E-07	4.09E-13	0.809	0.556	0.046	1.32E-08
LMO4	1.29E-16	0.634	0.615	0.239	4.16E-12	1.13E-03	0.517	0.407	0.239	1.00E+00	1.79E-04	1.085	0.778	0.239	1.00E+00
LOXL2	6.63E-77	1.108	0.604	0.074	2.13E-72	9.66E-37	1.600	0.519	0.074	3.11E-32	2.72E-03	0.510	0.333	0.074	1.00E+00
LPCAT3	4.77E-16	0.197	0.253	0.051	1.54E-11	7.26E-13	0.229	0.278	0.051	2.34E-08	2.49E-02	0.150	0.222	0.051	1.00E+00
MDK	8.27E-10	0.640	0.571	0.256	2.66E-05	1.41E-02	0.221	0.426	0.256	1.00E+00	8.14E-04	1.450	0.667	0.256	1.00E+00
MFAP4	1.26E-29	-2.646	0.396	0.818	4.06E-25	3.48E-13	-1.794	0.574	0.818	1.12E-08	6.57E-03	-1.741	0.667	0.818	1.00E+00
MGP	1.38E-26	-3.025	0.440	0.815	4.45E-22	5.33E-19	-2.526	0.352	0.815	1.72E-14	3.24E-02	-0.549	0.556	0.815	1.00E+00
MIF	1.29E-13	1.277	0.813	0.763	4.17E-09	2.24E-23	1.906	0.944	0.763	7.21E-19	1.13E-02	1.049	1.000	0.763	1.00E+00
MIR4435-2HG	2.70E-83	1.568	0.681	0.095	8.68E-79	1.70E-06	0.312	0.296	0.095	5.49E-02	9.17E-07	1.464	0.556	0.095	2.95E-02
MRPL36	5.19E-08	0.158	0.462	0.195	1.67E-03	8.03E-08	0.735	0.463	0.195	2.59E-03	1.09E-03	0.451	0.667	0.195	1.00E+00
MSC	1.19E-50	0.813	0.484	0.068	3.82E-46	6.10E-23	0.835	0.407	0.068	1.96E-18	5.71E-09	0.919	0.556	0.068	1.84E-04
MT-ATP8	1.12E-56	2.577	0.945	0.330	3.61E-52	1.73E-32	1.915	0.963	0.330	5.57E-28	1.34E-03	1.027	0.778	0.330	1.00E+00
MT-ND6	5.24E-48	2.161	0.857	0.242	1.69E-43	3.10E-05	1.294	0.500	0.242	9.99E-01	2.21E-04	0.748	0.778	0.242	1.00E+00
MYH9	1.98E-41	1.259	0.736	0.197	6.39E-37	3.08E-13	0.906	0.593	0.197	9.93E-09	3.41E-03	1.105	0.556	0.197	1.00E+00
NDFIP1	8.23E-09	-1.018	0.582	0.659	2.65E-04	8.01E-05	-0.718	0.519	0.659	1.00E+00	4.50E-02	-1.170	0.444	0.659	1.00E+00
NFKB2	1.48E-39	0.693	0.527	0.098	4.78E-35	1.91E-09	0.436	0.352	0.098	6.15E-05	6.51E-04	0.540	0.444	0.098	1.00E+00
NNMT	1.15E-12	0.935	0.813	0.587	3.70E-08	3.09E-16	1.535	0.870	0.587	9.94E-12	4.40E-02	0.424	1.000	0.587	1.00E+00
NR4A1	1.40E-02	-0.816	0.341	0.445	1.00E+00	1.51E-06	-1.935	0.130	0.445	4.86E-02	3.54E-02	-2.109	0.111	0.445	1.00E+00
NUB1	2.14E-15	0.430	0.462	0.143	6.88E-11	6.45E-07	0.619	0.389	0.143	2.08E-02	5.81E-03	1.537	0.444	0.143	1.00E+00
OSBPL8	2.48E-07	0.210	0.473	0.213	7.97E-03	2.02E-03	0.451	0.389	0.213	1.00E+00	4.40E-04	0.893	0.667	0.213	1.00E+00
OSMR	5.83E-63	0.876	0.637	0.096	1.88E-58	2.76E-17	0.779	0.444	0.096	8.87E-13	2.30E-02	0.313	0.333	0.096	1.00E+00
OSTC	1.35E-05	0.429	0.659	0.463	4.34E-01	2.45E-02	0.292	0.574	0.463	1.00E+00	4.50E-02	0.891	0.778	0.463	1.00E+00
P4HB	1.68E-05	0.703	0.725	0.548	5.42E-01	3.53E-05	0.803	0.704	0.548	1.00E+00	4.74E-02	0.843	0.889	0.548	1.00E+00
PAPSS2	7.27E-64	0.693	0.363	0.031	2.34E-59	2.59E-10	0.331	0.185	0.031	8.35E-06	1.36E-03	0.193	0.222	0.031	1.00E+00
PARP14	5.41E-34	0.807	0.505	0.101	1.74E-29	6.35E-07	0.524	0.315	0.101	2.05E-02	1.96E-02	0.463	0.333	0.101	1.00E+00
PDGFRL	4.81E-10	-1.442	0.154	0.441	1.55E-05	6.46E-08	-1.165	0.056	0.441	2.08E-03	3.16E-02	-1.532	0.111	0.441	1.00E+00
PFN1	3.05E-03	0.517	0.769	0.754	1.00E+00	5.72E-27	1.821	0.963	0.754	1.84E-22	6.63E-03	1.130	1.000	0.754	1.00E+00
PLAAT4	2.99E-13	0.482	0.582	0.230	9.65E-09	2.13E-07	0.903	0.500	0.230	6.86E-03	1.66E-03	0.933	0.667	0.230	1.00E+00
PLAC9	1.47E-29	-2.157	0.659	0.855	4.75E-25	1.99E-18	-2.178	0.519	0.855	6.42E-14	1.43E-02	-1.141	0.556	0.855	1.00E+00
PLEKHO1	4.73E-41	0.484	0.407	0.058	1.52E-36	1.57E-08	0.434	0.241	0.058	5.05E-04	4.19E-02	0.140	0.222	0.058	1.00E+00
PLSCR1	1.51E-25	1.011	0.648	0.207	4.87E-21	3.34E-09	0.852	0.519	0.207	1.08E-04	2.83E-02	0.299	0.556	0.207	1.00E+00
PLXND1	7.82E-29	0.789	0.484	0.106	2.52E-24	2.50E-06	0.368	0.315	0.106	8.04E-02	2.96E-02	0.341	0.333	0.106	1.00E+00
PPIB	2.42E-09	0.881	0.835	0.730	7.78E-05	4.77E-11	0.992	0.852	0.730	1.54E-06	9.17E-04	1.014	1.000	0.730	1.00E+00
PRSS23	1.38E-85	3.109	0.890	0.188	4.45E-81	2.63E-31	3.142	0.722	0.188	8.47E-27	6.25E-05	1.372	0.667	0.188	1.00E+00
PSMB5	2.04E-02	0.187	0.549	0.368	1.00E+00	1.48E-02	0.377	0.537	0.368	1.00E+00	3.19E-02	0.453	0.778	0.368	1.00E+00
PSMB9	7.60E-31	1.123	0.626	0.185	2.45E-26	5.01E-18	1.190	0.611	0.185	1.61E-13	8.13E-04	0.947	0.667	0.185	1.00E+00
RAB13	2.01E-11	0.769	0.714	0.414	6.47E-07	7.52E-21	1.777	0.833	0.414	2.42E-16	2.87E-03	1.937	0.778	0.414	1.00E+00

RARRES2	1.35E-13	1.095	0.692	0.344	4.36E-09	1.62E-14	1.699	0.741	0.344	5.22E-10	8.42E-06	1.431	1.000	0.344	2.71E-01
RBP5	2.36E-72	0.690	0.429	0.039	7.61E-68	5.72E-76	1.569	0.537	0.039	1.84E-71	6.13E-31	2.334	0.778	0.039	1.97E-26
RPL18	6.47E-10	-0.778	0.802	0.905	2.08E-05	8.98E-05	0.446	0.963	0.905	1.00E+00	2.36E-02	0.680	1.000	0.905	1.00E+00
RPL22L1	6.60E-26	1.350	0.736	0.312	2.13E-21	6.74E-17	1.578	0.722	0.312	2.17E-12	3.98E-02	1.067	0.556	0.312	1.00E+00
RPL41	9.86E-49	-2.416	0.901	0.985	3.18E-44	1.37E-04	-0.411	1.000	0.985	1.00E+00	4.88E-04	0.732	1.000	0.985	1.00E+00
RPS4Y1	1.23E-12	0.382	0.648	0.253	3.96E-08	4.57E-12	0.777	0.704	0.253	1.47E-07	1.42E-06	1.666	0.889	0.253	4.59E-02
S100A4	3.08E-07	-0.779	0.835	0.931	9.93E-03	3.57E-02	-0.433	0.907	0.931	1.00E+00	1.00E-02	-1.347	0.778	0.931	1.00E+00
SELENOP	7.83E-34	-2.485	0.176	0.822	2.52E-29	1.28E-19	-2.365	0.204	0.822	4.12E-15	1.22E-03	-1.908	0.556	0.822	1.00E+00
SEMA5A	4.08E-58	0.691	0.451	0.053	1.32E-53	2.75E-04	0.165	0.167	0.053	1.00E+00	1.55E-11	0.938	0.556	0.053	4.99E-07
SLFN5	6.64E-28	0.677	0.495	0.111	2.14E-23	3.80E-02	0.175	0.204	0.111	1.00E+00	3.64E-02	0.282	0.333	0.111	1.00E+00
SLIT3	2.03E-19	-1.995	0.253	0.641	6.54E-15	2.10E-15	-2.139	0.037	0.641	6.77E-11	1.91E-02	-1.660	0.444	0.641	1.00E+00
SNHG5	4.05E-07	-1.048	0.692	0.737	1.31E-02	4.86E-11	-1.716	0.463	0.737	1.56E-06	2.60E-02	-1.462	0.667	0.737	1.00E+00
SOD3	2.59E-19	-1.317	0.780	0.898	8.34E-15	3.36E-04	-0.567	0.852	0.898	1.00E+00	1.12E-04	-2.301	0.556	0.898	1.00E+00
SPCS3	6.27E-24	0.432	0.505	0.125	2.02E-19	3.50E-07	0.264	0.370	0.125	1.13E-02	3.56E-03	0.649	0.444	0.125	1.00E+00
SPHK1	0.00E+00	1.830	0.681	0.020	0.00E+00	1.35E-84	1.177	0.407	0.020	4.34E-80	8.21E-20	1.086	0.444	0.020	2.65E-15
SQLE	2.53E-20	0.274	0.264	0.047	8.16E-16	2.14E-09	0.493	0.222	0.047	6.90E-05	1.59E-02	0.128	0.222	0.047	1.00E+00
STAT1	7.38E-83	1.838	0.747	0.116	2.38E-78	3.42E-09	0.916	0.370	0.116	1.10E-04	2.65E-03	0.665	0.444	0.116	1.00E+00
STK17A	4.74E-60	0.920	0.473	0.056	1.53E-55	1.66E-22	0.585	0.370	0.056	5.35E-18	3.14E-02	0.608	0.222	0.056	1.00E+00
SULF1	0.00E+00	3.513	0.791	0.028	0.00E+00	5.65E-36	1.064	0.315	0.028	1.82E-31	4.37E-08	0.494	0.333	0.028	1.41E-03
SVEP1	1.98E-07	-1.191	0.143	0.386	6.37E-03	8.12E-08	-1.487	0.019	0.386	2.62E-03	2.21E-02	-1.546	0.000	0.386	1.00E+00
TAF9	1.27E-02	-0.158	0.297	0.163	1.00E+00	2.24E-06	0.395	0.407	0.163	7.23E-02	1.92E-03	0.542	0.556	0.163	1.00E+00
TAP1	1.45E-54	0.808	0.538	0.081	4.68E-50	1.28E-17	0.547	0.407	0.081	4.12E-13	1.61E-04	0.253	0.444	0.081	1.00E+00
TDO2	0.00E+00	1.264	0.418	0.001	0.00E+00	0.00E+00	0.955	0.370	0.001	0.00E+00	5.60E-19	0.306	0.111	0.001	1.80E-14
TGFB3	1.63E-22	0.475	0.407	0.094	5.26E-18	7.36E-22	0.900	0.481	0.094	2.37E-17	6.22E-04	0.347	0.444	0.094	1.00E+00
THY1	8.32E-10	0.868	0.725	0.485	2.68E-05	3.50E-18	2.261	0.815	0.485	1.13E-13	2.17E-03	1.460	0.778	0.485	1.00E+00
TIMP1	2.37E-33	3.439	0.934	0.855	7.62E-29	8.83E-18	2.933	0.981	0.855	2.84E-13	1.33E-03	2.755	1.000	0.855	1.00E+00
TMEM123	6.32E-10	0.355	0.538	0.224	2.04E-05	7.70E-06	0.601	0.463	0.224	2.48E-01	7.11E-04	0.916	0.667	0.224	1.00E+00
TMEM173	1.13E-22	0.813	0.626	0.210	3.64E-18	4.89E-05	0.591	0.444	0.210	1.00E+00	7.40E-03	1.592	0.556	0.210	1.00E+00
TMEM176A	2.02E-17	0.943	0.637	0.267	6.50E-13	1.70E-31	2.152	0.852	0.267	5.49E-27	3.97E-03	1.775	0.667	0.267	1.00E+00
TMEM176B	1.14E-18	1.336	0.703	0.339	3.68E-14	2.74E-24	2.099	0.833	0.339	8.83E-20	3.68E-03	1.021	0.778	0.339	1.00E+00
TNC	1.29E-114	3.228	0.747	0.084	4.15E-110	8.97E-60	2.677	0.685	0.084	2.89E-55	5.41E-11	2.265	0.667	0.084	1.74E-06
TNFRSF21	5.72E-32	0.478	0.198	0.019	1.84E-27	4.89E-51	0.741	0.315	0.019	1.57E-46	1.26E-05	0.222	0.222	0.019	4.06E-01
TNFRSF6B	1.68E-118	1.692	0.549	0.041	5.40E-114	8.27E-133	2.484	0.722	0.041	2.66E-128	3.50E-10	2.051	0.444	0.041	1.13E-05
TNIP1	2.61E-09	0.456	0.451	0.189	8.41E-05	2.57E-05	0.717	0.389	0.189	8.29E-01	1.18E-04	0.787	0.667	0.189	1.00E+00
TYMP	5.20E-12	0.666	0.747	0.406	1.67E-07	3.73E-34	2.489	0.944	0.406	1.20E-29	1.90E-02	0.546	0.778	0.406	1.00E+00
UQCR10	1.51E-04	0.241	0.714	0.484	1.00E+00	1.72E-02	0.378	0.630	0.484	1.00E+00	1.51E-02	0.516	0.889	0.484	1.00E+00
XAF1	2.17E-65	1.499	0.582	0.079	6.99E-61	3.24E-14	0.559	0.370	0.079	1.04E-09	6.31E-03	0.275	0.333	0.079	1.00E+00

Table S5. Differentially expressed genes in endothelial cells that are common among the MF patient samples.

	MF6					MF8					MF12					MF17				
	p val	avg logFC	pct.1	pct.2	p val adj	p val	avg logFC	pct.1	pct.2	p val adj	p_val	avg logFC	pct.1	pct.2	p val adj	p_val	avg logFC	pct.1	pct.2	p val adj
A2M	3.01E-03	0.352	0.847	0.808	1.00E+00	3.34E-03	0.298	0.790	0.808	1.00E+00	2.30E-04	0.255	0.857	0.808	1.00E+00	3.85E-02	0.889	0.857	0.808	1.00E+00
ABRACL	1.44E-31	0.915	0.557	0.175	4.64E-27	4.14E-12	0.589	0.370	0.175	1.33E-07	4.31E-20	0.533	0.414	0.175	1.39E-15	2.65E-04	0.628	0.714	0.175	1.00E+00
ACOT7	3.92E-50	0.479	0.374	0.049	1.26E-45	1.97E-43	0.597	0.321	0.049	6.35E-39	1.76E-20	0.326	0.207	0.049	5.67E-16	4.33E-03	0.131	0.286	0.049	1.00E+00
ACTB	1.55E-11	0.725	0.985	0.934	5.00E-07	7.41E-03	0.136	0.944	0.934	1.00E+00	1.10E-09	0.436	0.980	0.934	3.55E-05	2.95E-01	0.245	1.000	0.934	1.00E+00
ACVRL1	1.78E-09	0.482	0.565	0.349	5.74E-05	5.55E-05	0.375	0.463	0.349	1.00E+00	1.07E-09	0.498	0.502	0.349	3.44E-05	1.61E-03	0.780	0.857	0.349	1.00E+00
AL583785.1	4.44E-12	0.833	0.466	0.223	1.43E-07	4.78E-03	0.554	0.296	0.223	1.00E+00	3.64E-02	0.191	0.281	0.223	1.00E+00	1.91E-01	0.330	0.429	0.223	1.00E+00
ANAPC11	1.61E-06	0.333	0.702	0.516	5.20E-02	2.34E-06	0.427	0.617	0.516	7.52E-02	3.88E-05	0.322	0.596	0.516	1.00E+00	1.21E-01	0.437	0.714	0.516	1.00E+00
ANKRD65	7.51E-14	0.271	0.252	0.071	2.42E-09	4.38E-17	0.409	0.253	0.071	1.41E-12	6.12E-08	0.234	0.172	0.071	1.97E-03	7.89E-11	1.210	0.714	0.071	2.54E-06
ANTKMT	1.24E-07	0.203	0.298	0.136	3.99E-03	2.34E-05	0.351	0.247	0.136	7.53E-01	1.63E-03	0.131	0.207	0.136	1.00E+00	2.64E-02	0.495	0.429	0.136	1.00E+00
AP2S1	3.10E-29	1.228	0.779	0.495	1.00E-24	1.13E-09	0.700	0.605	0.495	3.64E-05	1.20E-21	0.820	0.680	0.495	3.85E-17	9.85E-04	0.723	1.000	0.495	1.00E+00
ARF3	9.53E-11	0.442	0.511	0.278	3.07E-06	4.74E-07	0.392	0.438	0.278	1.53E-02	4.17E-06	0.415	0.389	0.278	1.34E-01	5.89E-03	0.387	0.714	0.278	1.00E+00
ARHGDIB	3.85E-14	0.835	0.763	0.496	1.24E-09	1.72E-05	0.551	0.580	0.496	5.55E-01	3.82E-07	0.482	0.631	0.496	1.23E-02	6.66E-03	0.854	0.857	0.496	1.00E+00
ARPC1A	9.38E-04	0.311	0.580	0.461	1.00E+00	2.16E-02	0.212	0.525	0.461	1.00E+00	2.53E-04	0.318	0.547	0.461	1.00E+00	1.20E-01	0.389	0.714	0.461	1.00E+00
ARPC3	7.13E-23	0.932	0.863	0.684	2.30E-18	5.42E-21	0.801	0.809	0.684	1.75E-16	2.48E-11	0.496	0.759	0.684	7.97E-07	4.66E-02	0.424	0.857	0.684	1.00E+00
ATOX1	2.27E-15	0.631	0.786	0.587	7.31E-11	2.91E-03	0.363	0.642	0.587	1.00E+00	1.09E-24	0.879	0.768	0.587	3.51E-20	1.02E-02	1.107	0.857	0.587	1.00E+00
ATP5F1D	7.20E-04	0.250	0.771	0.637	1.00E+00	1.92E-08	0.620	0.784	0.637	6.19E-04	2.37E-13	0.608	0.759	0.637	7.64E-09	2.86E-02	0.451	1.000	0.637	1.00E+00
ATP5IF1	4.27E-10	0.544	0.794	0.590	1.37E-05	8.97E-09	0.544	0.716	0.590	2.89E-04	4.91E-09	0.484	0.709	0.590	1.58E-04	1.38E-01	0.194	0.857	0.590	1.00E+00
ATP5MF	5.68E-18	0.676	0.756	0.445	1.83E-13	5.28E-12	0.694	0.660	0.445	1.70E-07	3.86E-37	1.029	0.754	0.445	1.24E-32	1.09E-01	0.410	0.714	0.445	1.00E+00
ATP6V1E1	8.68E-09	0.355	0.565	0.326	2.80E-04	3.51E-06	0.296	0.475	0.326	1.13E-01	1.22E-05	0.180	0.458	0.326	3.92E-01	2.09E-03	0.770	0.857	0.326	1.00E+00
AURKAIP1	1.91E-09	0.563	0.672	0.475	6.15E-05	4.27E-13	0.758	0.673	0.475	1.38E-08	9.30E-11	0.546	0.616	0.475	2.99E-06	1.66E-01	0.150	0.714	0.475	1.00E+00
BAD	9.44E-05	0.202	0.511	0.350	1.00E+00	8.50E-09	0.526	0.512	0.350	2.74E-04	1.07E-06	0.353	0.473	0.350	3.44E-02	5.57E-02	0.459	0.714	0.350	1.00E+00
BAX	1.35E-09	0.569	0.588	0.346	4.36E-05	2.53E-08	0.486	0.506	0.346	8.13E-04	4.95E-17	0.759	0.567	0.346	1.59E-12	3.34E-03	0.909	0.714	0.346	1.00E+00
BCAP31	4.84E-05	0.226	0.672	0.527	1.00E+00	7.66E-08	0.470	0.648	0.527	2.47E-03	1.44E-08	0.444	0.650	0.527	4.62E-04	2.29E-01	0.102	0.714	0.527	1.00E+00
BEX3	8.34E-20	0.888	0.672	0.377	2.68E-15	1.33E-04	0.373	0.469	0.377	1.00E+00	9.69E-21	0.788	0.606	0.377	3.12E-16	9.51E-03	0.740	0.714	0.377	1.00E+00
C17orf49	6.90E-05	0.345	0.672	0.545	1.00E+00	9.72E-03	0.173	0.599	0.545	1.00E+00	2.59E-12	0.475	0.709	0.545	8.35E-08	1.99E-03	0.621	1.000	0.545	1.00E+00
C4orf48	1.45E-44	0.505	0.366	0.052	4.68E-40	1.31E-17	0.420	0.216	0.052	4.24E-13	6.34E-55	0.910	0.345	0.052	2.04E-50	1.23E-05	0.530	0.429	0.052	3.96E-01
CALR	3.76E-14	0.812	0.794	0.657	1.21E-09	4.27E-10	0.664	0.753	0.657	1.38E-05	5.79E-13	0.637	0.764	0.657	1.86E-08	2.58E-03	1.157	1.000	0.657	1.00E+00
CAMK1D	1.33E-05	0.174	0.244	0.119	4.27E-01	1.93E-06	0.164	0.241	0.119	6.22E-02	1.41E-07	0.286	0.236	0.119	4.54E-03	1.49E-04	0.384	0.571	0.119	1.00E+00
CARD16	2.96E-05	0.206	0.458	0.302	9.53E-01	1.18E-01	0.122	0.340	0.302	1.00E+00	6.03E-11	0.466	0.493	0.302	1.94E-06	6.53E-05	1.125	0.857	0.302	1.00E+00
CAVIN3	9.95E-25	1.299	0.809	0.592	3.20E-20	6.05E-23	1.177	0.759	0.592	1.95E-18	6.76E-37	1.265	0.818	0.592	2.18E-32	1.77E-01	0.621	0.714	0.592	1.00E+00
CCDC124	1.61E-02	0.161	0.443	0.338	1.00E+00	2.61E-02	0.279	0.395	0.338	1.00E+00	2.64E-03	0.283	0.399	0.338	1.00E+00	7.09E-04	0.783	0.857	0.338	1.00E+00
CCDC85B	3.06E-11	0.784	0.885	0.716	9.85E-07	5.11E-08	0.595	0.765	0.716	1.65E-03	4.60E-24	0.863	0.842	0.716	1.48E-19	7.17E-03	0.813	1.000	0.716	1.00E+00
CDC42EP1	3.34E-07	0.237	0.511	0.297	1.08E-02	7.82E-01	0.178	0.290	0.297	1.00E+00	1.70E-01	0.180	0.320	0.297	1.00E+00	2.86E-02	0.758	0.571	0.297	1.00E+00
CDIPT	3.04E-03	0.146	0.466	0.346	1.00E+00	2.63E-03	0.165	0.432	0.346	1.00E+00	1.80E-03	0.294	0.419	0.346	1.00E+00	3.80E-02	0.351	0.714	0.346	1.00E+00
CETP	2.47E-04	0.185	0.122	0.050	1.00E+00	3.17E-10	0.220	0.167	0.050	1.02E-05	2.67E-19	0.330	0.202	0.050	8.59E-15	4.15E-03	0.271	0.286	0.050	1.00E+00
CFI	1.89E-10	0.492	0.557	0.322	6.08E-06	4.85E-07	0.466	0.488	0.322	1.56E-02	4.36E-04	0.123	0.424	0.322	1.00E+00	1.51E-01	0.446	0.571	0.322	1.00E+00
CFL1	1.07E-16	0.653	0.901	0.833	3.45E-12	1.25E-14	0.600	0.858	0.833	4.03E-10	4.59E-26	0.663	0.921	0.833	1.48E-21	1.14E-01	0.409	1.000	0.833	1.00E+00
CHMP6	3.05E-02	0.125	0.168	0.107	1.00E+00	8.34E-08	0.189	0.241	0.107	2.69E-03	1.47E-05	0.155	0.202	0.107	4.73E-01	1.23E-01	0.111	0.286	0.107	1.00E+00
CIAO2B	1.44E-06	0.284	0.679	0.474	4.62E-02	5.35E-05	0.364	0.568	0.474	1.00E+00	1.01E-01	0.141	0.507	0.474	1.00E+00	1.21E-01	0.201	0.857	0.474	1.00E+00
CINP	2.06E-11	0.317	0.351	0.140	6.64E-07	1.62E-06	0.219	0.272	0.140	5.23E-02	1.50E-07	0.224	0.266	0.140	4.83E-03	4.15E-02	0.119	0.429	0.140	1.00E+00
CLEC14A	2.22E-06	0.455	0.733	0.616	7.16E-02	1.87E-09	0.674	0.747	0.616	6.04E-05	3.12E-15	0.795	0.764	0.616	1.00E-10	1.29E-02	1.256	0.857	0.616	1.00E+00
CLTB	1.81E-12	0.594	0.725	0.494	5.82E-08	6.63E-10	0.662	0.654	0.494	2.14E-05	3.15E-07	0.337	0.611	0.494	1.01E-02	1.93E-02	0.535	0.857	0.494	1.00E+00
CNDP2	1.48E-17	0.471	0.481	0.190	4.78E-13	9.38E-04	0.283	0.290	0.190	1.00E+00	1.94E-08	0.249	0.340	0.190	6.26E-04	2.39E-04	0.540	0.714	0.190	1.00E+00
CNIH1	2.86E-08	0.536	0.580	0.416	9.22E-04	2.91E-05	0.477	0.543	0.416	9.37E-01	3.28E-14	0.658	0.591	0.416	1.05E-09	3.45E-02	0.439	0.714	0.416	1.00E+00
CNPY4	1.81E-13	0.135	0.252	0.072	5.82E-09	1.76E-08	0.216	0.191	0.072	5.66E-04	1.06E-02	0.111	0.118	0.072	1.00E+00	3.75E-04	0.173	0.429	0.072	1.00E+00

COL15A1	9.86E-06	0.524	0.656	0.568	3.17E-01	3.34E-01	0.203	0.556	0.568	1.00E+00	6.66E-13	0.793	0.695	0.568	2.15E-08	5.26E-02	0.875	0.714	0.568	1.00E+00
COL4A1	3.35E-31	1.500	0.771	0.435	1.08E-26	1.18E-05	0.746	0.549	0.435	3.81E-01	4.36E-23	1.277	0.680	0.435	1.40E-18	3.46E-04	1.639	0.857	0.435	1.00E+00
COL4A2	9.43E-31	1.285	0.802	0.448	3.04E-26	1.24E-05	0.520	0.562	0.448	3.99E-01	1.06E-20	1.091	0.665	0.448	3.40E-16	1.23E-02	1.477	0.714	0.448	1.00E+00
COMMD4	1.76E-04	0.106	0.374	0.236	1.00E+00	5.29E-10	0.504	0.414	0.236	1.70E-05	3.44E-07	0.278	0.369	0.236	1.11E-02	2.35E-01	0.138	0.429	0.236	1.00E+00
COPE	1.17E-11	0.551	0.672	0.482	3.77E-07	3.90E-14	0.737	0.673	0.482	1.26E-09	2.93E-11	0.570	0.631	0.482	9.44E-07	1.09E-01	0.259	0.857	0.482	1.00E+00
CORO1B	6.27E-12	0.575	0.580	0.326	2.02E-07	7.70E-03	0.215	0.401	0.326	1.00E+00	7.08E-11	0.561	0.498	0.326	2.28E-06	1.30E-01	0.259	0.571	0.326	1.00E+00
COTL1	2.38E-33	1.535	0.710	0.344	7.67E-29	2.80E-31	1.519	0.654	0.344	9.02E-27	1.80E-29	1.225	0.626	0.344	5.79E-25	2.04E-02	0.837	0.714	0.344	1.00E+00
COX5A	8.77E-09	0.445	0.718	0.571	2.83E-04	3.91E-18	0.837	0.753	0.571	1.26E-13	4.52E-05	0.279	0.655	0.571	1.00E+00	1.43E-01	0.264	0.857	0.571	1.00E+00
CRIP2	5.23E-03	0.190	0.878	0.810	1.00E+00	1.21E-07	0.450	0.846	0.810	3.89E-03	1.57E-11	0.481	0.887	0.810	5.07E-07	1.29E-02	0.691	1.000	0.810	1.00E+00
CSTB	6.96E-28	1.107	0.878	0.636	2.24E-23	1.18E-23	1.105	0.864	0.636	3.78E-19	3.82E-17	0.628	0.818	0.636	1.23E-12	1.45E-01	0.458	0.857	0.636	1.00E+00
CTSA	2.10E-06	0.268	0.534	0.359	6.78E-02	1.81E-04	0.245	0.475	0.359	1.00E+00	9.01E-07	0.290	0.488	0.359	2.90E-02	6.25E-05	1.041	1.000	0.359	1.00E+00
CTSB	5.73E-09	0.401	0.649	0.430	1.85E-04	5.22E-08	0.546	0.556	0.430	1.68E-03	2.49E-16	0.705	0.621	0.430	8.01E-12	6.25E-02	0.359	0.714	0.430	1.00E+00
CTSS	7.33E-37	1.189	0.611	0.197	2.36E-32	1.15E-11	0.529	0.401	0.197	3.71E-07	2.16E-08	0.387	0.345	0.197	6.95E-04	8.29E-04	0.404	0.714	0.197	1.00E+00
CYBA	1.59E-16	0.768	0.618	0.290	5.13E-12	8.11E-05	0.409	0.414	0.290	1.00E+00	7.02E-11	0.554	0.488	0.290	2.26E-06	1.08E-02	0.865	0.714	0.290	1.00E+00
CYBC1	2.94E-11	0.288	0.458	0.212	9.47E-07	4.28E-05	0.258	0.340	0.212	1.00E+00	8.96E-11	0.383	0.384	0.212	2.89E-06	8.99E-03	0.649	0.571	0.212	1.00E+00
DBI	2.90E-10	0.620	0.763	0.609	9.35E-06	4.60E-15	0.731	0.784	0.609	1.48E-10	5.77E-04	0.200	0.665	0.609	1.00E+00	1.94E-02	0.470	0.857	0.609	1.00E+00
DBN1	2.22E-16	0.313	0.412	0.146	7.15E-12	5.38E-10	0.370	0.315	0.146	1.73E-05	5.68E-10	0.288	0.296	0.146	1.83E-05	2.95E-01	0.117	0.286	0.146	1.00E+00
DDRGK1	1.62E-05	0.215	0.473	0.309	5.20E-01	9.68E-04	0.287	0.407	0.309	1.00E+00	2.36E-09	0.405	0.468	0.309	7.62E-05	7.55E-02	0.452	0.571	0.309	1.00E+00
DECR1	6.09E-07	0.370	0.519	0.350	1.96E-02	1.79E-03	0.210	0.432	0.350	1.00E+00	6.56E-09	0.427	0.507	0.350	2.11E-04	4.10E-01	0.179	0.429	0.350	1.00E+00
DMAC1	1.19E-08	0.367	0.489	0.269	3.84E-04	3.85E-04	0.253	0.377	0.269	1.00E+00	1.20E-09	0.338	0.438	0.269	3.87E-05	1.64E-02	0.210	0.714	0.269	1.00E+00
DNAJC15	4.53E-10	0.408	0.603	0.388	1.46E-05	5.50E-02	0.195	0.444	0.388	1.00E+00	4.84E-10	0.471	0.557	0.388	1.56E-05	1.11E-01	0.265	0.714	0.388	1.00E+00
DNMT1	9.11E-14	0.633	0.405	0.159	2.94E-09	8.48E-12	0.416	0.352	0.159	2.73E-07	1.83E-02	0.166	0.212	0.159	1.00E+00	3.03E-01	0.187	0.286	0.159	1.00E+00
DYSF	5.61E-14	0.523	0.427	0.176	1.81E-09	2.26E-07	0.428	0.321	0.176	7.27E-03	6.01E-10	0.393	0.330	0.176	1.93E-05	3.08E-03	0.513	0.571	0.176	1.00E+00
EDF1	6.67E-06	0.411	0.885	0.777	2.15E-01	7.91E-12	0.514	0.846	0.777	2.55E-07	2.11E-11	0.463	0.852	0.777	6.80E-07	6.79E-02	0.857	1.000	0.777	1.00E+00
EGFL7	5.96E-07	0.416	0.824	0.672	1.92E-02	7.51E-08	0.336	0.802	0.672	2.42E-03	4.55E-25	0.813	0.901	0.672	1.47E-20	1.04E-01	0.198	1.000	0.672	1.00E+00
ELOB	1.28E-04	0.454	0.802	0.704	1.00E+00	1.18E-04	0.447	0.778	0.704	1.00E+00	1.06E-27	0.786	0.852	0.704	3.43E-23	4.96E-02	0.565	0.857	0.704	1.00E+00
ENG	6.58E-31	1.374	0.863	0.660	2.12E-26	1.61E-13	0.986	0.759	0.660	5.19E-09	2.06E-60	1.600	0.901	0.660	6.63E-56	2.21E-04	2.254	1.000	0.660	1.00E+00
EPSTI1	1.95E-55	0.707	0.458	0.068	6.27E-51	4.54E-04	0.143	0.142	0.068	1.00E+00	1.49E-14	0.242	0.217	0.068	4.79E-10	1.83E-02	0.581	0.286	0.068	1.00E+00
FAAP20	3.02E-03	0.206	0.504	0.375	1.00E+00	1.17E-03	0.230	0.475	0.375	1.00E+00	7.60E-07	0.352	0.507	0.375	2.45E-02	2.91E-01	0.177	0.571	0.375	1.00E+00
FAM167B	1.23E-34	0.995	0.504	0.139	3.95E-30	4.17E-27	0.935	0.420	0.139	1.34E-22	9.11E-37	0.874	0.448	0.139	2.93E-32	2.29E-04	1.438	0.571	0.139	1.00E+00
FAM174C	2.54E-06	0.182	0.458	0.274	8.19E-02	2.25E-06	0.415	0.414	0.274	7.23E-02	2.84E-14	0.554	0.468	0.274	9.14E-10	3.35E-01	0.149	0.429	0.274	1.00E+00
FAM207A	9.94E-16	0.300	0.336	0.107	3.20E-11	1.20E-04	0.137	0.204	0.107	1.00E+00	2.38E-10	0.289	0.246	0.107	7.67E-06	1.19E-01	0.239	0.286	0.107	1.00E+00
FAM50A	5.98E-05	0.198	0.473	0.314	1.00E+00	2.36E-02	0.249	0.383	0.314	1.00E+00	1.01E-03	0.178	0.394	0.314	1.00E+00	1.09E-02	0.589	0.714	0.314	1.00E+00
FBXW5	3.47E-07	0.327	0.519	0.314	1.12E-02	6.39E-07	0.407	0.463	0.314	2.06E-02	6.10E-07	0.385	0.438	0.314	1.97E-02	4.24E-02	0.127	0.714	0.314	1.00E+00
FDPS	6.12E-14	0.507	0.710	0.446	1.97E-09	2.50E-06	0.339	0.562	0.446	8.04E-02	8.59E-13	0.581	0.616	0.446	2.77E-08	3.70E-03	0.499	1.000	0.446	1.00E+00
FKBP10	2.26E-05	0.303	0.374	0.229	7.28E-01	8.16E-08	0.355	0.389	0.229	2.63E-03	3.72E-10	0.480	0.384	0.229	1.20E-05	1.70E-01	0.287	0.429	0.229	1.00E+00
FKBP1A	3.25E-23	1.214	0.878	0.818	1.05E-18	6.07E-15	0.720	0.827	0.818	1.96E-10	1.01E-70	1.802	0.946	0.818	3.24E-66	7.11E-04	1.193	1.000	0.818	1.00E+00
G6PC3	1.16E-07	0.148	0.305	0.134	3.74E-03	1.19E-05	0.177	0.253	0.134	3.83E-01	8.23E-08	0.276	0.261	0.134	2.65E-03	2.26E-02	0.217	0.429	0.134	1.00E+00
GADD45GIP1	4.04E-13	0.758	0.733	0.493	1.30E-08	3.07E-18	0.997	0.685	0.493	9.89E-14	5.49E-17	0.667	0.690	0.493	1.77E-12	1.24E-01	0.121	0.857	0.493	1.00E+00
GAPDH	4.01E-03	0.250	0.954	0.915	1.00E+00	5.38E-12	0.542	0.957	0.915	1.73E-07	2.93E-16	0.442	0.961	0.915	9.45E-12	2.63E-01	0.475	1.000	0.915	1.00E+00
GET3	5.13E-10	0.321	0.534	0.293	1.65E-05	1.04E-07	0.467	0.451	0.293	3.36E-03	2.86E-06	0.268	0.424	0.293	9.20E-02	6.63E-02	0.554	0.571	0.293	1.00E+00
GPR137	2.19E-11	0.275	0.351	0.143	7.04E-07	8.65E-03	0.220	0.210	0.143	1.00E+00	6.42E-06	0.247	0.246	0.143	2.07E-01	2.69E-02	0.243	0.429	0.143	1.00E+00
GRAP	3.91E-04	0.261	0.137	0.060	1.00E+00	4.57E-07	0.197	0.160	0.060	1.47E-02	3.41E-04	0.163	0.123	0.060	1.00E+00	4.79E-05	0.687	0.429	0.060	1.00E+00
GRN	8.38E-12	0.623	0.702	0.522	2.70E-07	4.74E-08	0.450	0.642	0.522	1.53E-03	3.47E-22	0.839	0.719	0.522	1.12E-17	9.69E-02	0.173	0.857	0.522	1.00E+00
GUK1	3.04E-02	0.186	0.817	0.778	1.00E+00	2.68E-28	0.895	0.901	0.778	8.63E-24	9.43E-25	0.724	0.862	0.778	3.04E-20	3.66E-02	0.471	1.000	0.778	1.00E+00
H2AFJ	6.13E-03	0.299	0.519	0.404	1.00E+00	2.69E-05	0.566	0.543	0.404	8.65E-01	9.18E-14	0.617	0.601	0.404	2.96E-09	2.65E-03	0.841	0.857	0.404	1.00E+00
H2AFV	5.77E-03	0.179	0.588	0.440	1.00E+00	2.09E-02	0.527	0.475	0.440	1.00E+00	4.46E-02	0.187	0.478	0.440	1.00E+00	1.01E-01	0.415	0.714	0.440	1.00E+00
HAGLROS	3.75E-10	0.196	0.260	0.094	1.21E-05	4.98E-04	0.159	0.173	0.094	1.00E+00	2.50E-02	0.155	0.138	0.094	1.00E+00	8.26E-02	0.146	0.286	0.094	1.00E+00
HCLS1	1.10E-09	0.139	0.229	0.075	3.55E-05	3.23E-03	0.162	0.136	0.075	1.00E+00	5.76E-06	0.171	0.163	0.075	1.85E-01	2.79E-04	0.859	0.429	0.075	1.00E+00
HLA-C	9.58E-19	0.797	0.962	0.913	3.09E-14	2.58E-06	0.293	0.926	0.913	8.30E-02	1.87E-23	0.616	0.946	0.913	6.02E-19	2.98E-02	0.505	1.000	0.913	1.00E+00
HM13	2.47E-03	0.158	0.458	0.344	1.00E+00	1.65E-02	0.181	0.420	0.344	1.00E+00	4.75E-04	0.304	0.433	0.344	1.00E+00	2.01E-01	0.152	0.571	0.344	1.00E+00

HMOX2	4.51E-12	0.522	0.519	0.288	1.45E-07	3.51E-06	0.334	0.432	0.288	1.13E-01	4.33E-03	0.207	0.350	0.288	1.00E+00	1.45E-01	0.105	0.571	0.288	1.00E+00
HPRT1	6.90E-05	0.215	0.374	0.225	1.00E+00	1.20E-06	0.253	0.370	0.225	3.85E-02	1.82E-05	0.204	0.335	0.225	5.86E-01	1.62E-01	0.156	0.429	0.225	1.00E+00
HSPG2	2.54E-17	0.968	0.832	0.554	8.20E-13	3.13E-02	0.117	0.599	0.554	1.00E+00	4.06E-46	1.389	0.847	0.554	1.31E-41	1.64E-03	1.524	0.857	0.554	1.00E+00
HYAL2	3.83E-19	0.992	0.710	0.414	1.23E-14	6.58E-26	1.244	0.704	0.414	2.12E-21	1.71E-33	1.086	0.734	0.414	5.50E-29	1.33E-03	1.166	0.857	0.414	1.00E+00
ICAM2	5.75E-17	0.876	0.710	0.438	1.85E-12	5.24E-06	0.425	0.568	0.438	1.69E-01	1.02E-04	0.166	0.547	0.438	1.00E+00	2.74E-03	1.147	0.857	0.438	1.00E+00
IFI30	3.07E-27	0.744	0.473	0.140	9.89E-23	1.37E-05	0.208	0.259	0.140	4.41E-01	1.39E-14	0.479	0.330	0.140	4.47E-10	1.30E-03	0.331	0.571	0.140	1.00E+00
IFITM2	2.12E-03	0.158	0.870	0.863	1.00E+00	1.54E-08	0.396	0.901	0.863	4.95E-04	1.59E-04	0.176	0.901	0.863	1.00E+00	9.03E-05	1.356	1.000	0.863	1.00E+00
IFITM3	1.35E-29	0.937	0.985	0.942	4.35E-25	1.42E-14	0.480	0.975	0.942	4.57E-10	1.59E-43	0.846	0.985	0.942	5.13E-39	4.29E-04	1.013	1.000	0.942	1.00E+00
IGFBP7	4.47E-39	1.986	0.977	0.913	1.44E-34	1.34E-35	1.274	0.963	0.913	4.32E-31	8.81E-32	0.868	0.961	0.913	2.84E-27	1.07E-03	1.786	1.000	0.913	1.00E+00
IL18BP	1.08E-13	0.201	0.321	0.107	3.48E-09	1.26E-01	0.103	0.142	0.107	1.00E+00	1.40E-07	0.250	0.222	0.107	4.52E-03	3.07E-05	0.898	0.571	0.107	9.90E-01
IL32	9.43E-49	1.952	0.916	0.467	3.04E-44	9.93E-08	0.913	0.599	0.467	3.20E-03	9.76E-01	0.419	0.429	0.467	1.00E+00	2.11E-03	1.633	0.857	0.467	1.00E+00
IL3RA	7.02E-07	0.518	0.626	0.493	2.26E-02	7.43E-02	0.177	0.519	0.493	1.00E+00	1.31E-03	0.203	0.591	0.493	1.00E+00	9.68E-01	0.211	0.429	0.493	1.00E+00
INSR	4.37E-03	0.190	0.252	0.158	1.00E+00	9.53E-05	0.565	0.259	0.158	1.00E+00	8.04E-06	0.785	0.261	0.158	2.59E-01	1.15E-03	1.145	0.571	0.158	1.00E+00
ISG15	5.26E-33	1.635	0.832	0.443	1.69E-28	2.33E-08	0.608	0.599	0.443	7.52E-04	6.01E-10	0.731	0.616	0.443	1.94E-05	2.77E-02	0.198	0.857	0.443	1.00E+00
KRTCAP2	1.13E-02	0.175	0.786	0.686	1.00E+00	1.20E-03	0.212	0.741	0.686	1.00E+00	1.43E-09	0.411	0.793	0.686	4.59E-05	9.88E-02	0.700	1.000	0.686	1.00E+00
LAMB1	2.83E-14	0.393	0.481	0.212	9.12E-10	3.34E-07	0.229	0.370	0.212	1.08E-02	4.66E-05	0.268	0.320	0.212	1.00E+00	4.35E-04	0.726	0.714	0.212	1.00E+00
LAMTOR2	4.43E-15	0.594	0.649	0.387	1.43E-10	3.26E-11	0.511	0.574	0.387	1.05E-06	3.38E-18	0.731	0.611	0.387	1.09E-13	6.97E-02	0.284	0.714	0.387	1.00E+00
LAYN	1.47E-15	0.537	0.374	0.134	4.74E-11	9.98E-25	0.757	0.414	0.134	3.21E-20	9.10E-02	0.105	0.172	0.134	1.00E+00	3.19E-06	0.856	0.714	0.134	1.03E-01
LGALS9	6.46E-48	1.144	0.672	0.193	2.08E-43	1.96E-03	0.209	0.284	0.193	1.00E+00	5.74E-18	0.699	0.419	0.193	1.85E-13	1.38E-04	1.797	0.714	0.193	1.00E+00
LGMN	2.33E-05	0.212	0.435	0.273	7.52E-01	5.25E-04	0.290	0.377	0.273	1.00E+00	1.23E-01	0.120	0.305	0.273	1.00E+00	2.81E-06	0.967	1.000	0.273	9.05E-02
LMAN2	1.05E-07	0.365	0.603	0.424	3.39E-03	1.70E-03	0.263	0.512	0.424	1.00E+00	1.73E-06	0.381	0.527	0.424	5.57E-02	9.32E-02	0.698	0.714	0.424	1.00E+00
LSM7	8.20E-04	0.262	0.626	0.490	1.00E+00	8.00E-03	0.165	0.568	0.490	1.00E+00	3.10E-05	0.258	0.586	0.490	9.97E-01	1.03E-01	0.362	0.714	0.490	1.00E+00
MAP1LC3A	8.74E-03	0.157	0.496	0.375	1.00E+00	3.59E-02	0.200	0.432	0.375	1.00E+00	5.26E-04	0.290	0.468	0.375	1.00E+00	1.74E-02	0.633	0.714	0.375	1.00E+00
MAPK12	2.46E-03	0.184	0.183	0.100	1.00E+00	1.91E-02	0.171	0.154	0.100	1.00E+00	1.92E-04	0.148	0.177	0.100	1.00E+00	1.23E-04	0.119	0.571	0.100	1.00E+00
MGAT4A	3.68E-06	0.226	0.359	0.197	1.18E-01	5.93E-01	0.174	0.173	0.197	1.00E+00	4.80E-02	0.136	0.241	0.197	1.00E+00	5.34E-03	0.550	0.571	0.197	1.00E+00
MGP	1.41E-18	1.686	0.794	0.644	4.55E-14	2.68E-04	0.696	0.698	0.644	1.00E+00	1.70E-08	0.858	0.695	0.644	5.46E-04	1.28E-02	0.386	1.000	0.644	1.00E+00
MICOS10	5.37E-10	0.506	0.756	0.563	1.73E-05	6.50E-16	0.772	0.741	0.563	2.09E-11	3.85E-05	0.243	0.650	0.563	1.00E+00	2.79E-02	0.389	0.857	0.563	1.00E+00
MIEN1	1.18E-02	0.227	0.389	0.299	1.00E+00	9.23E-08	0.393	0.457	0.299	2.97E-03	1.40E-04	0.297	0.394	0.299	1.00E+00	5.90E-03	0.739	0.714	0.299	1.00E+00
MIF	1.64E-21	0.939	0.916	0.801	5.27E-17	1.35E-23	0.938	0.920	0.801	4.33E-19	1.02E-28	0.817	0.931	0.801	3.30E-24	1.13E-01	0.468	1.000	0.801	1.00E+00
MLEC	4.01E-17	0.608	0.450	0.177	1.29E-12	1.50E-18	0.802	0.420	0.177	4.84E-14	7.50E-14	0.575	0.360	0.177	2.42E-09	1.92E-05	1.192	0.714	0.177	6.20E-01
MPG	5.05E-03	0.173	0.473	0.361	1.00E+00	2.43E-08	0.648	0.506	0.361	7.82E-04	5.16E-05	0.369	0.453	0.361	1.00E+00	4.00E-02	0.812	0.714	0.361	1.00E+00
MRPL17	2.76E-20	0.733	0.603	0.268	8.87E-16	3.32E-15	0.789	0.506	0.268	1.07E-10	9.83E-09	0.354	0.429	0.268	3.17E-04	2.11E-02	0.716	0.571	0.268	1.00E+00
MRPL20	1.19E-05	0.361	0.626	0.467	3.83E-01	9.16E-13	0.607	0.648	0.467	2.95E-08	4.04E-07	0.422	0.596	0.467	1.30E-02	1.28E-02	0.748	0.857	0.467	1.00E+00
MRPL34	2.10E-06	0.354	0.481	0.289	6.75E-02	7.72E-11	0.494	0.481	0.289	2.49E-06	2.84E-04	0.309	0.374	0.289	1.00E+00	2.84E-05	1.062	1.000	0.289	9.14E-01
MRPL52	3.79E-10	0.487	0.656	0.407	1.22E-05	4.47E-03	0.275	0.481	0.407	1.00E+00	3.51E-06	0.336	0.532	0.407	1.13E-01	6.67E-03	0.796	0.857	0.407	1.00E+00
MRPS15	9.78E-11	0.416	0.595	0.339	3.15E-06	1.90E-05	0.392	0.475	0.339	6.12E-01	4.42E-04	0.338	0.414	0.339	1.00E+00	1.15E-01	0.101	0.714	0.339	1.00E+00
MRPS16	1.04E-09	0.273	0.489	0.257	3.33E-05	5.91E-09	0.591	0.426	0.257	1.90E-04	2.46E-03	0.209	0.335	0.257	1.00E+00	2.62E-01	0.148	0.429	0.257	1.00E+00
MRPS21	7.27E-06	0.447	0.702	0.507	2.34E-01	1.35E-06	0.466	0.623	0.507	4.33E-02	5.13E-15	0.644	0.695	0.507	1.65E-10	3.54E-02	0.685	0.857	0.507	1.00E+00
MSRB2	7.60E-19	0.352	0.366	0.113	2.45E-14	3.67E-10	0.186	0.272	0.113	1.18E-05	1.32E-25	0.505	0.355	0.113	4.24E-21	2.60E-04	0.219	0.571	0.113	1.00E+00
MTCH2	1.53E-07	0.203	0.389	0.200	4.94E-03	1.40E-07	0.295	0.358	0.200	4.52E-03	6.29E-09	0.383	0.350	0.200	2.03E-04	2.26E-02	0.160	0.571	0.200	1.00E+00
MTLN	7.73E-11	0.431	0.580	0.320	2.49E-06	1.39E-03	0.218	0.420	0.320	1.00E+00	1.43E-05	0.346	0.433	0.320	4.61E-01	1.57E-01	0.372	0.571	0.320	1.00E+00
MYDGF	2.13E-14	0.604	0.718	0.483	6.86E-10	7.75E-07	0.348	0.623	0.483	2.50E-02	1.14E-01	0.101	0.493	0.483	1.00E+00	6.71E-01	0.105	0.571	0.483	1.00E+00
MYL6	5.75E-22	0.834	0.977	0.916	1.85E-17	1.39E-16	0.517	0.914	0.916	4.46E-12	1.92E-12	0.422	0.956	0.916	6.19E-08	1.41E-01	0.316	1.000	0.916	1.00E+00
NAA38	2.16E-09	0.586	0.687	0.508	6.96E-05	5.47E-06	0.389	0.617	0.508	1.76E-01	1.30E-25	1.050	0.719	0.508	4.19E-21	2.39E-03	0.606	1.000	0.508	1.00E+00
NDUFA11	1.23E-03	0.204	0.718	0.603	1.00E+00	1.10E-05	0.514	0.667	0.603	3.55E-01	1.67E-13	0.592	0.764	0.603	5.39E-09	5.65E-03	0.639	1.000	0.603	1.00E+00
NDUFA13	1.00E-10	0.559	0.809	0.630	3.22E-06	4.64E-11	0.520	0.772	0.630	1.50E-06	1.37E-13	0.554	0.768	0.630	4.42E-09	1.23E-02	0.698	1.000	0.630	1.00E+00
NDUFA6	7.21E-09	0.434	0.656	0.447	2.32E-04	2.06E-04	0.524	0.543	0.447	1.00E+00	1.97E-02	0.228	0.488	0.447	1.00E+00	1.54E-01	0.128	0.714	0.447	1.00E+00
NDUFA8	2.67E-13	0.581	0.618	0.370	8.59E-09	1.15E-11	0.470	0.574	0.370	3.69E-07	9.66E-08	0.443	0.488	0.370	3.11E-03	7.18E-02	0.327	0.714	0.370	1.00E+00
NDUFB11	2.99E-04	0.341	0.672	0.565	1.00E+00	2.07E-04	0.256	0.648	0.565	1.00E+00	3.89E-15	0.631	0.719	0.565	1.25E-10	2.45E-01	0.725	0.714	0.565	1.00E+00
NDUFB2	4.08E-14	0.656	0.824	0.587	1.31E-09	2.48E-06	0.384	0.679	0.587	7.99E-02	8.07E-20	0.758	0.759	0.587	2.60E-15	2.73E-01	0.348	0.857	0.587	1.00E+00
NDUFS5	3.30E-02	0.152	0.779	0.678	1.00E+00	4.15E-09	0.559	0.784	0.678	1.34E-04	3.36E-14	0.610	0.793	0.678	1.08E-09	3.13E-02	0.761	1.000	0.678	1.00E+00

NDUFS6	3.74E-10	0.579	0.687	0.483	1.20E-05	3.50E-09	0.599	0.623	0.483	1.13E-04	9.31E-21	0.750	0.695	0.483	3.00E-16	1.17E-01	0.119	0.857	0.483	1.00E+00
NDUFS7	3.90E-09	0.416	0.618	0.403	1.26E-04	1.17E-12	0.693	0.617	0.403	3.77E-08	6.16E-05	0.283	0.507	0.403	1.00E+00	1.59E-01	0.674	0.714	0.403	1.00E+00
NDUFS8	6.60E-08	0.634	0.580	0.393	2.13E-03	5.30E-10	0.575	0.574	0.393	1.71E-05	2.62E-07	0.385	0.507	0.393	8.43E-03	5.11E-03	0.538	0.857	0.393	1.00E+00
NECAP2	9.33E-09	0.290	0.534	0.318	3.01E-04	1.56E-02	0.278	0.377	0.318	1.00E+00	8.54E-02	0.175	0.345	0.318	1.00E+00	1.40E-03	0.635	0.857	0.318	1.00E+00
NEDD8	6.62E-12	0.598	0.832	0.693	2.13E-07	9.70E-07	0.420	0.759	0.693	3.12E-02	1.73E-09	0.365	0.759	0.693	5.58E-05	1.80E-01	0.340	0.857	0.693	1.00E+00
NME4	7.03E-06	0.344	0.427	0.258	2.26E-01	3.63E-08	0.368	0.414	0.258	1.17E-03	5.54E-07	0.380	0.379	0.258	1.78E-02	7.18E-02	0.346	0.571	0.258	1.00E+00
NUDT14	4.48E-05	0.237	0.519	0.348	1.00E+00	5.49E-05	0.221	0.475	0.348	1.00E+00	1.53E-04	0.206	0.453	0.348	1.00E+00	2.45E-05	1.027	1.000	0.348	7.89E-01
NUDT16	2.73E-03	0.229	0.351	0.241	1.00E+00	6.29E-01	0.161	0.247	0.241	1.00E+00	6.54E-02	0.124	0.281	0.241	1.00E+00	4.02E-04	0.362	0.857	0.241	1.00E+00
NUDT5	6.66E-25	0.580	0.573	0.212	2.15E-20	1.18E-15	0.535	0.451	0.212	3.80E-11	1.33E-14	0.473	0.424	0.212	4.28E-10	2.21E-03	0.350	0.714	0.212	1.00E+00
OAS2	1.68E-11	0.328	0.328	0.128	5.43E-07	6.19E-02	0.122	0.173	0.128	1.00E+00	3.79E-05	0.200	0.222	0.128	1.00E+00	1.60E-02	0.376	0.429	0.128	1.00E+00
OPTN	1.42E-11	0.587	0.641	0.416	4.56E-07	3.62E-02	0.143	0.481	0.416	1.00E+00	2.22E-04	0.321	0.498	0.416	1.00E+00	2.35E-03	0.823	0.857	0.416	1.00E+00
OST4	1.92E-18	0.720	0.847	0.688	6.17E-14	2.01E-08	0.492	0.790	0.688	6.49E-04	8.39E-20	0.661	0.803	0.688	2.70E-15	2.22E-02	0.373	1.000	0.688	1.00E+00
P4HB	1.01E-13	0.711	0.710	0.507	3.24E-09	4.21E-10	0.693	0.667	0.507	1.36E-05	2.62E-13	0.587	0.650	0.507	8.44E-09	1.61E-04	0.967	1.000	0.507	1.00E+00
PARVB	1.50E-09	0.392	0.542	0.322	4.84E-05	5.42E-05	0.341	0.438	0.322	1.00E+00	7.74E-11	0.501	0.502	0.322	2.49E-06	6.63E-03	0.681	0.714	0.322	1.00E+00
PAXX	3.18E-05	0.101	0.435	0.257	1.00E+00	2.98E-05	0.243	0.395	0.257	9.61E-01	2.32E-14	0.613	0.463	0.257	7.47E-10	1.17E-01	0.294	0.571	0.257	1.00E+00
PDIA3	9.77E-11	0.646	0.733	0.624	3.15E-06	2.95E-02	0.155	0.654	0.624	1.00E+00	3.28E-05	0.298	0.685	0.624	1.00E+00	2.71E-02	0.638	0.857	0.624	1.00E+00
PDLIM2	6.08E-05	0.187	0.504	0.338	1.00E+00	2.38E-01	0.138	0.364	0.338	1.00E+00	6.95E-02	0.181	0.374	0.338	1.00E+00	1.26E-02	0.614	0.714	0.338	1.00E+00
PDLIM7	5.27E-13	0.391	0.466	0.217	1.70E-08	4.49E-15	0.503	0.451	0.217	1.45E-10	1.20E-15	0.542	0.433	0.217	3.86E-11	1.27E-01	0.434	0.429	0.217	1.00E+00
PFN1	9.76E-29	1.010	0.931	0.815	3.14E-24	1.94E-06	0.466	0.809	0.815	6.26E-02	6.65E-31	0.828	0.931	0.815	2.14E-26	2.48E-02	0.910	1.000	0.815	1.00E+00
PGAM1	8.02E-10	0.589	0.656	0.458	2.58E-05	1.31E-03	0.369	0.537	0.458	1.00E+00	8.64E-03	0.138	0.512	0.458	1.00E+00	1.95E-03	0.925	0.857	0.458	1.00E+00
PKM	3.91E-03	0.304	0.702	0.642	1.00E+00	5.97E-04	0.365	0.704	0.642	1.00E+00	5.20E-05	0.341	0.719	0.642	1.00E+00	1.50E-01	0.599	0.857	0.642	1.00E+00
PLOD1	4.55E-14	0.392	0.397	0.160	1.46E-09	1.60E-06	0.401	0.290	0.160	5.16E-02	6.00E-11	0.364	0.320	0.160	1.93E-06	1.08E-03	0.896	0.571	0.160	1.00E+00
PLVAP	3.91E-02	0.112	0.763	0.708	1.00E+00	2.21E-05	0.540	0.716	0.708	7.12E-01	1.13E-06	0.319	0.823	0.708	3.64E-02	1.07E-03	1.836	1.000	0.708	1.00E+00
POGLUT1	1.19E-12	0.273	0.336	0.123	3.82E-08	1.63E-03	0.119	0.204	0.123	1.00E+00	5.84E-02	0.134	0.163	0.123	1.00E+00	1.32E-02	0.216	0.429	0.123	1.00E+00
POLD4	2.23E-05	0.328	0.511	0.358	7.18E-01	8.60E-08	0.397	0.525	0.358	2.77E-03	3.65E-05	0.261	0.458	0.358	1.00E+00	7.55E-03	0.672	0.857	0.358	1.00E+00
PON2	3.15E-09	0.477	0.519	0.319	1.02E-04	1.69E-07	0.421	0.475	0.319	5.43E-03	1.96E-28	0.962	0.611	0.319	6.30E-24	4.19E-02	0.784	0.571	0.319	1.00E+00
PPIB	1.10E-18	0.903	0.809	0.670	3.55E-14	8.87E-14	0.724	0.778	0.670	2.86E-09	9.12E-18	0.726	0.803	0.670	2.94E-13	1.52E-04	1.180	1.000	0.670	1.00E+00
PPP4C	1.75E-06	0.329	0.588	0.416	5.63E-02	1.82E-03	0.251	0.525	0.416	1.00E+00	4.01E-14	0.513	0.606	0.416	1.29E-09	1.10E-01	0.291	0.714	0.416	1.00E+00
PRADC1	8.47E-09	0.138	0.267	0.102	2.73E-04	2.00E-14	0.356	0.290	0.102	6.43E-10	4.34E-13	0.290	0.261	0.102	1.40E-08	6.62E-03	0.145	0.429	0.102	1.00E+00
PRKAR2A	1.02E-01	0.104	0.275	0.215	1.00E+00	2.47E-01	0.144	0.247	0.215	1.00E+00	9.42E-04	0.218	0.296	0.215	1.00E+00	1.85E-02	0.564	0.571	0.215	1.00E+00
PSMB10	1.09E-23	0.865	0.656	0.304	3.52E-19	2.98E-04	0.376	0.407	0.304	1.00E+00	1.66E-21	0.782	0.557	0.304	5.34E-17	1.29E-02	0.421	0.714	0.304	1.00E+00
PSMB6	4.98E-11	0.502	0.748	0.542	1.60E-06	1.41E-07	0.458	0.654	0.542	4.56E-03	2.25E-03	0.215	0.606	0.542	1.00E+00	9.52E-02	0.264	0.857	0.542	1.00E+00
PSMD13	1.18E-06	0.223	0.527	0.341	3.79E-02	1.76E-10	0.503	0.543	0.341	5.67E-06	5.76E-04	0.356	0.429	0.341	1.00E+00	1.90E-02	0.568	0.714	0.341	1.00E+00
PUF60	3.58E-04	0.170	0.481	0.339	1.00E+00	4.86E-03	0.160	0.426	0.339	1.00E+00	1.51E-02	0.135	0.394	0.339	1.00E+00	4.12E-02	0.426	0.714	0.339	1.00E+00
PXDN	2.09E-13	0.513	0.481	0.233	6.72E-09	5.17E-10	0.509	0.414	0.233	1.67E-05	1.54E-14	0.562	0.429	0.233	4.97E-10	1.99E-02	0.320	0.571	0.233	1.00E+00
PYCARD	7.03E-13	0.420	0.489	0.245	2.26E-08	6.58E-09	0.515	0.426	0.245	2.12E-04	4.67E-20	0.688	0.493	0.245	1.50E-15	6.25E-02	0.833	0.571	0.245	1.00E+00
RAB13	2.80E-18	0.797	0.847	0.699	9.03E-14	5.90E-34	1.231	0.846	0.699	1.90E-29	5.09E-25	0.892	0.813	0.699	1.64E-20	3.47E-02	0.472	0.857	0.699	1.00E+00
RAB32	7.69E-19	0.366	0.397	0.131	2.48E-14	3.10E-09	0.382	0.284	0.131	1.00E-04	2.32E-07	0.225	0.251	0.131	7.48E-03	1.72E-02	0.383	0.429	0.131	1.00E+00
RABAC1	5.39E-06	0.345	0.771	0.667	1.74E-01	2.09E-03	0.297	0.685	0.667	1.00E+00	1.69E-09	0.438	0.759	0.667	5.44E-05	6.83E-02	0.419	0.857	0.667	1.00E+00
RAI14	2.57E-18	0.650	0.603	0.289	8.28E-14	6.85E-08	0.516	0.457	0.289	2.21E-03	8.05E-08	0.662	0.429	0.289	2.59E-03	9.27E-03	0.618	0.714	0.289	1.00E+00
RFXANK	7.53E-14	0.438	0.481	0.218	2.42E-09	4.72E-13	0.420	0.444	0.218	1.52E-08	3.77E-07	0.251	0.345	0.218	1.21E-02	1.38E-01	0.532	0.429	0.218	1.00E+00
RGCC	2.63E-07	0.499	0.527	0.306	8.46E-03	2.04E-03	0.499	0.407	0.306	1.00E+00	5.76E-03	0.301	0.389	0.306	1.00E+00	5.82E-03	1.344	0.714	0.306	1.00E+00
RGS3	3.63E-33	1.297	0.626	0.221	1.17E-28	1.26E-05	0.449	0.358	0.221	4.04E-01	1.38E-02	0.334	0.276	0.221	1.00E+00	1.34E-01	0.813	0.429	0.221	1.00E+00
RHOG	2.03E-12	0.478	0.565	0.316	6.53E-08	8.59E-03	0.213	0.395	0.316	1.00E+00	1.98E-04	0.318	0.404	0.316	1.00E+00	8.27E-06	0.937	1.000	0.316	2.66E-01
RNASEK	3.39E-15	0.672	0.855	0.735	1.09E-10	1.48E-07	0.430	0.784	0.735	4.75E-03	3.26E-17	0.607	0.837	0.735	1.05E-12	3.56E-04	1.015	1.000	0.735	1.00E+00
RNF145	1.07E-10	0.531	0.595	0.347	3.45E-06	5.08E-02	0.248	0.401	0.347	1.00E+00	2.48E-05	0.410	0.448	0.347	8.00E-01	4.80E-02	0.243	0.714	0.347	1.00E+00
RNF7	2.01E-12	0.540	0.718	0.473	6.46E-08	5.63E-11	0.623	0.617	0.473	1.81E-06	9.77E-08	0.381	0.591	0.473	3.15E-03	6.37E-02	0.514	0.714	0.473	1.00E+00
ROMO1	3.67E-04	0.388	0.611	0.476	1.00E+00	5.64E-02	0.240	0.556	0.476	1.00E+00	7.94E-18	0.664	0.690	0.476	2.56E-13	5.62E-02	0.354	0.857	0.476	1.00E+00
RPL28	2.77E-12	0.482	0.977	0.951	8.91E-08	4.55E-08	0.298	1.000	0.951	1.47E-03	1.76E-23	0.521	0.975	0.951	5.67E-19	1.91E-01	0.572	1.000	0.951	1.00E+00
RPS19BP1	3.31E-16	0.703	0.748	0.483	1.06E-11	7.12E-08	0.618	0.630	0.483	2.29E-03	4.69E-09	0.368	0.616	0.483	1.51E-04	9.36E-03	0.704	0.857	0.483	1.00E+00
S100A11	3.24E-32	1.234	0.931	0.812	1.04E-27	3.57E-22	0.944	0.938	0.812	1.15E-17	3.02E-22	0.791	0.892	0.812	9.72E-18	1.62E-01	0.258	1.000	0.812	1.00E+00

S100A13	3.78E-09	0.580	0.832	0.679	1.22E-04	1.65E-02	0.158	0.716	0.679	1.00E+00	1.04E-13	0.684	0.798	0.679	3.34E-09	5.98E-02	0.418	1.000	0.679	1.00E+00
SCAND1	3.04E-15	0.594	0.710	0.449	9.78E-11	1.72E-19	0.860	0.673	0.449	5.53E-15	6.20E-18	0.730	0.640	0.449	2.00E-13	1.13E-02	0.526	0.857	0.449	1.00E+00
SDF2L1	1.55E-07	0.277	0.473	0.265	4.99E-03	2.39E-07	0.253	0.432	0.265	7.71E-03	3.20E-09	0.405	0.433	0.265	1.03E-04	9.66E-02	0.368	0.571	0.265	1.00E+00
SEC61B	2.81E-10	0.531	0.832	0.681	9.05E-06	3.17E-16	0.753	0.796	0.681	1.02E-11	7.75E-06	0.407	0.739	0.681	2.50E-01	5.81E-03	0.552	1.000	0.681	1.00E+00
SELENOM	6.70E-13	1.010	0.733	0.586	2.16E-08	1.35E-13	0.771	0.728	0.586	4.35E-09	3.84E-10	0.561	0.704	0.586	1.24E-05	1.68E-03	1.813	1.000	0.586	1.00E+00
SERF2	1.28E-42	1.152	0.977	0.863	4.11E-38	5.56E-37	0.944	0.951	0.863	1.79E-32	1.11E-69	1.240	0.961	0.863	3.57E-65	1.33E-03	0.933	1.000	0.863	1.00E+00
SERPINB6	1.53E-05	0.324	0.580	0.459	4.94E-01	1.55E-10	0.520	0.630	0.459	5.00E-06	8.46E-23	0.720	0.695	0.459	2.72E-18	2.57E-02	0.476	0.857	0.459	1.00E+00
SERPINH1	6.23E-08	0.523	0.771	0.616	2.01E-03	8.50E-02	0.102	0.660	0.616	1.00E+00	8.06E-04	0.223	0.714	0.616	1.00E+00	4.52E-02	1.034	0.857	0.616	1.00E+00
SH3BGRL3	4.38E-09	0.503	0.901	0.758	1.41E-04	5.33E-22	0.953	0.877	0.758	1.72E-17	1.68E-22	0.786	0.882	0.758	5.41E-18	2.27E-02	0.709	1.000	0.758	1.00E+00
SHISA5	5.78E-07	0.354	0.595	0.401	1.86E-02	1.84E-05	0.330	0.537	0.401	5.94E-01	4.91E-08	0.391	0.552	0.401	1.58E-03	6.20E-04	0.970	0.857	0.401	1.00E+00
SHKBP1	2.50E-08	0.164	0.412	0.209	8.06E-04	4.06E-04	0.277	0.315	0.209	1.00E+00	5.00E-04	0.195	0.296	0.209	1.00E+00	1.56E-02	0.517	0.571	0.209	1.00E+00
SIRT6	1.63E-04	0.242	0.191	0.093	1.00E+00	4.55E-04	0.178	0.173	0.093	1.00E+00	6.21E-07	0.173	0.197	0.093	2.00E-02	2.93E-03	0.289	0.429	0.093	1.00E+00
SLC25A39	5.87E-14	0.569	0.496	0.235	1.89E-09	1.95E-03	0.225	0.327	0.235	1.00E+00	2.01E-08	0.266	0.384	0.235	6.46E-04	6.91E-02	0.412	0.571	0.235	1.00E+00
SLC35B1	7.04E-04	0.173	0.344	0.223	1.00E+00	2.38E-12	0.458	0.426	0.223	7.68E-08	4.49E-03	0.184	0.291	0.223	1.00E+00	2.39E-02	0.598	0.571	0.223	1.00E+00
SLC39A3	1.40E-04	0.154	0.214	0.107	1.00E+00	9.72E-07	0.197	0.228	0.107	3.13E-02	5.23E-06	0.195	0.207	0.107	1.68E-01	7.71E-03	0.275	0.429	0.107	1.00E+00
SLCO2A1	1.81E-10	0.327	0.397	0.178	5.83E-06	2.04E-10	0.567	0.358	0.178	6.59E-06	3.06E-28	0.935	0.463	0.178	9.87E-24	6.72E-03	1.047	0.571	0.178	1.00E+00
SMIM29	8.22E-04	0.165	0.282	0.169	1.00E+00	3.72E-09	0.343	0.340	0.169	1.20E-04	6.63E-12	0.350	0.345	0.169	2.13E-07	6.28E-03	0.208	0.571	0.169	1.00E+00
SNRPC	1.92E-07	0.244	0.534	0.336	6.18E-03	1.46E-13	0.583	0.556	0.336	4.69E-09	2.21E-03	0.226	0.414	0.336	1.00E+00	3.22E-02	0.333	0.714	0.336	1.00E+00
SOX18	4.48E-07	0.460	0.565	0.369	1.44E-02	2.98E-12	0.744	0.574	0.369	9.61E-08	8.23E-25	0.864	0.660	0.369	2.65E-20	9.09E-04	1.099	0.857	0.369	1.00E+00
SPARC	1.03E-09	0.686	0.870	0.770	3.31E-05	1.12E-05	0.764	0.796	0.770	3.59E-01	5.42E-32	1.212	0.916	0.770	1.75E-27	9.27E-04	2.085	1.000	0.770	1.00E+00
SPCS3	5.26E-06	0.235	0.458	0.288	1.70E-01	5.28E-05	0.262	0.414	0.288	1.00E+00	1.91E-07	0.443	0.424	0.288	6.15E-03	1.08E-02	0.244	0.714	0.288	1.00E+00
SSBP4	3.93E-03	0.174	0.351	0.245	1.00E+00	4.12E-04	0.227	0.346	0.245	1.00E+00	3.67E-05	0.286	0.340	0.245	1.00E+00	7.26E-02	0.123	0.571	0.245	1.00E+00
SSNA1	6.47E-08	0.371	0.557	0.353	2.08E-03	4.79E-07	0.400	0.494	0.353	1.54E-02	6.73E-09	0.466	0.502	0.353	2.17E-04	4.40E-02	0.261	0.714	0.353	1.00E+00
SSR4	7.09E-14	0.682	0.824	0.634	2.28E-09	4.60E-11	0.611	0.747	0.634	1.48E-06	5.47E-14	0.545	0.768	0.634	1.76E-09	2.37E-01	0.586	0.857	0.634	1.00E+00
STK25	5.54E-04	0.205	0.435	0.297	1.00E+00	1.38E-04	0.153	0.426	0.297	1.00E+00	3.70E-03	0.171	0.365	0.297	1.00E+00	2.07E-02	0.192	0.714	0.297	1.00E+00
STMP1	9.00E-10	0.345	0.450	0.229	2.90E-05	3.14E-08	0.329	0.401	0.229	1.01E-03	4.81E-19	0.541	0.473	0.229	1.55E-14	1.41E-03	0.411	0.714	0.229	1.00E+00
SYNPO	3.79E-08	0.447	0.458	0.249	1.22E-03	5.49E-05	0.456	0.370	0.249	1.00E+00	5.42E-07	0.397	0.379	0.249	1.74E-02	5.04E-04	1.168	0.714	0.249	1.00E+00
TAPBP	4.95E-19	0.823	0.763	0.515	1.59E-14	3.77E-03	0.165	0.599	0.515	1.00E+00	5.68E-11	0.483	0.650	0.515	1.83E-06	3.56E-03	1.008	0.857	0.515	1.00E+00
TAX1BP3	4.77E-04	0.209	0.489	0.366	1.00E+00	2.14E-16	0.822	0.617	0.366	6.88E-12	4.40E-16	0.748	0.576	0.366	1.42E-11	2.51E-03	0.526	0.857	0.366	1.00E+00
TCIM	5.16E-13	1.107	0.557	0.293	1.66E-08	7.81E-10	0.570	0.494	0.293	2.52E-05	1.29E-03	0.246	0.379	0.293	1.00E+00	9.95E-05	1.797	0.857	0.293	1.00E+00
TEX264	4.64E-02	0.176	0.466	0.388	1.00E+00	1.16E-04	0.331	0.500	0.388	1.00E+00	5.11E-02	0.101	0.424	0.388	1.00E+00	5.59E-02	0.297	0.714	0.388	1.00E+00
TGFB1	5.18E-09	0.452	0.466	0.264	1.67E-04	1.79E-04	0.295	0.383	0.264	1.00E+00	1.30E-09	0.470	0.419	0.264	4.19E-05	2.60E-05	0.822	0.857	0.264	8.39E-01
TIMM13	3.98E-14	0.659	0.695	0.427	1.28E-09	8.02E-08	0.475	0.580	0.427	2.58E-03	1.45E-18	0.664	0.650	0.427	4.67E-14	5.61E-02	0.610	0.714	0.427	1.00E+00
TIMP1	6.03E-13	2.082	0.824	0.680	1.94E-08	8.03E-22	1.548	0.821	0.680	2.59E-17	5.59E-19	0.936	0.842	0.680	1.80E-14	2.20E-02	1.322	0.857	0.680	1.00E+00
TKT	1.87E-07	0.495	0.534	0.370	6.03E-03	1.41E-03	0.333	0.463	0.370	1.00E+00	1.13E-04	0.410	0.443	0.370	1.00E+00	2.73E-02	0.329	0.714	0.370	1.00E+00
TMED9	2.39E-13	0.688	0.718	0.479	7.69E-09	1.42E-13	0.703	0.660	0.479	4.57E-09	3.40E-12	0.598	0.635	0.479	1.10E-07	3.29E-02	0.373	0.857	0.479	1.00E+00
TMEM160	2.80E-06	0.224	0.527	0.329	9.02E-02	5.84E-12	0.650	0.537	0.329	1.88E-07	1.60E-09	0.505	0.488	0.329	5.15E-05	1.80E-01	0.118	0.571	0.329	1.00E+00
TMEM204	1.91E-05	0.226	0.527	0.351	6.16E-01	1.19E-07	0.335	0.525	0.351	3.85E-03	1.73E-09	0.441	0.517	0.351	5.56E-05	5.40E-02	1.032	0.571	0.351	1.00E+00
TMEM255B	1.08E-16	0.680	0.634	0.364	3.49E-12	5.16E-03	0.173	0.444	0.364	1.00E+00	1.48E-11	0.473	0.542	0.364	4.75E-07	4.52E-02	0.493	0.714	0.364	1.00E+00
TMSB10	1.48E-30	0.918	0.992	0.972	4.76E-26	2.27E-02	0.166	0.994	0.972	1.00E+00	1.93E-51	0.884	0.990	0.972	6.22E-47	9.70E-02	0.341	1.000	0.972	1.00E+00
TNFRSF1B	2.35E-05	0.267	0.389	0.233	7.57E-01	5.63E-01	0.119	0.204	0.233	1.00E+00	2.86E-04	0.123	0.335	0.233	1.00E+00	2.11E-01	0.160	0.429	0.233	1.00E+00
TNFRSF6B	9.96E-60	2.737	0.740	0.222	3.21E-55	1.55E-39	2.474	0.605	0.222	5.00E-35	2.98E-56	1.731	0.650	0.222	9.58E-52	1.52E-01	0.328	0.429	0.222	1.00E+00
TPI1	5.66E-09	0.525	0.817	0.690	1.82E-04	3.23E-11	0.555	0.778	0.690	1.04E-06	1.24E-12	0.540	0.793	0.690	4.01E-08	4.41E-03	0.702	1.000	0.690	1.00E+00
TPM4	2.11E-07	0.517	0.779	0.699	6.80E-03	1.45E-01	0.166	0.728	0.699	1.00E+00	9.95E-03	0.262	0.739	0.699	1.00E+00	3.25E-02	0.444	1.000	0.699	1.00E+00
TRAPPC1	1.73E-09	0.415	0.603	0.388	5.57E-05	1.30E-10	0.473	0.580	0.388	4.19E-06	3.30E-27	0.798	0.675	0.388	1.06E-22	4.78E-02	0.256	0.714	0.388	1.00E+00
TRIOBP	7.37E-04	0.195	0.489	0.336	1.00E+00	5.46E-04	0.394	0.438	0.336	1.00E+00	1.74E-07	0.447	0.463	0.336	5.60E-03	2.09E-02	0.634	0.714	0.336	1.00E+00
TTYH3	2.37E-12	0.174	0.214	0.059	7.63E-08	5.89E-03	0.170	0.111	0.059	1.00E+00	2.29E-14	0.336	0.197	0.059	7.39E-10	1.18E-02	0.116	0.286	0.059	1.00E+00
TUBB6	4.60E-17	0.681	0.656	0.380	1.48E-12	4.77E-17	0.962	0.605	0.380	1.54E-12	5.90E-06	0.294	0.493	0.380	1.90E-01	1.14E-01	0.302	0.714	0.380	1.00E+00
TWF2	7.32E-06	0.161	0.458	0.286	2.36E-01	2.64E-03	0.157	0.377	0.286	1.00E+00	1.71E-06	0.282	0.409	0.286	5.51E-02	2.36E-02	0.186	0.714	0.286	1.00E+00
TXNDC17	6.11E-18	0.777	0.695	0.404	1.97E-13	1.51E-09	0.570	0.586	0.404	4.86E-05	8.78E-09	0.648	0.542	0.404	2.83E-04	1.36E-02	0.301	0.857	0.404	1.00E+00
TXNDC5	1.22E-03	0.201	0.321	0.201	1.00E+00	2.08E-01	0.111	0.235	0.201	1.00E+00	8.50E-07	0.392	0.320	0.201	2.74E-02	1.09E-01	0.299	0.429	0.201	1.00E+00

UBE2J1	1.91E-02	0.132	0.473	0.377	1.00E+00	3.14E-01	0.150	0.395	0.377	1.00E+00	6.57E-01	0.104	0.379	0.377	1.00E+00	1.30E-03	0.872	0.857	0.377	1.00E+00
UBE2L3	4.89E-12	0.629	0.656	0.454	1.57E-07	2.50E-15	0.842	0.660	0.454	8.06E-11	3.05E-12	0.559	0.616	0.454	9.82E-08	1.59E-02	0.526	0.857	0.454	1.00E+00
UBL5	3.91E-13	0.715	0.786	0.652	1.26E-08	1.21E-06	0.405	0.765	0.652	3.89E-02	1.69E-24	0.810	0.828	0.652	5.43E-20	1.54E-03	0.839	1.000	0.652	1.00E+00
UFD1	5.82E-05	0.201	0.511	0.352	1.00E+00	3.73E-09	0.557	0.525	0.352	1.20E-04	7.16E-10	0.598	0.507	0.352	2.30E-05	2.09E-01	0.143	0.571	0.352	1.00E+00
UQCR10	3.58E-07	0.444	0.603	0.423	1.15E-02	3.30E-07	0.451	0.568	0.423	1.06E-02	9.43E-15	0.628	0.611	0.423	3.04E-10	4.10E-02	0.486	0.714	0.423	1.00E+00
UQCR11	9.15E-12	0.682	0.794	0.641	2.95E-07	6.19E-06	0.365	0.716	0.641	1.99E-01	4.18E-35	1.115	0.818	0.641	1.35E-30	4.86E-02	0.513	0.857	0.641	1.00E+00
UROD	3.51E-02	0.204	0.351	0.274	1.00E+00	8.80E-02	0.121	0.327	0.274	1.00E+00	4.32E-03	0.195	0.345	0.274	1.00E+00	3.57E-03	0.646	0.714	0.274	1.00E+00
VKORC1	9.63E-11	0.488	0.710	0.483	3.10E-06	1.98E-12	0.638	0.660	0.483	6.39E-08	7.22E-08	0.438	0.616	0.483	2.33E-03	2.74E-03	0.859	0.857	0.483	1.00E+00
VPS25	1.65E-06	0.189	0.275	0.128	5.32E-02	3.00E-18	0.382	0.364	0.128	9.67E-14	1.37E-15	0.382	0.320	0.128	4.42E-11	5.66E-04	1.021	0.571	0.128	1.00E+00
VWA1	1.60E-41	1.538	0.725	0.297	5.16E-37	7.37E-15	0.916	0.512	0.297	2.37E-10	2.88E-66	1.836	0.734	0.297	9.29E-62	9.47E-06	1.985	0.857	0.297	3.05E-01
WDR83OS	1.23E-03	0.314	0.740	0.642	1.00E+00	6.17E-09	0.488	0.753	0.642	1.99E-04	6.19E-06	0.420	0.700	0.642	1.99E-01	8.50E-02	0.489	0.857	0.642	1.00E+00
XAF1	3.04E-15	0.679	0.542	0.260	9.81E-11	2.03E-01	0.231	0.290	0.260	1.00E+00	4.02E-02	0.104	0.310	0.260	1.00E+00	7.06E-02	0.261	0.571	0.260	1.00E+00
YIPF3	4.94E-06	0.239	0.557	0.390	1.59E-01	3.47E-04	0.298	0.481	0.390	1.00E+00	5.75E-04	0.204	0.478	0.390	1.00E+00	2.37E-04	0.852	1.000	0.390	1.00E+00
ZNF428	7.16E-08	0.250	0.565	0.357	2.30E-03	6.34E-08	0.380	0.506	0.357	2.04E-03	9.05E-10	0.508	0.512	0.357	2.92E-05	6.74E-02	0.172	0.714	0.357	1.00E+00
ZNF580	7.38E-04	0.254	0.374	0.255	1.00E+00	1.73E-01	0.143	0.290	0.255	1.00E+00	4.24E-03	0.304	0.320	0.255	1.00E+00	7.26E-03	0.585	0.714	0.255	1.00E+00
ZNHIT1	1.19E-09	0.431	0.756	0.558	3.84E-05	5.18E-06	0.412	0.667	0.558	1.67E-01	1.12E-15	0.669	0.704	0.558	3.60E-11	1.19E-01	0.552	0.714	0.558	1.00E+00

	MF18					MF21					MF24				
	p val	avg logFC	pct.1	pct.2	p val adj	p val	avg logFC	pct.1	pct.2	p val adj	p_val	avg logFC	pct.1	pct.2	p val adj
A2M	5.88E-03	0.655	0.793	0.808	1.00E+00	2.29E-04	0.354	0.819	0.808	1.00E+00	1.09E-03	0.789	0.839	0.808	1.00E+00
ABRACL	1.35E-06	0.555	0.324	0.175	4.34E-02	1.92E-36	1.146	0.509	0.175	6.18E-32	6.22E-02	0.160	0.290	0.175	1.00E+00
ACOT7	1.08E-03	0.194	0.117	0.049	1.00E+00	8.21E-27	0.528	0.246	0.049	2.65E-22	3.44E-03	0.224	0.161	0.049	1.00E+00
ACTB	9.82E-14	1.303	0.955	0.934	3.16E-09	1.85E-11	0.569	0.959	0.934	5.95E-07	1.61E-02	0.552	0.968	0.934	1.00E+00
ACVRL1	9.96E-06	0.597	0.468	0.349	3.21E-01	7.48E-17	0.722	0.567	0.349	2.41E-12	7.27E-03	0.523	0.516	0.349	1.00E+00
AL583785.1	5.53E-01	0.386	0.225	0.223	1.00E+00	2.75E-07	0.785	0.351	0.223	8.85E-03	4.10E-02	0.408	0.355	0.223	1.00E+00
ANAPC11	5.62E-02	0.270	0.523	0.516	1.00E+00	1.92E-11	0.659	0.643	0.516	6.18E-07	2.03E-01	0.344	0.516	0.516	1.00E+00
ANKRD65	1.13E-06	0.369	0.189	0.071	3.64E-02	3.06E-04	0.327	0.140	0.071	1.00E+00	1.43E-06	0.598	0.290	0.071	4.59E-02
ANTKMT	1.33E-02	0.255	0.207	0.136	1.00E+00	2.32E-04	0.359	0.222	0.136	1.00E+00	9.64E-03	0.340	0.290	0.136	1.00E+00
AP2S1	6.23E-11	1.009	0.622	0.495	2.00E-06	2.45E-26	1.362	0.690	0.495	7.89E-22	5.75E-03	0.560	0.613	0.495	1.00E+00
ARF3	1.65E-03	0.440	0.369	0.278	1.00E+00	1.79E-04	0.377	0.363	0.278	1.00E+00	8.05E-03	0.330	0.452	0.278	1.00E+00
ARHGDIB	7.75E-05	0.770	0.568	0.496	1.00E+00	1.18E-07	0.704	0.602	0.496	3.79E-03	2.43E-06	1.019	0.774	0.496	7.83E-02
ARPC1A	7.69E-01	0.184	0.387	0.461	1.00E+00	4.07E-01	0.198	0.427	0.461	1.00E+00	2.88E-01	0.150	0.516	0.461	1.00E+00
ARPC3	2.34E-02	0.334	0.640	0.684	1.00E+00	6.25E-22	1.112	0.760	0.684	2.01E-17	4.94E-02	0.258	0.710	0.684	1.00E+00
ATOX1	2.16E-08	0.759	0.667	0.587	6.95E-04	1.07E-13	0.703	0.702	0.587	3.44E-09	2.12E-05	0.909	0.742	0.587	6.84E-01
ATP5F1D	8.44E-03	0.368	0.649	0.637	1.00E+00	1.71E-14	0.819	0.743	0.637	5.52E-10	6.64E-02	0.541	0.645	0.637	1.00E+00
ATP5IF1	1.77E-06	0.696	0.658	0.590	5.69E-02	1.93E-05	0.409	0.643	0.590	6.20E-01	2.66E-01	0.186	0.613	0.590	1.00E+00
ATP5MF	1.60E-13	1.128	0.649	0.445	5.16E-09	3.91E-02	0.318	0.456	0.445	1.00E+00	3.85E-02	0.480	0.548	0.445	1.00E+00
ATP6V1E1	9.23E-01	0.223	0.297	0.326	1.00E+00	1.13E-01	0.119	0.345	0.326	1.00E+00	3.51E-02	0.278	0.484	0.326	1.00E+00
AURKAIP1	1.50E-06	0.794	0.568	0.475	4.84E-02	3.85E-11	0.827	0.608	0.475	1.24E-06	4.26E-02	0.344	0.581	0.475	1.00E+00
BAD	8.67E-01	0.112	0.306	0.350	1.00E+00	2.48E-11	0.687	0.509	0.350	8.00E-07	4.84E-03	0.891	0.516	0.350	1.00E+00
BAX	1.03E-02	0.526	0.396	0.346	1.00E+00	4.35E-23	1.224	0.585	0.346	1.40E-18	2.86E-01	0.599	0.419	0.346	1.00E+00
BCAP31	3.29E-01	0.223	0.477	0.527	1.00E+00	5.24E-13	0.701	0.655	0.527	1.69E-08	1.94E-01	0.512	0.581	0.527	1.00E+00
BEX3	9.50E-08	0.712	0.523	0.377	3.06E-03	2.16E-06	0.513	0.474	0.377	6.95E-02	2.28E-01	0.377	0.452	0.377	1.00E+00
C17orf49	4.44E-01	0.217	0.514	0.545	1.00E+00	2.97E-07	0.610	0.614	0.545	9.55E-03	2.14E-01	0.249	0.581	0.545	1.00E+00
C4orf48	2.68E-36	0.984	0.342	0.052	8.64E-32	6.39E-78	1.262	0.433	0.052	2.06E-73	6.92E-03	0.244	0.161	0.052	1.00E+00
CALR	2.38E-04	0.542	0.676	0.657	1.00E+00	9.14E-21	1.088	0.778	0.657	2.94E-16	1.17E-02	0.554	0.710	0.657	1.00E+00
CAMK1D	3.51E-03	0.453	0.198	0.119	1.00E+00	6.41E-05	0.381	0.211	0.119	1.00E+00	1.01E-06	0.648	0.387	0.119	3.24E-02
CARD16	1.48E-09	0.768	0.505	0.302	4.75E-05	1.21E-02	0.237	0.357	0.302	1.00E+00	2.31E-01	0.536	0.355	0.302	1.00E+00

CAVIN3	1.75E-14	1.184	0.721	0.592	5.64E-10	1.49E-46	1.829	0.830	0.592	4.81E-42	2.40E-05	1.440	0.710	0.592	7.75E-01
CCDC124	2.50E-05	0.541	0.459	0.338	8.04E-01	1.05E-19	0.803	0.573	0.338	3.39E-15	8.52E-03	0.551	0.516	0.338	1.00E+00
CCDC85B	1.52E-08	1.145	0.739	0.716	4.91E-04	4.09E-35	1.253	0.848	0.716	1.32E-30	6.34E-04	0.859	0.774	0.716	1.00E+00
CDC42EP1	1.35E-01	0.240	0.324	0.297	1.00E+00	7.46E-04	0.437	0.363	0.297	1.00E+00	7.84E-02	0.372	0.419	0.297	1.00E+00
CDIPT	9.17E-01	0.127	0.306	0.346	1.00E+00	2.44E-03	0.406	0.392	0.346	1.00E+00	1.16E-01	0.103	0.452	0.346	1.00E+00
CETP	2.98E-04	0.146	0.126	0.050	1.00E+00	2.31E-04	0.285	0.111	0.050	1.00E+00	6.99E-06	0.333	0.226	0.050	2.25E-01
CFI	4.39E-01	0.253	0.315	0.322	1.00E+00	3.05E-02	0.264	0.368	0.322	1.00E+00	2.07E-01	0.123	0.419	0.322	1.00E+00
CFL1	2.93E-07	0.731	0.829	0.833	9.44E-03	2.88E-28	0.823	0.924	0.833	9.27E-24	3.07E-04	0.511	0.903	0.833	1.00E+00
CHMP6	9.14E-04	0.246	0.198	0.107	1.00E+00	1.25E-09	0.493	0.246	0.107	4.04E-05	5.64E-03	0.357	0.258	0.107	1.00E+00
CIAO2B	2.06E-01	0.170	0.450	0.474	1.00E+00	3.26E-06	0.374	0.567	0.474	1.05E-01	8.56E-01	0.112	0.452	0.474	1.00E+00
CINP	7.44E-01	0.131	0.144	0.140	1.00E+00	3.35E-05	0.440	0.240	0.140	1.00E+00	6.37E-01	0.116	0.161	0.140	1.00E+00
CLEC14A	4.31E-01	0.225	0.559	0.616	1.00E+00	4.24E-23	1.402	0.772	0.616	1.37E-18	2.47E-03	0.949	0.774	0.616	1.00E+00
CLTB	5.69E-01	0.246	0.432	0.494	1.00E+00	6.87E-12	0.754	0.643	0.494	2.21E-07	1.58E-01	0.378	0.484	0.494	1.00E+00
CNDP2	1.97E-01	0.226	0.225	0.190	1.00E+00	1.86E-05	0.446	0.298	0.190	5.99E-01	3.44E-05	0.611	0.452	0.190	1.00E+00
CNIH1	8.17E-01	0.221	0.351	0.416	1.00E+00	9.89E-07	0.600	0.509	0.416	3.18E-02	6.21E-01	0.128	0.419	0.416	1.00E+00
CNPY4	6.88E-03	0.218	0.135	0.072	1.00E+00	9.43E-07	0.223	0.170	0.072	3.04E-02	1.99E-01	0.242	0.129	0.072	1.00E+00
COL15A1	3.57E-08	1.157	0.658	0.568	1.15E-03	6.43E-05	0.685	0.614	0.568	1.00E+00	2.58E-04	1.295	0.677	0.568	1.00E+00
COL4A1	7.05E-57	3.446	0.901	0.435	2.27E-52	4.93E-35	1.686	0.760	0.435	1.59E-30	7.27E-04	1.384	0.645	0.435	1.00E+00
COL4A2	1.56E-67	3.518	0.955	0.448	5.03E-63	8.89E-34	1.749	0.754	0.448	2.86E-29	4.87E-10	2.017	0.806	0.448	1.57E-05
COMMD4	1.57E-03	0.392	0.333	0.236	1.00E+00	5.61E-05	0.404	0.333	0.236	1.00E+00	8.59E-02	0.122	0.355	0.236	1.00E+00
COPE	2.06E-04	0.586	0.550	0.482	1.00E+00	9.39E-11	0.776	0.608	0.482	3.02E-06	2.85E-01	0.182	0.548	0.482	1.00E+00
CORO1B	1.17E-02	0.285	0.387	0.326	1.00E+00	3.80E-13	0.769	0.503	0.326	1.22E-08	3.17E-02	0.422	0.452	0.326	1.00E+00
COTL1	1.38E-09	0.919	0.532	0.344	4.46E-05	9.06E-01	0.557	0.275	0.344	1.00E+00	3.75E-05	1.633	0.548	0.344	1.00E+00
COX5A	4.29E-05	0.593	0.631	0.571	1.00E+00	4.82E-18	0.903	0.713	0.571	1.55E-13	1.62E-01	0.363	0.613	0.571	1.00E+00
CRIP2	1.01E-19	1.141	0.892	0.810	3.24E-15	3.28E-53	1.644	0.912	0.810	1.06E-48	2.53E-08	0.958	0.935	0.810	8.14E-04
CSTB	3.69E-11	1.016	0.730	0.636	1.19E-06	1.54E-09	0.616	0.743	0.636	4.97E-05	9.22E-01	0.110	0.645	0.636	1.00E+00
CTSA	2.75E-01	0.159	0.270	0.359	1.00E+00	7.64E-08	0.479	0.485	0.359	2.46E-03	5.03E-02	0.280	0.484	0.359	1.00E+00
CTSB	1.50E-07	0.969	0.541	0.430	4.84E-03	1.91E-20	1.101	0.632	0.430	6.17E-16	1.73E-02	0.583	0.516	0.430	1.00E+00
CTSS	6.96E-04	0.602	0.297	0.197	1.00E+00	1.10E-08	0.599	0.351	0.197	3.54E-04	1.46E-01	0.248	0.290	0.197	1.00E+00
CYBA	1.83E-10	0.901	0.505	0.290	5.91E-06	6.33E-25	1.016	0.585	0.290	2.04E-20	1.81E-02	0.582	0.484	0.290	1.00E+00
CYBC1	4.43E-02	0.233	0.270	0.212	1.00E+00	1.92E-06	0.391	0.333	0.212	6.20E-02	1.10E-02	0.219	0.387	0.212	1.00E+00
DBI	6.90E-02	0.331	0.568	0.609	1.00E+00	3.70E-09	0.614	0.690	0.609	1.19E-04	2.63E-01	0.172	0.613	0.609	1.00E+00
DBN1	1.24E-19	0.799	0.432	0.146	3.98E-15	2.19E-09	0.528	0.292	0.146	7.05E-05	1.90E-03	0.617	0.323	0.146	1.00E+00
DDRGK1	9.67E-01	0.195	0.279	0.309	1.00E+00	1.03E-13	0.752	0.497	0.309	3.32E-09	4.13E-02	0.201	0.452	0.309	1.00E+00
DECR1	8.43E-01	0.200	0.315	0.350	1.00E+00	6.21E-03	0.434	0.398	0.350	1.00E+00	6.27E-01	0.216	0.355	0.350	1.00E+00
DMAC1	2.18E-01	0.217	0.297	0.269	1.00E+00	2.54E-05	0.442	0.374	0.269	8.19E-01	1.41E-01	0.196	0.387	0.269	1.00E+00
DNAJC15	6.54E-01	0.115	0.360	0.388	1.00E+00	1.15E-13	0.970	0.550	0.388	3.69E-09	1.04E-02	0.500	0.548	0.388	1.00E+00
DNMT1	2.40E-02	0.293	0.225	0.159	1.00E+00	5.41E-05	0.386	0.257	0.159	1.00E+00	2.51E-01	0.187	0.226	0.159	1.00E+00
DYSF	1.31E-34	1.336	0.577	0.176	4.21E-30	2.56E-10	0.778	0.333	0.176	8.24E-06	9.83E-03	0.528	0.323	0.176	1.00E+00
EDF1	3.54E-02	0.269	0.730	0.777	1.00E+00	2.88E-20	0.764	0.901	0.777	9.26E-16	1.94E-01	0.230	0.774	0.777	1.00E+00
EGFL7	3.06E-12	1.078	0.820	0.672	9.85E-08	1.19E-23	0.949	0.825	0.672	3.84E-19	8.29E-04	0.763	0.839	0.672	1.00E+00
ELOB	9.71E-14	1.029	0.784	0.704	3.13E-09	9.28E-07	0.469	0.737	0.704	2.99E-02	1.88E-03	0.601	0.806	0.704	1.00E+00
ENG	2.96E-19	1.240	0.829	0.660	9.53E-15	4.95E-79	2.521	0.947	0.660	1.59E-74	5.44E-07	1.466	0.806	0.660	1.75E-02
EPSTI1	2.90E-04	0.499	0.153	0.068	1.00E+00	3.84E-03	0.276	0.123	0.068	1.00E+00	3.29E-02	0.731	0.161	0.068	1.00E+00
FAAP20	8.80E-09	0.879	0.550	0.375	2.83E-04	5.15E-08	0.661	0.491	0.375	1.66E-03	1.13E-01	0.417	0.452	0.375	1.00E+00
FAM167B	5.08E-21	1.361	0.423	0.139	1.64E-16	4.01E-12	1.042	0.304	0.139	1.29E-07	1.39E-03	0.497	0.323	0.139	1.00E+00
FAM174C	6.65E-02	0.235	0.315	0.274	1.00E+00	9.86E-07	0.479	0.386	0.274	3.17E-02	8.65E-01	0.101	0.258	0.274	1.00E+00
FAM207A	8.34E-04	0.310	0.198	0.107	1.00E+00	1.51E-03	0.308	0.175	0.107	1.00E+00	2.75E-02	0.107	0.226	0.107	1.00E+00
FAM50A	1.10E-06	0.580	0.459	0.314	3.53E-02	9.92E-05	0.338	0.398	0.314	1.00E+00	8.07E-02	0.347	0.419	0.314	1.00E+00
FBXW5	1.00E-01	0.318	0.342	0.314	1.00E+00	6.61E-07	0.616	0.427	0.314	2.13E-02	2.23E-01	0.216	0.387	0.314	1.00E+00
FDPS	7.37E-06	0.490	0.559	0.446	2.37E-01	2.72E-14	0.731	0.632	0.446	8.75E-10	8.44E-03	0.468	0.613	0.446	1.00E+00

FKBP10	1.05E-05	0.695	0.360	0.229	3.38E-01	3.80E-03	0.294	0.292	0.229	1.00E+00	1.10E-01	0.338	0.323	0.229	1.00E+00
FKBP1A	1.20E-04	0.790	0.757	0.818	1.00E+00	6.64E-11	0.531	0.860	0.818	2.14E-06	2.49E-06	1.112	0.935	0.818	8.01E-02
G6PC3	9.30E-03	0.255	0.207	0.134	1.00E+00	7.63E-13	0.547	0.310	0.134	2.46E-08	3.18E-02	0.241	0.258	0.134	1.00E+00
GADD45GIP1	3.20E-04	0.700	0.559	0.493	1.00E+00	1.17E-29	1.290	0.725	0.493	3.77E-25	1.20E-03	0.978	0.645	0.493	1.00E+00
GAPDH	1.47E-04	0.498	0.892	0.915	1.00E+00	2.53E-24	0.790	0.971	0.915	8.14E-20	2.20E-01	0.303	0.968	0.915	1.00E+00
GET3	3.26E-01	0.188	0.306	0.293	1.00E+00	1.68E-07	0.386	0.427	0.293	5.41E-03	4.30E-01	0.217	0.226	0.293	1.00E+00
GPR137	1.32E-01	0.170	0.180	0.143	1.00E+00	2.46E-05	0.339	0.240	0.143	7.92E-01	2.97E-02	0.376	0.258	0.143	1.00E+00
GRAP	2.94E-33	1.044	0.351	0.060	9.46E-29	5.18E-06	0.294	0.146	0.060	1.67E-01	1.61E-02	0.342	0.161	0.060	1.00E+00
GRN	8.19E-08	0.658	0.631	0.522	2.64E-03	4.00E-24	1.051	0.719	0.522	1.29E-19	1.94E-02	0.606	0.581	0.522	1.00E+00
GUK1	4.35E-01	0.113	0.712	0.778	1.00E+00	9.92E-17	0.725	0.848	0.778	3.20E-12	3.76E-03	0.576	0.806	0.778	1.00E+00
H2AFJ	8.94E-02	0.322	0.432	0.404	1.00E+00	9.70E-11	0.834	0.550	0.404	3.12E-06	9.59E-03	0.603	0.548	0.404	1.00E+00
H2AFV	9.18E-01	0.106	0.378	0.440	1.00E+00	5.45E-01	0.156	0.398	0.440	1.00E+00	4.53E-03	0.786	0.613	0.440	1.00E+00
HAGLROS	7.21E-05	0.369	0.198	0.094	1.00E+00	6.34E-22	0.805	0.310	0.094	2.04E-17	9.86E-03	0.203	0.226	0.094	1.00E+00
HCLS1	2.27E-26	0.702	0.351	0.075	7.30E-22	3.41E-22	0.781	0.281	0.075	1.10E-17	1.36E-03	0.248	0.226	0.075	1.00E+00
HLA-C	1.33E-02	0.139	0.937	0.913	1.00E+00	1.23E-20	0.684	0.942	0.913	3.97E-16	7.18E-07	0.884	0.935	0.913	2.31E-02
HM13	3.95E-04	0.500	0.432	0.344	1.00E+00	1.85E-10	0.622	0.497	0.344	5.95E-06	1.24E-04	0.705	0.581	0.344	1.00E+00
HMOX2	9.80E-15	0.846	0.541	0.288	3.16E-10	2.64E-13	0.903	0.474	0.288	8.51E-09	1.97E-02	0.426	0.452	0.288	1.00E+00
HPRT1	3.08E-01	0.138	0.171	0.225	1.00E+00	1.67E-01	0.135	0.246	0.225	1.00E+00	1.60E-01	0.310	0.323	0.225	1.00E+00
HSPG2	9.46E-52	2.865	0.919	0.554	3.05E-47	3.82E-40	1.592	0.819	0.554	1.23E-35	1.16E-15	2.231	0.935	0.554	3.73E-11
HYAL2	2.03E-05	0.619	0.523	0.414	6.54E-01	1.99E-07	0.537	0.550	0.414	6.42E-03	6.04E-01	0.182	0.419	0.414	1.00E+00
ICAM2	1.28E-08	0.871	0.577	0.438	4.12E-04	7.52E-42	1.603	0.778	0.438	2.42E-37	1.71E-04	0.998	0.645	0.438	1.00E+00
IFI30	6.09E-03	0.494	0.216	0.140	1.00E+00	2.91E-06	0.709	0.251	0.140	9.37E-02	9.69E-01	0.109	0.129	0.140	1.00E+00
IFITM2	4.42E-01	0.230	0.802	0.863	1.00E+00	8.42E-50	1.171	0.959	0.863	2.71E-45	3.28E-03	0.499	0.903	0.863	1.00E+00
IFITM3	1.01E-03	0.515	0.892	0.942	1.00E+00	7.30E-91	1.816	0.994	0.942	2.35E-86	1.53E-04	0.619	1.000	0.942	1.00E+00
IGFBP7	1.80E-14	1.333	0.901	0.913	5.80E-10	8.23E-18	1.716	0.965	0.913	2.65E-13	5.82E-09	1.376	0.968	0.913	1.87E-04
IL18BP	1.06E-06	0.560	0.243	0.107	3.41E-02	3.82E-44	1.022	0.444	0.107	1.23E-39	3.13E-05	0.747	0.323	0.107	1.00E+00
IL32	2.55E-45	2.701	0.883	0.467	8.21E-41	1.20E-38	2.144	0.754	0.467	3.88E-34	2.03E-01	0.399	0.548	0.467	1.00E+00
IL3RA	2.27E-02	0.398	0.505	0.493	1.00E+00	3.45E-33	1.460	0.737	0.493	1.11E-28	9.10E-02	0.376	0.548	0.493	1.00E+00
INSR	4.02E-21	1.393	0.459	0.158	1.29E-16	1.25E-03	0.512	0.234	0.158	1.00E+00	1.58E-04	0.969	0.387	0.158	1.00E+00
ISG15	2.45E-02	0.216	0.495	0.443	1.00E+00	1.31E-03	0.324	0.515	0.443	1.00E+00	6.39E-01	0.201	0.355	0.443	1.00E+00
KRTCAP2	4.31E-01	0.195	0.595	0.686	1.00E+00	3.07E-12	0.639	0.754	0.686	9.90E-08	2.62E-01	0.294	0.677	0.686	1.00E+00
LAMB1	5.89E-55	2.165	0.739	0.212	1.90E-50	3.64E-13	0.614	0.415	0.212	1.17E-08	6.60E-07	0.850	0.548	0.212	2.13E-02
LAMTOR2	7.57E-04	0.448	0.459	0.387	1.00E+00	4.39E-14	0.712	0.573	0.387	1.41E-09	8.71E-01	0.148	0.355	0.387	1.00E+00
LAYN	1.08E-03	0.333	0.234	0.134	1.00E+00	3.30E-05	0.469	0.234	0.134	1.00E+00	2.61E-01	0.379	0.194	0.134	1.00E+00
LGALS9	1.34E-22	1.176	0.514	0.193	4.32E-18	3.26E-24	1.027	0.468	0.193	1.05E-19	1.33E-10	1.444	0.581	0.193	4.27E-06
LGMN	8.06E-02	0.287	0.315	0.273	1.00E+00	6.89E-04	0.527	0.345	0.273	1.00E+00	4.38E-01	0.148	0.323	0.273	1.00E+00
LMAN2	1.65E-01	0.291	0.414	0.424	1.00E+00	1.74E-05	0.542	0.503	0.424	5.61E-01	1.32E-01	0.307	0.516	0.424	1.00E+00
LSM7	1.08E-02	0.332	0.523	0.490	1.00E+00	4.15E-02	0.296	0.497	0.490	1.00E+00	9.73E-01	0.331	0.419	0.490	1.00E+00
MAP1LC3A	4.79E-02	0.460	0.396	0.375	1.00E+00	6.94E-11	0.761	0.526	0.375	2.24E-06	5.47E-01	0.274	0.387	0.375	1.00E+00
MAPK12	2.83E-01	0.109	0.126	0.100	1.00E+00	3.00E-02	0.276	0.146	0.100	1.00E+00	2.31E-01	0.154	0.161	0.100	1.00E+00
MGAT4A	7.26E-01	0.200	0.189	0.197	1.00E+00	1.14E-01	0.249	0.135	0.197	1.00E+00	1.51E-02	0.405	0.355	0.197	1.00E+00
MGP	1.27E-01	0.578	0.640	0.644	1.00E+00	2.66E-19	2.396	0.749	0.644	8.57E-15	3.64E-03	1.328	0.742	0.644	1.00E+00
MICOS10	2.47E-01	0.284	0.514	0.563	1.00E+00	1.30E-02	0.229	0.573	0.563	1.00E+00	1.87E-01	0.373	0.581	0.563	1.00E+00
MIEN1	2.06E-01	0.234	0.315	0.299	1.00E+00	6.28E-10	0.614	0.450	0.299	2.02E-05	5.17E-02	0.215	0.419	0.299	1.00E+00
MIF	4.87E-18	1.455	0.820	0.801	1.57E-13	1.78E-42	1.353	0.912	0.801	5.72E-38	2.16E-02	0.751	0.839	0.801	1.00E+00
MLEC	1.20E-19	1.170	0.468	0.177	3.88E-15	1.31E-33	1.447	0.497	0.177	4.21E-29	7.19E-04	0.318	0.387	0.177	1.00E+00
MPG	1.51E-01	0.255	0.369	0.361	1.00E+00	1.77E-13	0.799	0.544	0.361	5.70E-09	3.64E-02	0.235	0.516	0.361	1.00E+00
MRPL17	3.39E-06	0.744	0.414	0.268	1.09E-01	1.24E-52	1.788	0.678	0.268	3.98E-48	4.24E-01	0.351	0.290	0.268	1.00E+00
MRPL20	9.80E-02	0.185	0.477	0.467	1.00E+00	2.26E-06	0.503	0.550	0.467	7.29E-02	3.59E-01	0.163	0.516	0.467	1.00E+00
MRPL34	2.48E-01	0.105	0.306	0.289	1.00E+00	1.61E-06	0.508	0.398	0.289	5.17E-02	1.76E-01	0.223	0.355	0.289	1.00E+00
MRPL52	6.93E-03	0.407	0.459	0.407	1.00E+00	2.47E-03	0.404	0.444	0.407	1.00E+00	2.35E-01	0.357	0.452	0.407	1.00E+00

MRPS15	8.70E-02	0.344	0.360	0.339	1.00E+00	7.58E-06	0.557	0.439	0.339	2.44E-01	2.12E-02	0.712	0.484	0.339	1.00E+00
MRPS16	4.82E-01	0.148	0.207	0.257	1.00E+00	4.07E-06	0.487	0.368	0.257	1.31E-01	8.53E-01	0.163	0.258	0.257	1.00E+00
MRPS21	3.26E-06	0.545	0.613	0.507	1.05E-01	9.00E-04	0.268	0.561	0.507	1.00E+00	3.76E-01	0.202	0.548	0.507	1.00E+00
MSRB2	5.56E-04	0.307	0.207	0.113	1.00E+00	5.94E-02	0.128	0.152	0.113	1.00E+00	1.20E-01	0.399	0.194	0.113	1.00E+00
MTCH2	7.45E-01	0.119	0.198	0.200	1.00E+00	5.13E-05	0.377	0.298	0.200	1.00E+00	5.07E-02	0.212	0.323	0.200	1.00E+00
MTLN	1.74E-02	0.278	0.387	0.320	1.00E+00	5.90E-03	0.358	0.374	0.320	1.00E+00	2.93E-01	0.438	0.387	0.320	1.00E+00
MYDGF	3.47E-01	0.175	0.441	0.483	1.00E+00	2.20E-14	0.848	0.632	0.483	7.07E-10	1.79E-01	0.317	0.548	0.483	1.00E+00
MYL6	5.27E-11	0.838	0.910	0.916	1.70E-06	2.61E-18	0.685	0.936	0.916	8.39E-14	1.05E-03	0.825	0.935	0.916	1.00E+00
NAA38	1.02E-02	0.421	0.523	0.508	1.00E+00	8.10E-03	0.328	0.526	0.508	1.00E+00	3.35E-04	1.124	0.645	0.508	1.00E+00
NDUFA11	3.01E-03	0.335	0.613	0.603	1.00E+00	6.50E-21	0.937	0.766	0.603	2.09E-16	3.78E-01	0.474	0.548	0.603	1.00E+00
NDUFA13	8.79E-08	0.710	0.712	0.630	2.83E-03	3.61E-15	0.668	0.760	0.630	1.16E-10	3.95E-01	0.153	0.613	0.630	1.00E+00
NDUFA6	3.15E-02	0.367	0.468	0.447	1.00E+00	4.90E-11	0.627	0.596	0.447	1.58E-06	2.43E-01	0.352	0.484	0.447	1.00E+00
NDUFA8	8.83E-01	0.156	0.333	0.370	1.00E+00	3.13E-06	0.469	0.474	0.370	1.01E-01	9.48E-01	0.269	0.323	0.370	1.00E+00
NDUFB11	6.99E-01	0.198	0.505	0.565	1.00E+00	6.24E-10	0.649	0.649	0.565	2.01E-05	1.26E-01	0.299	0.613	0.565	1.00E+00
NDUFB2	1.19E-05	0.554	0.640	0.587	3.85E-01	2.27E-10	0.709	0.678	0.587	7.31E-06	4.75E-02	0.318	0.677	0.587	1.00E+00
NDUFS5	1.04E-01	0.265	0.622	0.678	1.00E+00	1.65E-03	0.379	0.684	0.678	1.00E+00	5.45E-01	0.294	0.613	0.678	1.00E+00
NDUFS6	2.08E-02	0.262	0.505	0.483	1.00E+00	3.77E-06	0.476	0.567	0.483	1.21E-01	7.48E-02	0.149	0.581	0.483	1.00E+00
NDUFS7	5.41E-03	0.502	0.459	0.403	1.00E+00	6.89E-13	0.768	0.573	0.403	2.22E-08	4.65E-03	0.479	0.581	0.403	1.00E+00
NDUFS8	1.62E-03	0.414	0.450	0.393	1.00E+00	6.22E-12	0.696	0.550	0.393	2.00E-07	8.92E-02	0.471	0.484	0.393	1.00E+00
NECAP2	6.85E-01	0.250	0.288	0.318	1.00E+00	5.58E-02	0.253	0.339	0.318	1.00E+00	8.42E-01	0.240	0.290	0.318	1.00E+00
NEDD8	8.33E-01	0.143	0.586	0.693	1.00E+00	3.32E-09	0.395	0.766	0.693	1.07E-04	5.68E-01	0.185	0.645	0.693	1.00E+00
NME4	4.46E-09	0.795	0.441	0.258	1.44E-04	1.88E-18	0.950	0.485	0.258	6.07E-14	1.12E-01	0.385	0.355	0.258	1.00E+00
NUDT14	5.19E-02	0.325	0.378	0.348	1.00E+00	2.94E-25	1.015	0.614	0.348	9.46E-21	8.46E-02	0.236	0.452	0.348	1.00E+00
NUDT16	8.08E-01	0.290	0.225	0.241	1.00E+00	3.77E-01	0.111	0.246	0.241	1.00E+00	5.99E-01	0.119	0.194	0.241	1.00E+00
NUDT5	4.76E-03	0.457	0.297	0.212	1.00E+00	1.77E-06	0.419	0.333	0.212	5.71E-02	3.87E-01	0.297	0.258	0.212	1.00E+00
OAS2	8.58E-01	0.172	0.117	0.128	1.00E+00	7.43E-01	0.138	0.111	0.128	1.00E+00	8.35E-02	0.196	0.226	0.128	1.00E+00
OPTN	1.72E-10	0.914	0.586	0.416	5.55E-06	9.40E-06	0.494	0.503	0.416	3.03E-01	5.78E-03	0.516	0.581	0.416	1.00E+00
OST4	1.74E-11	0.874	0.766	0.688	5.60E-07	2.00E-05	0.326	0.696	0.688	6.43E-01	4.54E-01	0.139	0.613	0.688	1.00E+00
P4HB	1.06E-06	0.688	0.586	0.507	3.41E-02	5.34E-11	0.697	0.626	0.507	1.72E-06	2.35E-03	0.580	0.645	0.507	1.00E+00
PARVB	1.15E-01	0.302	0.342	0.322	1.00E+00	2.86E-19	0.766	0.561	0.322	9.22E-15	6.46E-04	0.509	0.516	0.322	1.00E+00
PAXX	2.54E-01	0.192	0.279	0.257	1.00E+00	4.41E-22	0.889	0.520	0.257	1.42E-17	1.73E-03	0.595	0.452	0.257	1.00E+00
PDIA3	1.15E-02	0.408	0.604	0.624	1.00E+00	3.41E-07	0.484	0.684	0.624	1.10E-02	4.65E-01	0.126	0.613	0.624	1.00E+00
PDLIM2	2.90E-02	0.317	0.378	0.338	1.00E+00	7.10E-03	0.336	0.386	0.338	1.00E+00	1.92E-01	0.228	0.419	0.338	1.00E+00
PDLIM7	1.02E-16	1.201	0.486	0.217	3.30E-12	1.60E-14	0.857	0.415	0.217	5.15E-10	1.70E-03	0.429	0.419	0.217	1.00E+00
PFN1	1.46E-12	1.088	0.838	0.815	4.69E-08	1.01E-44	1.279	0.906	0.815	3.24E-40	1.28E-05	0.922	0.903	0.815	4.11E-01
PGAM1	7.28E-02	0.393	0.441	0.458	1.00E+00	2.25E-14	0.906	0.614	0.458	7.24E-10	2.63E-03	0.600	0.581	0.458	1.00E+00
PKM	1.85E-01	0.157	0.604	0.642	1.00E+00	3.17E-04	0.495	0.667	0.642	1.00E+00	8.98E-01	0.216	0.581	0.642	1.00E+00
PLOD1	7.79E-16	1.077	0.405	0.160	2.51E-11	4.88E-34	1.151	0.474	0.160	1.57E-29	5.21E-05	0.683	0.387	0.160	1.00E+00
PLVAP	1.80E-03	0.534	0.712	0.708	1.00E+00	6.01E-13	0.934	0.825	0.708	1.93E-08	8.17E-04	1.037	0.806	0.708	1.00E+00
POGLUT1	7.38E-01	0.161	0.126	0.123	1.00E+00	8.25E-01	0.199	0.111	0.123	1.00E+00	5.37E-02	0.344	0.226	0.123	1.00E+00
POLD4	1.80E-02	0.330	0.405	0.358	1.00E+00	6.57E-10	0.624	0.503	0.358	2.12E-05	2.90E-02	0.244	0.516	0.358	1.00E+00
PON2	4.03E-07	0.685	0.468	0.319	1.30E-02	6.84E-01	0.107	0.298	0.319	1.00E+00	5.30E-02	0.562	0.419	0.319	1.00E+00
PPIB	1.35E-17	1.169	0.784	0.670	4.36E-13	7.27E-50	1.648	0.871	0.670	2.34E-45	2.83E-04	0.904	0.806	0.670	1.00E+00
PPP4C	8.19E-03	0.392	0.459	0.416	1.00E+00	1.18E-07	0.576	0.538	0.416	3.79E-03	3.92E-01	0.298	0.484	0.416	1.00E+00
PRADC1	1.13E-01	0.119	0.144	0.102	1.00E+00	2.32E-08	0.329	0.228	0.102	7.49E-04	2.33E-01	0.166	0.161	0.102	1.00E+00
PRKAR2A	1.67E-01	0.128	0.144	0.215	1.00E+00	8.26E-01	0.179	0.199	0.215	1.00E+00	4.58E-01	0.112	0.258	0.215	1.00E+00
PSMB10	8.55E-03	0.555	0.360	0.304	1.00E+00	3.82E-14	0.854	0.503	0.304	1.23E-09	3.00E-01	0.431	0.355	0.304	1.00E+00
PSMB6	4.76E-01	0.213	0.477	0.542	1.00E+00	1.24E-18	0.856	0.713	0.542	4.00E-14	3.75E-01	0.348	0.548	0.542	1.00E+00
PSMD13	6.58E-02	0.308	0.378	0.341	1.00E+00	7.84E-05	0.474	0.433	0.341	1.00E+00	2.99E-01	0.252	0.419	0.341	1.00E+00
PUF60	2.52E-01	0.330	0.333	0.339	1.00E+00	1.54E-04	0.358	0.415	0.339	1.00E+00	2.44E-01	0.247	0.419	0.339	1.00E+00
PXDN	2.45E-27	2.038	0.577	0.233	7.89E-23	1.10E-02	0.468	0.281	0.233	1.00E+00	3.85E-01	0.312	0.290	0.233	1.00E+00

PYCARD	3.64E-03	0.401	0.333	0.245	1.00E+00	1.38E-21	0.998	0.509	0.245	4.43E-17	4.05E-01	0.172	0.290	0.245	1.00E+00
RAB13	7.54E-05	0.582	0.667	0.699	1.00E+00	8.14E-40	1.551	0.877	0.699	2.62E-35	1.75E-03	0.698	0.742	0.699	1.00E+00
RAB32	1.27E-03	0.392	0.225	0.131	1.00E+00	2.61E-09	0.491	0.275	0.131	8.41E-05	7.95E-06	0.698	0.387	0.131	2.56E-01
RABAC1	2.32E-02	0.355	0.622	0.667	1.00E+00	1.12E-30	1.156	0.807	0.667	3.62E-26	5.74E-02	0.302	0.710	0.667	1.00E+00
RAI14	3.23E-16	1.161	0.559	0.289	1.04E-11	7.13E-18	0.955	0.520	0.289	2.30E-13	1.97E-06	1.121	0.581	0.289	6.36E-02
RFXANK	2.45E-06	0.402	0.369	0.218	7.88E-02	2.22E-05	0.373	0.322	0.218	7.14E-01	2.30E-01	0.317	0.290	0.218	1.00E+00
RGCC	1.74E-12	1.040	0.577	0.306	5.61E-08	1.08E-01	0.117	0.351	0.306	1.00E+00	1.64E-01	0.558	0.387	0.306	1.00E+00
RGS3	7.36E-68	2.715	0.802	0.221	2.37E-63	9.09E-37	1.448	0.561	0.221	2.93E-32	2.11E-05	0.894	0.484	0.221	6.81E-01
RHOG	8.50E-01	0.190	0.279	0.316	1.00E+00	2.42E-09	0.670	0.462	0.316	7.80E-05	9.40E-04	0.752	0.516	0.316	1.00E+00
RNASEK	3.48E-04	0.370	0.721	0.735	1.00E+00	1.81E-42	1.260	0.865	0.735	5.83E-38	1.09E-06	0.875	0.903	0.735	3.51E-02
RNF145	2.27E-07	0.735	0.495	0.347	7.31E-03	5.91E-02	0.204	0.380	0.347	1.00E+00	1.18E-06	1.013	0.645	0.347	3.79E-02
RNF7	3.87E-01	0.199	0.423	0.473	1.00E+00	4.93E-22	0.984	0.678	0.473	1.59E-17	5.92E-03	0.624	0.581	0.473	1.00E+00
ROMO1	9.32E-09	1.008	0.595	0.476	3.00E-04	1.88E-02	0.285	0.497	0.476	1.00E+00	1.43E-02	0.594	0.548	0.476	1.00E+00
RPL28	7.99E-05	0.545	0.964	0.951	1.00E+00	1.51E-28	0.906	1.000	0.951	4.86E-24	8.30E-02	0.458	0.968	0.951	1.00E+00
RPS19BP1	1.84E-02	0.399	0.495	0.483	1.00E+00	3.13E-10	0.745	0.602	0.483	1.01E-05	4.09E-02	0.505	0.581	0.483	1.00E+00
S100A11	1.02E-08	0.773	0.838	0.812	3.29E-04	2.96E-49	1.543	0.912	0.812	9.53E-45	3.12E-02	0.730	0.806	0.812	1.00E+00
S100A13	8.35E-10	0.956	0.748	0.679	2.69E-05	1.49E-04	0.476	0.702	0.679	1.00E+00	1.41E-04	0.915	0.742	0.679	1.00E+00
SCAND1	1.84E-07	0.764	0.568	0.449	5.93E-03	7.03E-19	1.024	0.637	0.449	2.27E-14	6.45E-01	0.176	0.419	0.449	1.00E+00
SDF2L1	6.07E-01	0.175	0.261	0.265	1.00E+00	2.90E-04	0.300	0.363	0.265	1.00E+00	1.22E-01	0.557	0.355	0.265	1.00E+00
SEC61B	2.32E-04	0.497	0.712	0.681	1.00E+00	1.67E-09	0.491	0.743	0.681	5.39E-05	2.10E-02	0.412	0.710	0.681	1.00E+00
SELENOM	5.06E-01	0.214	0.523	0.586	1.00E+00	1.40E-52	1.818	0.865	0.586	4.51E-48	2.03E-04	0.928	0.710	0.586	1.00E+00
SERF2	2.27E-18	1.046	0.901	0.863	7.31E-14	1.05E-66	1.401	0.959	0.863	3.39E-62	2.23E-05	0.920	0.871	0.863	7.19E-01
SERPINB6	8.97E-01	0.270	0.396	0.459	1.00E+00	8.63E-11	0.650	0.579	0.459	2.78E-06	5.78E-04	0.821	0.645	0.459	1.00E+00
SERPINH1	2.03E-02	0.293	0.649	0.616	1.00E+00	3.36E-14	0.923	0.719	0.616	1.08E-09	5.15E-01	0.149	0.645	0.616	1.00E+00
SH3BGRL3	4.63E-07	0.589	0.793	0.758	1.49E-02	6.55E-58	1.548	0.924	0.758	2.11E-53	5.63E-04	0.936	0.806	0.758	1.00E+00
SHISA5	1.22E-01	0.265	0.405	0.401	1.00E+00	5.37E-10	0.668	0.520	0.401	1.73E-05	1.32E-01	0.375	0.484	0.401	1.00E+00
SHKBP1	2.11E-02	0.143	0.279	0.209	1.00E+00	2.08E-03	0.290	0.281	0.209	1.00E+00	2.62E-03	0.196	0.419	0.209	1.00E+00
SIRT6	1.17E-03	0.195	0.180	0.093	1.00E+00	8.55E-04	0.194	0.164	0.093	1.00E+00	6.87E-03	0.610	0.226	0.093	1.00E+00
SLC25A39	2.92E-04	0.533	0.342	0.235	1.00E+00	1.88E-14	0.726	0.439	0.235	6.06E-10	9.35E-04	0.531	0.452	0.235	1.00E+00
SLC35B1	6.44E-01	0.102	0.189	0.223	1.00E+00	2.20E-01	0.178	0.240	0.223	1.00E+00	4.32E-01	0.158	0.258	0.223	1.00E+00
SLC39A3	6.73E-06	0.328	0.234	0.107	2.17E-01	1.82E-13	0.374	0.281	0.107	5.85E-09	5.14E-06	0.819	0.355	0.107	1.65E-01
SLCO2A1	3.01E-02	0.116	0.090	0.178	1.00E+00	5.58E-05	0.774	0.275	0.178	1.00E+00	1.56E-02	0.946	0.323	0.178	1.00E+00
SMIM29	5.18E-01	0.163	0.180	0.169	1.00E+00	1.65E-04	0.392	0.257	0.169	1.00E+00	1.50E-02	0.313	0.323	0.169	1.00E+00
SNRPC	4.54E-01	0.132	0.333	0.336	1.00E+00	1.69E-11	0.537	0.515	0.336	5.46E-07	3.07E-02	0.464	0.484	0.336	1.00E+00
SOX18	4.36E-29	1.520	0.730	0.369	1.40E-24	4.16E-36	1.420	0.708	0.369	1.34E-31	1.14E-07	1.524	0.677	0.369	3.67E-03
SPARC	8.04E-10	1.340	0.775	0.770	2.59E-05	3.43E-25	1.167	0.912	0.770	1.11E-20	8.25E-07	1.374	0.935	0.770	2.66E-02
SPCS3	7.10E-02	0.305	0.333	0.288	1.00E+00	5.72E-07	0.412	0.415	0.288	1.84E-02	8.71E-02	0.368	0.387	0.288	1.00E+00
SSBP4	9.87E-03	0.341	0.315	0.245	1.00E+00	1.34E-01	0.132	0.175	0.245	1.00E+00	4.36E-01	0.108	0.290	0.245	1.00E+00
SSNA1	1.49E-07	0.564	0.514	0.353	4.79E-03	8.39E-10	0.619	0.503	0.353	2.70E-05	3.45E-03	0.573	0.516	0.353	1.00E+00
SSR4	6.42E-05	0.481	0.676	0.634	1.00E+00	4.66E-21	0.917	0.760	0.634	1.50E-16	1.29E-01	0.374	0.645	0.634	1.00E+00
STK25	4.58E-02	0.354	0.342	0.297	1.00E+00	1.88E-03	0.377	0.357	0.297	1.00E+00	5.04E-01	0.299	0.355	0.297	1.00E+00
STMP1	1.65E-07	0.673	0.396	0.229	5.31E-03	4.08E-15	0.678	0.444	0.229	1.31E-10	3.26E-04	0.305	0.484	0.229	1.00E+00
SYNPO	1.63E-06	0.682	0.396	0.249	5.24E-02	3.43E-11	0.840	0.421	0.249	1.10E-06	9.09E-01	0.425	0.226	0.249	1.00E+00
TAPBP	3.40E-02	0.428	0.495	0.515	1.00E+00	4.47E-04	0.320	0.567	0.515	1.00E+00	3.34E-03	0.389	0.645	0.515	1.00E+00
TAX1BP3	4.96E-01	0.178	0.342	0.366	1.00E+00	1.26E-13	0.720	0.556	0.366	4.05E-09	1.41E-01	0.248	0.452	0.366	1.00E+00
TCIM	2.27E-02	0.223	0.378	0.293	1.00E+00	1.46E-12	0.736	0.520	0.293	4.71E-08	1.59E-02	0.623	0.452	0.293	1.00E+00
TEX264	6.74E-01	0.117	0.324	0.388	1.00E+00	1.37E-11	0.793	0.538	0.388	4.41E-07	2.16E-01	0.230	0.452	0.388	1.00E+00
TGFB1	2.58E-11	0.845	0.477	0.264	8.32E-07	8.76E-06	0.492	0.374	0.264	2.82E-01	1.46E-01	0.375	0.355	0.264	1.00E+00
TIMM13	4.34E-02	0.251	0.450	0.427	1.00E+00	1.50E-05	0.564	0.497	0.427	4.82E-01	1.12E-01	0.624	0.516	0.427	1.00E+00
TIMP1	5.01E-01	1.232	0.649	0.680	1.00E+00	2.06E-24	1.608	0.801	0.680	6.63E-20	2.19E-04	1.437	0.806	0.680	1.00E+00
TKT	4.30E-01	0.341	0.351	0.370	1.00E+00	1.38E-07	0.697	0.468	0.370	4.44E-03	1.09E-01	0.341	0.484	0.370	1.00E+00

TMED9	3.63E-01	0.157	0.450	0.479	1.00E+00	1.04E-19	0.924	0.673	0.479	3.34E-15	3.95E-02	0.384	0.613	0.479	1.00E+00
TMEM160	6.63E-01	0.139	0.315	0.329	1.00E+00	2.07E-07	0.594	0.462	0.329	6.66E-03	1.48E-01	0.364	0.419	0.329	1.00E+00
TMEM204	1.41E-04	0.542	0.468	0.351	1.00E+00	2.05E-30	1.162	0.673	0.351	6.60E-26	2.78E-04	0.870	0.581	0.351	1.00E+00
TMEM255B	1.35E-09	0.657	0.541	0.364	4.36E-05	6.82E-14	0.840	0.544	0.364	2.20E-09	5.74E-02	0.225	0.484	0.364	1.00E+00
TMSB10	9.02E-28	1.305	0.982	0.972	2.90E-23	6.42E-16	0.600	0.994	0.972	2.07E-11	2.38E-03	0.538	1.000	0.972	1.00E+00
TNFRSF1B	6.84E-02	0.397	0.279	0.233	1.00E+00	8.57E-15	0.870	0.439	0.233	2.76E-10	4.68E-04	0.547	0.484	0.233	1.00E+00
TNFRSF6B	6.54E-18	1.065	0.532	0.222	2.11E-13	1.28E-105	3.841	0.836	0.222	4.12E-101	1.78E-11	2.512	0.645	0.222	5.72E-07
TPI1	2.83E-02	0.418	0.649	0.690	1.00E+00	2.12E-32	1.175	0.842	0.690	6.84E-28	9.43E-03	0.512	0.774	0.690	1.00E+00
TPM4	1.15E-09	0.821	0.775	0.699	3.69E-05	1.27E-14	0.802	0.789	0.699	4.09E-10	2.95E-02	0.393	0.742	0.699	1.00E+00
TRAPPC1	6.15E-03	0.290	0.441	0.388	1.00E+00	1.20E-19	0.961	0.608	0.388	3.88E-15	2.50E-01	0.241	0.452	0.388	1.00E+00
TRIOBP	4.85E-03	0.436	0.405	0.336	1.00E+00	1.83E-02	0.368	0.363	0.336	1.00E+00	7.07E-01	0.200	0.355	0.336	1.00E+00
TTYH3	4.02E-18	0.447	0.261	0.059	1.29E-13	1.10E-05	0.261	0.140	0.059	3.53E-01	1.31E-02	0.181	0.161	0.059	1.00E+00
TUBB6	1.46E-15	1.112	0.622	0.380	4.71E-11	6.04E-07	0.537	0.497	0.380	1.95E-02	1.31E-01	0.426	0.452	0.380	1.00E+00
TWF2	3.36E-02	0.275	0.333	0.286	1.00E+00	7.10E-06	0.557	0.386	0.286	2.29E-01	2.52E-04	0.482	0.548	0.286	1.00E+00
TXNDC17	3.68E-01	0.160	0.378	0.404	1.00E+00	2.78E-03	0.313	0.450	0.404	1.00E+00	1.19E-01	0.512	0.484	0.404	1.00E+00
TXNDC5	4.94E-01	0.183	0.207	0.201	1.00E+00	6.33E-01	0.132	0.199	0.201	1.00E+00	5.33E-01	0.281	0.226	0.201	1.00E+00
UBE2J1	1.04E-01	0.243	0.396	0.377	1.00E+00	6.23E-13	0.788	0.550	0.377	2.01E-08	2.58E-01	0.318	0.419	0.377	1.00E+00
UBE2L3	1.35E-01	0.278	0.450	0.454	1.00E+00	4.49E-03	0.322	0.474	0.454	1.00E+00	8.71E-01	0.502	0.419	0.454	1.00E+00
UBL5	3.74E-06	0.700	0.694	0.652	1.20E-01	2.90E-03	0.322	0.673	0.652	1.00E+00	6.25E-03	0.748	0.710	0.652	1.00E+00
UFD1	4.10E-01	0.233	0.333	0.352	1.00E+00	1.47E-04	0.434	0.421	0.352	1.00E+00	8.08E-04	0.603	0.548	0.352	1.00E+00
UQCR10	1.98E-03	0.429	0.495	0.423	1.00E+00	2.86E-06	0.425	0.515	0.423	9.21E-02	6.80E-02	0.444	0.548	0.423	1.00E+00
UQCR11	6.08E-04	0.597	0.631	0.641	1.00E+00	1.27E-09	0.633	0.725	0.641	4.10E-05	1.54E-07	1.164	0.839	0.641	4.95E-03
UROD	8.42E-02	0.233	0.315	0.274	1.00E+00	9.50E-05	0.447	0.363	0.274	1.00E+00	1.05E-01	0.378	0.387	0.274	1.00E+00
VKORC1	6.42E-01	0.100	0.432	0.483	1.00E+00	1.13E-12	0.666	0.620	0.483	3.64E-08	4.25E-02	0.325	0.581	0.483	1.00E+00
VPS25	1.73E-04	0.197	0.243	0.128	1.00E+00	8.34E-09	0.552	0.269	0.128	2.69E-04	9.37E-02	0.103	0.226	0.128	1.00E+00
VWA1	1.58E-34	2.156	0.685	0.297	5.10E-30	3.05E-33	1.617	0.614	0.297	9.83E-29	1.29E-13	2.287	0.742	0.297	4.17E-09
WDR83OS	4.41E-01	0.216	0.559	0.642	1.00E+00	2.57E-12	0.589	0.719	0.642	8.29E-08	1.91E-02	0.448	0.677	0.642	1.00E+00
XAF1	1.32E-05	1.040	0.396	0.260	4.24E-01	3.83E-04	0.364	0.351	0.260	1.00E+00	4.59E-02	0.588	0.387	0.260	1.00E+00
YIPF3	4.92E-01	0.190	0.378	0.390	1.00E+00	4.96E-09	0.563	0.515	0.390	1.60E-04	1.76E-01	0.262	0.484	0.390	1.00E+00
ZNF428	2.00E-08	0.704	0.523	0.357	6.43E-04	2.14E-18	0.772	0.585	0.357	6.89E-14	2.22E-02	0.171	0.516	0.357	1.00E+00
ZNF580	1.57E-01	0.294	0.279	0.255	1.00E+00	3.55E-07	0.524	0.380	0.255	1.14E-02	1.55E-01	0.148	0.355	0.255	1.00E+00
ZNHIT1	2.57E-02	0.367	0.559	0.558	1.00E+00	2.49E-04	0.363	0.591	0.558	1.00E+00	1.62E-02	0.572	0.645	0.558	1.00E+00

Table S6. Connectome analysis of T lymphocyte-focused intercellular communication in the TME of advanced MF.

A. MF samples combined

source	target	ligand	receptor	pair	mode	p_val_adj.lig	p_val_adj.rec
B Cells	T lymphocytes	CD70	CD27	CD70 - CD27	UNCAT	2.93E-39	0
B Cells	T lymphocytes	HLA-C	CD3D	HLA-C - CD3D	MHC	1.46E-55	0
B Cells	T lymphocytes	HLA-B	CD3D	HLA-B - CD3D	MHC	2.90E-14	0
B Cells	T lymphocytes	HLA-B	CD3G	HLA-B - CD3G	MHC	2.90E-14	0
B Cells	T lymphocytes	HLA-C	CD3G	HLA-C - CD3G	MHC	1.46E-55	0
B Cells	T lymphocytes	ICAM3	ITGAL	ICAM3 - ITGAL	Cell-cell adhesion	7.43E-18	0
B Cells	T lymphocytes	SEMA7A	ITGB1	SEMA7A - ITGB1	Semaphorins	2.37E-38	0
B Cells	T lymphocytes	ICAM3	ITGB2	ICAM3 - ITGB2	Cell-cell adhesion	7.43E-18	0
B Cells	T lymphocytes	HLA-E	KLRC1	HLA-E - KLRC1	MHC	5.41E-26	0
B Cells	T lymphocytes	TNF	TNFRSF1B	TNF - TNFRSF1B	TNF	8.82E-29	0
Dendritic Cell/Macrophages	T lymphocytes	CCL13	CXCR3	CCL13 - CXCR3	CC	0	0
Dendritic Cell/Macrophages	T lymphocytes	CXCL9	CXCR3	CXCL9 - CXCR3	CXCL	0	0
Dendritic Cell/Macrophages	T lymphocytes	CXCL10	CXCR3	CXCL10 - CXCR3	CXCL	0	0
Dendritic Cell/Macrophages	T lymphocytes	ICAM1	IL2RG	ICAM1 - IL2RG	Cell-cell adhesion	0	0
Dendritic Cell/Macrophages	T lymphocytes	LYZ	ITGAL	LYZ - ITGAL	UNCAT	0	0
Dendritic Cell/Macrophages	T lymphocytes	F13A1	ITGB1	F13A1 - ITGB1	UNCAT	0	0
Dendritic Cell/Macrophages	T lymphocytes	CD14	ITGB1	CD14 - ITGB1	UNCAT	0	0
Dendritic Cell/Macrophages	T lymphocytes	MMP9	ITGB2	MMP9 - ITGB2	MMP	0	0
Dendritic Cell/Macrophages	T lymphocytes	ICAM1	ITGB2	ICAM1 - ITGB2	Cell-cell adhesion	0	0
Dendritic Cell/Macrophages	T lymphocytes	APOE	SORL1	APOE - SORL1	APOE	1.22E-294	0
Endothelial Cells	T lymphocytes	ICAM2	ITGAL	ICAM2 - ITGAL	Cell-cell adhesion	0	0
Endothelial Cells	T lymphocytes	COL4A1	ITGB1	COL4A1 - ITGB1	Collagens	0	0
Endothelial Cells	T lymphocytes	LAMB1	ITGB1	LAMB1 - ITGB1	Laminins	0	0
Endothelial Cells	T lymphocytes	HSPG2	ITGB1	HSPG2 - ITGB1	Matrix glycoproteins	0	0
Endothelial Cells	T lymphocytes	COL18A1	ITGB1	COL18A1 - ITGB1	Collagens	0	0
Endothelial Cells	T lymphocytes	TGM2	ITGB1	TGM2 - ITGB1	UNCAT	0	0
Endothelial Cells	T lymphocytes	LAMA5	ITGB1	LAMA5 - ITGB1	Laminins	0	0
Endothelial Cells	T lymphocytes	ICAM2	ITGB2	ICAM2 - ITGB2	Cell-cell adhesion	0	0
Endothelial Cells	T lymphocytes	CD34	SELL	CD34 - SELL	Cell-cell adhesion	0	0
Endothelial Cells	T lymphocytes	PODXL	SELL	PODXL - SELL	Cell-cell adhesion	0	0
Fibroblasts	T lymphocytes	FBN1	ITGB1	FBN1 - ITGB1	UNCAT	0	0
Fibroblasts	T lymphocytes	TNC	ITGB1	TNC - ITGB1	UNCAT	0	0
Fibroblasts	T lymphocytes	COL6A1	ITGB1	COL6A1 - ITGB1	Collagens	0	0
Fibroblasts	T lymphocytes	FBLN1	ITGB1	FBLN1 - ITGB1	UNCAT	0	0
Fibroblasts	T lymphocytes	COL1A1	ITGB1	COL1A1 - ITGB1	Collagens	0	0
Fibroblasts	T lymphocytes	COL3A1	ITGB1	COL3A1 - ITGB1	Collagens	0	0
Fibroblasts	T lymphocytes	COL5A2	ITGB1	COL5A2 - ITGB1	Collagens	0	0

Fibroblasts	T lymphocytes	COL6A3	ITGB1	COL6A3 - ITGB1	Collagens	0	0
Fibroblasts	T lymphocytes	COL6A2	ITGB1	COL6A2 - ITGB1	Collagens	0	0
Fibroblasts	T lymphocytes	COL1A2	ITGB1	COL1A2 - ITGB1	Collagens	0	0
Keratinocytes	T lymphocytes	IL18	CD48	IL18 - CD48	Interleukins	0	0
Keratinocytes	T lymphocytes	ZG16B	CXCR4	ZG16B - CXCR4	UNCAT	0	0
Keratinocytes	T lymphocytes	LGALS3BP	ITGB1	LGALS3BP - ITGB1	UNCAT	0	0
Keratinocytes	T lymphocytes	ADAM15	ITGB1	ADAM15 - ITGB1	ADAM	0	0
Keratinocytes	T lymphocytes	COL7A1	ITGB1	COL7A1 - ITGB1	Collagens	0	0
Keratinocytes	T lymphocytes	LAMB3	ITGB1	LAMB3 - ITGB1	Laminins	0	0
Keratinocytes	T lymphocytes	PLAT	ITGB2	PLAT - ITGB2	Vasoactive	0	0
T lymphocytes	T lymphocytes	HLA-C	CD3D	HLA-C - CD3D	MHC	0	0
T lymphocytes	T lymphocytes	HLA-C	CD3G	HLA-C - CD3G	MHC	0	0
T lymphocytes	T lymphocytes	CCL5	CXCR3	CCL5 - CXCR3	CC	0	0
T lymphocytes	T lymphocytes	TGFB1	CXCR4	TGFB1 - CXCR4	TGFB	0	0
T lymphocytes	T lymphocytes	ICAM3	ITGAL	ICAM3 - ITGAL	Cell-cell adhesion	0	0
T lymphocytes	T lymphocytes	CD40LG	ITGB2	CD40LG - ITGB2	UNCAT	0	0
T lymphocytes	T lymphocytes	ICAM3	ITGB2	ICAM3 - ITGB2	Cell-cell adhesion	0	0
T lymphocytes	T lymphocytes	HLA-E	KLRC1	HLA-E - KLRC1	MHC	0	0

B. Healthy Control samples combined

source	target	ligand	receptor	pair	mode	p_val_adj.lig	p_val_adj.rec
Dendritic Cell/Macrophages	T lymphocytes	HLA-B	CD3D	HLA-B - CD3D	MHC	8.14E-68	0
Dendritic Cell/Macrophages	T lymphocytes	B2M	CD3D	B2M - CD3D	UNCAT	9.63E-36	0
Dendritic Cell/Macrophages	T lymphocytes	MMP9	CD44	MMP9 - CD44	MMP	0	4.91E-62
Dendritic Cell/Macrophages	T lymphocytes	TGFB1	CXCR4	TGFB1 - CXCR4	TGFB	1.58E-101	0
Dendritic Cell/Macrophages	T lymphocytes	CXCL16	CXCR6	CXCL16 - CXCR6	CXCL	0	0
Dendritic Cell/Macrophages	T lymphocytes	IL10	IL10RA	IL10 - IL10RA	Interleukins	0	0
Dendritic Cell/Macrophages	T lymphocytes	ICAM1	IL2RG	ICAM1 - IL2RG	Cell-cell adhesion	2.32E-260	0
Dendritic Cell/Macrophages	T lymphocytes	LYZ	ITGAL	LYZ - ITGAL	UNCAT	0	0
Dendritic Cell/Macrophages	T lymphocytes	MMP9	ITGB2	MMP9 - ITGB2	MMP	0	0
Dendritic Cell/Macrophages	T lymphocytes	TNF	TNFRSF1B	TNF - TNFRSF1B	TNF	0	0
Endothelial Cells	T lymphocytes	HLA-B	CD3D	HLA-B - CD3D	MHC	0	0
Endothelial Cells	T lymphocytes	B2M	CD3D	B2M - CD3D	UNCAT	0	0
Endothelial Cells	T lymphocytes	HLA-C	CD3D	HLA-C - CD3D	MHC	0	0
Endothelial Cells	T lymphocytes	HLA-A	CD3D	HLA-A - CD3D	MHC	0	0
Endothelial Cells	T lymphocytes	HLA-B	CD3G	HLA-B - CD3G	MHC	0	0
Endothelial Cells	T lymphocytes	HLA-A	CD3G	HLA-A - CD3G	MHC	0	0
Endothelial Cells	T lymphocytes	HLA-C	CD3G	HLA-C - CD3G	MHC	0	0
Endothelial Cells	T lymphocytes	B2M	CD3G	B2M - CD3G	UNCAT	0	0
Endothelial Cells	T lymphocytes	ICAM1	IL2RG	ICAM1 - IL2RG	Cell-cell adhesion	0	0
Endothelial Cells	T lymphocytes	IL15	IL2RG	IL15 - IL2RG	Interleukins	0	0
Fibroblasts	T lymphocytes	CXCL12	CCR4	CXCL12 - CCR4	CC	0	0

Fibroblasts	T lymphocytes	COL1A1	CD44	COL1A1 - CD44	Collagens	0	4.91E-62
Fibroblasts	T lymphocytes	COL1A2	CD44	COL1A2 - CD44	Collagens	0	4.91E-62
Fibroblasts	T lymphocytes	CXCL12	CXCR4	CXCL12 - CXCR4	CXCL	0	0
Fibroblasts	T lymphocytes	THBS2	ITGA4	THBS2 - ITGA4	Matrix glycoproteins	0	1.20E-109
Fibroblasts	T lymphocytes	VCAN	ITGA4	VCAN - ITGA4	UNCAT	0	1.20E-109
Fibroblasts	T lymphocytes	C3	ITGB2	C3 - ITGB2	Complement	0	0
Fibroblasts	T lymphocytes	F10	ITGB2	F10 - ITGB2	UNCAT	0	0
Fibroblasts	T lymphocytes	SPON2	ITGB2	SPON2 - ITGB2	UNCAT	0	0
Fibroblasts	T lymphocytes	FN1	ITGB7	FN1 - ITGB7	Fibronectin	0	2.18E-215
Keratinocytes	T lymphocytes	DEFB1	CCR6	DEFB1 - CCR6	CC	0	0
Keratinocytes	T lymphocytes	IL18	CD48	IL18 - CD48	Interleukins	0	0
Keratinocytes	T lymphocytes	CDH1	ITGB7	CDH1 - ITGB7	Cell-cell adhesion	0	2.18E-215
T lymphocytes	T lymphocytes	B2M	CD3D	B2M - CD3D	UNCAT	0	0
T lymphocytes	T lymphocytes	HLA-B	CD3D	HLA-B - CD3D	MHC	0	0
T lymphocytes	T lymphocytes	HLA-A	CD3D	HLA-A - CD3D	MHC	4.70E-301	0
T lymphocytes	T lymphocytes	HLA-C	CD3D	HLA-C - CD3D	MHC	8.48E-281	0
T lymphocytes	T lymphocytes	HLA-B	CD3G	HLA-B - CD3G	MHC	0	0
T lymphocytes	T lymphocytes	HLA-C	CD3G	HLA-C - CD3G	MHC	8.48E-281	0
T lymphocytes	T lymphocytes	TGFB1	CXCR4	TGFB1 - CXCR4	TGFB	1.94E-140	0
T lymphocytes	T lymphocytes	ICAM3	ITGAL	ICAM3 - ITGAL	Cell-cell adhesion	0	0
T lymphocytes	T lymphocytes	ICAM3	ITGB2	ICAM3 - ITGB2	Cell-cell adhesion	0	0
T lymphocytes	T lymphocytes	CD40LG	ITGB2	CD40LG - ITGB2	UNCAT	0	0

C. AD samples combined

source	target	ligand	receptor	pair	mode	p_val_adj.lig	p_val_adj.rec
Dendritic Cell/Macrophages	T lymphocytes	CXCL16	CXCR6	CXCL16 - CXCR6	CXCL	0	0
Dendritic Cell/Macrophages	T lymphocytes	ICAM1	IL2RA	ICAM1 - IL2RA	Cell-cell adhesion	0	0
Dendritic Cell/Macrophages	T lymphocytes	ICAM1	IL2RG	ICAM1 - IL2RG	Cell-cell adhesion	0	0
Dendritic Cell/Macrophages	T lymphocytes	GNAI2	S1PR4	GNAI2 - S1PR4	UNCAT	0	4.13E-146
Endothelial Cells	T lymphocytes	HLA-B	CD3D	HLA-B - CD3D	MHC	1.38E-60	0
Endothelial Cells	T lymphocytes	HLA-C	CD3D	HLA-C - CD3D	MHC	8.06E-28	0
Endothelial Cells	T lymphocytes	HLA-A	CD3D	HLA-A - CD3D	MHC	1.53E-20	0
Endothelial Cells	T lymphocytes	HLA-C	CD3G	HLA-C - CD3G	MHC	8.06E-28	0
Endothelial Cells	T lymphocytes	HLA-A	CD3G	HLA-A - CD3G	MHC	1.53E-20	0
Endothelial Cells	T lymphocytes	HLA-B	CD3G	HLA-B - CD3G	MHC	1.38E-60	0
Endothelial Cells	T lymphocytes	CXCL12	CXCR4	CXCL12 - CXCR4	CXCL	4.36E-203	0
Endothelial Cells	T lymphocytes	ICAM1	IL2RA	ICAM1 - IL2RA	Cell-cell adhesion	7.86E-51	0
Endothelial Cells	T lymphocytes	ICAM1	IL2RG	ICAM1 - IL2RG	Cell-cell adhesion	7.86E-51	0
Endothelial Cells	T lymphocytes	IL15	IL2RG	IL15 - IL2RG	Interleukins	1.05E-36	0
Fibroblasts	T lymphocytes	CD70	CD27	CD70 - CD27	UNCAT	1.46E-99	0
Fibroblasts	T lymphocytes	CXCL12	CXCR4	CXCL12 - CXCR4	CXCL	0	0
Fibroblasts	T lymphocytes	VCAM1	ITGB7	VCAM1 - ITGB7	Cell-cell adhesion	4.51E-288	3.69E-118

Fibroblasts	T lymphocytes	FN1	ITGB7	FN1 - ITGB7	Fibronectin	0	3.69E-118
Fibroblasts	T lymphocytes	LAMB2	RPSA	LAMB2 - RPSA	Laminins	0	6.97E-236
Fibroblasts	T lymphocytes	LAMA2	RPSA	LAMA2 - RPSA	Laminins	0	6.97E-236
Fibroblasts	T lymphocytes	TNFSF12	TNFRSF25	TNFSF12 - TNFRSF25	TNF	1.07E-34	5.27E-121
Keratinocytes	DEFB1	CCR6	DEFB1 - CCR6	CC	DEFB1	0	2.06E-155
Keratinocytes	IL18	CD48	IL18 - CD48	Interleukins	IL18	0	0
Keratinocytes	ZG16B	CXCR4	ZG16B - CXCR4	UNCAT	ZG16B	0	0
Keratinocytes	CDH1	ITGB7	CDH1 - ITGB7	Cell-cell adhesion	CDH1	0	3.69E-118
T lymphocytes	T lymphocytes	B2M	CD247	B2M - CD247	UNCAT	0	0
T lymphocytes	T lymphocytes	HLA-C	CD3D	HLA-C - CD3D	MHC	0	0
T lymphocytes	T lymphocytes	B2M	CD3D	B2M - CD3D	UNCAT	0	0
T lymphocytes	T lymphocytes	HLA-A	CD3D	HLA-A - CD3D	MHC	0	0
T lymphocytes	T lymphocytes	HLA-B	CD3D	HLA-B - CD3D	MHC	0	0
T lymphocytes	T lymphocytes	HLA-A	CD3G	HLA-A - CD3G	MHC	0	0
T lymphocytes	T lymphocytes	HLA-C	CD3G	HLA-C - CD3G	MHC	0	0
T lymphocytes	T lymphocytes	B2M	CD3G	B2M - CD3G	UNCAT	0	0

D. PS samples combined

**source**	**target**	**ligand**	**receptor**	**pair**	**mode**	**p_val_adj.lig**	**p_val_adj.rec**
Dendritic Cell/Macrophages	T lymphocytes	LYZ	ITGAL	LYZ - ITGAL	UNCAT	1.80E-79	2.19E-12
Dendritic Cell/Macrophages	T lymphocytes	MMP9	ITGB2	MMP9 - ITGB2	MMP	1.09E-22	1.41E-19
Endothelial Cells	T lymphocytes	IL15	IL2RG	IL15 - IL2RG	Interleukins	0.00011103	2.36E-23
Endothelial Cells	T lymphocytes	ICAM1	IL2RG	ICAM1 - IL2RG	Cell-cell adhesion	9.74E-51	2.36E-23
Endothelial Cells	T lymphocytes	ICAM1	ITGAL	ICAM1 - ITGAL	Cell-cell adhesion	9.74E-51	2.19E-12
Endothelial Cells	T lymphocytes	ICAM2	ITGAL	ICAM2 - ITGAL	Cell-cell adhesion	2.57E-33	2.19E-12
Endothelial Cells	T lymphocytes	CYR61	ITGB2	CYR61 - ITGB2	Matrix (assorted)	2.21E-07	1.41E-19
Endothelial Cells	T lymphocytes	ICAM1	ITGB2	ICAM1 - ITGB2	Cell-cell adhesion	9.74E-51	1.41E-19
Endothelial Cells	T lymphocytes	ICAM2	ITGB2	ICAM2 - ITGB2	Cell-cell adhesion	2.57E-33	1.41E-19
Endothelial Cells	T lymphocytes	CTGF	ITGB2	CTGF - ITGB2	Matrix (assorted)	6.37E-90	1.41E-19
Endothelial Cells	T lymphocytes	TNFSF12	TNFRSF25	TNFSF12 - TNFRSF25	TNF	3.90E-05	0.02036471
Keratinocytes	T lymphocytes	IL18	CD48	IL18 - CD48	Interleukins	1.18E-51	6.92E-41
Keratinocytes	T lymphocytes	PLAU	ITGB2	PLAU - ITGB2	Vasoactive	2.18E-24	1.41E-19
Keratinocytes	T lymphocytes	PLAT	ITGB2	PLAT - ITGB2	Vasoactive	4.52E-36	1.41E-19
T lymphocytes	T lymphocytes	B2M	CD247	B2M - CD247	UNCAT	3.62E-62	8.26E-22
T lymphocytes	T lymphocytes	B2M	CD3D	B2M - CD3D	UNCAT	3.62E-62	2.05E-93
T lymphocytes	T lymphocytes	B2M	CD3G	B2M - CD3G	UNCAT	3.62E-62	6.71E-52
T lymphocytes	T lymphocytes	ICAM3	ITGAL	ICAM3 - ITGAL	Cell-cell adhesion	7.23E-21	2.19E-12
T lymphocytes	T lymphocytes	ICAM3	ITGB2	ICAM3 - ITGB2	Cell-cell adhesion	7.23E-21	1.41E-19

E. MF17 Clonotype 1

**source**	**target**	**ligand**	**receptor**	**pair**	**mode**	**p_val_adj.lig**	**p_val_adj.rec**
B Cells	MF17 Clonotype 1	BMP8B	ACVR2A	BMP8B - ACVR2A	BMP	4.03E-55	5.20E-71
B Cells	MF17 Clonotype 1	BMP6	ACVR2A	BMP6 - ACVR2A	BMP	7.58E-97	5.20E-71

B Cells	MF17 Clonotype 1	COL4A3	CD47	COL4A3 - CD47	Collagens	3.33E-42	5.41E-18
B Cells	MF17 Clonotype 1	EBI3	IL27RA	EBI3 - IL27RA	UNCAT	1.01E-17	3.99E-23
B Cells	MF17 Clonotype 1	IL15	IL2RG	IL15 - IL2RG	Interleukins	3.73E-32	9.12E-19
B Cells	MF17 Clonotype 1	EBI3	IL6ST	EBI3 - IL6ST	UNCAT	1.01E-17	5.90E-44
B Cells	MF17 Clonotype 1	COL4A3	ITGB1	COL4A3 - ITGB1	Collagens	3.33E-42	8.57E-114
B Cells	MF17 Clonotype 1	ICAM4	ITGB1	ICAM4 - ITGB1	Cell-cell adhesion	1.14E-66	8.57E-114
B Cells	MF17 Clonotype 1	LAMA5	ITGB1	LAMA5 - ITGB1	Laminins	2.40E-30	8.57E-114
B Cells	MF17 Clonotype 1	ICAM4	ITGB2	ICAM4 - ITGB2	Cell-cell adhesion	1.14E-66	6.52E-47
MF17 Clonotype 1	MF17 Clonotype 1	BTLA	CD247	BTLA - CD247	TNF	2.63E-61	1.18E-20
MF17 Clonotype 1	MF17 Clonotype 1	CD70	CD27	CD70 - CD27	UNCAT	6.20E-35	7.75E-73
MF17 Clonotype 1	MF17 Clonotype 1	HLA-C	CD3D	HLA-C - CD3D	MHC	0.003984053	8.94E-63
MF17 Clonotype 1	MF17 Clonotype 1	HLA-B	CD3D	HLA-B - CD3D	MHC	4.94E-06	8.94E-63
MF17 Clonotype 1	MF17 Clonotype 1	IL16	CD4	IL16 - CD4	Interleukins	2.17E-60	2.86E-45
MF17 Clonotype 1	MF17 Clonotype 1	PKM	CD44	PKM - CD44	UNCAT	5.33E-44	2.10E-20
MF17 Clonotype 1	MF17 Clonotype 1	VIM	CD44	VIM - CD44	UNCAT	2.55E-85	2.10E-20
MF17 Clonotype 1	MF17 Clonotype 1	CD40LG	ITGB2	CD40LG - ITGB2	UNCAT	2.68E-116	6.52E-47
MF17 Clonotype 1	MF17 Clonotype 1	ICAM3	ITGB2	ICAM3 - ITGB2	Cell-cell adhesion	9.80E-70	6.52E-47
MF17 Clonotype 1	MF17 Clonotype 1	PODXL2	SELL	PODXL2 - SELL	Cell-cell adhesion	3.77E-19	9.21E-140
Dendritic Cell/Macrophages	MF17 Clonotype 1	MMP9	CD44	MMP9 - CD44	MMP	3.89E-101	2.10E-20
Dendritic Cell/Macrophages	MF17 Clonotype 1	IL18	CD48	IL18 - CD48	Interleukins	1.60E-241	6.44E-36
Dendritic Cell/Macrophages	MF17 Clonotype 1	EBI3	IL27RA	EBI3 - IL27RA	UNCAT	1.08E-90	3.99E-23
Dendritic Cell/Macrophages	MF17 Clonotype 1	ICAM1	IL2RG	ICAM1 - IL2RG	Cell-cell adhesion	3.32E-47	9.12E-19
Dendritic Cell/Macrophages	MF17 Clonotype 1	EBI3	IL6ST	EBI3 - IL6ST	UNCAT	1.08E-90	5.90E-44
Dendritic Cell/Macrophages	MF17 Clonotype 1	F13A1	ITGB1	F13A1 - ITGB1	UNCAT	9.31E-146	8.57E-114
Dendritic Cell/Macrophages	MF17 Clonotype 1	CD14	ITGB1	CD14 - ITGB1	UNCAT	9.85E-246	8.57E-114
Dendritic Cell/Macrophages	MF17 Clonotype 1	MMP9	ITGB2	MMP9 - ITGB2	MMP	3.89E-101	6.52E-47
Dendritic Cell/Macrophages	MF17 Clonotype 1	ICAM1	ITGB2	ICAM1 - ITGB2	Cell-cell adhesion	3.32E-47	6.52E-47
Dendritic Cell/Macrophages	MF17 Clonotype 1	APOE	SORL1	APOE - SORL1	APOE	3.82E-126	8.79E-50
Endothelial Cells	MF17 Clonotype 1	COL4A1	CD47	COL4A1 - CD47	Collagens	8.40E-86	5.41E-18
Endothelial Cells	MF17 Clonotype 1	ICAM1	IL2RG	ICAM1 - IL2RG	Cell-cell adhesion	1.12E-21	9.12E-19
Endothelial Cells	MF17 Clonotype 1	COL4A1	ITGB1	COL4A1 - ITGB1	Collagens	8.40E-86	8.57E-114
Endothelial Cells	MF17 Clonotype 1	LAMB1	ITGB1	LAMB1 - ITGB1	Laminins	1.12E-91	8.57E-114
Endothelial Cells	MF17 Clonotype 1	HSPG2	ITGB1	HSPG2 - ITGB1	Matrix glycoproteins	4.63E-23	8.57E-114
Endothelial Cells	MF17 Clonotype 1	LAMA5	ITGB1	LAMA5 - ITGB1	Laminins	1.32E-60	8.57E-114
Endothelial Cells	MF17 Clonotype 1	ICAM1	ITGB2	ICAM1 - ITGB2	Cell-cell adhesion	1.12E-21	6.52E-47
Endothelial Cells	MF17 Clonotype 1	LAMB2	RPSA	LAMB2 - RPSA	Laminins	2.55E-42	9.78E-115
Endothelial Cells	MF17 Clonotype 1	CD34	SELL	CD34 - SELL	Cell-cell adhesion	0	9.21E-140
Endothelial Cells	MF17 Clonotype 1	PODXL	SELL	PODXL - SELL	Cell-cell adhesion	1.57E-56	9.21E-140
Fibroblasts	MF17 Clonotype 1	CCL2	CCR4	CCL2 - CCR4	CC	0	1.79E-10
Fibroblasts	MF17 Clonotype 1	CCL19	CCR7	CCL19 - CCR7	CC	0	1.64E-95
Fibroblasts	MF17 Clonotype 1	COL1A2	CD44	COL1A2 - CD44	Collagens	0	2.10E-20

Fibroblasts	MF17 Clonotype 1	COL1A1	CD44	COL1A1 - CD44	Collagens	1.41E-167	2.10E-20
Fibroblasts	MF17 Clonotype 1	COL6A1	ITGB1	COL6A1 - ITGB1	Collagens	0	8.57E-114
Fibroblasts	MF17 Clonotype 1	COL3A1	ITGB1	COL3A1 - ITGB1	Collagens	0	8.57E-114
Fibroblasts	MF17 Clonotype 1	COL1A1	ITGB1	COL1A1 - ITGB1	Collagens	1.41E-167	8.57E-114
Fibroblasts	MF17 Clonotype 1	TNC	ITGB1	TNC - ITGB1	UNCAT	0	8.57E-114
Fibroblasts	MF17 Clonotype 1	COL1A2	ITGB1	COL1A2 - ITGB1	Collagens	0	8.57E-114
Fibroblasts	MF17 Clonotype 1	CFH	SELL	CFH - SELL	Complement	0	9.21E-140
Keratinocytes	MF17 Clonotype 1	BMP2	ACVR2A	BMP2 - ACVR2A	BMP	1.87E-237	5.20E-71
Keratinocytes	MF17 Clonotype 1	CDH1	ITGAE	CDH1 - ITGAE	Cell-cell adhesion	0	0.02204917
Keratinocytes	MF17 Clonotype 1	ADAM15	ITGB1	ADAM15 - ITGB1	ADAM	3.44E-76	8.57E-114
Keratinocytes	MF17 Clonotype 1	LAMB3	ITGB1	LAMB3 - ITGB1	Laminins	1.61E-255	8.57E-114
Keratinocytes	MF17 Clonotype 1	LGALS3BP	ITGB1	LGALS3BP - ITGB1	UNCAT	2.48E-147	8.57E-114
Keratinocytes	MF17 Clonotype 1	COL4A5	ITGB1	COL4A5 - ITGB1	Collagens	5.27E-256	8.57E-114
Keratinocytes	MF17 Clonotype 1	LAMC2	ITGB1	LAMC2 - ITGB1	Laminins	6.45E-196	8.57E-114
Keratinocytes	MF17 Clonotype 1	CDH1	ITGB7	CDH1 - ITGB7	Cell-cell adhesion	0	0.018785255
Keratinocytes	MF17 Clonotype 1	JAG1	NOTCH1	JAG1 - NOTCH1	NOTCH	1.18E-222	0.009203694
Keratinocytes	MF17 Clonotype 1	LGALS3BP	VANGL1	LGALS3BP - VANGL1	UNCAT	2.48E-147	1.05E-08
T lymphocytes	MF17 Clonotype 1	CCL5	CCR4	CCL5 - CCR4	CC	7.12E-82	1.79E-10
T lymphocytes	MF17 Clonotype 1	B2M	CD247	B2M - CD247	UNCAT	3.64E-12	1.18E-20
T lymphocytes	MF17 Clonotype 1	B2M	CD3D	B2M - CD3D	UNCAT	3.64E-12	8.94E-63
T lymphocytes	MF17 Clonotype 1	HLA-A	CD3D	HLA-A - CD3D	MHC	1.36E-13	8.94E-63
T lymphocytes	MF17 Clonotype 1	HLA-A	CD3G	HLA-A - CD3G	MHC	1.36E-13	2.09E-38
T lymphocytes	MF17 Clonotype 1	B2M	CD3G	B2M - CD3G	UNCAT	3.64E-12	2.09E-38
T lymphocytes	MF17 Clonotype 1	B2M	TFRC	B2M - TFRC	Transferrin	3.64E-12	5.48E-08

F. MF18 Clonotype 1

source	target	ligand	receptor	pair	mode	p_val_adj.lig	p_val_adj.rec
MF18 Clonotype 1	MF18 Clonotype 1	TNFSF10	TNFRSF10B	TNFSF10 - TNFRSF10B	TNF	6.11E-195	5.78E-14
MF18 Clonotype 1	MF18 Clonotype 1	TNFSF4	TNFRSF4	TNFSF4 - TNFRSF4	TNF	1.74E-258	5.60E-41
MF18 Clonotype 1	MF18 Clonotype 1	CSF3	CSF1R	CSF3 - CSF1R	CSF	1.18E-247	3.10E-40
MF18 Clonotype 1	MF18 Clonotype 1	LGALS3BP	VANGL1	LGALS3BP - VANGL1	UNCAT	1.13E-253	2.01E-17
MF18 Clonotype 1	MF18 Clonotype 1	TNFSF10	TNFRSF10A	TNFSF10 - TNFRSF10A	TNF	6.11E-195	2.59E-38
MF18 Clonotype 1	MF18 Clonotype 1	TIMP1	CD63	TIMP1 - CD63	MMP	2.05E-256	4.29E-46
MF18 Clonotype 1	MF18 Clonotype 1	GZMB	PGRMC1	GZMB - PGRMC1	UNCAT	1.70E-87	6.97E-57
MF18 Clonotype 1	MF18 Clonotype 1	CSF2	CSF2RB	CSF2 - CSF2RB	CSF	4.51E-110	2.89E-104
MF18 Clonotype 1	MF18 Clonotype 1	GNAI2	DRD2	GNAI2 - DRD2	UNCAT	2.33E-90	5.63E-100
MF18 Clonotype 1	MF18 Clonotype 1	GNAI2	CXCR3	GNAI2 - CXCR3	UNCAT	2.33E-90	4.49E-109
Dendritic Cell/Macrophages	MF18 Clonotype 1	APOE	LRP8	APOE - LRP8	APOE	8.87E-193	1.86E-22
Dendritic Cell/Macrophages	MF18 Clonotype 1	MMP9	ITGB2	MMP9 - ITGB2	MMP	5.06E-172	1.03E-06
Dendritic Cell/Macrophages	MF18 Clonotype 1	IL18	IL18RAP	IL18 - IL18RAP	Interleukins	1.45E-239	1.98E-52
Dendritic Cell/Macrophages	MF18 Clonotype 1	IL18	CD48	IL18 - CD48	Interleukins	1.45E-239	6.65E-41
Dendritic Cell/Macrophages	MF18 Clonotype 1	CCL3	CCR1	CCL3 - CCR1	CC	1.04E-97	9.79E-63

Dendritic Cell/Macrophages	MF18 Clonotype 1	CCL13	CCR1	CCL13 - CCR1	CC	3.81E-130	9.79E-63
Dendritic Cell/Macrophages	MF18 Clonotype 1	CCL13	CXCR3	CCL13 - CXCR3	CC	3.81E-130	4.49E-109
Dendritic Cell/Macrophages	MF18 Clonotype 1	CXCL10	CXCR3	CXCL10 - CXCR3	CXCL	5.45E-157	4.49E-109
Dendritic Cell/Macrophages	MF18 Clonotype 1	CXCL9	CXCR3	CXCL9 - CXCR3	CXCL	4.33E-191	4.49E-109
Dendritic Cell/Macrophages	MF18 Clonotype 1	PTGS2	PTGDR2	PTGS2 - PTGDR2	UNCAT	4.36E-53	2.78E-284
Endothelial Cells	MF18 Clonotype 1	COL18A1	ITGA5	COL18A1 - ITGA5	Collagens	3.67E-203	0.004990343
Endothelial Cells	MF18 Clonotype 1	EFNA1	EPHB6	EFNA1 - EPHB6	Ephrins	2.87E-212	5.17E-29
Endothelial Cells	MF18 Clonotype 1	LAMC1	ITGA3	LAMC1 - ITGA3	Laminins	5.13E-172	1.86E-36
Endothelial Cells	MF18 Clonotype 1	LAMA5	ITGA3	LAMA5 - ITGA3	Laminins	1.68E-220	1.86E-36
Endothelial Cells	MF18 Clonotype 1	LAMA4	ITGA3	LAMA4 - ITGA3	Laminins	3.36E-221	1.86E-36
Endothelial Cells	MF18 Clonotype 1	THBS1	ITGA3	THBS1 - ITGA3	Matrix glycoproteins	1.80E-245	1.86E-36
Endothelial Cells	MF18 Clonotype 1	LAMB1	ITGA3	LAMB1 - ITGA3	Laminins	5.06E-237	1.86E-36
Endothelial Cells	MF18 Clonotype 1	CCL14	CCR1	CCL14 - CCR1	CC	0	9.79E-63
Endothelial Cells	MF18 Clonotype 1	THBS1	CD47	THBS1 - CD47	Matrix glycoproteins	1.80E-245	2.58E-53
Endothelial Cells	MF18 Clonotype 1	COL4A1	CD47	COL4A1 - CD47	Collagens	0	2.58E-53
Fibroblasts	MF18 Clonotype 1	COL1A1	ITGA5	COL1A1 - ITGA5	Collagens	9.02E-286	0.004990343
Fibroblasts	MF18 Clonotype 1	FN1	PLAUR	FN1 - PLAUR	Fibronectin	6.75E-260	3.22E-14
Fibroblasts	MF18 Clonotype 1	FN1	ITGA3	FN1 - ITGA3	Fibronectin	6.75E-260	1.86E-36
Fibroblasts	MF18 Clonotype 1	THBS2	CD47	THBS2 - CD47	Matrix glycoproteins	0	2.58E-53
Fibroblasts	MF18 Clonotype 1	FN1	NT5E	FN1 - NT5E	Fibronectin	6.75E-260	2.58E-158
Fibroblasts	MF18 Clonotype 1	TNC	NT5E	TNC - NT5E	UNCAT	0	2.58E-158
Fibroblasts	MF18 Clonotype 1	C3	IFITM1	C3 - IFITM1	Complement	9.22E-209	5.08E-95
Fibroblasts	MF18 Clonotype 1	EFEMP2	CATSPER1	EFEMP2 - CATSPER1	UNCAT	4.80E-270	2.08E-300
Keratinocytes	MF18 Clonotype 1	EFNA3	EPHB6	EFNA3 - EPHB6	Ephrins	1.61E-105	5.17E-29
Keratinocytes	MF18 Clonotype 1	EFNA1	EPHB6	EFNA1 - EPHB6	Ephrins	3.54E-115	5.17E-29
Keratinocytes	MF18 Clonotype 1	LAMC2	ITGA3	LAMC2 - ITGA3	Laminins	5.29E-139	1.86E-36
Keratinocytes	MF18 Clonotype 1	LAMB3	ITGA3	LAMB3 - ITGA3	Laminins	2.42E-112	1.86E-36
Keratinocytes	MF18 Clonotype 1	LAMC2	CD151	LAMC2 - CD151	Laminins	5.29E-139	4.15E-41
Keratinocytes	MF18 Clonotype 1	LAMB3	CD151	LAMB3 - CD151	Laminins	2.42E-112	4.15E-41
Keratinocytes	MF18 Clonotype 1	IL18	CD48	IL18 - CD48	Interleukins	1.14E-44	6.65E-41
Keratinocytes	MF18 Clonotype 1	COL4A5	CD47	COL4A5 - CD47	Collagens	1.92E-87	2.58E-53
Keratinocytes	MF18 Clonotype 1	HBEGF	CD9	HBEGF - CD9	EGF	1.22E-20	1.00E-145
Keratinocytes	MF18 Clonotype 1	NTF4	NTRK1	NTF4 - NTRK1	UNCAT	3.86E-147	2.59E-294
T lymphocytes	MF18 Clonotype 1	HLA-A	KIR3DL1	HLA-A - KIR3DL1	MHC	6.25E-90	4.74E-43
T lymphocytes	MF18 Clonotype 1	HLA-E	KIR3DL1	HLA-E - KIR3DL1	MHC	8.65E-104	4.74E-43
T lymphocytes	MF18 Clonotype 1	HLA-B	CD3D	HLA-B - CD3D	MHC	9.80E-62	6.45E-25
T lymphocytes	MF18 Clonotype 1	HLA-C	CD3D	HLA-C - CD3D	MHC	6.56E-58	6.45E-25
T lymphocytes	MF18 Clonotype 1	HLA-A	CD3D	HLA-A - CD3D	MHC	6.25E-90	6.45E-25
T lymphocytes	MF18 Clonotype 1	CCL4	CCR1	CCL4 - CCR1	CC	6.38E-17	9.79E-63
T lymphocytes	MF18 Clonotype 1	CCL5	CCR1	CCL5 - CCR1	CC	1.36E-104	9.79E-63
T lymphocytes	MF18 Clonotype 1	CXCL13	CXCR3	CXCL13 - CXCR3	CXCL	4.10E-34	4.49E-109

T lymphocytes	MF18 Clonotype 1	CCL5	CXCR3	CCL5 - CXCR3	CC	1.36E-104	4.49E-109

G. MF21 Clonotype 1

source	target	ligand	receptor	pair	mode	p_val_adj.lig	p_val_adj.rec
B Cells	MF21 Clonotype 1	HLA-C	CD3D	HLA-C - CD3D	MHC	0.020141973	7.53E-67
B Cells	MF21 Clonotype 1	HLA-C	CD3G	HLA-C - CD3G	MHC	0.020141973	3.77E-73
B Cells	MF21 Clonotype 1	ICAM2	ITGAL	ICAM2 - ITGAL	Cell-cell adhesion	0.000302199	1.29E-43
B Cells	MF21 Clonotype 1	ICAM3	ITGAL	ICAM3 - ITGAL	Cell-cell adhesion	1.15E-05	1.29E-43
B Cells	MF21 Clonotype 1	ICAM2	ITGB2	ICAM2 - ITGB2	Cell-cell adhesion	0.000302199	1.77E-34
B Cells	MF21 Clonotype 1	ICAM3	ITGB2	ICAM3 - ITGB2	Cell-cell adhesion	1.15E-05	1.77E-34
MF21 Clonotype 1	MF21 Clonotype 1	CCL22	CCR4	CCL22 - CCR4	CC	3.08E-12	0
MF21 Clonotype 1	MF21 Clonotype 1	B2M	CD247	B2M - CD247	UNCAT	1.26E-31	2.47E-139
MF21 Clonotype 1	MF21 Clonotype 1	BTLA	CD247	BTLA - CD247	TNF	2.11E-274	2.47E-139
MF21 Clonotype 1	MF21 Clonotype 1	CD70	CD27	CD70 - CD27	UNCAT	7.96E-122	3.07E-116
MF21 Clonotype 1	MF21 Clonotype 1	BTLA	CD79A	BTLA - CD79A	TNF	2.11E-274	3.95E-115
MF21 Clonotype 1	MF21 Clonotype 1	IGFL2	IGFLR1	IGFL2 - IGFLR1	IGF	2.00E-79	7.61E-54
MF21 Clonotype 1	MF21 Clonotype 1	CD40LG	ITGB2	CD40LG - ITGB2	UNCAT	1.11E-174	1.77E-34
MF21 Clonotype 1	MF21 Clonotype 1	HLA-A	KIR3DL2	HLA-A - KIR3DL2	MHC	1.27E-07	0
MF21 Clonotype 1	MF21 Clonotype 1	PTHLH	PTH2R	PTHLH - PTH2R	UNCAT	0	1.45E-187
MF21 Clonotype 1	MF21 Clonotype 1	TNFSF11	TNFRSF11B	TNFSF11 - TNFRSF11B	TNF	2.36E-235	3.67E-50
Dendritic Cell/Macrophages	MF21 Clonotype 1	CCL3	CCR4	CCL3 - CCR4	CC	4.36E-47	0
Dendritic Cell/Macrophages	MF21 Clonotype 1	CCL17	CCR4	CCL17 - CCR4	CC	1.08E-33	0
Dendritic Cell/Macrophages	MF21 Clonotype 1	CCL17	CCR8	CCL17 - CCR8	CC	1.08E-33	6.98E-100
Dendritic Cell/Macrophages	MF21 Clonotype 1	FN1	CD79A	FN1 - CD79A	Fibronectin	3.38E-51	3.95E-115
Dendritic Cell/Macrophages	MF21 Clonotype 1	ICAM1	IL2RA	ICAM1 - IL2RA	Cell-cell adhesion	2.87E-14	7.35E-161
Dendritic Cell/Macrophages	MF21 Clonotype 1	LYZ	ITGAL	LYZ - ITGAL	UNCAT	0	1.29E-43
Dendritic Cell/Macrophages	MF21 Clonotype 1	MMP9	ITGB2	MMP9 - ITGB2	MMP	6.57E-199	1.77E-34
Dendritic Cell/Macrophages	MF21 Clonotype 1	SELPLG	SELL	SELPLG - SELL	Cell-cell adhesion	4.04E-17	2.91E-301
Dendritic Cell/Macrophages	MF21 Clonotype 1	FN1	TNFRSF11B	FN1 - TNFRSF11B	Fibronectin	3.38E-51	3.67E-50
Dendritic Cell/Macrophages	MF21 Clonotype 1	TNFSF13	TNFRSF11B	TNFSF13 - TNFRSF11B	TNF	1.49E-100	3.67E-50
Endothelial Cells	MF21 Clonotype 1	CXCL12	CCR4	CXCL12 - CCR4	CC	0	0
Endothelial Cells	MF21 Clonotype 1	COL4A1	CD47	COL4A1 - CD47	Collagens	0	0.001080796
Endothelial Cells	MF21 Clonotype 1	CXCL12	CXCR3	CXCL12 - CXCR3	CXCL	0	2.78E-93
Endothelial Cells	MF21 Clonotype 1	ICAM2	ITGAL	ICAM2 - ITGAL	Cell-cell adhesion	2.62E-232	1.29E-43
Endothelial Cells	MF21 Clonotype 1	ICAM2	ITGB2	ICAM2 - ITGB2	Cell-cell adhesion	2.62E-232	1.77E-34
Endothelial Cells	MF21 Clonotype 1	HLA-A	KIR3DL2	HLA-A - KIR3DL2	MHC	8.82E-14	0
Endothelial Cells	MF21 Clonotype 1	CFH	SELL	CFH - SELL	Complement	5.86E-211	2.91E-301
Endothelial Cells	MF21 Clonotype 1	PODXL	SELL	PODXL - SELL	Cell-cell adhesion	5.56E-301	2.91E-301
Endothelial Cells	MF21 Clonotype 1	CD34	SELL	CD34 - SELL	Cell-cell adhesion	0	2.91E-301
Endothelial Cells	MF21 Clonotype 1	VWF	TNFRSF11B	VWF - TNFRSF11B	Vasoactive	2.81E-195	3.67E-50
Fibroblasts	MF21 Clonotype 1	CCL19	CCR7	CCL19 - CCR7	CC	2.36E-248	1.18E-267
Fibroblasts	MF21 Clonotype 1	CCL19	CXCR3	CCL19 - CXCR3	CC	2.36E-248	2.78E-93

Fibroblasts	MF21 Clonotype 1	COL1A1	FLT4	COL1A1 - FLT4	Collagens	5.78E-172	3.53E-18
Fibroblasts	MF21 Clonotype 1	COL1A2	FLT4	COL1A2 - FLT4	Collagens	0	3.53E-18
Fibroblasts	MF21 Clonotype 1	C3	IFITM1	C3 - IFITM1	Complement	0	4.71E-11
Fibroblasts	MF21 Clonotype 1	C3	ITGB2	C3 - ITGB2	Complement	0	1.77E-34
Fibroblasts	MF21 Clonotype 1	HLA-A	KIR3DL2	HLA-A - KIR3DL2	MHC	1.50E-08	0
Fibroblasts	MF21 Clonotype 1	DCN	MET	DCN - MET	MET	4.50E-150	2.23E-49
Fibroblasts	MF21 Clonotype 1	CFH	SELL	CFH - SELL	Complement	3.11E-74	2.91E-301
Fibroblasts	MF21 Clonotype 1	VCAN	SELL	VCAN - SELL	Cell-cell adhesion	1.00E-148	2.91E-301
Keratinocytes	MF21 Clonotype 1	CCL2	CCR4	CCL2 - CCR4	CC	0.000338301	0
Keratinocytes	MF21 Clonotype 1	HLA-B	CD3D	HLA-B - CD3D	MHC	4.04E-16	7.53E-67
Keratinocytes	MF21 Clonotype 1	CDH1	CDH2	CDH1 - CDH2	Cell-cell adhesion	2.67E-256	1.04E-104
Keratinocytes	MF21 Clonotype 1	FGF11	FGFR1	FGF11 - FGFR1	FGF	2.02E-46	2.40E-82
Keratinocytes	MF21 Clonotype 1	IGFL1	IGFLR1	IGFL1 - IGFLR1	IGF	1.46E-127	7.61E-54
Keratinocytes	MF21 Clonotype 1	LTF	TFRC	LTF - TFRC	Transferrin	5.55E-198	5.87E-45
Keratinocytes	MF21 Clonotype 1	TNFSF10	TNFRSF11B	TNFSF10 - TNFRSF11B	TNF	3.92E-126	3.67E-50
T lymphocytes	MF21 Clonotype 1	CCL5	CCR4	CCL5 - CCR4	CC	2.93E-195	0
T lymphocytes	MF21 Clonotype 1	CCL4	CCR8	CCL4 - CCR8	CC	7.06E-49	6.98E-100
T lymphocytes	MF21 Clonotype 1	CD70	CD27	CD70 - CD27	UNCAT	7.86E-17	3.07E-116
T lymphocytes	MF21 Clonotype 1	HLA-A	CD3D	HLA-A - CD3D	MHC	5.35E-14	7.53E-67
T lymphocytes	MF21 Clonotype 1	HLA-C	CD3D	HLA-C - CD3D	MHC	5.34E-10	7.53E-67
T lymphocytes	MF21 Clonotype 1	HLA-A	CD3G	HLA-A - CD3G	MHC	5.35E-14	3.77E-73
T lymphocytes	MF21 Clonotype 1	CCL5	CXCR3	CCL5 - CXCR3	CC	2.93E-195	2.78E-93
T lymphocytes	MF21 Clonotype 1	HLA-A	KIR3DL2	HLA-A - KIR3DL2	MHC	5.35E-14	0
T lymphocytes	MF21 Clonotype 1	SELPLG	SELL	SELPLG - SELL	Cell-cell adhesion	8.93E-12	2.91E-301

H. MF24 Clonotype 1

source	target	ligand	receptor	pair	mode	p_val_adj.lig	p_val_adj.rec
B Cells	MF24 Clonotype 1	GSTP1	TRAF2	GSTP1 - TRAF2	TNF	9.41E-67	0.00078291
B Cells	MF24 Clonotype 1	EBI3	IL27RA	EBI3 - IL27RA	UNCAT	1.49E-43	5.71E-14
B Cells	MF24 Clonotype 1	ICAM1	ITGB2	ICAM1 - ITGB2	Cell-cell adhesion	6.40E-11	3.72E-08
B Cells	MF24 Clonotype 1	IL16	KCNA3	IL16 - KCNA3	Interleukins	1.00E-06	3.67E-24
B Cells	MF24 Clonotype 1	CCL17	CCR4	CCL17 - CCR4	CC	1.23E-39	5.64E-25
B Cells	MF24 Clonotype 1	ICAM1	IL2RG	ICAM1 - IL2RG	Cell-cell adhesion	6.40E-11	7.48E-12
B Cells	MF24 Clonotype 1	EBI3	IL6ST	EBI3 - IL6ST	UNCAT	1.49E-43	1.07E-66
B Cells	MF24 Clonotype 1	IL6	IL6ST	IL6 - IL6ST	Interleukins	2.22E-95	1.07E-66
B Cells	MF24 Clonotype 1	IL6	IL6R	IL6 - IL6R	Interleukins	2.22E-95	1.41E-81
B Cells	MF24 Clonotype 1	SEMA7A	ITGB1	SEMA7A - ITGB1	Semaphorins	6.39E-21	7.03E-89
MF24 Clonotype 1	MF24 Clonotype 1	CD40LG	ITGB2	CD40LG - ITGB2	UNCAT	0	3.72E-08
MF24 Clonotype 1	MF24 Clonotype 1	IGFBP4	LRP6	IGFBP4 - LRP6	IGF	1.76E-163	4.53E-51
MF24 Clonotype 1	MF24 Clonotype 1	HBEGF	CD9	HBEGF - CD9	EGF	2.73E-24	6.93E-85
MF24 Clonotype 1	MF24 Clonotype 1	APP	GPC1	APP - GPC1	UNCAT	4.14E-94	2.79E-81
MF24 Clonotype 1	MF24 Clonotype 1	BTLA	CD247	BTLA - CD247	TNF	5.61E-44	2.66E-84

MF24 Clonotype 1	MF24 Clonotype 1	ADAM17	ITGB1	ADAM17 - ITGB1	ADAM	0.000131188	7.03E-89
MF24 Clonotype 1	MF24 Clonotype 1	TIMP2	ITGB1	TIMP2 - ITGB1	MMP	1.11E-18	7.03E-89
MF24 Clonotype 1	MF24 Clonotype 1	COL6A3	ITGB1	COL6A3 - ITGB1	Collagens	1.03E-24	7.03E-89
MF24 Clonotype 1	MF24 Clonotype 1	MDK	ITGB1	MDK - ITGB1	NEGF	6.43E-26	7.03E-89
MF24 Clonotype 1	MF24 Clonotype 1	LRPAP1	SORL1	LRPAP1 - SORL1	Intracellular trafficking	0.001099076	4.87E-150
Dendritic Cell/Macrophages	MF24 Clonotype 1	MMP9	ITGB2	MMP9 - ITGB2	MMP	1.04E-63	3.72E-08
Dendritic Cell/Macrophages	MF24 Clonotype 1	PLAU	ITGB2	PLAU - ITGB2	Vasoactive	3.20E-123	3.72E-08
Dendritic Cell/Macrophages	MF24 Clonotype 1	PLAU	PLAUR	PLAU - PLAUR	Vasoactive	3.20E-123	4.43E-20
Dendritic Cell/Macrophages	MF24 Clonotype 1	MMP12	PLAUR	MMP12 - PLAUR	MMP	1.33E-230	4.43E-20
Dendritic Cell/Macrophages	MF24 Clonotype 1	CCL17	CCR4	CCL17 - CCR4	CC	3.19E-101	5.64E-25
Dendritic Cell/Macrophages	MF24 Clonotype 1	CD14	ITGB1	CD14 - ITGB1	UNCAT	2.67E-122	7.03E-89
Dendritic Cell/Macrophages	MF24 Clonotype 1	PLAU	ITGB1	PLAU - ITGB1	Vasoactive	3.20E-123	7.03E-89
Dendritic Cell/Macrophages	MF24 Clonotype 1	F13A1	ITGB1	F13A1 - ITGB1	UNCAT	3.21E-250	7.03E-89
Dendritic Cell/Macrophages	MF24 Clonotype 1	PLAU	MRC2	PLAU - MRC2	Vasoactive	3.20E-123	1.27E-168
Dendritic Cell/Macrophages	MF24 Clonotype 1	APOE	SORL1	APOE - SORL1	APOE	1.21E-48	4.87E-150
Endothelial Cells	MF24 Clonotype 1	HSPG2	PTPRS	HSPG2 - PTPRS	Matrix glycoproteins	0	3.86E-46
Endothelial Cells	MF24 Clonotype 1	COL4A1	CD47	COL4A1 - CD47	Collagens	0	1.98E-46
Endothelial Cells	MF24 Clonotype 1	COL18A1	GPC1	COL18A1 - GPC1	Collagens	2.09E-105	2.79E-81
Endothelial Cells	MF24 Clonotype 1	LAMA4	ITGB1	LAMA4 - ITGB1	Laminins	0	7.03E-89
Endothelial Cells	MF24 Clonotype 1	LAMA5	ITGB1	LAMA5 - ITGB1	Laminins	2.38E-206	7.03E-89
Endothelial Cells	MF24 Clonotype 1	LAMC1	ITGB1	LAMC1 - ITGB1	Laminins	5.21E-183	7.03E-89
Endothelial Cells	MF24 Clonotype 1	COL18A1	ITGB1	COL18A1 - ITGB1	Collagens	2.09E-105	7.03E-89
Endothelial Cells	MF24 Clonotype 1	LAMB1	ITGB1	LAMB1 - ITGB1	Laminins	1.41E-290	7.03E-89
Endothelial Cells	MF24 Clonotype 1	COL4A1	ITGB1	COL4A1 - ITGB1	Collagens	0	7.03E-89
Endothelial Cells	MF24 Clonotype 1	HSPG2	ITGB1	HSPG2 - ITGB1	Matrix glycoproteins	0	7.03E-89
Fibroblasts	MF24 Clonotype 1	C3	CD46	C3 - CD46	Complement	0	2.52E-09
Fibroblasts	MF24 Clonotype 1	C3	ITGB2	C3 - ITGB2	Complement	0	3.72E-08
Fibroblasts	MF24 Clonotype 1	C3	IFITM1	C3 - IFITM1	Complement	0	3.35E-05
Fibroblasts	MF24 Clonotype 1	TNC	ITGB1	TNC - ITGB1	UNCAT	5.80E-206	7.03E-89
Fibroblasts	MF24 Clonotype 1	COL1A1	ITGB1	COL1A1 - ITGB1	Collagens	4.67E-51	7.03E-89
Fibroblasts	MF24 Clonotype 1	COL6A2	ITGB1	COL6A2 - ITGB1	Collagens	3.75E-45	7.03E-89
Fibroblasts	MF24 Clonotype 1	COL6A1	ITGB1	COL6A1 - ITGB1	Collagens	4.68E-97	7.03E-89
Fibroblasts	MF24 Clonotype 1	COL1A2	ITGB1	COL1A2 - ITGB1	Collagens	0	7.03E-89
Fibroblasts	MF24 Clonotype 1	COL3A1	ITGB1	COL3A1 - ITGB1	Collagens	0	7.03E-89
Fibroblasts	MF24 Clonotype 1	APOE	SORL1	APOE - SORL1	APOE	2.20E-100	4.87E-150
Keratinocytes	MF24 Clonotype 1	GSTP1	TRAF2	GSTP1 - TRAF2	TNF	7.92E-23	0.00078291
Keratinocytes	MF24 Clonotype 1	CDH1	ITGAE	CDH1 - ITGAE	Cell-cell adhesion	4.55E-136	0.00022929
Keratinocytes	MF24 Clonotype 1	SLPI	CD4	SLPI - CD4	UNCAT	8.23E-246	4.32E-18
Keratinocytes	MF24 Clonotype 1	COL4A5	CD47	COL4A5 - CD47	Collagens	1.45E-193	1.98E-46
Keratinocytes	MF24 Clonotype 1	THBS2	CD47	THBS2 - CD47	Matrix glycoproteins	3.83E-187	1.98E-46
Keratinocytes	MF24 Clonotype 1	COL4A5	ITGB1	COL4A5 - ITGB1	Collagens	1.45E-193	7.03E-89

Keratinocytes	MF24 Clonotype 1	THBS2	ITGB1	THBS2 - ITGB1	Matrix glycoproteins	3.83E-187	7.03E-89
Keratinocytes	MF24 Clonotype 1	LGALS3BP	ITGB1	LGALS3BP - ITGB1	UNCAT	3.70E-82	7.03E-89
Keratinocytes	MF24 Clonotype 1	LAMB3	ITGB1	LAMB3 - ITGB1	Laminins	3.97E-117	7.03E-89
Keratinocytes	MF24 Clonotype 1	COL7A1	ITGB1	COL7A1 - ITGB1	Collagens	0	7.03E-89
T lymphocytes	MF24 Clonotype 1	CCL5	CCR4	CCL5 - CCR4	CC	1.15E-93	5.64E-25
T lymphocytes	MF24 Clonotype 1	TGFB1	CD109	TGFB1 - CD109	TGFB	0.003326219	1.67E-25
T lymphocytes	MF24 Clonotype 1	HLA-A	CD3D	HLA-A - CD3D	MHC	8.86E-44	3.28E-45
T lymphocytes	MF24 Clonotype 1	HLA-C	CD3D	HLA-C - CD3D	MHC	2.94E-42	3.28E-45
T lymphocytes	MF24 Clonotype 1	HLA-B	CD3D	HLA-B - CD3D	MHC	9.31E-50	3.28E-45
T lymphocytes	MF24 Clonotype 1	HLA-A	KIR3DL2	HLA-A - KIR3DL2	MHC	8.86E-44	6.41E-283

Table S7. Antibodies used in multi-color immunofluorescent microscopy.

Antibody	Company	Clone	Species
**CD20**	CELLSIGNAL	E7B7T	Rabbit
**CD138 (SDC1)**	ABCAM	EPR6454	Rabbit
**FPR2**	THERMO	Polyclonal	Rabbit
**FCN1**	THERMO	Polyclonal	Rabbit
**CLEC4C**	SIGMA	Polyclonal	Rabbit
**LILRA4**	THERMO	Polyclonal	Rabbit
**TNC**	ABCAM	EPR4219	Rabbit
**S100A8 (MRP8)**	ABCAM	EPR3554	Rabbit
**KRT6A**	ABCAM	EPR1603Y	Rabbit
**SMA**	DAKO	1A4	Mouse
**SOX18**	THERMO	Polyclonal	Rabbit
**CD31**	NOVUS	Polyclonal	Rabbit
**ITGB1**	THERMO	Polyclonal	Rabbit
**CD14**	THERMO	Polyclonal	Rabbit
**TOX**	SIGMA	Polyclonal	Rabbit
**CD3**	THERMO	F7.2.38	Mouse

Table S8. Proportion of different cell types in patient and healthy control samples.

A. Proportion of all cell types in MF and HC samples combined (Figure 1E, main text).

Cell type	MF6	MF8	MF12	MF17	MF18	MF21	MF24	HC1	HC2	HC3	HC4	HC5	HC6	HC7	HC8	HC9
Lymphocytes	44.90%	10.09%	48.97%	93.05%	75.62%	29.23%	82.68%	1.91%	3.39%	2.58%	2.82%	8.87%	3.68%	4.21%	8.70%	4.87%
Fibroblasts	2.80%	6.71%	3.68%	1.31%	2.59%	1.75%	0.22%	12.14%	43.66%	24.03%	19.12%	52.90%	64.82%	35.09%	50.35%	40.07%
Myeloid cells	32.66%	6.49%	21.62%	2.04%	12.39%	13.13%	3.56%	2.05%	3.77%	2.68%	2.55%	7.72%	3.68%	7.52%	9.31%	4.87%
Keratinocytes	6.84%	70.26%	14.72%	1.01%	2.05%	41.25%	0.80%	21.08%	14.26%	36.58%	41.14%	11.06%	10.39%	38.97%	9.93%	15.76%
Endothelial Cells	3.46%	3.04%	6.73%	0.16%	3.16%	5.53%	0.75%	19.07%	9.56%	13.51%	8.27%	6.60%	5.80%	5.88%	7.56%	12.54%
Pericytes	2.59%	1.22%	2.06%	0.12%	0.66%	1.49%	0.34%	22.40%	10.11%	11.76%	17.03%	9.90%	5.65%	4.06%	5.62%	10.89%
Epithelial/Secretory cells	0.18%	0.11%	0.20%	0.00%	0.00%	5.33%	0.05%	8.32%	3.06%	2.81%	3.09%	0.08%	0.38%	1.67%	4.04%	3.98%
B Cells	5.97%	0.26%	1.06%	2.32%	3.30%	1.58%	11.42%	0.03%	0.05%	0.00%	0.00%	0.12%	0.15%	0.00%	0.00%	0.03%
Smooth Muscle cells	0.13%	0.04%	0.00%	0.00%	0.00%	0.00%	0.00%	6.97%	2.79%	2.05%	3.35%	0.60%	3.37%	0.81%	1.41%	2.95%
Schwann cells	0.08%	0.06%	0.07%	0.00%	0.00%	0.03%	0.00%	5.55%	5.96%	1.95%	2.17%	0.80%	1.02%	1.04%	1.58%	3.01%
Mast cells	0.24%	0.83%	0.70%	0.00%	0.17%	0.03%	0.19%	0.17%	2.46%	1.11%	0.30%	1.19%	0.68%	0.27%	1.05%	0.30%
Melanocytes	0.16%	0.90%	0.20%	0.00%	0.06%	0.65%	0.00%	0.31%	0.93%	0.94%	0.15%	0.16%	0.38%	0.48%	0.44%	0.74%

B. Proportion of myeloid cells from individual MF and HC samples within each cluster (Figure 2D, main text).

Cluster	MF6	MF8	MF12	MF17	MF18	MF21	MF24	HC1	HC2	HC3	HC4	HC5	HC6	HC7	HC8	HC9
0	0.2%	0.4%	0.4%	0.2%	0.0%	0.0%	0.0%	3.4%	6.5%	5.9%	3.1%	21.7%	10.9%	21.0%	9.7%	16.8%
1	13.1%	32.0%	17.7%	0.3%	2.0%	18.0%	3.4%	1.2%	0.6%	3.2%	2.5%	2.0%	1.3%	1.8%	0.2%	0.9%
2	71.5%	5.6%	9.0%	1.6%	10.4%	0.5%	0.3%	0.1%	0.0%	0.0%	0.0%	0.1%	0.1%	0.1%	0.0%	0.8%
3	1.3%	2.7%	8.2%	0.2%	0.9%	0.2%	11.0%	4.2%	6.4%	10.2%	6.8%	9.1%	3.3%	20.1%	7.7%	7.7%
4	24.5%	2.5%	24.8%	3.8%	20.0%	16.6%	3.0%	0.5%	0.1%	0.2%	0.1%	0.2%	0.9%	1.5%	0.7%	0.8%
5	41.6%	2.7%	11.9%	2.3%	12.3%	4.6%	2.7%	0.9%	0.5%	2.3%	0.9%	5.0%	0.9%	4.6%	1.8%	5.0%
6	61.5%	0.0%	19.5%	0.6%	10.3%	0.0%	6.9%	0.0%	0.0%	0.0%	0.0%	0.0%	0.0%	0.6%	0.6%	0.0%
7	29.3%	14.6%	3.3%	8.9%	2.4%	15.5%	8.1%	0.8%	0.0%	0.0%	0.0%	7.3%	0.0%	4.1%	3.3%	2.4%
8	17.5%	11.3%	18.6%	2.1%	14.4%	20.6%	3.1%	3.1%	0.0%	0.0%	0.0%	2.1%	0.0%	1.0%	1.0%	5.2%

C. Proportion of keratinocytes from individual MF and HC samples within each cluster (Figure 4C, main text).

Cluster	MF6	MF8	MF12	MF17	MF18	MF21	MF24	HC1	HC2	HC3	HC4	HC5	HC6	HC7	HC8	HC9
0	0.1%	0.1%	0.2%	0.0%	0.0%	0.2%	0.0%	4.7%	3.4%	22.6%	24.5%	6.8%	6.4%	24.2%	1.7%	4.9%
1	5.2%	60.8%	6.4%	0.6%	0.7%	25.7%	0.4%	0.0%	0.0%	0.0%	0.0%	0.1%	0.0%	0.0%	0.0%	0.0%
2	3.4%	10.7%	0.1%	0.1%	1.0%	2.9%	0.4%	2.6%	2.9%	19.2%	3.9%	2.0%	2.3%	19.2%	2.0%	27.4%
3	0.1%	0.2%	0.4%	0.0%	0.0%	0.0%	0.1%	17.1%	4.4%	27.3%	16.2%	2.9%	1.8%	24.7%	1.2%	3.5%
4	0.8%	69.0%	9.7%	1.3%	2.0%	15.9%	1.0%	0.1%	0.0%	0.0%	0.0%	0.0%	0.0%	0.0%	0.0%	0.2%
5	0.8%	2.1%	2.3%	0.0%	0.4%	1.9%	0.1%	19.0%	7.6%	24.8%	10.2%	1.9%	5.5%	5.0%	3.3%	15.0%
6	0.5%	73.0%	8.7%	0.5%	1.8%	6.4%	0.3%	3.3%	1.5%	1.0%	0.5%	0.4%	0.1%	1.2%	0.1%	0.7%

D. Proportion of B cells from individual MF and HC samples within each cluster (Figure 3E, main text).

Cluster	MF6	MF8	MF12	MF17	MF18	MF21	MF24	HC1	HC2	HC3	HC4	HC5	HC6	HC7	HC8	HC9
0	0.5%	0.4%	0.0%	4.0%	15.3%	1.8%	78.0%	0.0%	0.0%	0.0%	0.0%	0.0%	0.0%	0.0%	0.5%	0.4%
1	82.7%	4.4%	9.7%	0.0%	2.8%	0.0%	0.0%	0.4%	0.0%	0.0%	0.0%	0.0%	0.0%	0.0%	82.7%	4.4%
2	8.9%	0.5%	3.9%	37.4%	10.3%	19.2%	15.3%	0.0%	0.5%	1.5%	2.0%	0.0%	0.0%	0.5%	8.9%	0.5%
3	62.0%	0.0%	19.3%	0.6%	11.1%	0.0%	5.8%	0.0%	0.0%	0.0%	0.0%	0.6%	0.6%	0.0%	62.0%	0.0%

E. Proportion of fibroblasts from individual MF and HC samples within each cluster (Figure S4B, Supplementary Materials).

Cluster	MF6	MF8	MF12	MF17	MF18	MF21	MF24	HC1	HC2	HC3	HC4	HC5	HC6	HC7	HC8	HC9
0	0.03%	0.14%	0.00%	0.10%	0.14%	0.03%	0.03%	2.47%	1.74%	5.29%	1.88%	10.12%	26.33%	20.31%	13.57%	17.81%
1	0.00%	0.05%	0.21%	0.00%	0.05%	0.00%	0.05%	3.60%	14.25%	11.16%	13.48%	9.26%	21.91%	10.39%	3.60%	11.99%
2	0.00%	0.11%	0.11%	0.00%	0.00%	0.00%	0.05%	3.36%	5.86%	6.98%	6.98%	26.65%	14.61%	11.62%	4.32%	19.35%
3	0.00%	0.11%	0.06%	0.00%	0.06%	0.11%	0.00%	8.17%	20.25%	25.07%	3.06%	6.75%	13.27%	8.39%	1.59%	13.10%
4	12.90%	42.75%	12.78%	6.51%	10.44%	6.27%	0.74%	0.25%	0.61%	0.98%	0.12%	1.97%	0.61%	1.72%	0.12%	1.23%
5	0.00%	0.38%	0.00%	0.00%	0.00%	0.00%	0.00%	0.00%	0.00%	0.00%	0.00%	85.82%	5.36%	3.83%	1.15%	3.45%

F. Proportion of endothelial cells from individual MF and HC samples within each cluster (Figure S5B, Supplementary Materials).

Cluster	MF6	MF8	MF12	MF17	MF18	MF21	MF24	HC1	HC2	HC3	HC4	HC5	HC6	HC7	HC8	HC9
0	1.57%	2.62%	3.14%	0.00%	0.13%	0.13%	0.65%	26.57%	9.42%	19.24%	8.77%	6.41%	4.32%	4.84%	5.24%	6.94%
1	0.14%	0.84%	0.14%	0.00%	0.00%	0.00%	0.14%	14.07%	3.06%	34.68%	10.17%	6.55%	7.52%	9.47%	1.39%	11.84%
2	1.17%	1.46%	1.90%	0.29%	0.00%	0.15%	0.00%	26.79%	7.03%	15.81%	6.15%	3.22%	3.95%	3.95%	1.61%	26.50%
3	15.59%	16.20%	23.30%	0.62%	14.81%	23.77%	3.40%	0.00%	0.00%	0.93%	0.15%	0.00%	0.00%	0.62%	0.15%	0.46%
4	3.57%	9.38%	4.02%	0.00%	3.57%	2.23%	0.45%	8.48%	8.48%	2.23%	6.70%	16.96%	14.73%	8.93%	4.46%	5.80%
5	0.00%	0.00%	0.00%	0.00%	4.08%	6.12%	0.68%	2.72%	1.36%	4.08%	2.04%	2.72%	2.72%	24.49%	6.12%	42.86%
6	0.74%	0.00%	3.70%	0.74%	0.00%	0.74%	0.74%	29.63%	8.89%	9.63%	11.85%	4.44%	1.48%	3.70%	3.70%	20.00%
